# Interplay of Monosaccharide Configurations on the Deacetylation with *Candida antarctica* Lipase-B

**DOI:** 10.1021/acs.joc.4c02582

**Published:** 2024-12-30

**Authors:** Kaarel Erik Hunt, Annette Miller, Kristin Liias, Tatsiana Jarg, Kadri Kriis, Tõnis Kanger

**Affiliations:** Department of Chemistry and Biotechnology, Tallinn University of Technology, Akadeemia tee 15, 12618 Tallinn, Estonia

## Abstract

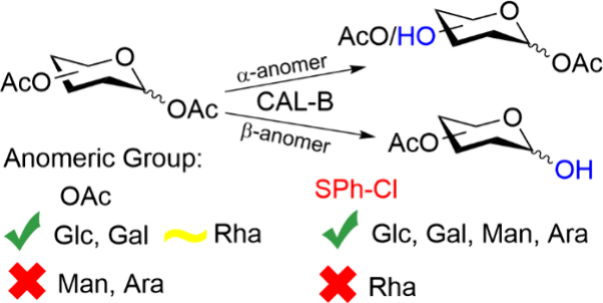

Configurational differences in monosaccharides determine the products and selectivity of the transesterification reaction with *Candida antarctica* lipase-B (CAL-B). The β-anomers of peresterified pyranose monosaccharides tend to yield anomeric deprotection products, while the α-anomers preferentially react at the sixth or fourth position. CAL-B differentiates between enantiomers, either reacting more rapidly with d-enantiomers of monosaccharides or having a different selectivity based on the enantiomer. Pivaloylated and benzoylated saccharides are the limits of the CAL-B transesterification reaction, while lower boiling point alcohols such as MeOH and EtOH can replace *n*-BuOH as the nucleophilic reagent. Finally, CAL-B can be successfully recycled in both long and short reaction time reactions.

## Introduction

Naturally occurring oligosaccharides, their derivatives, and glycoconjugates possess glycosidic bonds in specific positions and with particular stereochemistry. Consequently, the total synthesis of natural carbohydrate-containing compounds typically involves the use and transformations of several different protecting groups in multistep synthesis to enable the formation of glycosidic bonds at the precise positions and with specific stereochemistry.^1−5^

**Scheme 1:**
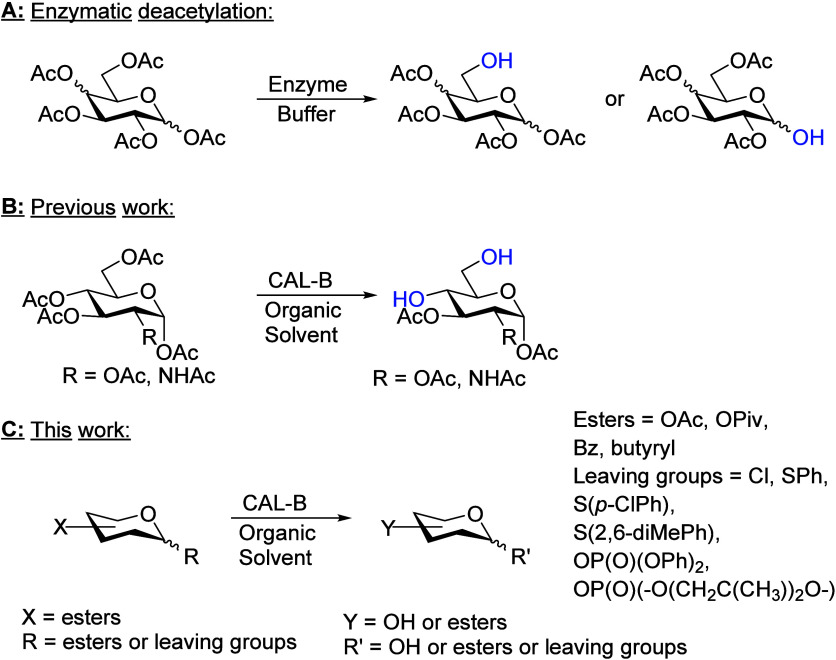
(A) Enzymes Have Been Used to Deprotect Mainly the First or the Sixth Position of Pyranose Monosaccharides; (B) Previously, We Have Shown That CAL-B in Organic Media Deprotects Mainly the Fourth and the Sixth Position in d-Glucopyranose-Based Monosaccharides; (C) in This Work, Several Different Protecting Groups Were Used, and the Influence of Configuration on the Deacetylation Reaction Were Investigated

To minimize the number of different protecting groups and thereby reduce the number of steps required to achieve selectively deprotected carbohydrates, several methods are available. Chemical procedures with pyranose monosaccharides primarily affect the first and/or the sixth positions, as these are the most easily manipulated due to their reactivity, compared to other positions.^6^ However, for both positions, there are several drawbacks to chemical processes like overreaction leading to a mixture of products,^7−9^ protecting group migration,^10,11^ very harsh conditions, or dangerous reagents.^12−14^ Enzymatic methods generally employ mild conditions and avoid hazardous chemicals, although they can also suffer from overreaction. Similar to chemical methods, enzymes preferably also target the first and the sixth positions (Scheme 1A).^15^ Enzymes are mostly used in aqueous buffer solutions,^15^ which introduces issues such as solubility of starting materials/products, complex workup procedures due to aqueous and mixed solvents, and relatively narrow conditions for the enzyme activity.^16^

It has been shown that *Candida antarctica* lipase-B (CAL-B) can overcome some of the problems previously listed as it can work in organic solvents,^17^ regains its activity in a wide temperature range,^18,19^ and, when immobilized, facilitates relatively easy workup procedures.^20,21^ Ester groups are substrates for hydrolytic enzymes, are commonly used to protect hydroxyl groups in carbohydrates, and are thus common in chemical and enzymatic methods of synthesis. We have previously shown that immobilized CAL-B Novozyme N435 can deacetylate a variety of acetyl-protected pyranose saccharides, with a general preference for deacetylation at the fourth and sixth positions in d-glucose-based compounds (d-glucose (d-Glc), d-glucosamine, and d-lactose (Lac)) (Scheme 1B).^20^

This work focuses on investigating how different core structures of carbohydrates influence the activity of CAL-B, how anomeric substituents and their configuration affect the catalytic activity, and the limitations of CAL-B’s tolerance for various protecting groups (Scheme 1C). The differences in configurations were tested on different pyranoses: d/l-Glc pentaacetate, its epimers d-galactose (Gal) and α-d-mannose pentaacetates, and peracetylated d/l-arabinose (Ara) enantiomers and l-rhamnose (Rha). Although the thiophenol leaving group (SPh) in the anomeric position is tolerated by CAL-B, the transesterification reaction takes 2 days with increased temperature and amounts of enzyme.^20^ Therefore, other substituents in the anomeric position: chloride, aliphatic cyclic phosphate esters, diarylphosphate esters, and thiophenol derivates, were also tested. Several bulkier protecting groups such as benzoyl (Bz), pivaloyl (Piv), and *n*-butyryl (But) esters for CAL-B were used to determine the enzyme’s limitations. Different alcohol nucleophiles were checked to replace *n*-butanol (*n*-BuOH) and concurringly the side product of *n*-butyl acetate with one that has a lower boiling point. Finally, immobilized CAL-B’s recyclability was tested in two different reactions with varying reaction times.

## Results and Discussion

### Anomeric Position

**Scheme 2:**
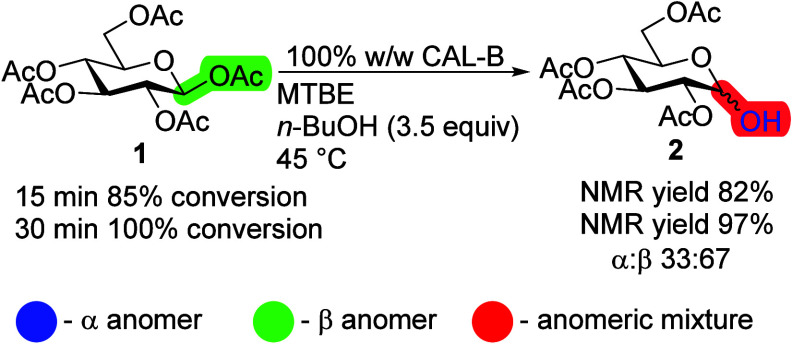
Transesterification Reaction with β-d-Glc Pentaacetate **1** with CAL-B Below the reaction is the color code used throughout the article: anomeric substituents highlighted in blue are α-anomers, green are the β-anomers, and red corresponds to the α/β-mixture; reaction conditions: β-d-Glc **1** pentaacetate (100 mg), 100% CAL-B (w/w), *n*-BuOH (3.5 equiv), MTBE (10 mL), 45 °C.

Transesterification reaction with β-d-Glc pentaacetate **1**, immobilized CAL-B (w/w saccharide/CAL-B; Novozyme N435), and *n*-BuOH in methyl *tert*-butyl ether (MTBE) was complete in 30 min, resulting in the selectively deprotected first position (Scheme 2, Figure S1). Please note that the w/w ratio was used to determine only the selectivity pattern of the reaction. To compare the reactions of saccharides with different molecular weights, the ratio of molar mass to enzyme activity units should be used. Based on these and previous results,^20^ we have shown that CAL-B targets different positions depending on the anomer of d-Glc pentaacetate with a higher catalytic activity toward β-d-Glc pentaacetate.

**Scheme 3:**
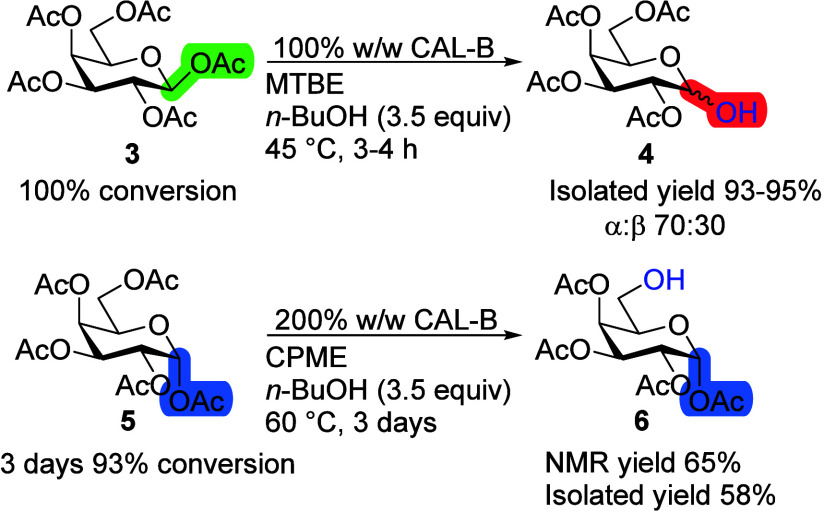
Difference in Regioselectivity for d-Gal Anomers Reaction conditions: monosaccharide (100 mg), 100% or 200% CAL-B (w/w), *n*-BuOH (3.5 equiv), MTBE or CPME (10 mL), 45 or 60 °C.

Similar to d-Glc, the β-anomer of d-Gal pentaacetate **3** reacts much faster than its α-anomer **5**, achieving full conversion within 3–4 h with a very high selectivity toward the main product of the first position deacetylated **4**. In contrast, the α-anomer **5** requires 3 days of reaction time, an increased amount of immobilized enzyme, cyclopentyl methyl ether (CPME) as a solvent, and an elevated temperature (60 °C) (Scheme 3). CAL-B has shown similar catalytic activity in CPME compared to MTBE.^20^ As such, when increased temperatures were used, CPME was used interchangeably with MTBE. The isolated yield of the sixth position deacetylated Gal tetraacetate **6** was 58%, with no single clear main side product, but rather multiple further deacetylated products. Once again, CAL-B shows a preference for different positions depending on the Gal pentaacetate anomeric configuration.

### Anomeric Substituents

**Scheme 4:**
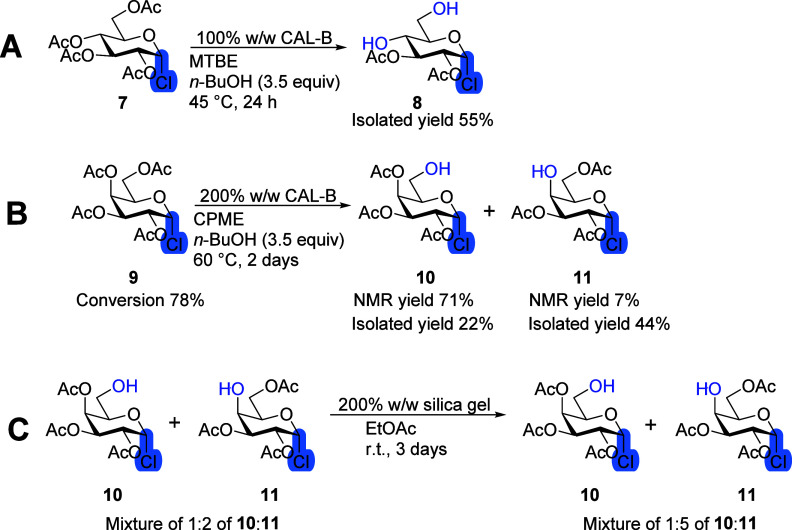
Influence of the Chloride Leaving Group on CAL-B Transesterification Reaction: (A) Reaction of α-d-Glc-Cl Tetraacetate **7** with CAL-B; (B) α-d-Gal-Cl Tetraacetate **9** with CAL-B; NMR yields for Products **10** and **11** Are from the Crude Reaction Mixture Being Heavily in Favor of Product **10**, while after Purification and Isolation, Product **11** Appeared as the Main Product; (C) Mixture of Products Was Stirred with Silica Gel to Check Its Influence on the Acyl Migration, Reaction Conditions: Monosaccharide (100 mg), 100% or 200% CAL-B (w/w), *n*-BuOH (3.5 equiv), MTBE or CPME (10 mL), 45 or 60 °C; Acyl Migration: Mixture of **10** and **11** (30 mg), Silica Gel (60 mg), EtOAc (1 mL), Room Temperature

**Figure 1:**
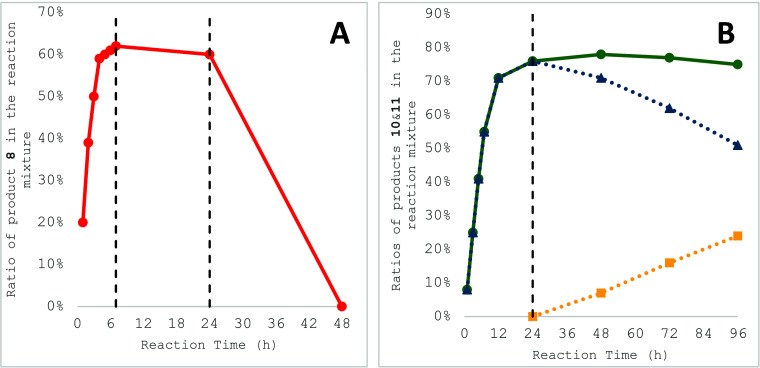
(A) CAL-B transesterification reactions with d-Glc-Cl tetraacetate **7**. The red circles show the NMR yield of product **8**. The three steps of the transesterification reaction profile are separated by dashed lines. Reaction conditions: d-Glc-Cl tetraacetate **7** (100 mg), 100% CAL-B (w/w), *n*-BuOH (3.5 equiv), MTBE (10 mL), and 45 °C. (B) CAL-B transesterification reaction with d-Gal-Cl tetraacetate **9**. The blue triangles show the NMR yield of product **10**, yellow squares show the NMR yield of product **11**, and green circles are the sum of the two product NMR yields. The two steps of the transesterification reaction are separated by a dashed line. Reaction conditions: d-Gal-Cl tetraacetate **9** (100 mg), 200% CAL-B (w/w), *n*-BuOH (3.5 equiv), CPME (10 mL), and 60 °C.

The reaction of α-d-Glc-Cl tetraacetate **7** with CAL-B (Scheme 4A) proceeded similar to peracetylated α-d-Glc **42** with one major product of fourth and sixth positions deacetylated **8**. After 7 h, the conversion reached approximately 62% according to ^1^H NMR. After 24 h, the conversion remained the same with small amounts (<5%) of side products appearing. After 48 h, product **8** was no longer detected by NMR and several overreaction products were observed, while approximately 38% of the unreacted starting material remained in the reaction mixture. The transesterification reaction has stopped after 7 h, indicating that the formed product **8** inhibits the access of the starting material **7** to the catalytic pocket of CAL-B. The observed progress of the initial slow formation of overreaction products (<5% in 17 h) followed by a rapid increase in overreaction and then full conversion from **8** in 24 h matches well with the product activation reaction characteristics.^22^ It is supposed that the reaction profile of d-Glc-Cl **7** with CAL-B consists of three steps: an initial selective transesterification, followed by the inhibition and a lag phase, and finally a second nonselective transesterification (Figure 1A). The reaction of d-Gal-Cl tetraacetate **9** with CAL-B has the same regioselectivity with d-Gal pentaacetate **5** (Scheme S1), while the reaction profile was similar to that of d-Glc-Cl tetraacetate **7**. The first step, initial selective transesterification, happened during 24 h and afforded the sixth position deacetylated product **10** in 76% NMR yield (Scheme 4B, Figure 1B). The second step, the inhibition and lag phase, started after 24 h. It continued for the 4 days during which the reaction was followed as only <5% overreaction products were detected by NMR. The second step was accompanied by acyl migration, which might have inhibited any further transesterification and prolonged the second step. After 48 h, the fourth position deprotected product **11** was detected, and by the fourth day, its NMR yield had increased to 24%. Following purification by column chromatography on silica gel, the overall ratio of products **10**:**11** had changed to 1:2. To prove that acyl migration continues during column chromatography, a portion of the isolated mixture of products was stirred with 200% silica gel (w/w) for 3 days. The acyl migration was carried out successfully, resulting in a final ratio of 1:5 in favor of tetraacetate **11**. Prolonged stirring with a higher amount of 1000% silica (w/w) led to decomposition.

**Scheme 5:**
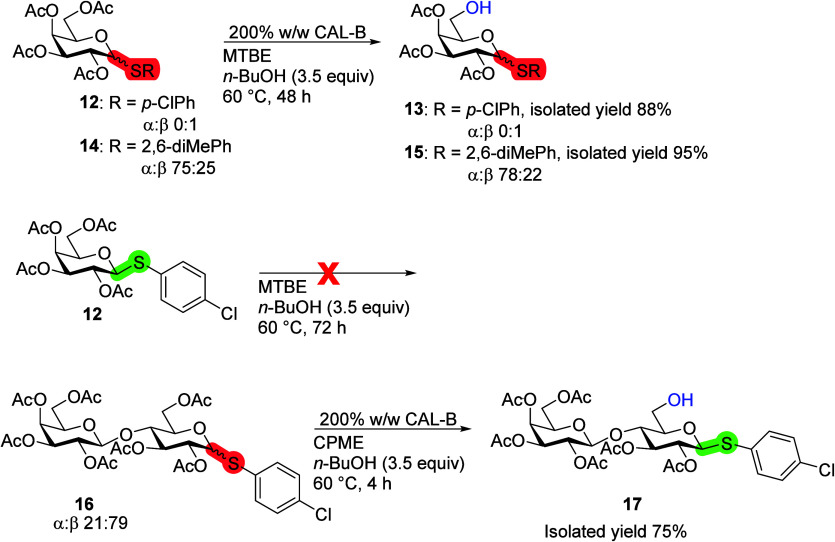
Influence of Different Thiophenol Derivatives on the CAL-B Deacetylation Reaction Blank reaction, where no enzyme was added, showed no reactivity; reaction conditions: saccharide (100 mg), 200% CAL-B (w/w), *n*-BuOH (3.5 equiv), MTBE or CPME (10 mL), 60 °C.

Thioglycoside **12**, equipped with a *p*-chlorothiophenol leaving group, was selectively deacetylated in the sixth position, yielding product **13** in 88% yield in 48 h (Scheme 5). Compared to a thiophenol leaving group, there was a slight increase in the yield, but the reaction time remained 48 h.^20^*p*-Chlorothiophenol is a solid and less foul-smelling compared to thiophenol; as such, other carbohydrates of interest were used with *p*-chlorothiophenol as the sulfur-containing leaving group instead of thiophenol due to its practical advantages. A blank reaction of thioglycoside **12** and *n*-BuOH was checked in the absence of an enzyme. There was no reaction with thioglycoside **12** in 72 h at 60 °C according to ^1^H NMR. The anomeric mixture of peracetylated d-Gal with the 2,6-dimethylthiophenol leaving group **14** reacted similar to previously mentioned thioglycosides, producing the sixth position deacetylated product **15**. After 48 h, close to full conversion was reached, no clear anomeric preference was seen, very selective with only an unreacted starting material leftover, and even higher yield was achieved. Peracetylated d-Lac has been shown to undergo deacetylation in the sixth position of the d-Glc ring with CAL-B. This was also observed when using peracetylated d-Lac **16** with *p*-chlorothiophenol as the leaving group. The selective sixth position deacetylation product **17** was the major outcome of the reaction with the β-anomer, while most of the α-anomer of d-Lac **16** did not react. The reaction of d-Lac **16** with CAL-B reacted similar to peracetylated β-d-Glc-SPh rather than d-Gal thioglycosides. This suggests that the saccharide equipped with a leaving group has a larger influence on CAL-B catalytic activity than the nonreducing terminal saccharide.^20^

**Scheme 6:**
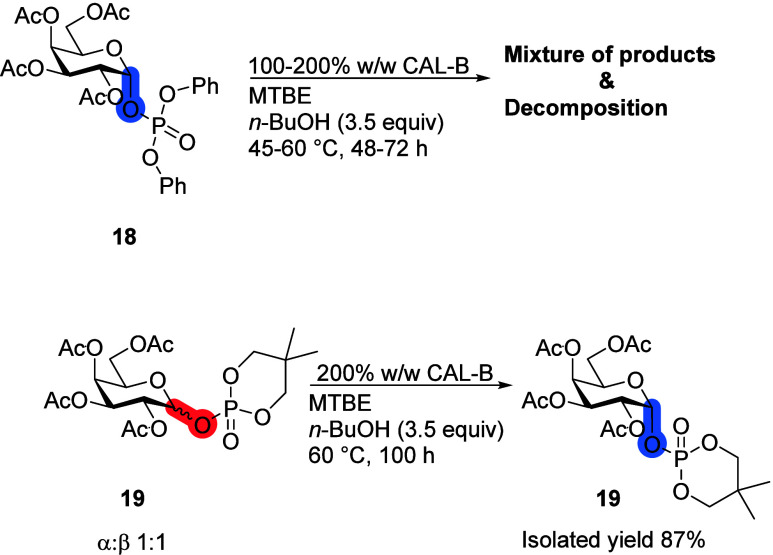
Influence of the Phosphorous Leaving Group on the CAL-B Deacetylation Reaction Reaction conditions: monosaccharide (100 mg), 200% CAL-B (w/w), *n*-BuOH (3.5 equiv), MTBE (10 mL), 60 °C.

Next, Gal with phosphate leaving groups, which are the native substrates in enzymatic glycosylations,^23^ were under the study (Scheme 6). Acyclic phosphate ester **18** and its products were too unstable to give reasonable and practically applicable results (Scheme S2, Table S1). Therefore, the more stable cyclic aliphatic phosphate ester **19** was tested for the deacetylation with CAL-B instead. Surprisingly, with 200% CAL-B and at 60 °C almost no products of the transesterification reaction were detected. Instead, anomeric enrichment had happened. The ratio of α/β anomers in compound **19** was initially approximately 1:1; after 4 days, it had fully converted to the α-anomer. Phosphate and phosphonate esters are known to inhibit lipases,^24−26^ and the latter compounds have been crystallized with CAL-B, showing their interactions with the catalytic triad.^27^ As for monosaccharide **19**’s phosphate leaving group, it does not inhibit CAL-B completely nor does it form an irreversible covalent bond with the catalytic serine-105. Instead, it seems to interact in the catalytic site and inhibit the transesterification reaction. We speculate that the β-anomer of **19** is preferred over the α-anomer, and as it interacts with the catalytic triad, it temporarily breaks the bond between the Gal first position’s oxygen and the phosphate leaving group’s phosphorus atom. The formed anion then epimerises into the α-configuration, which is thermodynamically favored due to the anomeric effect. Finally, the bond is formed between Gal and the phosphate leaving group, thus forming the α-anomer of **19** as the sole product. An alternative nonenzymatic approach consisting of dissociation/association mechanism cannot also be excluded.

To conclude, acetate is a specific protecting group in the anomeric position, enabling regioselective deacetylation with CAL-B depending on the relative configuration of the substituent. Sterically more demanding groups, such as thiophenol derivatives, direct the attack to other positions or block it completely (as with phosphates). A smaller chloride substituent forms products that inhibit the activity of CAL-B and initiate acyl migration. Either due to different positions being deacetylated or the due to the characteristics of the substituents (steric influence, electronic effects, etc.), the reactions were significantly slower, especially with nonacetate β-anomeric substituents, demanding higher temperatures and more enzyme to be used.

### Second Position

**Scheme 7:**
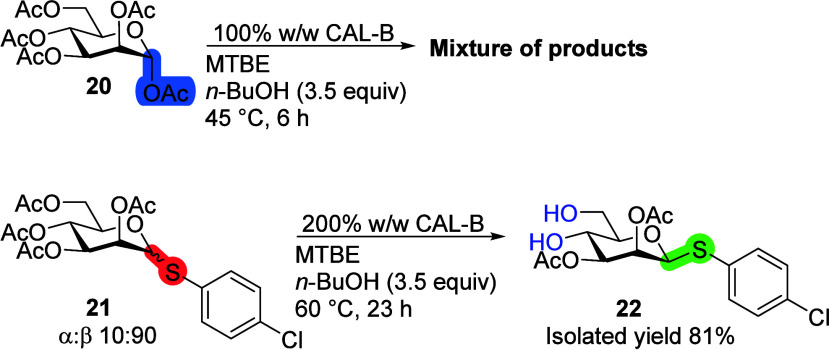
Influence of the Second Position on the CAL-B Transesterification Reactions Reaction conditions: monosaccharide (100 mg), 100 or 200% CAL-B (w/w), *n*-BuOH (3.5 equiv), MTBE (10 mL), 45 or 60 °C.

To check the influence of the substituents in the second position on the deacetylation reaction with CAL-B, α-d-mannose pentaacetate **20** was used (Scheme 7). The reaction reached full conversion in 6 h. Unfortunately, TLC showed many products, and crude NMR analysis revealed at least 11 anomeric protons. These analysis results show that α-mannose pentaacetate **20** cannot be selectively deprotected using CAL-B. d-Mannose with the *p*-chlorothiophenol leaving group (compound **21**) did not react at all in 48 h with 45 °C and 100% CAL-B. Increasing the amount of enzyme and using elevated temperatures led to the selective deacetylation of the β-anomer in 12 h in the sixth and the fourth positions affording product **22**, while the α-anomer did not react in 23 h. Compared to peracetylated β-d-Glc-SPh,^20^ CAL-B has lower catalytic activity with d-mannose **21** but the same selectivity.

### Fourth Position

Comparing the two epimers, d-Gal and d-Glc, it is clear, that their structural differences influence the catalytic activity of the enzyme and therefore the rate of the reaction with CAL-B. If the substituents in the fourth and in the fifth positions are *trans* to each other (compound **1**, Scheme 2), the reaction is roughly 6–8 times faster compared to when they are cis (compound **3**, Scheme 3). Corresponding α-anomers, compounds **5** (Scheme 3) and **42** (Scheme 12), lead to different products. The sixth position deacetylation is the major product **6** for d-Gal, but for d-Glc, deacetylation of the sixth position is an intermediate through which the main product **43** is formed. Thus, the fourth position’s configuration affects whether it is deacetylated during the transesterification reaction. Similar results for α-anomers can be seen with chloride substituents (compounds **7** and **9**, Scheme 4), even though due to the substituent effects, the fourth position can be deacetylated through acyl migration. If the fourth position is deacetylated during the reaction, further deacetylation for both epimers does not happen contrary to the reactions in buffer solution.^20,28^

### d- and l-Configurations

**Scheme 8:**
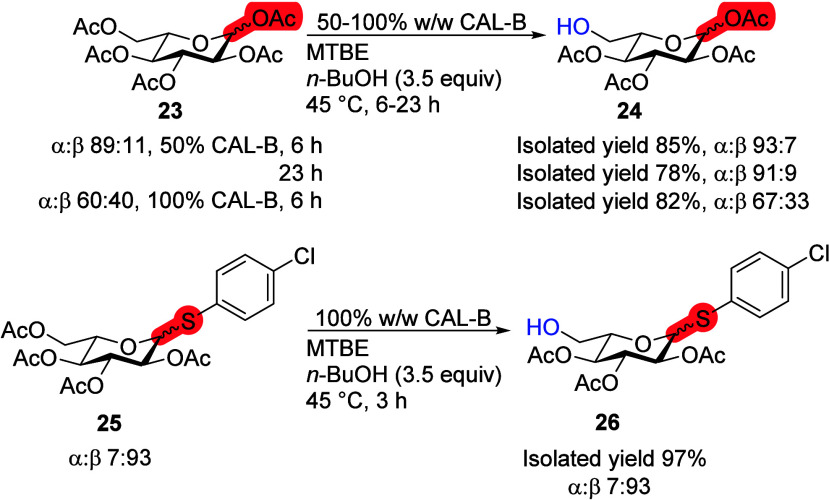
Influence of l-Configuration of Glc on the Transesterification Reaction with CAL-B Reaction conditions: monosaccharide (100 mg), 50 or 100% CAL-B (w/w), *n*-BuOH (3.5 equiv), MTBE (10 mL), 45 °C.

To further investigate the influence of configurational differences, CAL-B transesterification reactions were conducted on l-Glc pentaacetate **23** and *p*-chlorothiophenol equipped l-Glc tetraacetate **25** (Scheme 8). Anomeric enrichment^29,30^ was carried out on l-Glc pentaacetate **23** from an α:β ratio of 60:40 to 89:11 (Scheme S3). The reaction of the obtained anomeric mixture with CAL-B deacetylated the sixth position of both anomers in 6 h (product **24**). No anomeric preference was detected. Full conversion was not reached even after 23 h, which indicates that product **24** inhibits CAL-B catalytic activity. For l-thioglycoside **25**, CAL-B showed a very high selectivity toward deacetylating the sixth position and yielding product **26** in almost quantitative yield (97%) with fully retained anomeric configuration (Scheme 8). For Glc, CAL-B does not prefer one enantiomer over the other, but can deacetylate the d-enantiomer more than once,^20^ while showing no anomeric preference for the l-enantiomer.

**Scheme 9:**
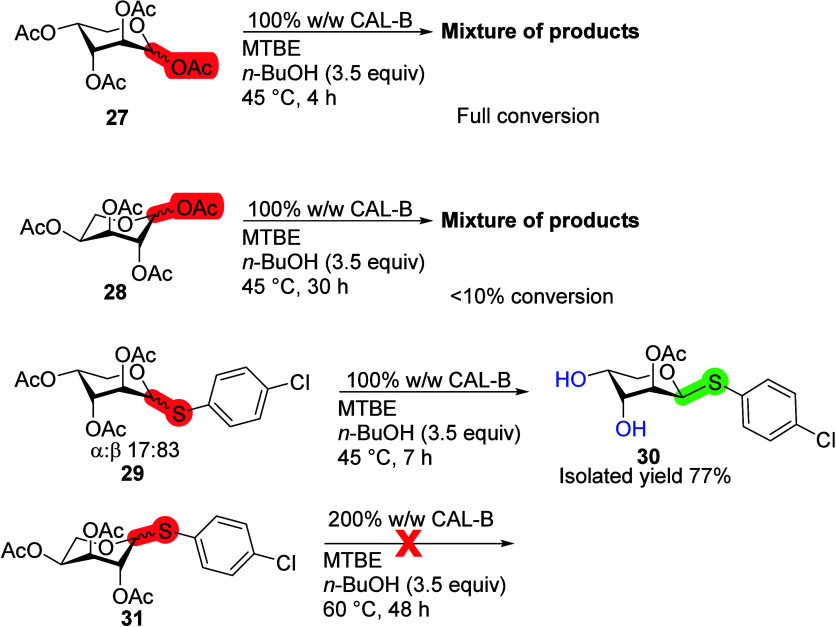
Influence of d- and l-Configurations of Arabinopyrnoses on the CAL-B Deacetylation Reaction Reaction conditions: monosaccharide (100 mg), 100% or 200% CAL-B (w/w), *n*-BuOH (3.5 equiv), MTBE (10 mL), 45 or 60 °C.

Our study of the configurational differences was continued on the d- and l-enantiomers of arabinopyranoses (Ara) with CAL-B transesterification reactions. Both peracetylated Ara enantiomers (**27** and **28**, Scheme 9) lacked selectivity, giving multitude of products. Nevertheless, d-Ara tetraacetate **27** reacted much faster than l-Ara tetraacetate **28**, having reached full conversion in 4 h, while l-Ara **28** had <10% conversion in 30 h. When thioglycosides **29** and **31** were used in transesterification reactions with CAL-B, both enantiomeric and anomeric preferences were observed. The reaction of β-d-Ara **29** reached full conversion in 7 h, affording a monoacetate **30** (77% overall yield, 92% comparing β-anomers only), while α-d-Ara **29** remained unreacted after 24 h according to ^1^H NMR. l-Ara with *p*-chlorothiophenol leaving group **31** did not react even at elevated temperatures and increased amounts of enzyme. These results indicate a clear preference of the d-enantiomer of Ara over the l-enantiomer for CAL-B.

**Scheme 10:**
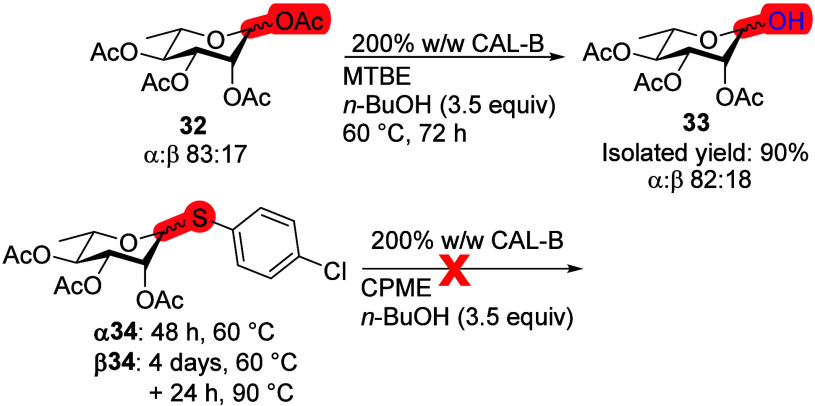
Investigation of l-Rha Transesterification Reaction with CAL-B Reaction conditions: monosaccharide (100 mg), 200% CAL-B (w/w), *n*-BuOH (3.5 equiv), MTBE or CPME (10 mL), 60 °C or 60 → 90 °C.

For l-rhamnose (Rha), peracetylated Rha **32** reacted slowly at 45 °C. Increasing the temperature and the amount of enzyme led to the first position deprotected product **33** after 3 days in 90% isolated yield (Scheme 10). There was no anomeric selectivity observed for Rha. Comparing l-Rha **32** (which is 6-deoxy-l-mannopyranose) to the d-mannose pentaacetate **20** reaction with CAL-B (Scheme 7) showed an increase in selectivity. Unfortunately, no further comparison can be made about selectivity, as both anomers of Rha equipped with *p*-chlorothiophenol leaving group **34** did not react even after increasing the temperature to 90 °C.

### Different Protecting Groups

**Scheme 11:**
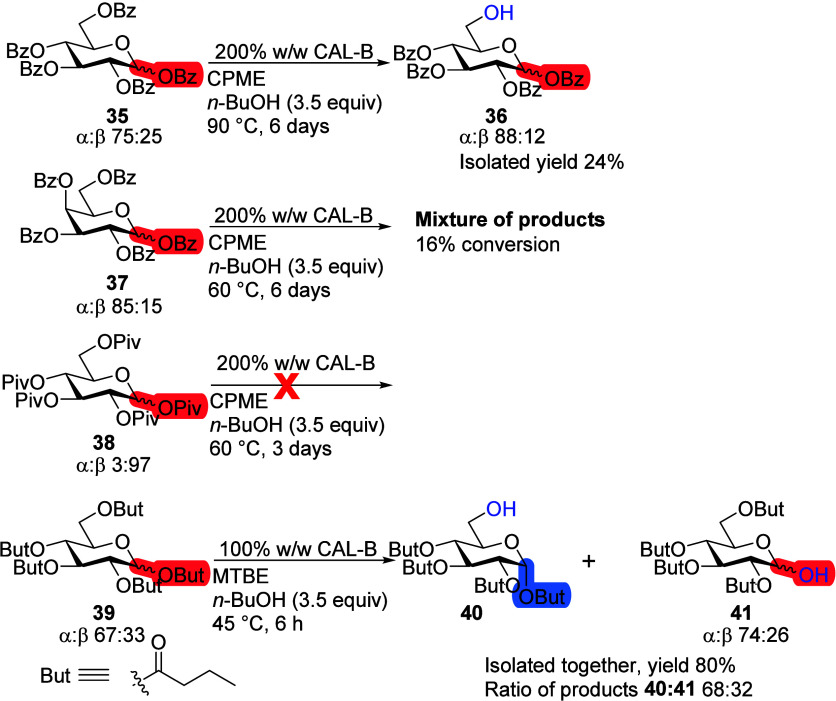
CAL-B Deacetylation Reaction with Monosaccharides Protected with Various Protecting Groups Reaction conditions: monosaccharide (100 mg), *n*-BuOH (3.5 equiv), 100–200% CAL-B, MTBE or CPME (10 mL), 45–90 °C.

Deprotection of benzoylated d-Glc **35** with CAL-B was selective toward the sixth position affording compound **36** but was very slow even after increasing the temperature to 90 °C (Scheme 11). The initial α/β anomeric ratio of d-Glc **35** was 75:25, which did not change throughout the reaction according to crude ^1^H NMR data. Benzoylated d-Gal **37** had the same very slow rate of reaction as compound **35**. After 6 days with 200% CAL-B and 60 °C, 16% conversion was reached according to NMR, but there was no clear selectivity as at least five different products were detected. Continuing with bulky protecting groups, pivaloylated d-Glc **38** gave no reaction even after 3 days at 60 °C with 200% CAL-B. Butyryl ester-protected d-Glc **39** on the other hand showed full conversion after 6 h. Two products, sixth position deprotected **40** and deprotected anomers **41**, were isolated in one fraction with a total yield of 80%. The ratio of the formed products **40**:**41** was 2:1, which coincided with the initial anomeric ratio of the starting material **39**. Since the product **40** was purely an α-anomer, it can be concluded that the sixth position deacetylation occurs with the α-anomer of **39**, while the β-anomer is deacetylated in the first position.

To summarize, CAL-B shows some catalytic activity with benzoylated monosaccharides, but they react rather slowly or give mixtures of products (for d-Gal) to be practically viable. Pivaloylated d-Glc **38** seems to be the limit for CAL-B as there was no reaction at all. Butyrated d-Glc **39** shows similar anomeric preferences as seen with acetyl protecting groups, although only a monodeacetylated product is formed. As CAL-B’s natural substrates are fatty acid esters, it seems to prefer linear aliphatic ester protecting groups.

### Nucleophilic Transesterification Reagents

**Scheme 12:**
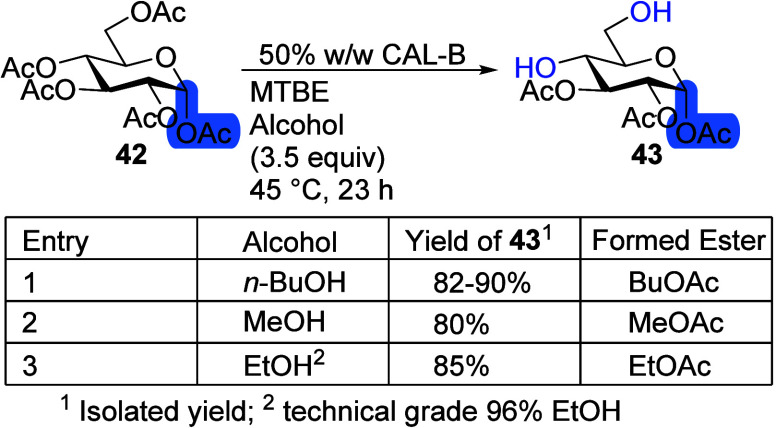
Different Alcohols for Transesterification with α-d-Glc Pentaacetate **42** Reaction conditions: α-d-Glc pentaacetate **42** (100 mg), 50% CAL-B (w/w), alcohol (3.5 equiv), MTBE (10 mL), 45 °C, 23 h.

During the deacetylation reaction with CAL-B when *n*-BuOH is used as a nucleophilic reagent, *n*-BuOAc as a side product is formed. To replace it with lower boiling esters, ethanol and methanol were tested as nucleophilic transesterification reagents (Scheme 12). Using MeOH or EtOH would simplify the workup and working with the crude reaction mixtures.

Previously, when 50% CAL-B (w/w) was used in the deacetylation reaction with α-d-Glc pentaacetate **42**, after 23 h, 82% yield was achieved.^20^ Using MeOH and EtOH as the nucleophilic reagents did not influence the reaction outcome significantly (Scheme 12, entries 1–3). When EtOH was used with peracetylated β-d-Glc **1**, in 30 min, 95% NMR yield was achieved with the products' anomeric ratio α:β 54:46, compared to the *n*-BuOH outcome of 97% NMR yield with α:β 33:67 (Scheme 2). Crude NMR was clear of any side products of the transesterification reaction (see the SI). The only notable difference when using EtOH instead of *n*-BuOH as the nucleophilic reagent was the anomeric ratio of the products. As such, both alcohols seem to be good alternatives to *n*-BuOH.

### Enzyme Recycling

**Figure 2:**
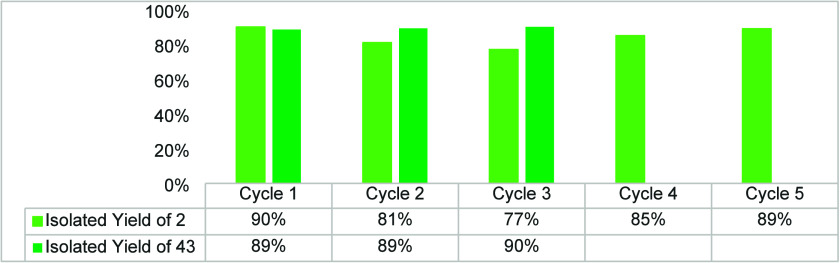
Recycling of immobilized CAL-B. Light green bars show the results of recycling CAL-B in transesterification reactions with β-d-Glc pentaacetate **1**; reaction conditions: β-d-Glc pentaacetate **1** (100 mg), 100% CAL-B (w/w), *n*-BuOH (3.5 equiv), MTBE (10 mL), 45 °C, 30 min. Dark green bars show the results of recycling CAL-B in transesterification reactions with α-d-Glc pentaacetate **42**; reaction conditions: α-d-Glc pentaacetate **42** (100 mg), 50% CAL-B (w/w), *n*-BuOH (3.5 equiv), MTBE (10 mL), 45 °C, 23 h.

Novozyme N435 has been successfully recycled from organic media reactions and reused by Rodrigues et al.^31^ We chose two reactions for testing of the recyclability of immobilized CAL-B: a short reaction time (30 min) with β-d-Glc pentaacetate **1** (Scheme 2) with 100% CAL-B and a long reaction time (23 h) with α-d-Glc pentaacetate **42** with 50% CAL-B (Scheme 12). Five cycles were carried out with short reaction time to mainly test how the washing and filtering would influence CAL-B, while three cycles were done with long reaction time to mainly test the influence of mechanical stirring on the stability of the immobilized enzyme (Figure 2, Table S2). After each reaction, immobilized CAL-B was washed with DCM (∼50 mL) and air-dried for 1 h. The isolated yields of deacetylated tetraacetate **2** varied in the range of experimental errors. Recycling CAL-B in transesterification reactions with α-d-Glc pentaacetate **42** had very stable results, showing that mechanical stirring does not influence the catalytic activity of CAL-B. Based on these results, we conclude that CAL-B can be recycled successfully in both long and short reaction time reactions.

## Conclusions

In conclusion, an experimentally simple protocol for the deacetylation of various pyranose monosaccharide derivatives with immobilized CAL-B was elaborated, providing valuable synthetic intermediates for the carbohydrate synthesis. Depending on the configuration and the nature of the protecting groups, several trends and correlations were identified within CAL-B transesterification reactions.

The anomeric position directs the transesterification reaction outcomes to different positions, affording selectively deprotected products with varying reaction rates. The second position’s configuration, for α-pentaacetates, determines whether the transesterification reaction is selective or not. The fourth position’s configuration influences the rate of the reaction and whether the main product is a diol or monodeprotected saccharide. CAL-B can differentiate between d- and l-enantiomers of pyranose monosaccharides. We also found that saccharides with a Cl-leaving group do not reach full conversion as they form products that inhibit the transesterification reaction. Meanwhile, the thiophenol leaving group and its derivates give similar results but overall decrease the rate of the reaction. Phosphorus leaving groups seem to interact with CAL-B directly, inhibiting the transesterification reaction, and instead anomeric enrichment happens. Bulkier protecting groups either give a slow reaction (benzoyl), do not react (pivaloyl), or partially follow the same behavior of acetylated counterparts (butyrated). We showed that *n*-BuOH can be replaced by lower boiling point nucleophilic reagents such as MeOH and EtOH. Finally, CAL-B was successfully recycled and reused in two different reactions up to five cycles.

## Experimental Section

### General Experimental Information

Full assignment of ^1^H and ^13^C chemical shifts was based on the 1D and 2D (COSY, HSQC and HMBC) FT NMR spectra measured with a Bruker Avance III 400 MHz instrument. Residual solvent signals were used (CDCl_3_: δ = 7.26 ^1^H NMR, 77.2 ^13^C NMR; CD_3_OD: δ = 3.31 ^1^H NMR, 49.0 ^13^C NMR; (CD_3_)_2_SO: δ = 2.50 ^1^H NMR, 39.5 ^13^C NMR; D_2_O: δ = 4.79 ^1^H NMR) as internal standards. High-resolution mass spectra were recorded with an Agilent Technologies 6540 UHD Accurate-Mass QTOF LC/MS spectrometer by using AJ-ESI ionization. Prior to analysis, the instrument was calibrated in a mass range of *m*/*z* 50–3200. Optical rotations were obtained with an Anton Paar GWB Polarimeter MCP 500. Melting points were determined using a polarizing optical microscope Nagema-K8. Precoated Merck silica gel 60 F_254_ plates were used for TLC, and column chromatography was performed with Merck 60 (0.040–0.063 mm) mesh silica gel. Commercial reagents and solvents were generally used as received. DCM was distilled over CaH or phosphorus pentoxide, ethyl acetate (EtOAc) and acetone over phosphorus pentoxide, and MeOH and toluene over sodium. Petroleum ether (PE) had a boiling point of 40–60 °C. Immobilized *C. antarctica* lipase-B, on hydrophobic acrylic resin, Novozyme N435, with 10,000 (propyl laurate unit/g) activity was a kind gift from Novozymes A/S.

### General Procedure for Preparation of 1,2,3-Tri-*O*-acetyl-α-d-glucopyranoside **43** with CAL-B

In a manner analogous to Kanger et al.,^20^ α-d-glucose pentaacetate **42** (100 mg), MTBE (10 mL), and *n*-BuOH (3.5 equiv) were mixed at 45 °C. After saccharide was dissolved, stirring was set to 100 rpm, and 50% CAL-B (50 mg) was added. The reaction was followed by TLC, and upon completion after 23 h, the reaction mixture was filtered, immobilized enzymes were rinsed with DCM (∼50 mL), and the filtrate was concentrate in vacuo. The crude mixture was purified by silica gel column chromatography (PE:EtOAc 2:1 →1:4), yielding a white solid (70 mg, 90%). TLC – PE:EtOAc 1:2, *R*_*f*_ = 0.19; NMR matches with previously reported values.^20^

### Gram-Scale Synthesis of 1,2,3-Tri-*O*-acetyl-α-d-glucopyranoside **43**

According to the general procedure with α-d-Glc pentaacetate **42** (2.56 mmol, 1 g), MTBE (100 mL), *n*-BuOH (3.5 equiv, 821 μL), 45 °C, and 50% CAL-B (500 mg), the reaction was run for 23 h and resulted in a white solid (705 mg, 90%).

## Data Availability

The data underlying this study are available in the published article and its Supporting Information.

## Supporting Information Available

Experimental procedures, characterization data, and NMR spectra for all synthesized compounds (PDF)
