# Supplementary material for: Interplay of Monosaccharide Configurations on the Deacetylation with Candida antarctica Lipase-B
Source: J Org Chem. 2024 Dec 30;90(1):663–71. doi: 10.1021/acs.joc.4c02582 (PMC11731304; doi:10.1021/acs.joc.4c02582)
Supplement: Supplementary file 1 — jo4c02582_si_001.pdf [file jo4c02582_si_001.pdf]

Supporting Information

Interplay of monosaccharide configurations on the deacetylation with CAL-B

Kaarel Erik Hunt, Annette Miller, Kristin Liias, Tatsiana Jarg, Kadri Kriis and Tõnis Kanger*

Department of Chemistry and Biotechnology, Tallinn University of Technology, Akadeemia tee 15, 12618 Tallinn, Estonia.

Corresponding Author: Tõnis Kanger tonis.kanger@taltech.ee

Contents

Figure S1: CAL-B transesterification reactions with β-D-Glc pentaacetate 1 .... S4

Scheme S1: CAL-B transesterification reactions with α-D-Gal pentaacetate 5 and α-D-Gal-Cl tetraacetate 9 .... S5

Table S1 and Scheme S2: CAL-B transesterification reaction with α-D-Gal phosphate ester tetraacetate 18 .... S6

Scheme S3: Anomeric enrichment with L-Glc pentaacetate 23 .... S7

Table S2: CAL-B recycling reactions with β-D-Glc pentaacetate 1 and α-D-Glc pentaacetate 42 .... S8

General Experimental Information S9

Starting Material Synthesis S9

General Procedures S10

General procedure for transesterification reactions with CAL-B S10

Experimental procedures and characterisation of products. S10

2,3,4,6-tetra-*O*-acetyl-D-glucopyranose (**2**) S10

2,3,4,6-tetra-*O*-acetyl-D-galactopyranose (**4**) S10

1,2,3,4-tetra-*O*-acetyl-α-D-galactopyranoside (**6**) S11

2,3-di-*O*-acetyl-α-D-glucopyranosyl chloride (**8**) S11

2,3,4-tri-*O*-acetyl-α-D-galactopyranosyl chloride (**10**) and **2,3,6-tri-*O*-acetyl-α-D-galactopyranosyl chloride** (**11**) S11

4-chlorophenyl 2,3,4-tri-*O*-acetyl-1-thio-β-D-galactopyranoside (**13**) S11

2,6-dimethylphenyl 2,3,4-tri-*O*-acetyl-1-thio-D-galactopyranoside (**15**) S12

4-chlorophenyl (2,3,4,6-tetra-*O*-acetyl-β-D-galactopyranosyl)-(1→4)-2,3,6-tri-*O*-acetyl-1-thio-D-glucopyranoside (**16**) S12

4-chlorophenyl (2,3,4,6-tetra-*O*-acetyl-β-D-galactopyranosyl)-(1→4)-2,3-di-*O*-acetyl-1-thio-β-D-glucopyranoside (**17**) S13

5,5-dimethyl 2,3,4,6-tetra-*O*-acetyl-D-galactopyranosyl 1,3,2-dioxaphosphinane-2-oxide (**19**) S13

4-chlorophenyl 2,3-di-*O*-acetyl-1-thio-β-D-mannopyranoside (**22**) S14

1,2,3,4-tetra-*O*-acetyl-L-glucopyranoside (24) S14

4-chlorophenyl 2,3,4,6-tetra-*O*-acetyl-1-thio-L-glucopyranoside (25) S14

4-chlorophenyl 2,3,4-tri-*O*-acetyl-1-thio-L-glucopyranoside (26) S15

4-chlorophenyl 2,3,4-tri-*O*-acetyl-1-thio-D-arabinopyranoside (**29**) S15

4-chlorophenyl 2-O-acetyl-1-thio-β-D-arabinopyranoside (**30**) S15

4-chlorophenyl 2,3,4-tri-*O*-acetyl-1-thio-L-arabinopyranoside (**31**) S16

2,3,4-tri-*O*-acetyl-6-deoxy-L-mannopyranose (**33**) S16

1,2,3,4-tetra-*O*-benzoyl-D-glucopyranoside (**36**) S16

1,2,3,4-tetra-*O*-butyryl-α-D-glucopyranoside (40) and 2,3,4,6-tetra-*O*-butyryl-D-glucopyranose (**41**) S16

1,2,3-tri-*O*-acetyl-α-D-glucopyranoside (**43**) S17

Diphenyl 2,4,6-tri-*O*-α-D-galactopyranosyl phosphate (S1), diphenyl 2,3,6-tri-*O*-α-D-galactopyranosyl phosphate (S2) and **diphenyl 2,3,4-tri-*O*-α-D-galactopyranosyl phosphate** (**S3**) S17

NMR Spectra S19

2,3,4,6-tetra-*O*-acetyl-D-glucopyranose (**2**) α:β 7:3 .... S19

2, 3,4,6-tetra-*O*-acetyl-D-galactopyranose (**4**) α:β 7:3 .... S24

1,2,3,4-tetra-*O*-acetyl-α-D-galactopyranoside (**6**) S28

2,3,4,6-tetra-*O*-acetyl-α-D-glucopyranosyl chloride (**7**) S32

2,3-di-*O*-acetyl-α-D-glucopyranosyl chloride (**8**) S33

2,3,4,6-tetra-*O*-acetyl-α-D-galactopyranosyl chloride (**9**) S38

Mixture of 2,3,4-tri-*O*-acetyl-α-D-galactopyranosyl chloride (**10**) and **2,3,6-tri-*O*-acetyl-α-D-galactopyranosyl chloride** (**11**) ~1:1, **10** shown S39

Mixture of 2,3,4-tri-*O*-acetyl-α-D-galactopyranosyl chloride (**10**) and **2,3,6-tri-*O*-acetyl-α-D-galactopyranosyl chloride** (**11**) ~1:9, **11** shown S41

4-chlorophenyl 2,3,4,6-tetra-*O*-acetyl-1-thio-β-D-galactopyranoside (**12**) S46

4-chlorophenyl 2,3,4-tri-*O*-acetyl-1-thio-β-D-galactopyranoside (**13**) S47

2,6-dimethylphenyl 2,3,4,6-tetra-*O*-acetyl-1-thio-D-galactopyranoside (**14**) α:β 75:25 .... S52

2,6-dimethylphenyl 2,3,4-tri-*O*-acetyl-1-thio-D-galactopyranoside (**15**) α:β 78:22 .... S53

4-chlorophenyl (2,3,4,6-tetra-*O*-acetyl-β-D-galactopyranosyl)-(1→4)-2,3,6-tri-*O*-acetyl-1-thio-D-glucopyranoside (**16**) α:β 21:79 .... S58

4-chlorophenyl (2,3,4,6-tetra-*O*-acetyl-β-D-galactopyranosyl)-(1→4)-2,3-di-*O*-acetyl-1-thio-β-D-glucopyranoside (**17**) S63

diphenyl 2,3,4,6-tetra-*O*-α-D-galactopyranosyl phosphate (**18**) S68

5,5-dimethyl 2,3,4,6-tetra-*O*-D-galactopyranosyl 1,3,2-dioxaphosphinane (**19**) α:β 1:2 .... S69

4-chlorophenyl 2,3,4,6-tetra-*O*-acetyl-1-thio-D-mannopyranoside (**21**) α:β 1:9 .... S74

4-chlorophenyl 2,3-di-*O*-acetyl-1-thio-β-D-mannopyranoside (**22**) S75

1,2,3,4,6-penta-*O*-acetyl-L-glucopyranoside (**23**) α:β 3:2 .... S80

1,2,3,4,6-penta-*O*-acetyl-L-glucopyranoside (**23**) α:β 9:1 .... S84

1,2,3,4-tetra-*O*-acetyl-L-glucopyranoside (**24**) α:β 2:1 .... S85

4-chlorophenyl 2,3,4,6-tetra-*O*-acetyl-1-thio-L-glucopyranoside (**25**) α:β 7:93 .... S90

4-chlorophenyl 2,3,4-tri-*O*-acetyl-1-thio-L-glucopyranoside (**26**) α:β 7:93 .... S95

1,2,3,4-tetra-*O*-acetyl-D-arabinopyranoside (**27**) α:(β+furanose) 94:6 .... S100

1,2,3,4-tetra-*O*-acetyl-L-arabinopyranoside (**28**) α:(β+furanose) 91:9 .... S101

4-chlorophenyl 2,3,4-tri-*O*-acetyl-1-thio-D-arabinopyranoside (**29**) α:β 17:83 .... S102

4-chlorophenyl 2-*O*-acetyl-1-thio-β-D-arabinopyranoside (30) S107

4-chlorophenyl 2,3,4-tri-*O*-acetyl-1-thio-L-arabinopyranoside (**31**) α:β 17:83 .... S112

1,2,3,4-tetra-*O*-acetyl-6-deoxy-L-mannopyranoside (**32**) α:β 83:17 .... S117

2,3,4-tri-*O*-acetyl-6-deoxy-L-mannopyranose (**33**) α:β 91:9 .... S118

4-chlorophenyl 2,3,4-tri-*O*-acetyl-6-deoxy-1-thio-L-mannopyranoside (**34**) α:β 69:31 .... S120

1,2,3,4,6-penta-*O*-benzoyl-D-glucopyranoside (**35**) α:β 73:27 .... S121

1,2,3,4-tetra-*O*-benzoyl-D-glucopyranoside (**36**) α:β 88:12 .... S122

1,2,3,4,6-penta-*O*-benzoyl-D-galactopyranoside (**37**) α:β 73:27 .... S126

1,2,3,4,6-penta-*O*-pivaloyl-D-glucopyranoside (**38**) α:β 4:96 .... S127

1,2,3,4,6-penta-*O*-butyryl-D-glucopyranoside (**39**) α:β 2:1 .... S128

1,2,3,4-tetra-*O*-butyryl-α-D-glucopyranoside (**40**) and 2,3,4,6-tetra-*O*-butyryl-D-glucopyranoside (**41**) S129

1,2,3-tri-*O*-acetyl-α-D-glucopyranoside (**43**) S135

Diphenyl 2,4,6-tri-*O*-α-D-galactopyranosyl phosphate (S1), diphenyl 2,3,6-tri-*O*-α-D-galactopyranosyl phosphate (S2) and **diphenyl 2,3,4-tri-*O*-α-D-galactopyranosyl phosphate** (**S3**); **S1**:**S2**:**S3** 10:10:1 .... S137

References S142

Figure S1: CAL-B transesterification reactions with β-D-Glc pentaacetate 1

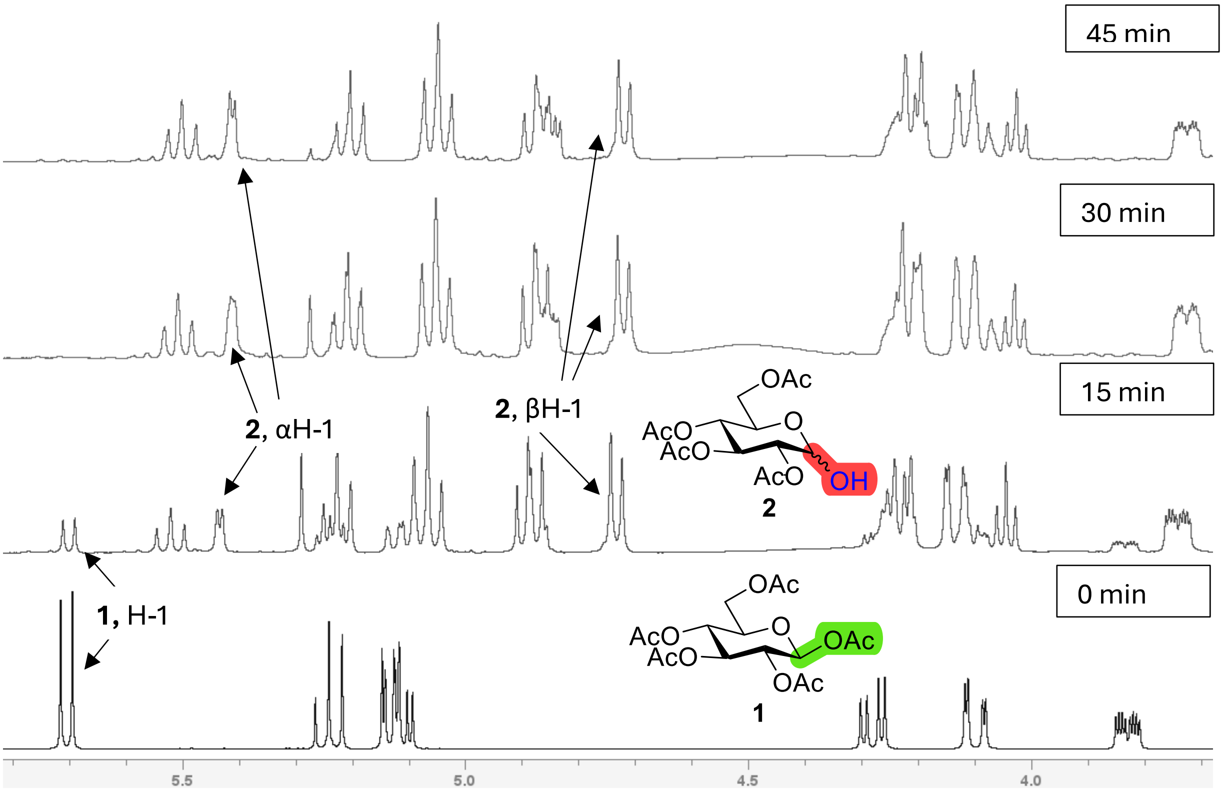

*Figure S1 Zoomed in snipped portions of ^{1}H NMR spectra from β-D-Glc **1** pentaacetate reacting with CAL-B (Scheme 2). Reaction conditions: **1** (100 mg), 100% CAL-B (w/w), n-BuOH (3.5 equiv), MTBE (10 mL), 45 °C.*

Figure S1 shows how peracetylated β-D-Glc **1** pentaacetate reacts in 0, 15, 30 and 45 min (Scheme 2). It seems that small amounts of side-products form in the first 15 min of the reaction and afterwards selective anomeric deprotection occurs leading to roughly 97% NMR yield. After 30 minutes there seems to be no clear further deprotection happening as 45 min spectra is almost replica of the 30 min one.

Scheme S1: CAL-B transesterification reactions with α-D-Gal pentaacetate 5 and α-D-Gal-Cl tetraacetate 9

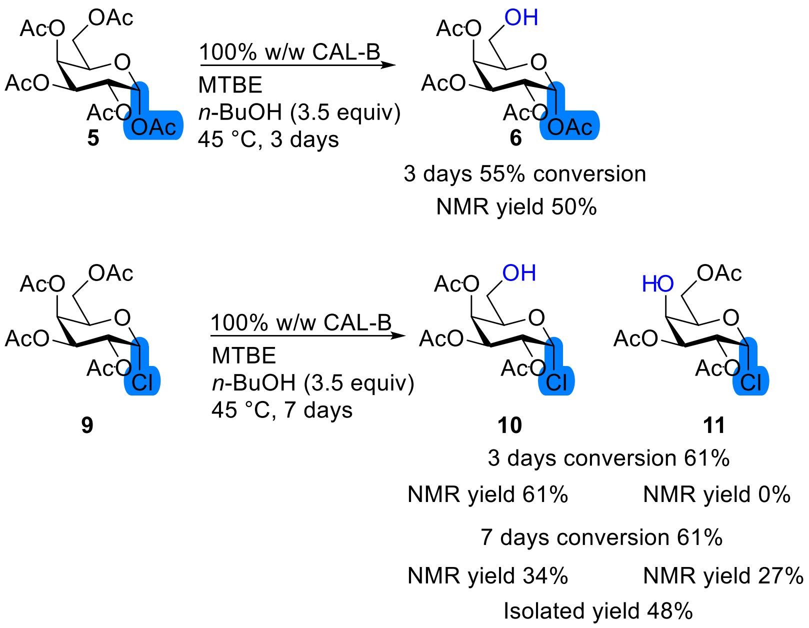

*Scheme S1 CAL-B deacetylation reactions with α-Gal compounds **5** and **9**. Reaction conditions: monosaccharide (100 mg), 100% CAL-B (w/w), n-BuOH (3.5 equiv), MTBE (10 mL), 45 °C.*

Reaction with peracetylated α-Gal **5** was sluggish and was stopped after 3 days. Despite being slow, the reaction had high selectivity with sixth position deacetylation product **6** dominating. While slightly faster Gal-Cl **9** had 61% conversion after 3 days but did not go any further as even after 7 days the conversion remained the same, indicating a shift from transesterification to acyl migration after 3 days. At 3 days the main product was sixth position deacetylated **10**, after 7 days fourth position deacetylation product **11** appeared as a secondary product. After purification it had become the major product with roughly ~2:1 ratio over **10**. The crude NMR showed 34% **10** and 27% **11**, which indicates that during column chromatography acyl migration had continued.

Table S1 and Scheme S2: CAL-B transesterification reaction with α-D-Gal phosphate ester tetraacetate 18

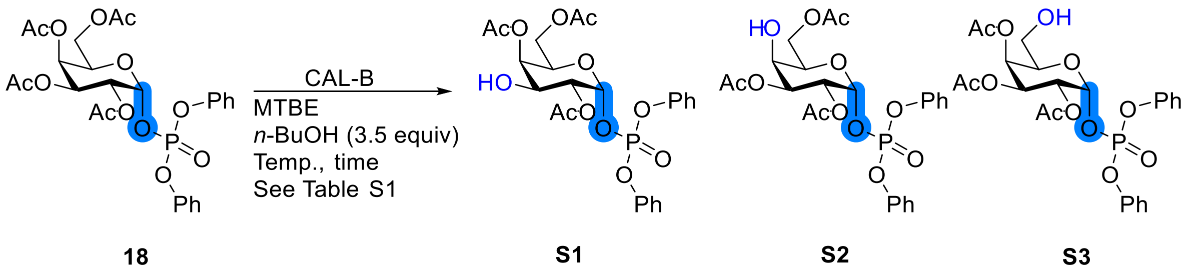

*Scheme S2 CAL-B deacetylation reactions with Gal equipped with aromatic phosphate esters **18**.*

Table S1 Deacetylation reaction with phosphate ester **18**. Prod. – product, conv. – conversion of crude by ^1H NMR. Reaction conditions: 130 mg phosphate ester **18**, 200% CAL-B (w/w), 3.5 equiv *n*-BuOH, 0.03 M MTBE, reflux (60 °C), 48 h; [1] 100 mg phosphate ester **18**, 100% CAL-B, 45 °C; [2] crude deteriorated in fridge after concentration; [3] 60 mg phosphate ester **18**, 72 h; [4] column chromatography run with 1% Et_3N in petroleum ether

No	CAL-B (w/w) (%)	Temp. (°C)	Conv. (%)	Prod. ratio crude			Isolated yield (%)	Prod. ratio purified		
				**S1**	**S2**	**S3**		**S1**	**S2**	**S3**
1[1]	100	45	40	0:0:1			-	-		
2	200	60	100	1:8:1[2]			-	-		
3	200	60	-	-			70	0:3:7		
4[3]	200	60	85	1:7:2			80	10:10:1[4]		

Using α-Gal equipped with aromatic phosphate esters **18** with 100% CAL-B at 45 °C resulted in a slow reaction, after 2 days the reaction seemed to have completely stopped and led to 40% conversion (Scheme S2, Table S1 no 1). Only sixth position deacetylation product **S3** was detected. When the temperature and the amount of CAL-B was increased, the reaction reached completion in 2 days and seemed to favour fourth position deacetylation, product **S2**, which is similar to acyl migration with Gal-Cl **9**, but without completely inhibiting the reaction (Table 1S no 2). Unfortunately, diaryl phosphorous leaving group is unstable and the first crude decomposed in fridge. The next run (Table S1, no 3), upon completion, was purified right away resulting in 70% isolated yield, with sixth position deacetylated **S3** being the dominant product instead of **S2** (Table S1, no 2, 4). If basic additive (1% Et_3N) was used during purification by silica gel column chromatography **S2** and now the third position deacetylation product **S1** dominated (Table S1, no 4). Products **S1-S3** were always isolated together in 1 fraction. Different basic and acid reagents were tried to control the acyl migration, which all led to decomposition. The same happened with prolonged concentration in vacuo. The authors discourage the use of this diaryl phosphorous leaving group as it is prone to decompose.

Scheme S3: Anomeric enrichment with L-Glc pentaacetate 23

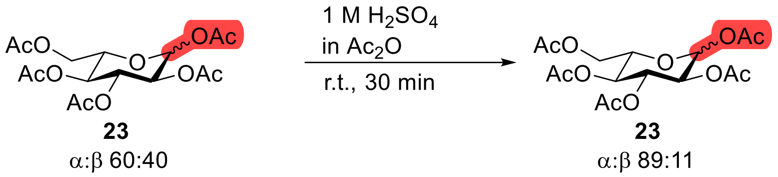

*Scheme S3 Anomeric enrichment with L-glucose pentaacetate **23***

Modified procedure from literature[1,2]: L-glucose pentaacetate (300 mg) with α:β 60:40 was dissolved in 1 M H_2SO_4 in Ac_2O (3 mL, from 6.25 mL stock (0.335 mL conc. H_2SO_4 and 5.915 mL Ac_2O)) in 50 mL round bottom flask and stirred at room temperature for 30 minutes. The reaction mixture was cooled down to 0 °C, cold water (~10 mL) and DCM (~20 mL) was added. The organic phase was extracted from water, washed with sat. aq. cold $NaHCO_3$, dried using phase separator and concentrated in vacuo. Yielded white solid 293 mg (98%) with α:β 89:11.

Table S2: CAL-B recycling reactions with β-D-Glc pentaacetate 1 and α-D-Glc pentaacetate 42

CAL-B transesterification reactions were carried out according to general procedure (see below p S11) with β-D-Glc pentaacetate **1** (100 mg x 5) and 100 mg CAL-B, and α-D-Glc pentaacetate **42** (100 mg x 3) and 50 mg CAL-B. After each reaction was complete (β – 30 min, α – 23 h), the reaction mixture was filtered using glass filter and CAL-B washed with ~50 mL DCM. The filtrate was concentrated in vacuo, analysed by NMR and purified by silica gel column chromatography. Isolated yields of products **2** and **43** are shown in Table S2 and more detailed information about products in the "Experimental procedures and characterisation of products" (p S11 and S18 respectively). Filtered CAL-B was left on the filter to air-dry. CAL-B was weighted prior using it again, weight shown in Table S2. Increase in weight can be contributed to leftover DCM in the immobilised enzyme.

Table S2 CAL-B recycling reaction outcomes. "Yield" is isolated yield.

CAL-B Transesterification Reactions with β-D-Glc Pentaacetate **1**					
	Cycle 1	Cycle 2	Cycle 3	Cycle 4	Cycle 5
CAL-B weight (mg)	100	108	107	105	104
Product **2** Yield (mg)	80	73	69	76	79
Product **2** Yield (%)	90	81	77	85	89
CAL-B Transesterification Reactions with α-D-Glc Pentaacetate **42**					
CAL-B weight (mg)	50	51	50		
Product **43** Yield (mg)	70	70	71		
Product **43** Yield (%)	89	89	90		

General Experimental Information

Full assignment of ^{1}H and ^{13}C chemical shifts were based on the 1D and 2D (COSY, HSQC, HMBC etc.) FT NMR spectra measured with a Bruker Avance III 400 MHz instrument. Residual solvent signals were used ($CDCl_3$: δ = 7.26 ^{1}H NMR, δ = 77.2 ^{13}C NMR; CD_3OD: δ = 3.31 ^{1}H NMR, δ = 49.0 ^{13}C NMR; $(CD_3)_2SO$: δ = 2.50 ^{1}H NMR, δ = 39.5 ^{13}C NMR; D_2O: δ = 4.79 ^{1}H NMR) as internal standards. High-resolution mass spectra were recorded with an Agilent Technologies 6540 UHD Accurate-Mass QTOF LC/MS spectrometer by using AJ-ESI ionisation. Prior to analysis the instrument was calibrated in the mass range of m/z 50–3200. Optical rotations were obtained with an Anton Paar GWB Polarimeter MCP 500. Melting points were determined using polarising optical microscope Nagema-K8. Precoated Merck silica gel 60 F_{254} plates were used for TLC and column chromatography was performed with Merck 60 (0.040–0.063 mm) mesh silica gel. Commercial reagents, and solvents were generally used as received. DCM was distilled over CaH or phosphorous pentoxide, ethyl acetate (EtOAc) and acetone over phosphorus pentoxide, MeOH and toluene over sodium. Petroleum ether (PE) had a boiling point of 40-60 °C. Silicon oil bath on top of magnetic stirrer with heating was used as a heat source for reactions requiring heating. Immobilised *Candida antarctica* Lipase-B on hydrophobic acrylic resin, Novozyme N435, with 10000 (propyl laurate unit/g) activity was a kind gift from Novozymes A/S.

Starting Material Synthesis

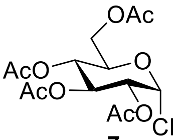
7

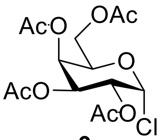
9

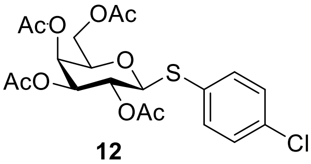
12

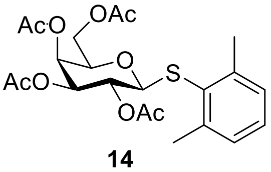
14

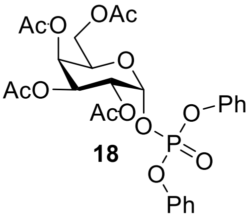
18

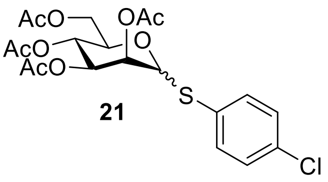
21

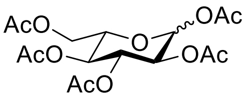
23

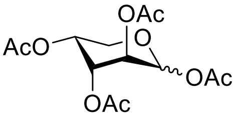
27

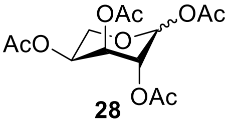
28

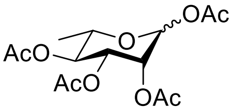
32

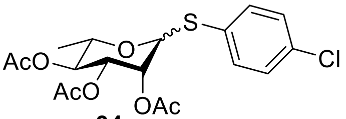
34

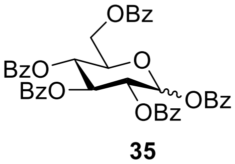
35

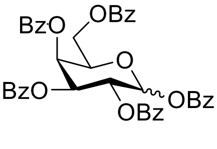
37

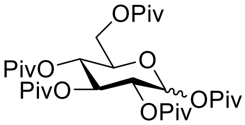
38

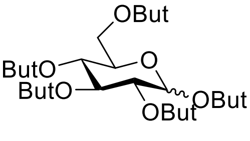
39

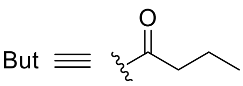

Glycoside chlorides (**7**[3], **9**[3])[4], thioglycosides (**12**[5], **21**[6])[7], 2,6-dimethylphenyl 2,3,4,6-tetra-*O*-acetyl-1-thio-D-glactopyranoside **14**[8], diphenyl 2,3,4,6-tetra-*O*-acetyl-α-D-galactopyranosyl phosphate **18**[9], 1,2,3,4,5-penta-*O*-L-glucopyranoside **23**[10], 1,2,3,4-tetra-*O*-acetyl-D/L-arabinopyranoside (**27**,**28**)[11], 1,2,3,4-tetra-*O*-acetyl-6-deoxy-L-mannopyranoside (**32**)[12], 4-chlorophenyl 2,3,4-tri-*O*-acetyl-6-deoxy-1-thio-L-mannopyranoside (**34**[13])[7], 1,2,3,4,6-penta-*O*-benzoyl-D-gluco/galactopyranoside (**35**[14], **37**[14,15])[15], 1,2,3,4,6-penta-*O*-pivaloyl-D-glucopyranoside **38**[16] and 1,2,3,4,6-penta-*O*-butyryl-D-glucopyranoside (**39**[17])[18] were synthesised by known literature procedures, with matching NMR values given in brackets. Commercially available saccharides (**1**, **3**, **5**, **20**, **42**) were used as received.

General Procedures

General procedure for transesterification reactions with CAL-B

In a manner analogous to Kanger et al. 2022[19] saccharide protected with ester functionalities (100 mg), MTBE or CPME (10 mL) and *n*-BuOH (3.5 equiv) were mixed at 45/60/90 °C. After saccharide was dissolved or after 10 minutes stirring was set to 100 rpm and 50/100/200% CAL-B (50/100/200 mg) was added. The reaction vessel was equipped with water cooling if necessary. The reaction was followed by TLC and upon completion the reaction mixture was filtered, immobilised enzymes were rinsed with DCM (~50 mL) and the filtrate was concentrate in vacuo. The crude mixture was purified by silica gel column chromatography (PE:EtOAc 2:1 →1:4) unless specified otherwise. Note: there is a small amount of leaching of resin occurring during DCM wash.

Experimental procedures and characterisation of products.

2,3,4,6-tetra-*O*-acetyl-D-glucopyranose (**2**)

According to a general procedure with β-Glc pentaacetate **1** (0.26 mmol, 100 mg), MTBE (10 mL), *n*-BuOH (3.5 equiv, 82 µL), 45 °C and 100% CAL-B (100 mg). Reaction was run for 30 minutes and after work up, the resulting crude was analysed by ^{1}H NMR. Comparison of 15-45 min reaction time outcomes shown in Scheme S1. Due to *n*-BuOH, *n*-BuOAc leftovers and some leaching from the resin, the crude yield was over 100%, α:β ~2:1. TLC – PE:EtOAc 1:2, R_f = 0.53;
α-^{1}H NMR (400 MHz, $CDCl_3$) δ 5.53 (t, *J* = 9.8 Hz, 1H, H-3), 5.46 (d, *J* = 3.4 Hz, 1H, H-1), 5.08 (t, *J* = 9.7 Hz, 1H, H-4), 4.90 (dd, *J* = 3.5, 10.2 Hz, 1H. H-2), 4.19-4.30 (m, 2H, H-5,6a/6b), 4.09-4.17 (m,1H, H-6a/6b), 3.32 (s, 1H, OH-1), 2.09 (s, 3H), 2.08 (s, 3H), 2.03 (s, 3H), 2.01 (s, 3H).
β-^{1}H NMR (400 MHz, $CDCl_3$) δ 5.25 (t, *J* = 9.6 Hz, 1H, H-3), 5.08 (t, *J* = 9.8 Hz, 1H, H-4), 4.88 (dd, *J* = 8.2, 9.7 Hz, 1H, H-2), 4.73 (d, *J* = 8.0 Hz, 1H, H-1), 4.19-4.30 (m, 1H, H-6a/6b), 4.09-4.17 (m, 1H, H-6a/6b), 3.75 (ddd, *J* = 2.3, 4.8, 10.1 Hz, 1H, H-5), 3.71 (s, 1H, OH-1), 2.09 (s, 3H), 2.09 (s, 3H), 2.03 (s, 3H), 2.01 (s, 3H).
NMR matches with previously reported values[20].

2,3,4,6-tetra-*O*-acetyl-D-galactopyranose (**4**)

According to general procedure with β-Gal pentaacetate **3** (2.56 mmol, 1 g), MTBE (100 mL), *n*-BuOH (3.5 equiv, 821 µL), 45 °C and 100% CAL-B (1 g). Reaction was run for 4 h and resulting in 851 mg (95%) of viscous oil with α:β 70:30. TLC – PE:EtOAc 1:2, R_f = 0.53; column chromatography eluent system PE:EtOAc 7:3 → 1:1;
α-^{1}H NMR (400 MHz, $CDCl_3$) δ 5.51 (t, *J* = 3.5 Hz, 1H, H-1), 5.46 (dd, *J* = 1.0, 3.3 Hz, 1H, H-4), 5.38–5.43 (m, 1H, H-3), 5.14 (ddd, *J* = 0.8, 3.5, 10.8 Hz, 1H, H-2), 4.46 (td, *J* = 0.8, 6.6 Hz, 1H, H-

5), 4.04–4.16 (m, 2H, H-6a,6b), 3.44 (d, *J* = 3.2 Hz, 1H, OH-1), 2.14 (s, 3H), 2.09 (s, 3H), 2.04 (s, 3H), 1.98 (s, 3H)
β-^{1}H NMR (400 MHz, $CDCl_3$) δ 5.38–5.43 (m, 1H, H-4), 5.05–5.08 (m, 2H, H-2,3), 4.66–4.72 (m, 1H, H-1), 4.04–4.16 (m, 2H, H-6a,6b), 3.95 (td, *J* = 1.0, 6.6 Hz, 1H, H-5), 3.84 (d, *J* = 8.9 Hz, 1H, OH-1), 2.15 (s, 3H), 2.09 (s, 3H), 2.04 (s, 3H), 1.98 (s, 3H)
NMR matches with previously reported values[21].

1,2,3,4-tetra-*O*-acetyl-α-D-galactopyranoside (**6**)

According to general procedure with α-Gal pentaacetate **5** (0.26 mmol, 100 mg), CPME (10 mL), *n*-BuOH (3.5 equiv, 82 µL), 60 °C and 200% CAL-B (200 mg). Reaction was run for 3 days resulting in 52 mg (58%) white solid. TLC – PE:EtOAc 1:2, R_f = 0.40; column chromatography eluent system PE:EtOAc 2:1 → 1:2; ^{1}H NMR (400 MHz, $CDCl_3$) δ 6.34 (d, *J* = 1.1 Hz, 1H, H-1), 5.47 (d, *J* = 1.8 Hz, 1H, H-4), 5.30-5.37 (m, 2H, H-2,3), 4.17 (t, *J* = 6.6 Hz, 1H, H-5), 3.65 (dd, *J* = 6.9, 11.6 Hz, 1H, H-6a/6b), 3.47 (dd, *J* = 6.1, 11.7 Hz, 1H, H-6a/6b), 2.15 (s, 3H), 2.14 (s, 3H), 2.00 (s, 3H), 1.99 (s, 3H); NMR matches with previously reported values[22].

2,3-di-*O*-acetyl-α-D-glucopyranosyl chloride (8)

According to general procedure with 2,3,4,6-tetra-*O*-acetyl-α-D-glucopyranosyl chloride **7** (0.28 mmol, 102 mg), MTBE (10 mL), *n*-BuOH (3.5 equiv, 89 µL), 45 °C and 100% CAL-B (102 mg). Reaction was run for 24 h resulting in 43 mg (55%) clear gel-like liquid; TLC – PE:EtOAc 1:1, R_f = 0.12; column chromatography eluent system PE:EtOAc 2:1 → 2:3; $[\alpha]_D^{20}$ +95.7 ($CHCl_3$, c 0.17); ^{1}H NMR (400 MHz, $CDCl_3$) δ 6.28 (d, *J* = 4.0 Hz, 1H, H-1), 5.40 (t, *J* = 9.7 Hz, 1H, H-3), 4.93 (dd, *J* = 4.0, 10.0 Hz, 1H, H-2), 4.03 (td, *J* = 3.3, 10.0 Hz, 1H, H-5), 3.90 (d, *J* = 3.3 Hz, 2H, H-6a,6b), 3.80 (t, *J* = 9.7 Hz, 1H, H-4), 3.00 (br.s, 2H, OH-4,6), 2.12 (s, 3H), 2.09 (s, 3H); ^{13}C{^{1}H} NMR (101 MHz, $CDCl_3$) δ 170.7, 170.3, 90.8 (C-1), 74.7 (C-5), 72.4 (C-3), 70.9 (C-2), 68.5 (C-4), 61.2 (C-6), 21.0, 20.8; HRMS (AJS-ESI): $[M+Na]^+$ for $C_{10}H_{15}ClO_7Na^+$ 305.0399 found 305.0389.

2,3,4-tri-*O*-acetyl-α-D-galactopyranosyl chloride (**10**) and **2,3,6-tri-*O*-acetyl-α-D-galactopyranosyl chloride (11)**

According to general procedure with 2,3,4,6-tetra-*O*-α-D-galactopyranosyl chloride **9** (0.28 mmol, 104 mg), CPME (10 mL), *n*-BuOH (3.5 equiv, 91 µL), 60 °C and 200% CAL-B (208 mg). Reaction was run for 2 days, resulting in 60 mg (66%) white solid, mixture of **10** (33%) and **11** (67%). Further purification with silica gel column chromatography led to 1:9 **10**:**11** mixture and ~1:1 **10**:**11** mixtures; TLC – PE:EtOAc 1:1, R_f = 0.40; column chromatography eluent system PE:EtOAc 4:1 → 1:9;
10-^{1}H NMR (400 MHz, $CDCl_3$) δ 6.38 (d, *J* = 4.0 Hz, 1H, H-1), 5.51 (dd, *J* = 0.9, 3.3 Hz, 1H, H-4), 5.43 (dd, *J* = 3.3, 10.7 Hz, 1H, H-3), 5.27 (dd, *J* = 4.0, 10.7 Hz, 1H, H-2), 4.36 (t, *J* = 6.2 Hz, 1H, H-5), 3.72 (dd, *J* = 6.7, 11.7 Hz, 1H, H-6a/6b), 3.55 (dd, *J* = 6.2, 11.7 Hz, 1H, H-6a/6b), 2.16 (s, 3H), 2.11 (s, 3H), 2.02 (s, 3H); ^{13}C{^{1}H} NMR (101 MHz, $CDCl_3$) δ 170.9, 170.3, 169.9, 91.4 (C-1), 72.1 (C-5), 68.3 (C-2), 68.1 (C-4), 67.3 (C-3), 60.5 (C-6), 20.8, 20.7, 20.7.
11-^{1}H NMR (400 MHz, $CDCl_3$) δ 6.35 (d, *J* = 1.9 Hz, 1H, H-1), 5.32-5.39 (m, 2H, H-2,3), 4.41 (dd, *J* = 5.9, 11.2 Hz, 1H, H-6a/6b), 4.34 (t, *J* = 6.0 Hz, 1H, H-5), 4.21 (dd, *J* = 6.1, 11.2 Hz, 1H, H-6a/6b), 4.17 (br.s, 1H, H-4), 2.58 (s, 1H, OH-4), 2.13 (s, 3H), 2.10 (s, 6H); ^{13}C{^{1}H} NMR (101 MHz, $CDCl_3$) δ 171.2, 170.3, 170.0, 91.7 (C-1), 70.8 (C-5), 69.5 (C-2/3), 67.9 (C-2/3), 67.2 (C-4), 62.2 (C-6), 21.0, 20.9, 20.8 HRMS (AJS-ESI) for **10** and **11** mixture: $[M+Na]^+$ for $C_{12}H_{17}ClO_8Na^+$ 347.0504 found 347.0495.

4-chlorophenyl 2,3,4-tri-*O*-acetyl-1-thio-β-D-galactopyranoside (13)

According to general procedure with 4-chlorophenyl 2,3,4,6-tetra-*O*-acetyl-1-thio-β-D-galactopyranoside **12** (0.21 mmol, 100 mg), MTBE (10 mL), *n*-BuOH (3.5 equiv, 67 µL), 60 °C and

200% CAL-B (200 mg). The reaction was complete in 48 h resulting in white solid 81 mg (88%); TLC – PE:EtOAc 2:3, R_f = 0.52; column chromatography eluent system PE:EtOAc 2:1 → 1:1; m.p. 35-37 (from $CHCl_3$); $[\alpha]_D^{20}$ +1.2 ($CHCl_3$, c 0.09); ^{1}H NMR (400 MHz, $CDCl_3$) δ 7.42-7.46 (m, 2H), 7.27-7.32 (m, 2H), 5.38 (d, *J* = 3.2 Hz, 1H, H-4), 5.22 (t, *J* = 9.9 Hz, 1H, H-2), 5.08 (dd, *J* = 3.3, 9.9 Hz, 1H, H-3), 4.69 (d, *J* = 9.8 Hz, 1H, H-1), 3.69-3.81 (m, 2H, H-5,6a/6b), 3.50 (ddd, *J* = 5.9, 11.3, 7.2 Hz, 1H, H-6a/6b), 2.20 (t, *J* = 6.7 Hz, 1H, OH-6), 2.12 (s, 3H), 2.09 (s, 3H), 1.99 (s, 3H); $^{13}C\{^1H\}$ NMR (101 MHz, $CDCl_3$) δ 171.2, 170.1, 169.5, 134.7, 134.2 (2C), 130.5, 129.2 (2C), 86.1 (C-1), 77.5 (C-5), 72.1 (C-3), 68.1 (C-4), 67.5 (C-2), 60.8 (C-6), 20.9, 20.8, 20.7; HRMS (AJS-ESI): $[M+Na]^+$ for $C_{18}H_{21}ClO_8SNa^+$ 455.0538 found 455.0520.

2,6-dimethylphenyl 2,3,4-tri-*O*-acetyl-1-thio-D-galactopyranoside (15)

According to general procedure with 2,6-dimethylphenyl 2,3,4,6-tetra-*O*-acetyl-1-thio-D-galactopyranoside **14** (0.22 mmol, 103 mg), MTBE (10 mL), *n*-BuOH (3.5 equiv, 70 µL), 60 °C and 200% CAL-B (206 mg). The reaction was complete in 48 h resulting in sticky oil 83 mg (95%) α:β 78:22. TLC – DCM:EtOAc 1:4, R_f = 0.74; column chromatography eluent system PE:EtOAc 3:1 → 1:1;

α-^{1}H NMR (400 MHz, $CDCl_3$) δ 5.58 (dd, *J* = 1.1, 3.5 Hz, 1H, H-1),7.08-7.20 (m, 3H), 5.51 (d, *J* = 1.2 Hz, 1H, H-4), 5.35-5.41 (m, 2H, H-2,3), 4.58 (t, *J* = 6.6 Hz, 1H, H-5), 3.61 (dd, *J* = 6.7, 11.6 Hz, 1H, H-6a/6b), 3.49 (dd, *J* = 6.3, 11.8 Hz, 1H, H-6a/6b), 2.49 (s, 6H), 2.15 (s, 3H), 2.13 (s, 3H), 2.04 (s, 3H); $^{13}C\{^1H\}$ NMR (101 MHz, $CDCl_3$) δ 171.2, 170.6, 170.0, 143.4, 129.1, 128.6, 128.5, 86.8 (C-1), 70.7 (C-5), 68.9 (C-4), 68.5 (C-2), 68.1 (C-3), 61.0 (C-6), 22.2, 20.9, 20.8, 20.8;

β-^{1}H NMR (400 MHz, $CDCl_3$) δ 7.08-7.20 (m, 3H), 5.32-5.43 (m, 2H, H-2,4), 5.04 (dd, *J* = 3.4, 9.9 Hz, 1H. H-3), 4.43 (d, *J* = 10.2 Hz, 1H, H-1), 3.63 (dd, *J* = 6.7, 10.2 Hz, 1H, H-6a/6b), 3.57 (t, *J* = 6.2 Hz, 1H, H-5), 3.45 (dd, *J* = 5.5, 11.4 Hz, 1H, H-6a/6b), 2.54 (s, 6H), 2.21 (s, 3H), 2.13 (s, 3H), 2.01 (s, 3H); $^{13}C\{^1H\}$ NMR (101 MHz, $CDCl_3$) δ 170.6, 170.1, 169.7, 143.9, 131.3, 130.9, 129.6, 89.2 (C-2), 77.1 (C-5), 72.1 (C-3), 68.3 (C-2/4), 68.3 (C-2/4), 60.8 (C-6), 22.5, 20.9, 20.8, 20.8.

HRMS (AJS-ESI): $[M+Na]^+$ for $C_{20}H_{26}O_8SNa^+$ 449.1241 found 499.1225.

4-chlorophenyl (2,3,4,6-tetra-*O*-acetyl-β-D-galactopyranosyl)-(1→4)-2,3,6-tri-*O*-acetyl-1-thio-D-glucopyranoside (16)

Following known procedure[7] with lactose octaacetate[23] (1.47 mmol, 1 g), 4-chlorothiophenol (1.22 equiv 260 mg), $BF_3{\cdot}Et_2O$ (3.0 equiv, 546 µL), dry DCM (3 mL) under argon. The reaction was complete in 4 days resulting in white solid 639 mg (57%) with α:β ratio of 1:4; TLC – PE:EtOAc 1:1, R_f = 0.47; column chromatography eluent system PE:EtOAc 2:1 → 1:1; m.p. 80-83 °C (from $CHCl_3$);

α-^{1}H NMR (400 MHz, $CDCl_3$) δ 7.34-7.39 (m, 2H), 7.25-7.30 (m, 2H) 5.79 (d, *J* = 5.7 Hz, 1H, H-1), 5.40 (dd, *J* = 8.9, 10.0 Hz, 1H, H-3), 5.35 (dd, *J* = 0.9, 3.5 Hz, 1H, H'-4), 5.13 (dd, *J* = 7.9, 10.4 Hz, 1H, H'-2), 5.02 (dd, *J* = 5.8, 10.1 Hz, 1H, H-2), 4.96 (dd, *J* = 3.4, 10.4 Hz, 1H, H'-3), 4.49 (d, *J* = 7.9 Hz, 1H, H'-1), 4.42 (ddd, *J* = 2.0, 5.9, 10.2 Hz, 1H, H-5), 4.37 (dd, *J* = 2.0, 11.9 Hz, 1H, H-6a/6b), 4.02-4.18 (m, 3H, H-6a/6b, H'-6a,6b), 3.88 (t, *J* = 6.5 Hz, 1H, H'-5), 3.74 (dd, *J* = 8.7, 10.0 Hz, 1H, H-4), 2.16 (s, 3H), 2.09 (s, 3H), 2.07 (s, 3H), 2.05 (s, 3H), 2.05 (s, 3H), 1.96 (s, 3H). $^{13}C\{^1H\}$ NMR (101 MHz, $CDCl_3$) δ 170.5, 170.4, 170.3, 170.2, 170.1, 169.5, 169.4, 134.1, 133.1 (2C), 131.4, 129.6 (2C), 101.2 (C'-1), 85.0 (C-1), 76.1 (C-4), 71.2 (C'-3), 70.9 (C'-5/C-2), 70.9 (C'-5/C-2), 70.0 (C-3), 69.2 (C-5), 66.8 (C'-4), 62.2 (C-6), 61.0 (C'-6), 22.1, 22.0 (2C), 21.9, 21.8 (2C), 21.7.

β-^{1}H NMR (400 MHz, $CDCl_3$) δ 7.40-7.44 (m, 2H), 7.225-7.30 (m, 2H), 5.33 (dd, *J* = 0.8, 3.3 Hz, 1H, H'-4), 5.20 (t, *J* = 9.1 Hz, 1H, H-3), 5.09 (dd, *J* = 7.9, 10.4 Hz, 1H, H'-2), 4.94 (dd, *J* = 3.4, 10.4 Hz, 1H, H'-3), 4.84 (t, *J* = 9.6 Hz, 1H, H-2), 4.60 (d, *J* = 10.0 Hz, 1H, H-1), 4.53 (dd, *J* = 1.9, 11.9 Hz, 1H, H-6a/6b), 4.46 (d, *J* = 7.9 Hz, 1H, H'-1), 4.02-4.18 (m, 3H, H-6a/6b, H'-6a,6b), 3.85 (t, *J* = 6.5 Hz, 1H, H'-5), 3.71 (t, *J* = 9.5 Hz, 1H, H-4), 3.61 (ddd, *J* = 1.9, 5.3, 9.9 Hz, 1H, H-5), 2.14 (s, 3H), 2.10 (s, 3H), 2.08 (s, 3H), 2.04 (s, 3H), 2.03 (s, 3H), 2.02 (s, 3H), 1.95 (s, 3H). $^{13}C\{^1H\}$ NMR (101 MHz, $CDCl_3$) δ 170.4, 170.3, 170.2, 170.2, 169.8, 169.6, 169.1, 135.2 (2C), 135.1, 129.4, 129.1 (2C),

101.2 (C'-1), 85.0 (C-1), 76.9 (C-5), 76.1 (C-4), 73.9 (C-3), 71.1 (C'-3), 70.8 (C'-5), 70.2 (C-2), 69.2 (C'-2), 66.7 (H'-4), 62.0 (C-6), 60.8 (C'-6), 22.1, 22.0 (2C), 21.9, 21.8 (2C), 21.7
HRMS (AJS-ESI): $[M+Na]^+$ for $C_{32}H_{39}ClO_{17}SNa^+$ 785.1489 found 785.1475.

4-chlorophenyl (2,3,4,6-tetra-*O*-acetyl-β-D-galactopyranosyl)-(1→4)-2,3-di-*O*-acetyl-1-thio-β-D-glucopyranoside (17)

According to general procedure with 4-chlorophenyl (2,3,4,6-tetra-*O*-acetyl-β-D-galactopyranosyl)-(1→4)-2,3,6-tri-*O*-acetyl-1-thio-D-glucopyranoside **16** (0.13 mmol, 100 mg), CPME (10 mL), *n*-BuOH (3.5 equiv, 42 µL), 60 °C and 200% CAL-B (200 mg). The reaction was complete in 4 h resulting in white solid 71 mg (75%); TLC – PE:EtOAc 1:2, R_f = 0.52; column chromatography eluent system DCM:EtOAc 1:0 → 7:3; m.p. 86-89 °C (from $CHCl_3$); $[\alpha]_D^{20}$ +19.0 ($CHCl_3$, c 0.06); ^1H NMR (400 MHz, $CDCl_3$) δ 7.36-7.41 (m, 2H), 7.27-7.32 (m, 2H), 5.33 (dd, *J* = 0.7, 3.3 Hz, 1H, H'-4), 5.20 (t, *J* = 9.3 Hz, 1H, H-3), 5.09 (dd, *J* = 7.9, 10.4 Hz, 1H, H'-2), 4.97 (dd, *J* = 3.4, 10.4 Hz, 1H, H'-3), 4.86 (t, *J* = 9.7 Hz, 1H, H-2), 4.67 (d, *J* = 10.0 Hz, 1H, H-1), 4.58 (d, *J* = 7.9 Hz, 1H, H'-1), 4.11 (dd, *J* = 6.4, 11.3 Hz, 1H, H'-6a/6b), 4.06 (dd, *J* = 7.3, 11.1 Hz, 1H, H'-6a/6b), 3.85-3.94 (m, 3H, H-4,6a/6b, H'-5), 3.73 (dd, *J* = 3.2, 12.2 Hz, 1H, H-6a/6b), 3.42 (td, *J* = 2.5, 9.9 Hz, 1H, H-5), 2.14 (s, 3H), 2.08 (s, 3H), 2.04 (s, 3H), 2.03 (s, 3H), 2.03 (s, 3H), 1.96 (s, 3H); $^{13}C\{^1H\}$ NMR (101 MHz, $CDCl_3$) δ 170.5, 170.3, 170.2, 169.9, 169.7, 169.2, 135.0, 134.6 (2C), 130.0, 129.4 (2C), 101.0 (C'-1), 85.6 (C-1), 79.2 (C-5), 74.6 (C-4), 74.1 (C-3), 71.1 (C'-3), 70.7 (C'-5), 70.4 (C-2), 69.3 (C'-2), 66.8 (C'-4), 60.9 (C'-6), 60.6 (C-6), 20.9 (2C), 20.8, 20.8, 20.8, 20.7; HRMS (AJS-ESI): $[M+Na]^+$ for $C_{30}H_{37}ClO_{16}SNa^+$ 743.1383 found 743.1368.

5,5-dimethyl 2,3,4,6-tetra-*O*-acetyl-D-galactopyranosyl 1,3,2-dioxaphosphinane-2-oxide (19)

Following known procedure[24] 2,3,4,6-tetra-*O*-acetyl-D-galactopyranose **4** (2.39 mmol, 1 g), was dissolved in dry DCM (24 mL) under argon, 2-chloro-5,5-dimethyl-1,3,2-dioxaphosphinane-2-oxide (3 equiv, 1.323 g) and 4-dimethylaminopyridine (3 equiv, 0.876 g) were added at room temperature. After stirring for 24 h, the reaction was diluted with DCM, washed with sat. aq. $NaHCO_3$, dried using phase separator and concentrated in vacuo. The crude was purified by silica gel column chromatography resulting in clear honey-like oil 1.0 g (86%) with α:β 36:64; TLC – PE:EtOAc 1:3, R_f = 0.55; column chromatography eluent system PE:EtOAc 1:0 → 3:7;
Anomeric enrichement – according to general procedure with 5,5-dimethyl 2,3,4,6-tetra-*O*-acetyl-D-galactopyranosyl 1,3,2-dioxaphosphinane-2-oxide **19** (82 mg), MTBE (8.2 mL), *n*-BuOH (3.5 equiv, 43 µL), 60 °C and 200% CAL-B. The reaction was run for 48 h resulting in clear honey-like oil 72 mg (87%), α-anomer only.
α-^1H NMR (400 MHz, $CDCl_3$) δ 5.97 (dd, *J* = 3.3, 7.1 Hz, 1H, H-1), 5.51 (dd, *J* = 1.1, 3.1 Hz, 1H, H-4), 5.28-5.37 (m, 1H, H-3), 5.25 (ddd, *J* = 1.9, 3.3, 10.9 Hz, 1H, H-2), 4.42 (t, *J* = 6.8 Hz, 1H, H-5), 4.08-4.28 (m, 4H, H-6a,6b, 2x$POCH_2$), 3.94-4.04 (m, 2H, 2x$POCH_2$), 2.16 (s, 3H), 2.10 (s, 3H), 2.04 (s, 3H), 2.01 (s, 3H), 1.26 (s, 3H, CCH_3), 0.95 (s, 3H, CCH_3); $^{13}C\{^1H\}$ NMR (101 MHz, $CDCl_3$) δ 170.5, 170.3, 170.2, 170.2, 94.3 (d, *J* = 5.0 Hz, C-1), 78.4 (d, *J* = 6.7 Hz $POCH_2$), 78.3 (d, *J* = 6.5 Hz $POCH_2$), 68.4 (C-5), 67.4 (C-4), 67.1 (C-3), 66.9 (d, *J* = 6.0 Hz, C-2), 61.5 (C-6), 32.3 (d, *J* = 6.1 Hz, $\underline{C}(CH_3)_2$, 21.7 ($C\underline{CH_3}$), 20.8, 20.7 (2C, $C\underline{CH_3}$), 20.7, 20.5;
β-^1H NMR (400 MHz, $CDCl_3$) δ 5.42 (dd, *J* = 0.7, 3.3 Hz, 1H, H-4), 5.28-5.37 (m, 2H, H-1,2), 5.03 (dd, *J* = 3.5, 10.2 Hz, 1H, H-3), 4.08-4.26 (m, 3H, H-6a/6b, 2x$POCH_2$), 3.94-4.04 (m, 2H, H-5,6a/6b), 3.89 (ddd, *J* = 2.8, 5.2, 22.6 Hz, 1H. $POCH_2$), 3.86 (ddd, *J* = 2.8, 5.1, 22.6 Hz, 1H, $POCH_2$), 2.17 (s, 3H), 2.09 (s, 3H), 2.04 (s, 3H), 1.99 (s, 3H), 1.28 (s, 3H, CCH_3), 0.87 (s, 3H, CCH_3); $^{13}C\{^1H\}$ NMR (101 MHz, $CDCl_3$) δ 170.4, 170.2, 170.1, 169.9, 96.8 (d, *J* = 4.3 Hz, C-1), 78.6 (d, *J* = 7.2 Hz $POCH_2$), 78.3 (d, *J* = 6.6 Hz $POCH_2$), 72.0 (C-5), 70.5 (C-3), 68.9 (d, *J* = 7.3 Hz, C-2),

66.8 (C-4), 61.0 (C-6), 32.3 (d, *J* = 6.1 Hz, <u>C</u>(CH_3)$_2$), 21.9 (C<u>C</u>$\underline{H_3}$), 20.9, 20.8 (2C), 20.6, 20.2 (C<u>C</u>$\underline{H_3}$);
HRMS (AJS-ESI): $[M+Na]^+$ for $C_{19}H_{29}O_{13}PNa^+$ 519.1238 found 519.1220.

4-chlorophenyl 2,3-di-*O*-acetyl-1-thio-β-D-mannopyranoside (22)

According to general procedure with 4-chloropnenyl 2,3,4,6-tetra-*O*-acetyl-1-thio-D-mannopyranoside **21** (0.211 mmol, 100 mg), CPME (10 mL), *n*-BuOH (3.5 equiv, 67 μL), 60 °C and 200% CAL-B (200 mg). The reaction was run for 23 h resulting in hazy syrup 67 mg (81%); TLC – PE:EtOAc 1:2, R_f = 0.25; column chromatography eluent system PE:EtOAc 1:1 → 19:31; $[\alpha]_D^{20}$ +71.3 ($CHCl_3$, c 0.1); ^{1}H NMR (400 MHz, $CDCl_3$) δ 7.39 (appt. d, *J* = 8.3 Hz, 2H), 7.27 (appt. d, *J* = 6.0 Hz, 2H), 5.45 (d, *J* = 1.4 Hz, 1H, H-2), 5.38 (s, 1H, H-1), 5.15 (dd, *J* = 3.1, 9.8 Hz, 1H, H-3), 4.18 (td, *J* = 3.4, 9.6 Hz, 1H, H-5), 4.06 (t, *J* = 9.8 Hz, 1H, H-4), 3.88 (s, 2H, H-6a,6b), 2.09 (s, 3H), 2.07 (s, 3H); ^{13}C{^{1}H} NMR (101 MHz, $CDCl_3$) δ 171.0, 170.1, 134.5, 133.6 (2xC), 131.4, 129.5 (2xC), 86.1 (C-1), 73.7 (C-5), 72.1 (C-3), 71.2 (C-2), 66.1 (C-4), 62.1 (C-6), 20.9, 20.9; HRMS (AJS-ESI): $[M+Na]^+$ for $C_{16}H_{19}ClO_7SNa^+$ 413.0432 found 413.0416.

1,2,3,4-tetra-*O*-acetyl-L-glucopyranoside (24)

According to general procedure with L-glucose pentaacetate **23** (0.256 mmol, 100 mg), MTBE (10 mL), *n*-BuOH (3.5 equiv, 82 μL), 45 °C and 50% CAL-B (50 mg). The reaction was run for 6 h resulting in white solid 75 mg (85%) with α:β 93:7; TLC – PE:EtOAc 1:2, R_f = 0.51; column chromatography eluent system PE:EtOAc 7:3 → 9:11; m.p. 89-92 °C (from DCM);
α - ^{1}H NMR (400 MHz, $CDCl_3$) δ 6.33 (d, *J* = 3.7 Hz, 1H, H-1), 5.52 (t, *J* = 9.9 Hz, 1H, H-3), 5.10 (t, *J* = 9.9 Hz, 1H, H-4), 5.06 (dd, *J* = 3.7, 10.3 Hz, 1H, H-2), 3.92 (ddd, *J* = 2.2, 4.1, 10.4 Hz, 1H, H-5), 3.72 (ddd, *J* = 2.1, 7.8, 12.8 Hz, 1H, H-6a/6b), 3.57 (ddd, *J* = 4.4, 5.9, 12.8 Hz, 1H, H-6a/6b), 2.26 (t, *J* = 6.5 Hz, 1H, OH-6), 2.17 (s, 3H), 2.07 (s, 3H), 2.03 (s, 3H), 2.01 (s, 3H); ^{13}C{^{1}H} NMR (101 MHz, $CDCl_3$) δ 170.3, 170.3, 169.8, 169.0, 89.2 (C-1), 72.2 (C-5), 69.7 (C-3), 69.5 (C-2), 68.4 (C-4), 61.0 (C-6), 21.0, 20.8, 20.7, 20.6;
β - ^{1}H NMR (400 MHz, $CDCl_3$) δ 5.72 (d, *J* = 8.3 Hz, 1H, H-1), 5.30 (t, *J* = 9.5 Hz, 1H, H-3), 5.03-5.13 (m, 2H, H-2, 4), 3.67-3.80 (m, 1H, H-6a/6b), 3.64 (ddd, *J* = 2.1, 4.1, 9.9 Hz, 1H, H-5), 3.54-3.61 (m, 1H, H-6a/6b), 2.27 (br.s, 1H, OH-6), 2.10 (s, 3H), 2.06 (s, 3H), 2.02 (s, 3H), 2.02 (s, 3H), ^{13}C{^{1}H} NMR (101 MHz, $CDCl_3$) δ 170.4, 170.2, 169.4, 169.2, 91.8 (C-1), 75.0 (C-5), 72.7 (C-3), 70.5 (C-2), 68.3 (C-4), 60.9 (C-6), 20.9, 20.7, 20.7, 20.7
HRMS (AJS-ESI): $[M+Na]^+$ for $C_{14}H_{20}O_{10}Na^+$ 371.0949 found 371.0965.

4-chlorophenyl 2,3,4,6-tetra-*O*-acetyl-1-thio-L-glucopyranoside (25)

Following known procedure[7] L-glucose pentaacetate **23** (1.28 mmol, 500 mg), 4-chlorothiophenol (1.2 equiv, 226 mg), $BF_3{\cdot}Et_2O$ (2.4 equiv, 386 μL), dry DCM (2.5 mL) and under argon. The reaction was run for 24 h resulting in white solid 331 mg (54%) with α:β 7:93; TLC – PE:EtOAc 2:1, R_f = 0.36; column chromatography eluent system PE:EtOAc 1:0 → 0:1; m.p. 104-107 °C (from DCM);
α - ^{1}H NMR (400 MHz, $CDCl_3$) δ 7.34-7.38 (m, 2H), 7.26-7.30 (m, 2H), 5.88 (d, *J* = 5.7 Hz, 1H, H-1), 5.41 (t, *J* = 9.8 Hz, 1H, H-3), 5.08 (dd, *J* = 5.8, 10.3 Hz, 1H, H-2), 5.06 (dd, *J* = 9.2, 9.9 Hz, 1H, H-4), 4.51 (ddd, *J* = 2.2, 5.3, 10.2 Hz, 1H, H-5), 4.25 (dd, *J* = 5.5, 12.6 Hz, 1H, H-6a/6b), 4.02 (dd, *J* = 2.2, 12.3 Hz, 1H, H-6a/6b), 2.09 (s, 3H), 2.04 (s, 3H), 2.03 (s, 3H), 2.02 (s, 3H); ^{13}C{^{1}H} NMR (101 MHz, $CDCl_3$) δ 170.0, 170.0, 169.7, 169.7, 133.3 (2xC), 131.0, 120.6, 129.4 (2xC), 85.1 (C-1), 70.8 (C-2), 70.4 (C-3), 68.6 (C-4), 68.4 (C-5), 62.0 (C-6), 20.9, 20.8, 20.7, 20.7
β - ^{1}H NMR (400 MHz, $CDCl_3$) δ 7.41-7.45 (m, 2H), 7.26-7.30 (m, 2H), 5.21 (t, *J* = 9.4 Hz, 1H, H-3), 5.00 (t, *J* = 9.8 Hz, 1H, H-4), 4.92 (t, *J* = 9.6 Hz, 1H, H-2), 4.64 (d, *J* = 10.0 Hz, 1H, H-1), 4.20 (dd, *J* = 4.7, 12.3 Hz, 1H, H-6a/6b), 4.16 (dd, *J* = 2.8, 12.3 Hz, 1H, H-6a/6b), 3.70 (ddd, *J* = 2.8, 4.8, 10.0 Hz, 1H, H-5), 2.08 (s, 3H), 2.07 (s, 3H), 2.01 (s, 3H), 1.98 (s, 3H); ^{13}C{^{1}H} NMR (101 MHz, $CDCl_3$) δ 170.6, 170.3, 169.5, 169.3, 135.1, 135.1 (2xC), 129.6, 129.2 (2xC), 85.3 (C-1), 76.0 (C-5), 74.0 (C-

3), 69.9 (C-2), 68.2 (C-4), 62.1 (C-6), 20.8 (2xC), 20.7, 20.7;
HRMS (AJS-ESI): $[M+Na]^+$ for $C_{20}H_{23}ClO_9SNa^+$ 497.0644 found 497.0655.

4-chlorophenyl 2,3,4-tri-*O*-acetyl-1-thio-L-glucopyranoside (26)

According to general procedure with 4-chlorophenyl 2,3,4-tetra-*O*-acetyl-1-thio-L-glucopyranoside **25** (0.211 mmol, 100 mg), MTBE (10 mL), *n*-BuOH (3.5 equiv, 67 µL), 45 °C and 100% CAL-B (100 mg). The reaction was run for 3 h resulting in white solid 88 mg (97%); TLC – PE:EtOAc 1:2, R_f = 0.65; column chromatography eluent system PE:EtOAc 4:1 → 3:2; m.p. 131-135 °C (from DCM);
α - ^{1}H NMR (400 MHz, $CDCl_3$) δ 7.34-7.38 (m, 2H), 7.27-7.33 (m, 2H), 5.89 (d, *J* = 5.7 Hz, 1H, H-1), 5.47 (t, *J* = 9.9 Hz, 1H, H-3), 5.06 (dd, *J* = 5.7, 10.3 Hz, 1H, H-2), 5.05 (t, *J* = 9.8 Hz, 1H, H-4), 4.29 (ddd, *J* = 2.3, 3.9, 10.2 Hz, 1H, H-5), 3.49-3.79 (m, 2H, H-6a, 6b), 2.20 (t, *J* = 6.6 Hz, 1H, OH-6), 2.10 (s, 3H), 2.08 (s, 3H), 2.05 (s, 3H); 170.7, 170.3, 170.0, 134.9, 133.4 (Cx2), 131.1, 129.5 (Cx2), 85.3 (C-1), 71.1 (C-2), 70.7 (C-5), 70.1 (C-3), 68.9 (C-4), 61.1 (C-6), 20.8, 20.8, 20.7
β - ^{1}H NMR (400 MHz, $CDCl_3$) δ 7.39-7.45 (m, 2H), 7.27-7.33 (m, 2H), 5.26 (t, *J* = 9.4 Hz, 1H, H-3), 4.98 (t, *J* = 9.7 Hz, 1H, H-4), 4.92 (t, *J* = 9.6 Hz, 1H, H-2), 4.69 (d, *J* = 10.1 Hz, 1H, H-1), 3.74 (ddd, *J* = 1.7, 8.1, 12.2 Hz, 1H, H-6a/6b), 3.58 (td, *J* = 5.4, 18.0 Hz, 1H, H-6a/6b), 3.53 (ddd, *J* = 2.1, 4.9, 9.8 Hz, 1H, H-5), 2.20 (t, *J* = 6.6 Hz, 1H, OH-6), 2.08 (s, 3H), 2.04 (s, 3H), 1.99 (s, 3H); ^{13}C{^{1}H} NMR (101 MHz, $CDCl_3$) δ 170.3, 170.2, 169.4, 135.1, 134.8 (2xC), 129.7, 129.4 (2xC), 85.4 (C-1), 78.5 (C-5), 73.8 (C-3), 70.1 (C-4), 68.5 (C-2), 61.5 (C-6), 20.9, 20.7 (2xC)
HRMS (AJS-ESI): $[M+Na]^+$ for $C_{18}H_{21}ClO_8SNa^+$ 455.0538 found 455.0549.

4-chlorophenyl 2,3,4-tri-*O*-acetyl-1-thio-D-arabinopyranoside (29)

Following known procedure[7] with 1,2,3,4-tetra-*O*-acetyl-D-arabinopyranoside **27** (0.63 mmol, 200 mg), 4-chlorothiophenol (1.2 equiv, 111 mg), $BF_3·Et_2O$ (2.4 equiv, 189 µL), dry DCM (1 mL) and under argon. The reaction was run for 20 h resulting in clear honey-like liquid 251 mg (99%) with α:β 17:83 and <5% furanose. TLC – PE:EtOAc 5:1, R_f = 0.33; column chromatography eluent system PE:EtOAc 9:1 → 83:17;
α-^{1}H NMR (400 MHz, $CDCl_3$) δ 7.33-7.38 (m, 2H), 7.26-7.30 (m, 2H), 5.85 (d, *J* = 5.1 Hz, 1H, H-1), 5.33-5.38 (m, 2H, H-2,4), 5.22-5.26 (m, 1H, H-3), 4.34 (dd, *J* = 1.3, 13.1 Hz, 1H, H-5a/5b), 3.74 (dd, *J* = 2.8, 13.0 Hz, 1H, H-5a/5b), 2.12 (s, 3H), 2.11 (s, 3H), 2.04 (s, 3H); ^{13}C{^{1}H} NMR (101 MHz, $CDCl_3$) δ 170.2, 169.9, 169.5, 133.6, 133.0 (2xC), 131.6, 129.4 (2xC), 86.0 (C-1), 68.6 (C-2,4), 67.6 (C-3), 61.5 (C-5), 21.0, 20.9, 20.8.
β-^{1}H NMR (400 MHz, $CDCl_3$) δ 7.41-7.46 (m, 2H), 7.26-7.30 (m, 2H), 5.25-5.29 (m, 1H, H-4), 5.21 (t, *J* = 8.0 Hz, 1H, H-2), 5.10 (dd, *J* = 3.3, 8.4 Hz, 1H, H-3), 4.76 (d, *J* = 7.7 Hz, 1H, H-1), 4.15 (dd, *J* = 4.3, 12.7 Hz, 1H, H-5a/5b), 3.67 (dd, *J* = 2.1, 12.7 Hz, 1H, H-5a/5b), 2.10 (s, 3H), 2.09 (s, 3H), 2.05 (s, 3H); ^{13}C{^{1}H} NMR (101 MHz, $CDCl_3$) δ 170.3, 170.1, 169.5, 134.5, 134.1 (2xC), 131.5, 129.2 (2xC), 86.6 (C-1), 70.5 (C-3), 68.5 (C-2), 67.5 (C-4), 65.4 (C-5), 21.0, 20.9. 20.8.
HRMS (AJS-ESI): $[M+Na]^+$ for $C_{17}H_{19}ClO_7SNa^+$ 425.0432 found 425.0418.

4-chlorophenyl 2-O-acetyl-1-thio-β-D-arabinopyranoside (30)

According to general procedure with 4-chlorophenyl 2,3,4-tri-*O*-acetyl-1-thio-D-arabinopyranoside **29** (0.248 mmol, 100 mg), MTBE (10 mL), *n*-BuOH (3.5 equiv, 80 µL), 45 °C and 100% CAL-B (100 mg). The reaction was run for 7 h resulting in clear film-like solid 61 mg (77%); m.p. 100-102 °C (from $CHCl_3$); TLC – PE:EtOAc 1:2, R_f = 0.23; column chromatography eluent system PE:EtOAc 9:11 → 2:3; $[\alpha]_D^{20}$ +9.4 ($CHCl_3$, c 0.1); ^{1}H NMR (400 MHz, $CDCl_3$) δ 7.41-7.45 (m, 2H), 7.26-7.30 (m, 2H), 5.02 (t, *J* = 7.8 Hz, 1H, H-2), 4.70 (d, *J* = 7.8 Hz, 1H, H-1), 4.14 (dd, *J* = 4.2, 12.4 Hz, 1H, H-5a/5b), 3.99 (dd, *J* = 3.8, 6.0 Hz, 1H, H-4), 3.78 (dd, *J* = 3.5, 7.8 Hz, 1H, H-3), 3.60 (dd, *J* = 2.2, 12.4 Hz, 1H, H-5a/5b), 2.15 (s, 3H; ^{13}C{^{1}H} NMR (101 MHz, $CDCl_3$) δ 171.0, 134.4, 133.9 (2xC), 131.7, 129.3 (2xC), 86.1 (C-1), 72.0 (C-2), 72.0 (C-3), 67.9 (C-4), 67.5 (C-5), 21.2; HRMS (AJS-ESI): $[M+Na]^+$ for $C_{13}H_{15}ClO_5SNa^+$ 341.0221 found 341.0207.

4-chlorophenyl 2,3,4-tri-*O*-acetyl-1-thio-L-arabinopyranoside (**31**)

Following known procedure[7] with 1,2,3,4-tetra-*O*-acetyl-L-arabinopyranoside **28** (0.63 mmol, 200 mg), 4-chlorothiophenol (1.2 equiv, 111 mg), $BF_3{\cdot}Et_2O$ (2.4 equiv, 189 µL), dry DCM (1 mL) and under argon. The reaction was run for 20 h resulting in clear honey-like liquid 239 mg (94%) with α:β 17:83 and <5% furanose; TLC – PE:EtOAc 5:1, R_f = 0.33; column chromatography eluent system PE:EtOAc 9:1 → 83:17;
α-^{1}H NMR (400 MHz, $CDCl_3$) δ 7.33-7.38 (m, 2H), 7.26-7.30 (m, 2H), 5.85 (d, *J* = 5.1 Hz, 1H, H-1), 5.33-5.39 (m, 2H, H-2,4), 5.22-5.26 (m, 1H), 4.35 (dd, *J* = 1.5, 13.1 Hz, 1H, H-5a/5b), 3.74 (dd, *J* = 2.8, 13.0 Hz, 1H, H-5a/5b), 2.13 (s, 3H), 2.11 (s, 3H), 2.04 (s, 3H); $^{13}C\{^1H\}$ NMR (101 MHz, $CDCl_3$) δ 170.2, 169.9, 169.5, 133.6, 133.0 (2xC), 131.6, 129.4 (2xC), 86.0 (C-1), 68.6 (C-2,4), 67.7 (C-3), 61.5 (C-5), 21.0, 20.9, 20.9;
β-^{1}H NMR (400 MHz, $CDCl_3$) δ 7.41-7.46 (m, 2H), 7.26-7.31 (m, 2H), 5.25-5.29 (m, 1H, H-4), 5.21 (t, *J* = 8.0 Hz, 1H, H-2), 5.10 (dd, *J* = 3.3, 8.3 Hz, 1H, H-3), 4.76 (d, *J* = 7.7 Hz, 1H, H-1), 4.15 (dd, *J* = 4.3, 12.7 Hz, 1H, H-5a/5b), 3.67 (dd, *J* = 2.1, 12.7 Hz, 1H, H-5a/5b), 2.10 (s, 3H), 2.09 (s, 3H), 2.05 (s, 3H); $^{13}C\{^1H\}$ NMR (101 MHz, $CDCl_3$) δ 170.3, 170.0, 169.5, 134.5, 134.1 (2xC), 131.5, 129.2 (2xC), 86.6 (C-1), 70.5 (C-3), 68.5 (C-2), 67.5 (C-4), 65.5 (C-5), 21.0, 21.0, 20.8.
HRMS (AJS-ESI): $[M+Na]^+$ for $C_{17}H_{19}ClO_7SNa^+$ 425.0432 found 425.0412.

2,3,4-tri-*O*-acetyl-6-deoxy-L-mannopyranose (**33**)

According to general procedure with 1,2,3,4-tetra-*O*-acetyl-6-deoxy-L-mannopyranoside **32** (0.386 mmol, 109 mg), CPME (10 mL), *n*-BuOH (3.5 equiv, 104 µL) 60 °C and 200% CAL-B (217 mg). The reaction was run for 3 days resulting in honey-like liquid 85 mg (90%), α:β 91:9; TLC – PE:EtOAc 1:1, R_f = 0.48; column chromatography eluent system PE:EtOAc 4:1 → 3:2;
α-^{1}H NMR (400 MHz, $CDCl_3$) δ 5.36 (dd, *J* = 3.4, 10.1 Hz, 1H, H-3), 5.27 (dd, *J* = 1.8, 3.4 Hz, 1H, H-2), 5.16 (br.s, 1H, H-1), 5.07 (t, *J* = 9.9 Hz, 1H, H-4), 4.12 (ddd, *J* = 6.2, 12.5, 9.8 Hz, 1H, H-5), 3.18 (s, 1H, OH-1), 2.15 (s, 3H), 2.05 (s, 3H), 1.99 (s, 3H), 1.22 (d, *J* = 6.4 Hz, 3H);
β-^{1}H NMR (400 MHz, $CDCl_3$) δ 5.36-5.40 (m, 1H, H-3), 5.00-5.04 (m, 2H, H-2,4), 4.94 (d, *J* = 7.5 Hz, 1H, H-1), 3.57 (ddd, *J* = 6.1, 9.8, 12.2 Hz, 1H, H-5), 3.48 (s, 1H, OH-1), 2.21 (s, 3H), 2.05 (s, 3H), 1.99 (s, 3H), 1.27 (d, *J* = 6.2 Hz, 3H);
NMR matches with previously reported values.[25]

1,2,3,4-tetra-*O*-benzoyl-D-glucopyranoside (**36**)

According to general procedure with 1,2,3,4,6-penta-*O*-benzoyl-D-glucopyranoside **35** (0.143 mmol, 100 mg), CPME (10 mL), *n*-BuOH (3.5 equiv, 46 µL), 90 °C and 200% CAL-B (200 mg). The reaction was run for 6 days resulting in white solid 21 mg (24%), α:β 88:12. TLC – PE:EtOAc 1:2, R_f = 0.45; column chromatography eluent system PE:EtOAc 17:3 → 7:3;
α-^{1}H NMR (400 MHz, $CDCl_3$) δ 8.14-8.18 (m, 2H), 7.97-8.02 (m, 2H), 7.85-7.92 (m, 4H), 7.63-7.68 (m, 1H), 7.52-7.58 (m, 3H), 7.38-7.49 (m, 4H), 7.27-7.34 (m, 4H), 6.85 (d, *J* = 3.7 Hz, 1H, H-1), 6.36 (t, *J* = 10.0 Hz, 1H, H-3), 5.67 (t, *J* = 10.0 Hz, 1H, H-4), 5.65 (dd, *J* = 3.7, 10.2 Hz, 1H. H-2), 4.26 (ddd, *J* = 2.1, 3.4, 10.2 Hz, 1H, H-5), 3.81-3.89 (m, 1H, H-6a/6b), 3.69-3.78 (m, 1H, H-6a/6b).
β-^{1}H NMR (400 MHz, $CDCl_3$) δ 7.95-8.06 (m, 4H), 7.85-7.92 (m, 4H), 7.52-7.58 (m, 3H), 7.38-7.49 (m, 5H), 7.27-7.34 (m, 4H), 6.25 (d, *J* = 8.3 Hz, 1H, H-1), 6.08 (t, *J* = 9.7 Hz, 1H, H-3), 5.84 (dd, *J* = 8.3, 9.6 Hz, 1H, H-2), 5.59-5.66 (m, 1H, H-4), 4.02 (ddd, *J* = 2.2, 4.0, 10.0 Hz, 1H, H-5), 3.81-3.89 (m, 1H, H-6a/6b), 3.69-3.78 (m, 1H, H-6a/6b).
NMR matches with previously reported values.[26]

1,2,3,4-tetra-*O*-butyryl-α-D-glucopyranoside (40) and 2,3,4,6-tetra-*O*-butyryl-D-glucopyranose (**41**)

According to general procedure with 1,2,3,4,6-penta-*O*-butyryl-D-glucopyranoside **39** (0.188 mmol, 100 mg) α:β 67:33, MTBE (10 mL), *n*-BuOH (3.5 equiv, 60 µL), 45 °C and 100% CAL-B (100

mg). Reaction was run for 6 h and resulted in 69 mg (80%) of honey-like oil composed of 68% **40** and 32% **41** (α:β 74:26). TLC – PE:EtOAc 3:1, R_f = 0.24; column chromatography eluent system PE:EtOAc 9:1 → 7:3; The NMR of **41** matches with previously reported values[27].

40- ^{1}H NMR (400 MHz, $CDCl_3$) δ 6.36 (d, *J* = 3.7 Hz, 1H, H-1), 5.56 (t, *J* = 9.9 Hz, 1H, H-3), 5.04-5.15 (m, 2H, H-2,4), 3.89 (ddd, *J* = 2.2, 4.0, 10.2 Hz, 1H, H-5), 3.69 (ddd, *J* = 1.9, 8.4, 12.8 Hz, 1H, H-6a/6b), 3.55 (td, *J* = 4.8, 12.6 Hz, 1H, H-6a/6b), 2.17-2.43 (m, 8H), 1.51-1.74 (m, 8H), 0.84-1.02 (m, 12H); ^{13}C{^{1}H} NMR (101 MHz, $CDCl_3$) δ 173.1, 172.8, 172.3, 171.7, 89.0 (C-1), 72.3 (C-5), 69.5 (C-2), 69.3 (C-3), 68.2 (C-4), 61.0 (C-6), 36.1, 36.1, 36.0, 35.8, 18.5, 18.4, 18.4, 18.3, 13.7, 13.7, 13.6, 13.6;

41-α-^{1}H NMR (400 MHz, $CDCl_3$) δ 5.56 (t, *J* = 9.9 Hz, 1H, H-3), 5.45 (br.t, *J* = 3.2 Hz, 1H, H-1), 5.04-5.16 (m, 1H, H-4), 4.90 (dd, *J* = 3.9, 10.1 Hz, 1H, H-2), 4.25 (ddd, *J* = 2.2, 3.9, 10.2 Hz, 1H, H-5), 4.20 (dd, *J* = 4.6, 11.8 Hz, 1H, H-6a/6b), 4.13 (dd, *J* = 2.2, 12.3 Hz, 1H, H-6a/6b), 3.26 (s, 1H, OH-1), 2.16-2.43 (m, *J* = 5.7 Hz, 8H), 1.49-1.75 (m, *J* = 4.4 Hz, 8H), 0.84-1.01 (m, *J* = 5.2 Hz, 12H);

41-β-^{1}H NMR (400 MHz, $CDCl_3$) δ 5.28 (t, *J* = 9.6 Hz, 1H, H-3), 5.04-5.16 (m, 1H, H-4), 4.88 (t, *J* = 9.1 Hz, 1H, H-2), 4.72 (br.t, *J* = 7.9 Hz, 1H, H-1), 4.11-4.23 (m, 2H, H-6a,6b), 3.74 (ddd, *J* = 2.3, 4.8, 10.3 Hz, 1H, H-5), 3.26 (s, 1H, OH-1), 2.16-2.43 (m, *J* = 5.7 Hz, 8H), 1.49-1.75 (m, *J* = 4.4 Hz, 8H), 0.84-1.01 (m, *J* = 5.2 Hz, 12H);

HRMS (AJS-ESI) of **40** and **41** mixture: $[M+Na]^+$ for $C_{22}H_{36}O_{10}Na^+$ 483.2201 found 483.2201.

1,2,3-tri-*O*-acetyl-α-D-glucopyranoside (**43**)

According to general procedure with α-Glc pentaacetate **42** (0.256 mmol, 100 mg), MTBE (10 mL), *n*-BuOH (3.5 equiv, 82 µL), 45 °C and 50% CAL-B (100 mg). Reaction was run for 23 h and resulted in 64 mg (82%) of white solid. TLC – PE:EtOAc 1:2, R_f = 0.19;

^{1}H NMR (400 MHz, $CDCl_3$) δ 6.28 (d, *J* = 3.7 Hz, 1H, H-1), 5.32 (t, *J* = 9.5 Hz, 1H, H-3), 5.00 (dd, *J* = 3.7, 10.2 Hz, 1H, H-2), 3.78-3.89 (m, 4H, H-4,5,6a,6b), 3.22 (s, 1H, OH-4/6), 2.40 (s, 1H, OH-4/6), 2.16 (s, 3H), 2.11 (s, 3H), 2.02 (s, 3H);

NMR matches with previously reported values[19].

Diphenyl 2,4,6-tri-*O*-α-D-galactopyranosyl phosphate (S1), diphenyl 2,3,6-tri-*O*-α-D-galactopyranosyl phosphate (S2) and **diphenyl 2,3,4-tri-*O*-α-D-galactopyranosyl phosphate (S3)**

According to general procedure with diphenyl 2,3,4,6-tetra-*O*-acetyl-α-D-galactopyranosyl phosphate **22** (60 mg), MTBE (6 mL), *n*-BuOH (3.5 equiv, 33 µL), 60 °C and 200% CAL-B (120 mg) resulted in mixtures of unstable products (Table S1), the products **S1**-**S3** were isolated in one fraction resulting in sticky transparent syrup 45 mg (80%); TLC – PE:EtOAc 1:1, R_f = 0.30; column chromatography eluent system PE(with 0.1% Et_3N):EtOAc 7:3 → 3:2;

S1 – ^{1}H NMR (400 MHz, $CDCl_3$) δ 7.40 – 7.31 (m, 4H), 7.28 – 7.16 (m, 6H), 6.11 (dd, *J* = 6.0, 3.3 Hz, 1H, H-1), 5.44 (dd, *J* = 3.5, 1.3 Hz, 1H, H-4),5.09 (dt, *J* = 10.4, 3.1 Hz, 1H, H-2), 4.33 (t, *J* = 6.5 Hz, 1H, C-5), 4.24 (dd, *J* = 10.4, 3.4 Hz, 1H, C-3), 4.12 (dd, *J* = 11.4, 6.8 Hz, 1H, H-6a/b), 3.93 (dd, *J* = 11.4, 6.8 Hz, 1H, H-6a/b), 2.16 (s, 3H), 1.96 (s, 3H), 1.92 (s, 3H); ^{13}C{^{1}H} NMR (101 MHz, $CDCl_3$) δ 171.0, 171.0, 170.5, 130.1, 130.0, 125.8, 120.5, 120.3, 120.2, 120.1, 96.0 (d, *J* = 5.99 Hz, C-1), 70.1 (d, *J* = 4.29 Hz, C-2), 70.0 (C-4), 69.3 (C-5), 66.4 (C-3), 61.7 (C-6), 20.8, 20.7, 20.5.

S2 - ^{1}H NMR (400 MHz, $CDCl_3$) δ 7.34 (td, *J* = 7.8, 3.0 Hz, 4H), 7.25 – 7.11 (m, 6H), 6.09 (dd, *J* = 6.3, 3.3 Hz, 1H, H-1), 5.37 (dt, *J* = 11.0, 3.0 Hz, 1H, H-2), 5.29 (dd, *J* = 10.8, 2.9 Hz, 1H, H-3), 4.28 (dd, *J* = 11.2, 5.8 Hz, 1H, H6a/b), 4.19 (td, 1H, H-5), 4.13 (dd, *J* = 5.2, 3.8 Hz, 1H, H-4), 4.06 (dd, *J* = 11.3, 6.3 Hz, 1H, H-6a/b), 2.09 (s, 3H), 1.93 (s, 3H), 1.84 (s, 3H); ^{13}C{^{1}H} NMR (101 MHz, $CDCl_3$) δ 171.1, 170.2, 170.2, 130.0, 129.9, 129.8, 125.8, 125.8, 120.4, 120.4, 120.3, 120.3, 120.2, 120.2, 120.2, 120.1, 96.2 (d, *J* = 5.79 Hz, C-1), 70.3 (C-5), 69.2(C-3), 67.2(C-4), 67.0 (d, *J* = 7.12 Hz, C2), 62.4 (C-6), 20.9, 20.7, 20.5.

S3 - ^{1}H NMR (400 MHz, $CDCl_3$) δ 7.39 – 7.30 (m, 4H), 7.28 – 7.15 (m, 6H), 6.10 (dd, *J* = 6.2, 3.4 Hz,

1H, H-1), 5.46 (dd, *J* = 3.2, 1.2 Hz, 1H, H-4), 5.38 (dd, *J* = 10.8, 3.2 Hz, 1H, H-3), 5.26 (dt, *J* = 10.9, 3.0 Hz, 1H, H-2), 4.16 (td, *J* = 6.4, 1.26 Hz, 1H, H-5), 3.52 (dd, *J* = 11.7, 6.6 Hz, 1H, H-6a/b), 3.42 (dd, *J* = 11.7, 6.2 Hz, 1H, H-6a/b), 2.14 (s, 3H), 2.01 (s, 3H), 1.86 (s, 3H); $^{13}C\{^{1}H\}$ NMR (101 MHz, $CDCl_3$) δ 170.9, 170.2, 169.9, 130.1, 130.0, 125.8, 120.5, 120.4, 120.2, 120.2, 120.1, 95.9 (dd, *J* = 5.46 Hz, C-1), 71.6 (C-5), 68.2 (C-4), 67.3 (d, *J* = 7.22 Hz, C-2), 67.0 (C-3), 60.5 (C-6), 20.9, 20.8, 20.7;
HRMS (AJS-ESI): $[M+Na]^+$ for $C_{24}H_{27}O_{12}PNa^+$ 561.1132 found 561.1132.

NMR Spectra

2,3,4,6-tetra-*O*-acetyl-D-glucopyranose (**2**) α:β 7:3 ^{1}H NMR (400 MHz) in $CDCl_3$

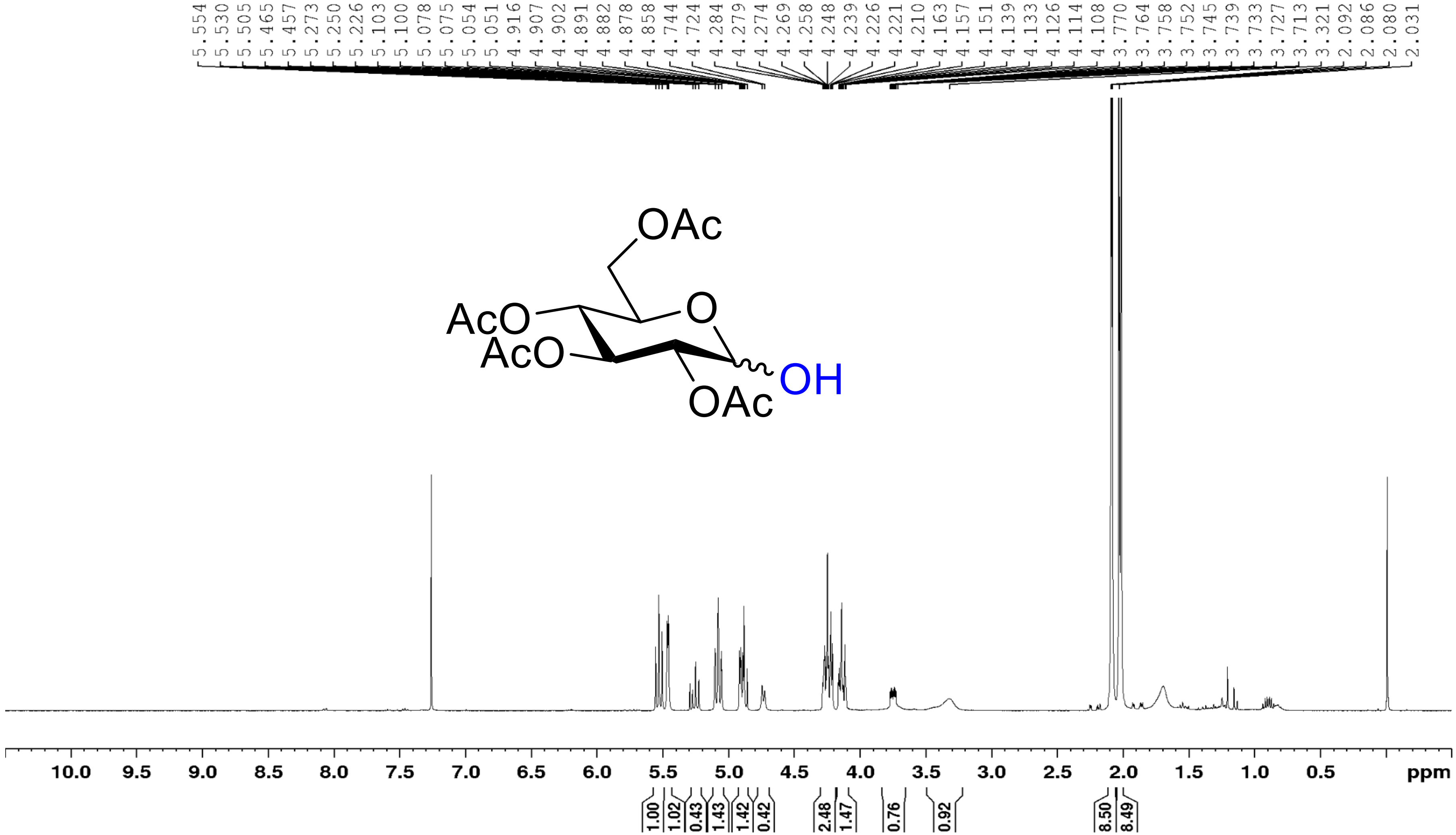

2,3,4,6-tetra-*O*-acetyl-D-glucopyranose (**2**) α:β 7:3 ^1H-^1H COSY NMR (400 MHz) in $CDCl_3$

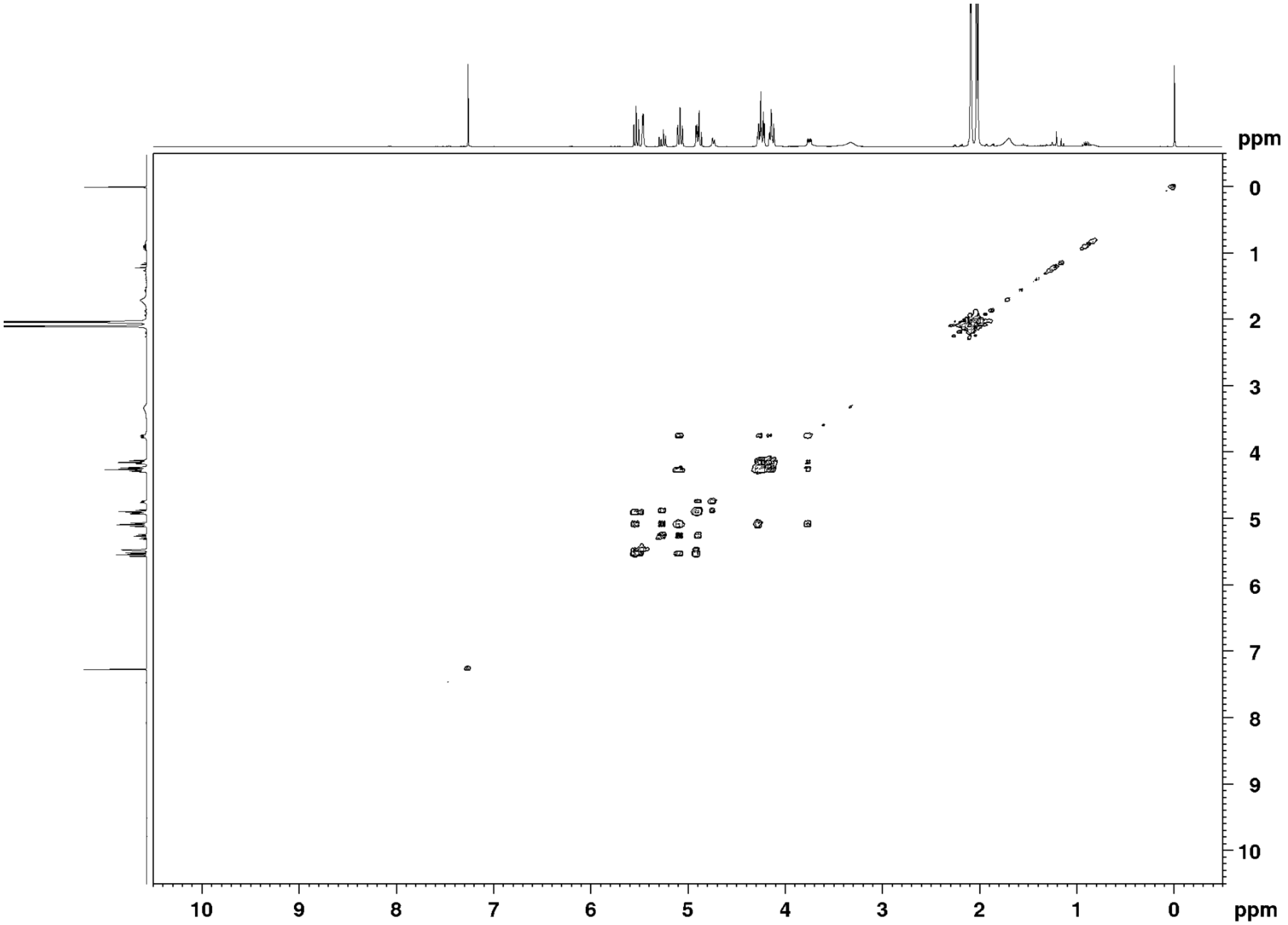

2,3,4,6-tetra-*O*-acetyl-D-glucopyranose (**2**) α:β 7:3 ^1H-$^{13}C\{^1H\}$ HSQC NMR (400 & 101 MHz) in $CDCl_3$

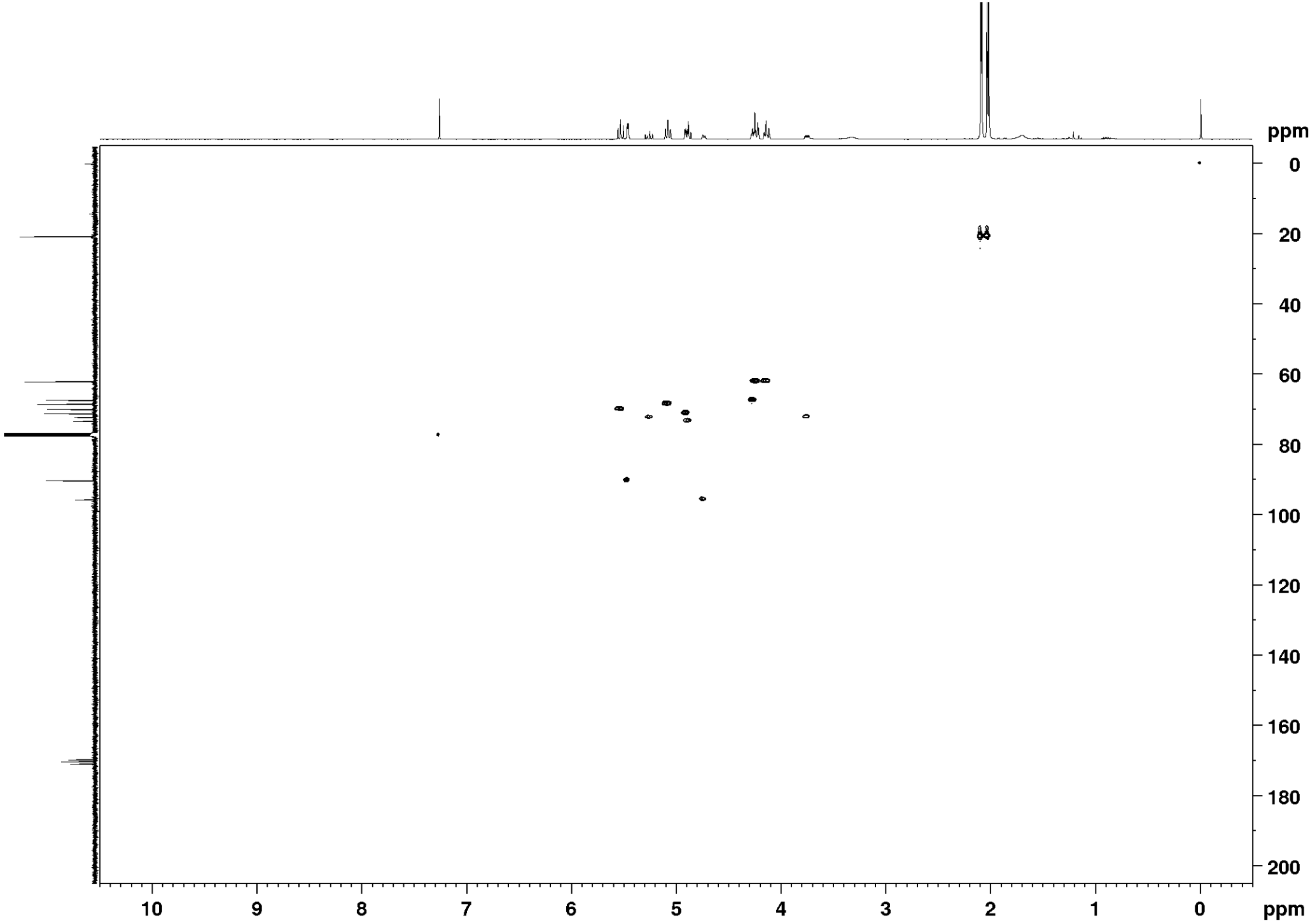

2,3,4,6-tetra-*O*-acetyl-D-glucopyranose (**2**) α:β 7:3 ^{1}H-$^{13}C\{^{1}H\}$ HMBC NMR (400 & 101 MHz) in $CDCl_3$

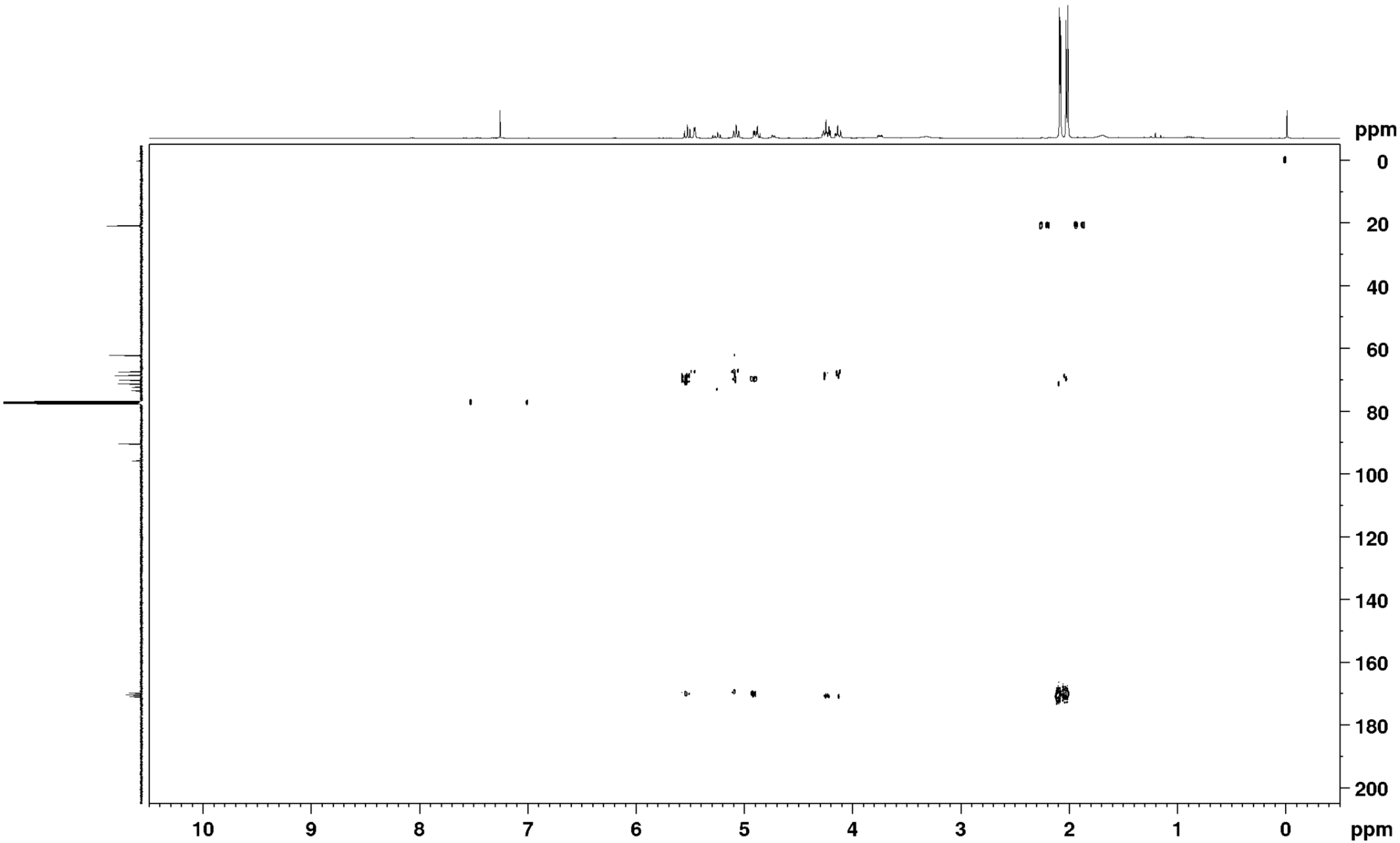

2,3,4,6-tetra-*O*-acetyl-D-glucopyranose (**2**) crude with EtOH α:β 54:46 ^{1}H NMR (400 MHz) in $CDCl_3$

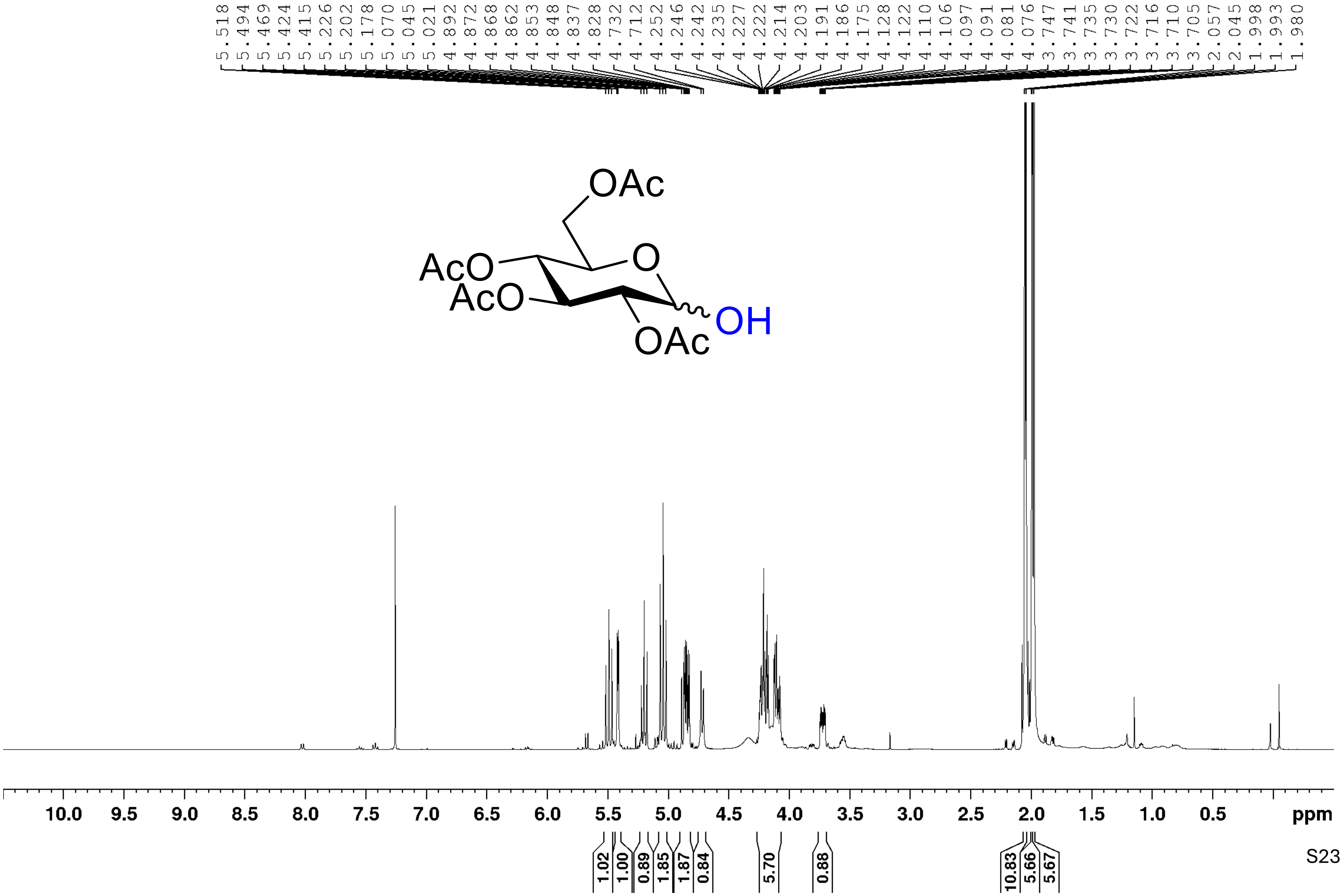

2, 3,4,6-tetra-*O*-acetyl-D-galactopyranose (**4**) α:β 7:3 ^{1}H NMR (400 MHz) in $CDCl_3$

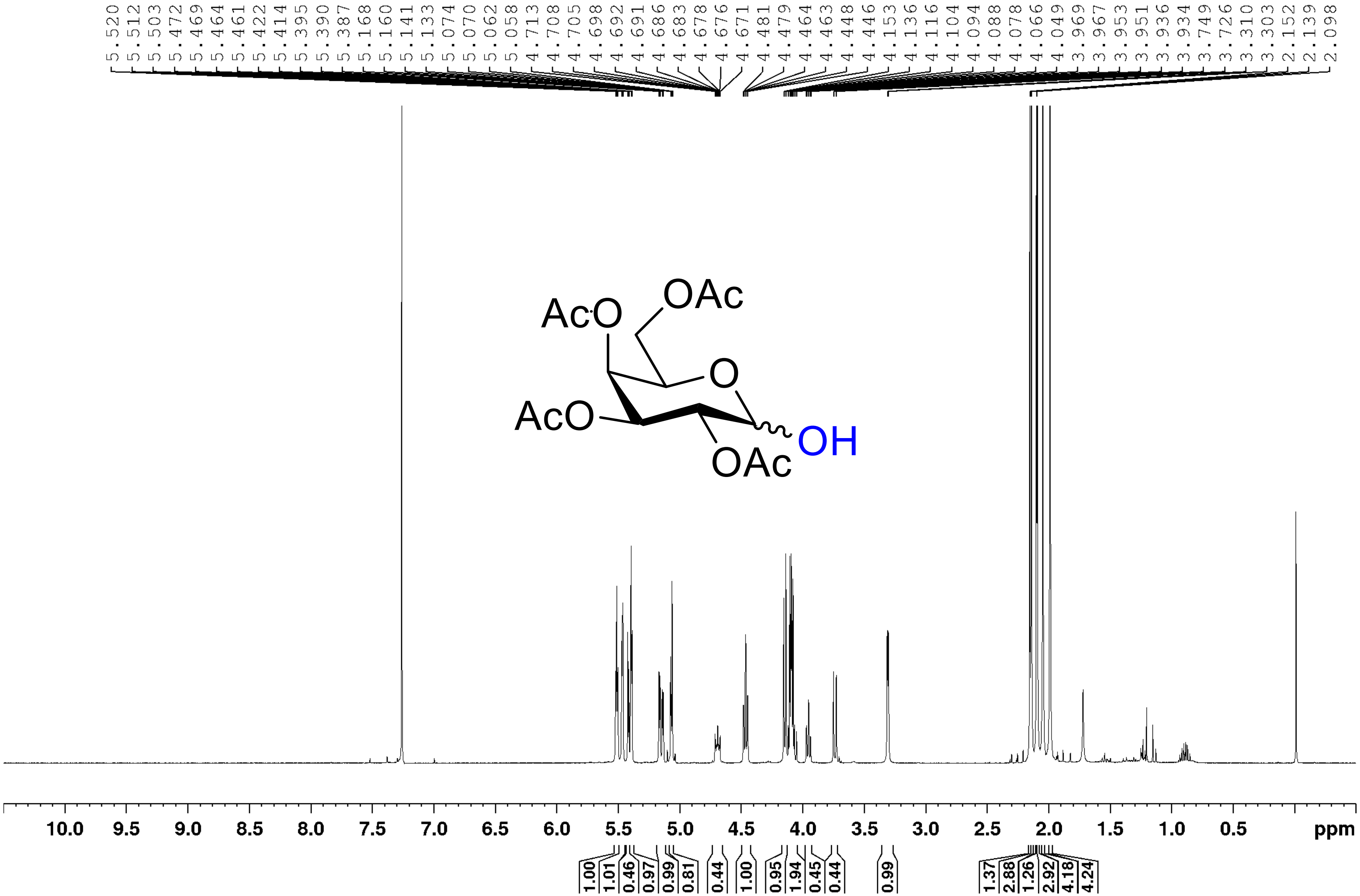

2,3,4,6-tetra-*O*-acetyl-D-galactopyranose (**4**) α:β 7:3 ^{1}H-^{1}H COSY NMR (400 MHz) in $CDCl_3$

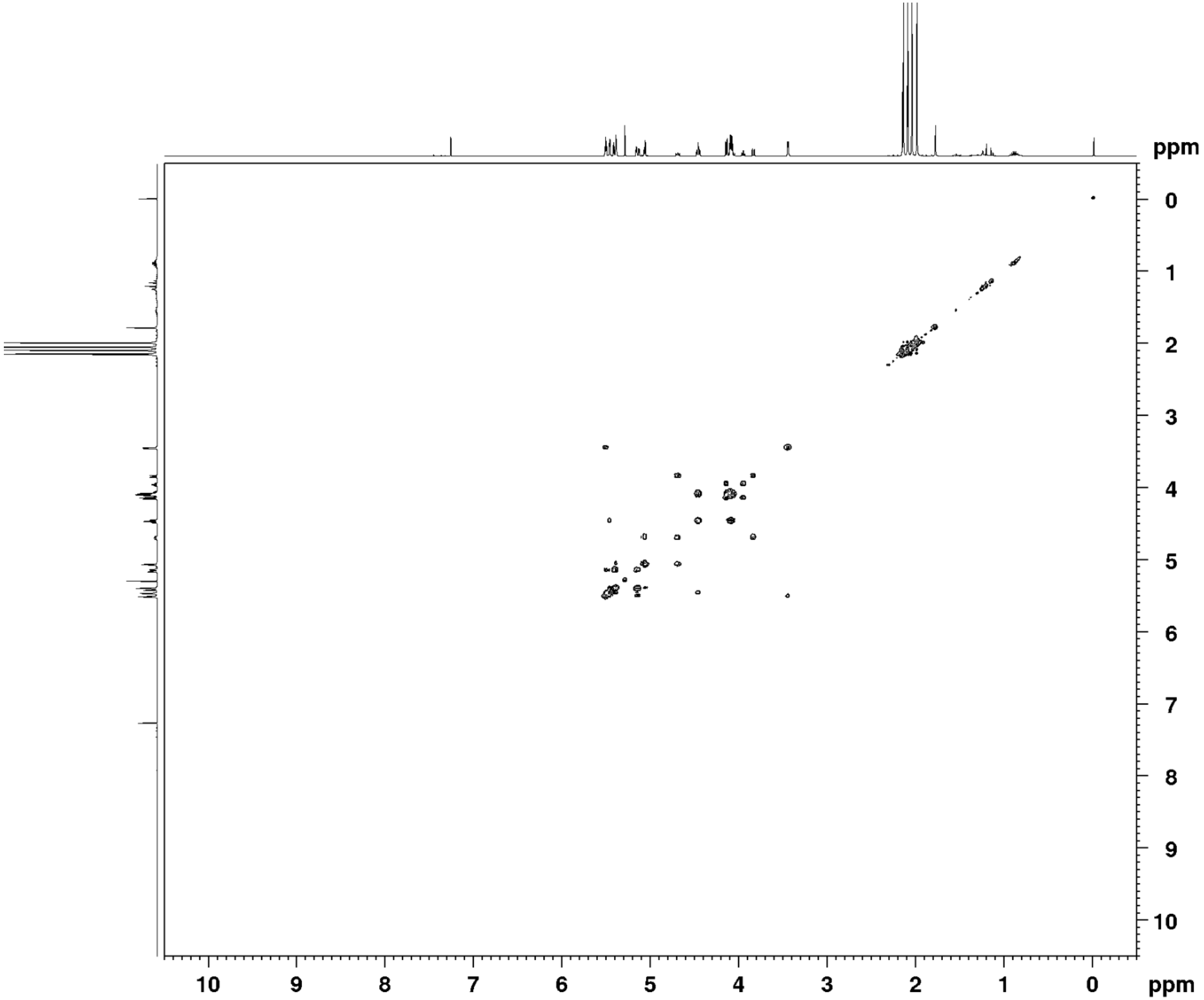

2,3,4,6-tetra-*O*-acetyl-D-galactopyranose (**4**) α:β 7:3 ^1H-$^{13}C\{^1H\}$ HSQC NMR (400 & 101 MHz) in $CDCl_3$

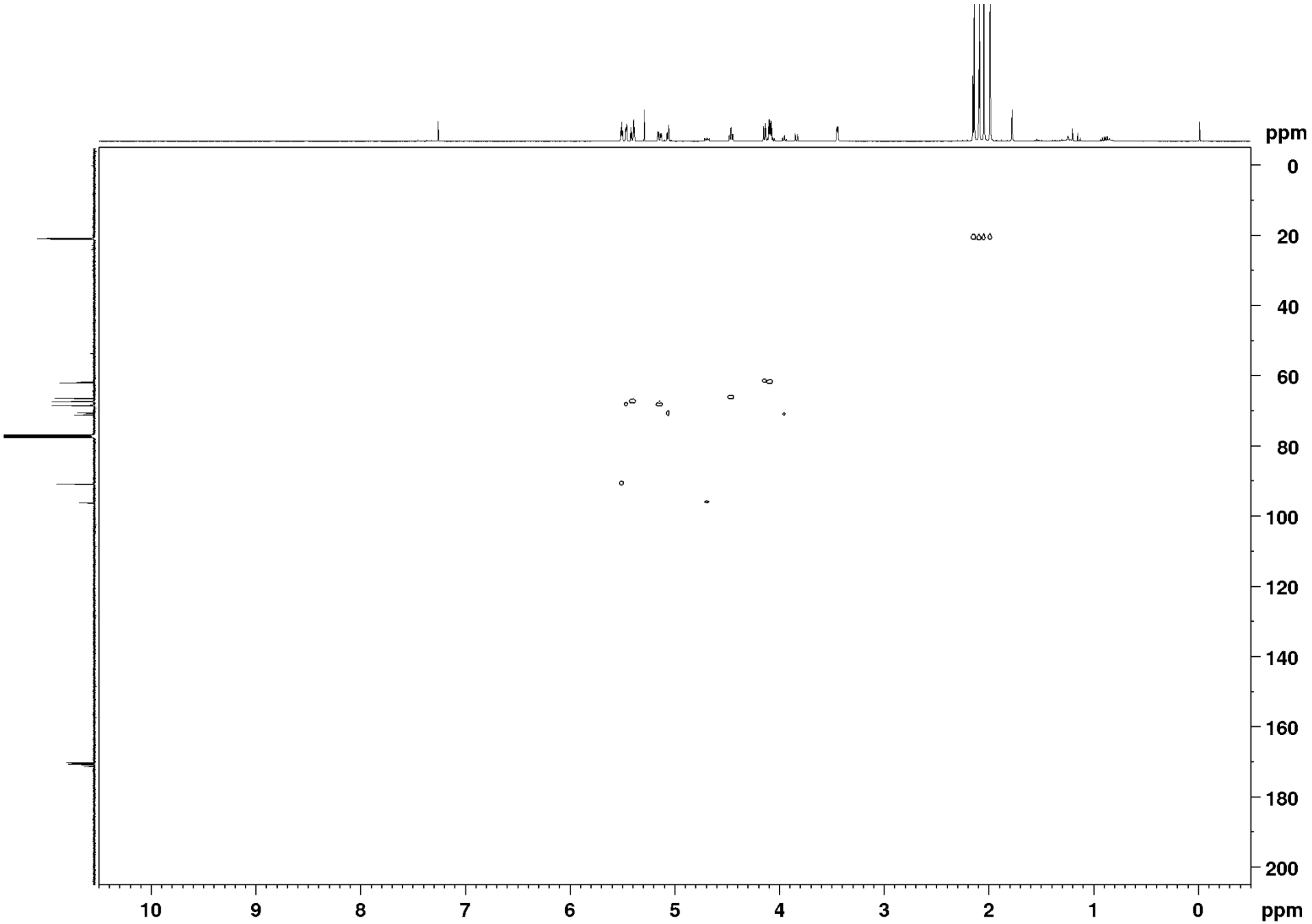

2,3,4,6-tetra-*O*-acetyl-D-galatopyranose (**4**) α:β 7:3 ^{1}H-^{13}C{^{1}H} HMBC NMR (400 & 101 MHz) in $CDCl_3$

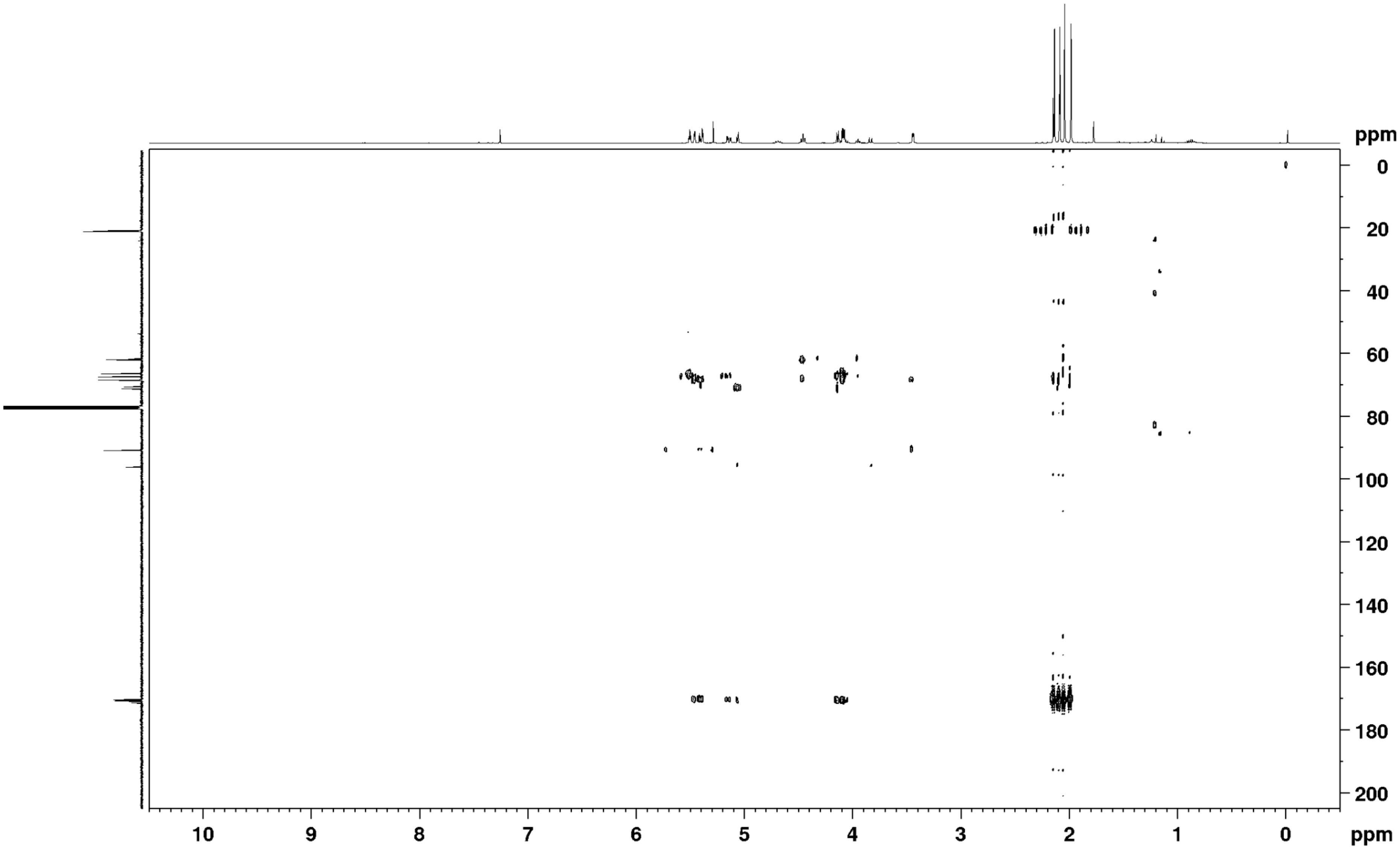

1,2,3,4-tetra-*O*-acetyl-α-D-galactopyranoside (**6**) ^{1}H NMR (400 MHz) in $CDCl_3$

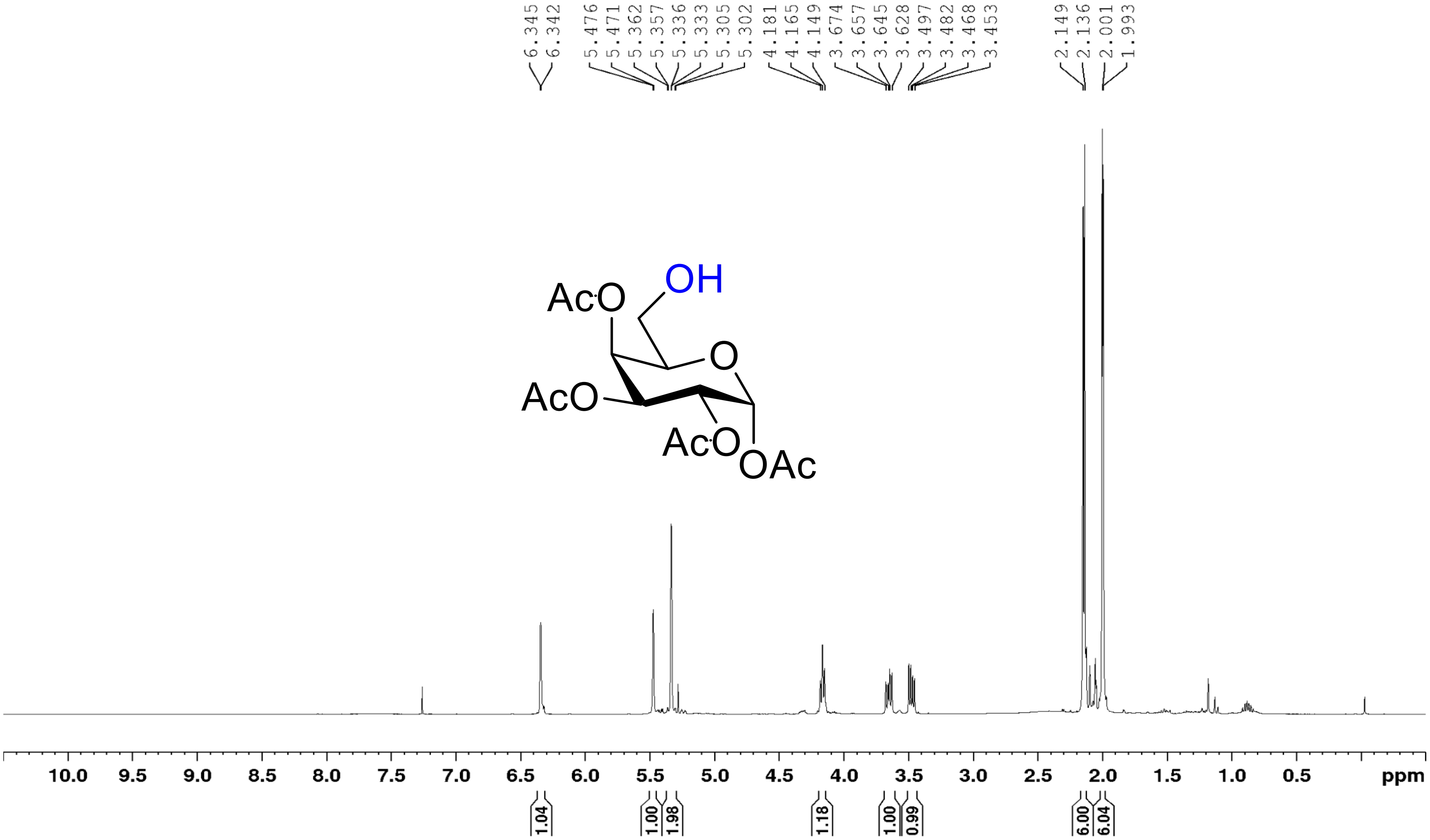

1,2,3,4-tetra-*O*-acetyl-α-D-galactopyranoside (**6**) ^{1}H-^{1}H COSY NMR (400 MHz) in $CDCl_3$

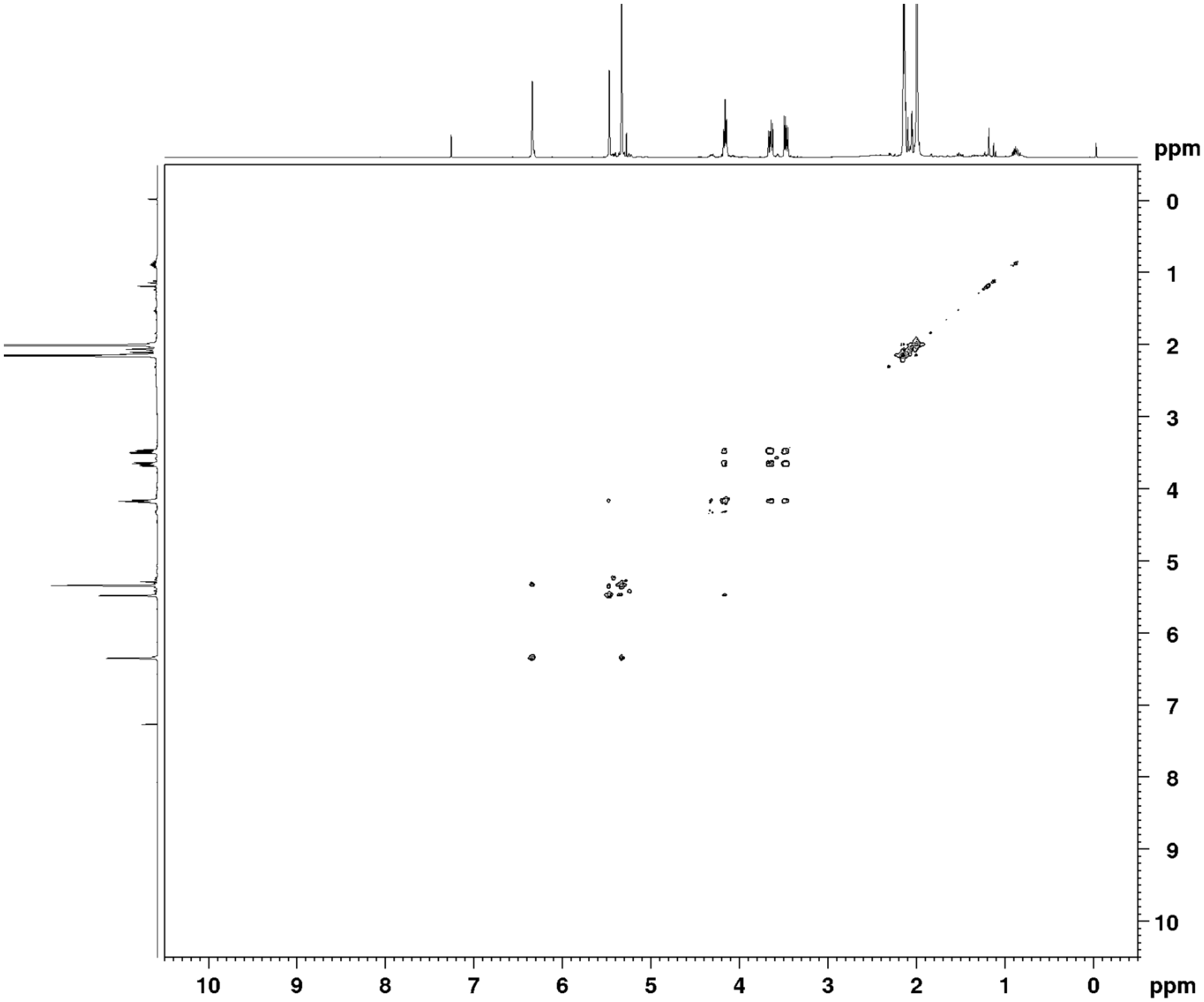

1,2,3,4-tetra-*O*-acetyl-α-D-galactopyranoside (**6**) ^{1}H-$^{13}C\{^{1}H\}$ HSQC NMR (400 & 101 MHz) in $CDCl_3$

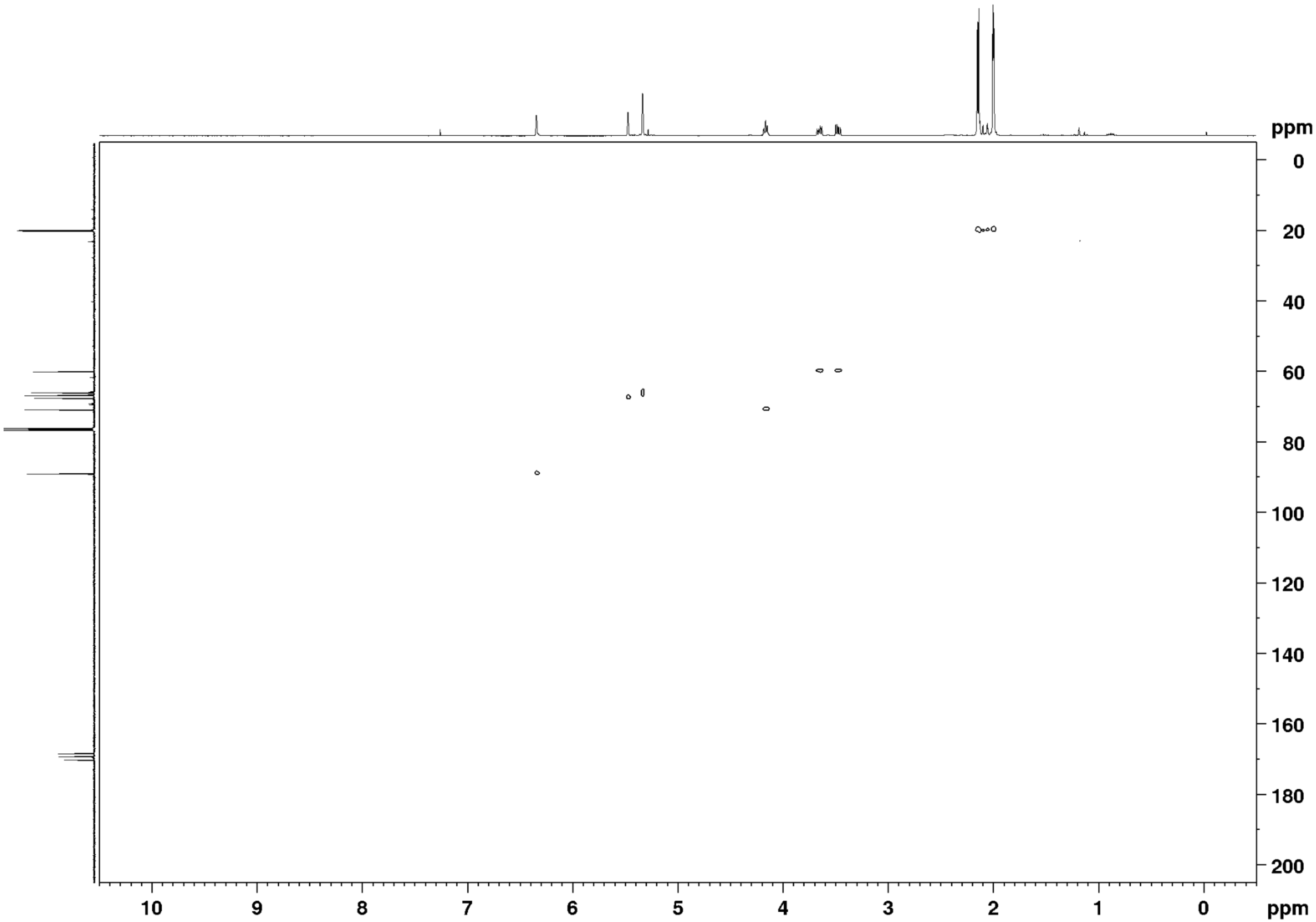

1,2,3,4-tetra-*O*-acetyl-α-D-galactopyranoside (**6**) ^{1}H-$^{13}C\{^{1}H\}$ HMBC NMR (400 & 101 MHz) in $CDCl_3$

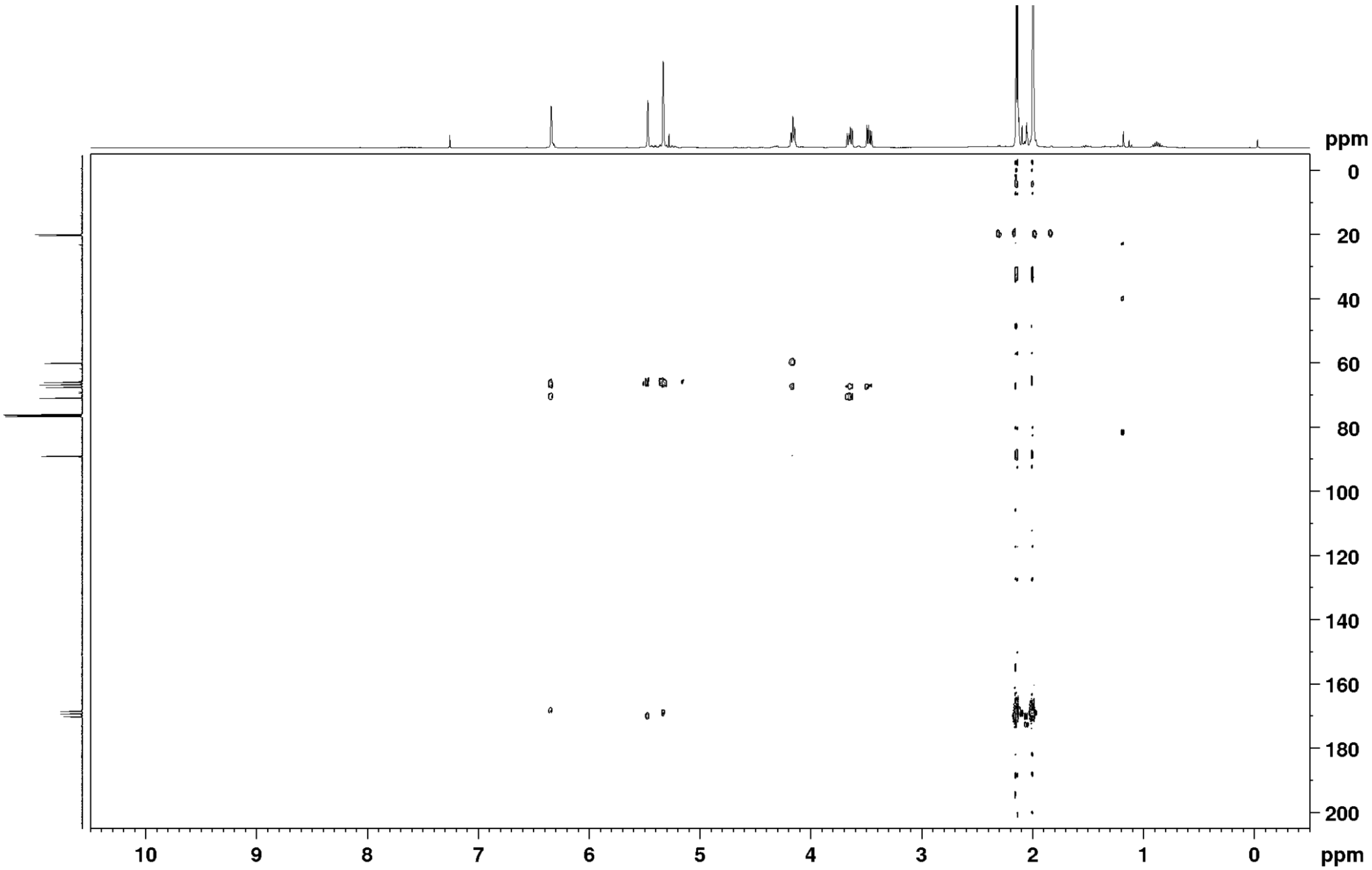

2,3,4,6-tetra-*O*-acetyl-α-D-glucopyranosyl chloride (**7**) ^{1}H NMR (400 MHz) in $CDCl_3$

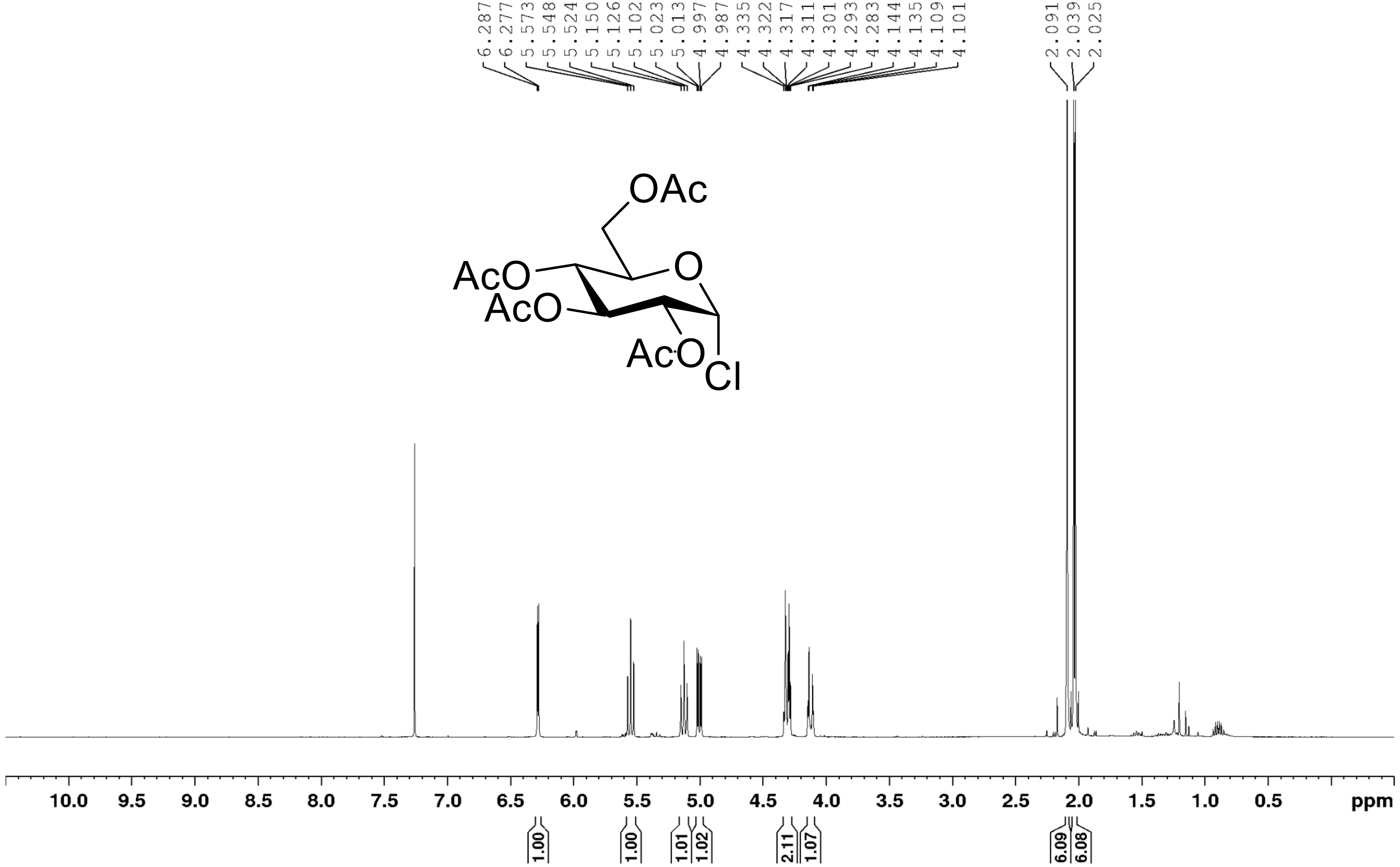

2,3-di-*O*-acetyl-α-D-glucopyranosyl chloride (**8**) ^{1}H NMR (400 MHz) in $CDCl_3$

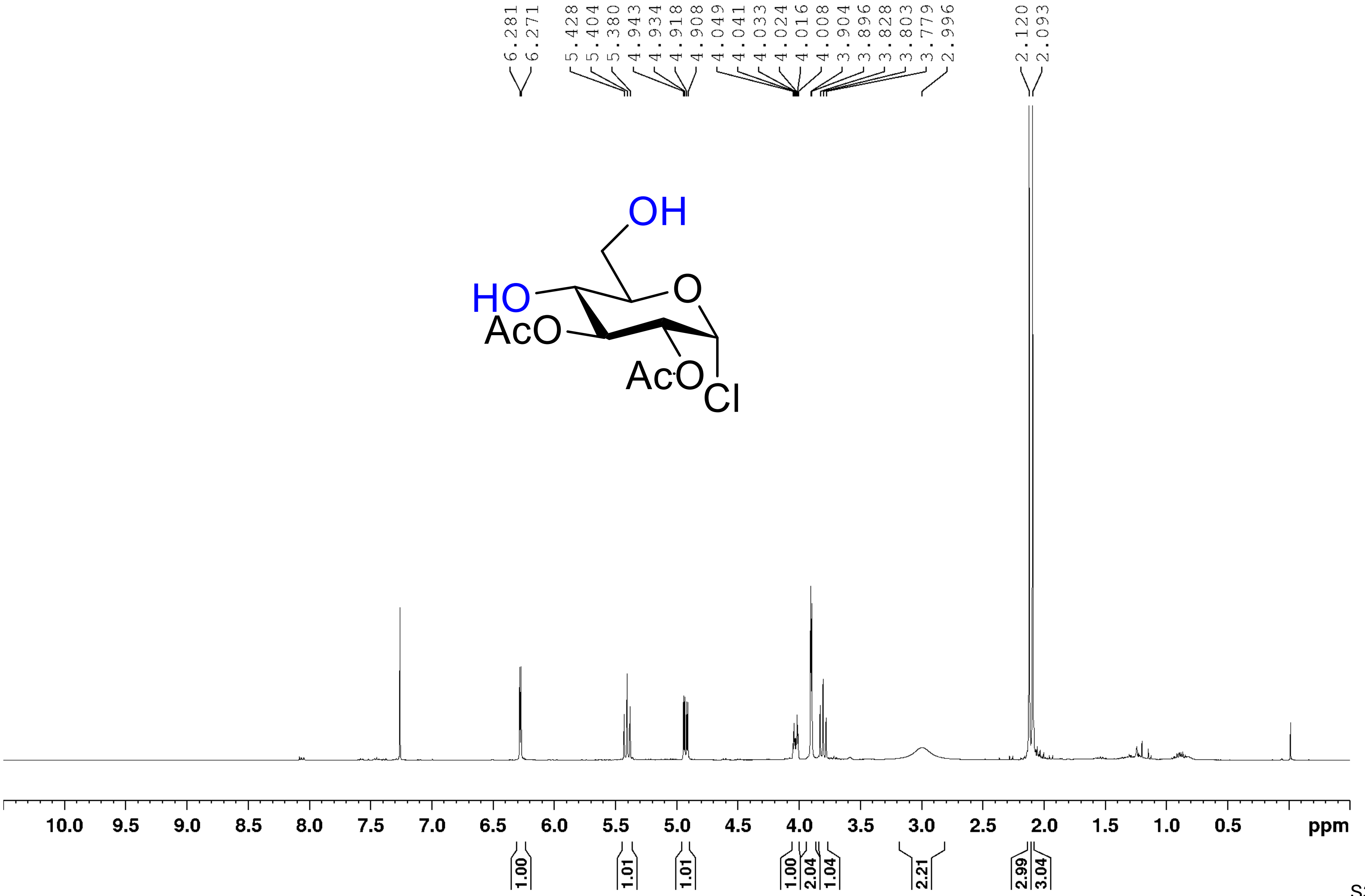

2,6-di-*O*-acetyl-α-D-glucopyranosyl chloride (8) ^{1}H-^{1}H COSY NMR (400 MHz) in $CDCl_3$

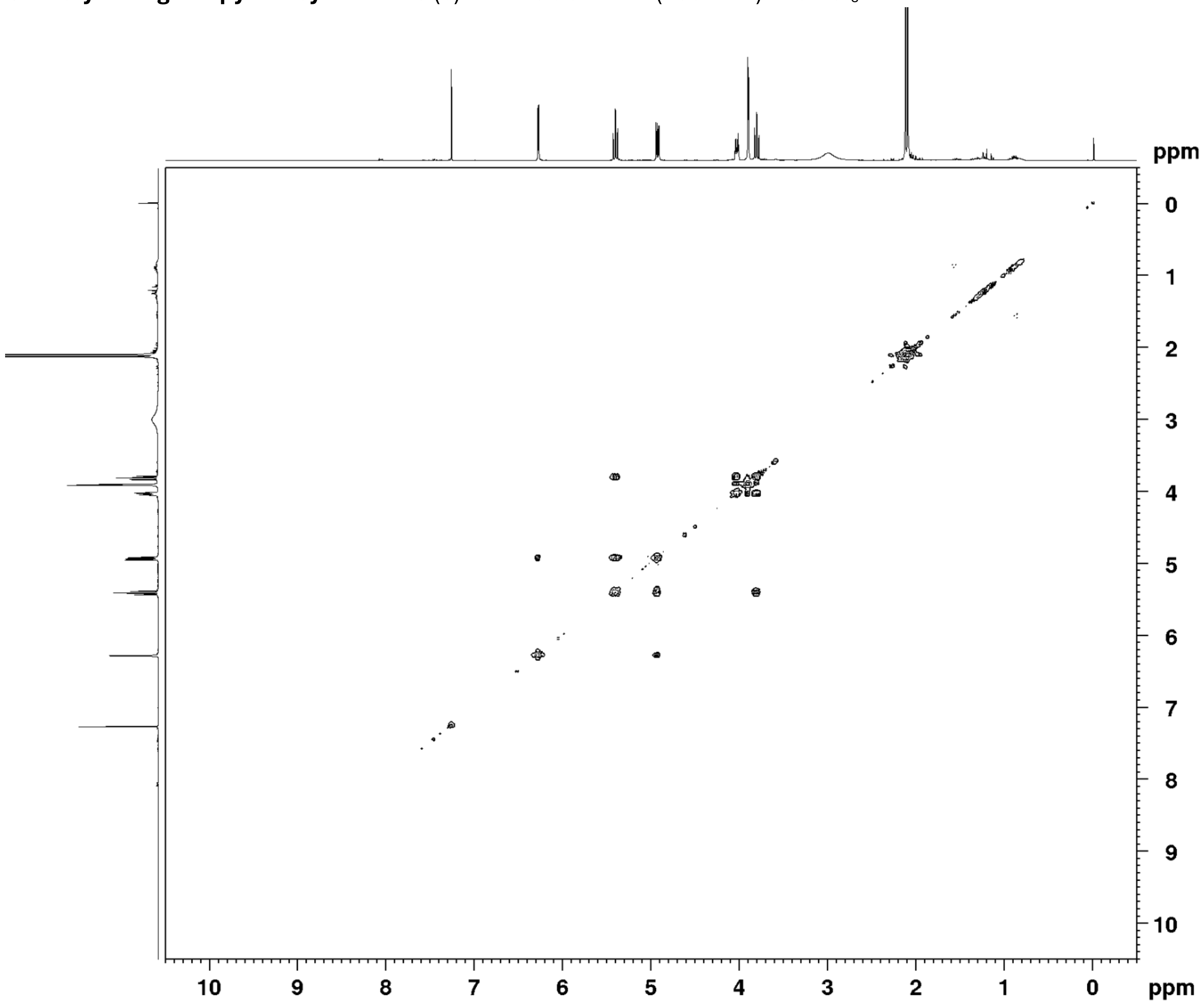

2,6-di-*O*-acetyl-α-D-glucopyranosyl chloride (**8**) ^{1}H-$^{13}C\{^{1}H\}$ HSQC NMR (400 & 101 MHz) in $CDCl_3$

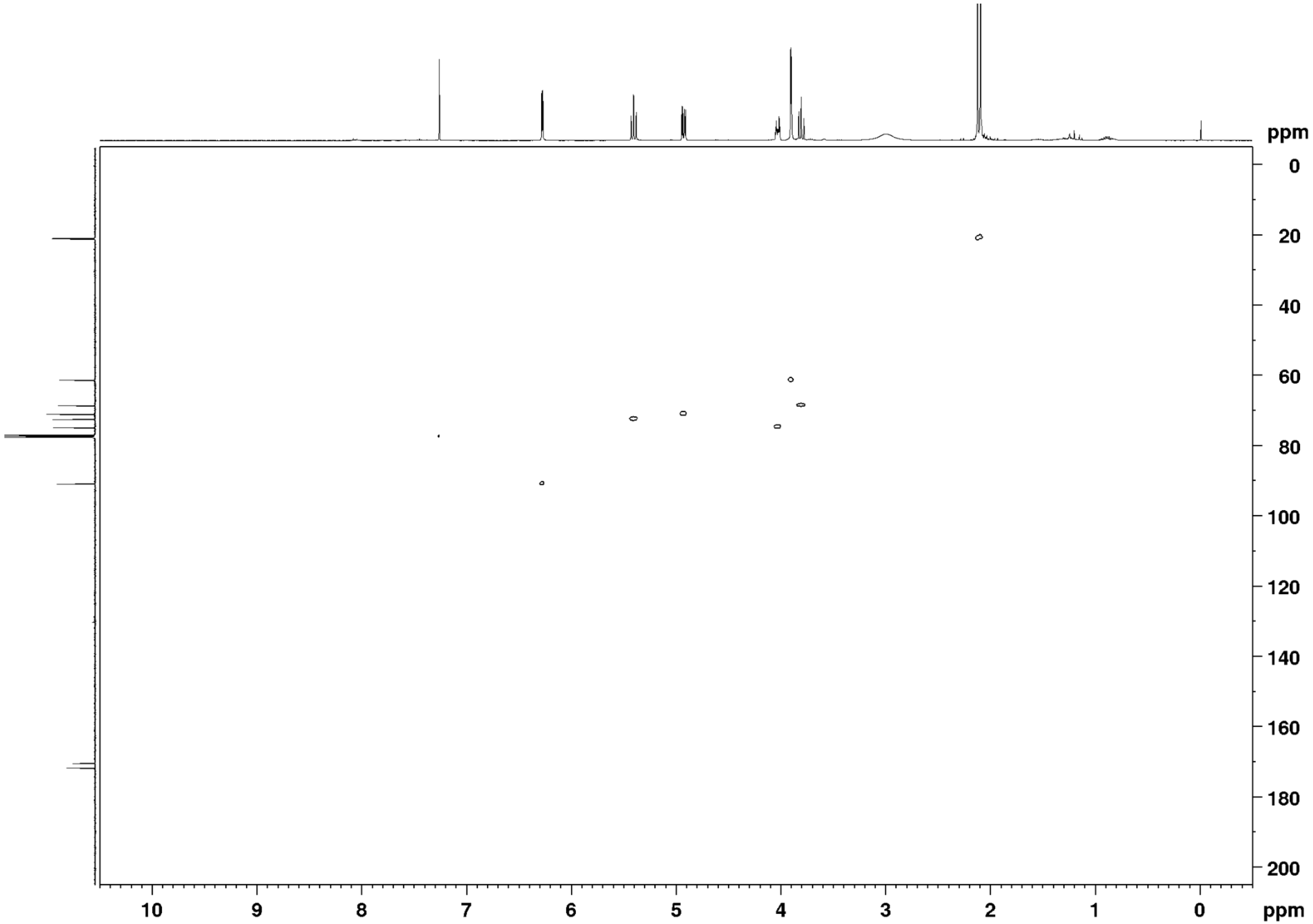

2,6-di-*O*-acetyl-α-D-glucopyranosyl chloride (**8**) ^{1}H-$^{13}C\{^{1}H\}$ HMBC NMR (400 & 101 MHz) in $CDCl_3$

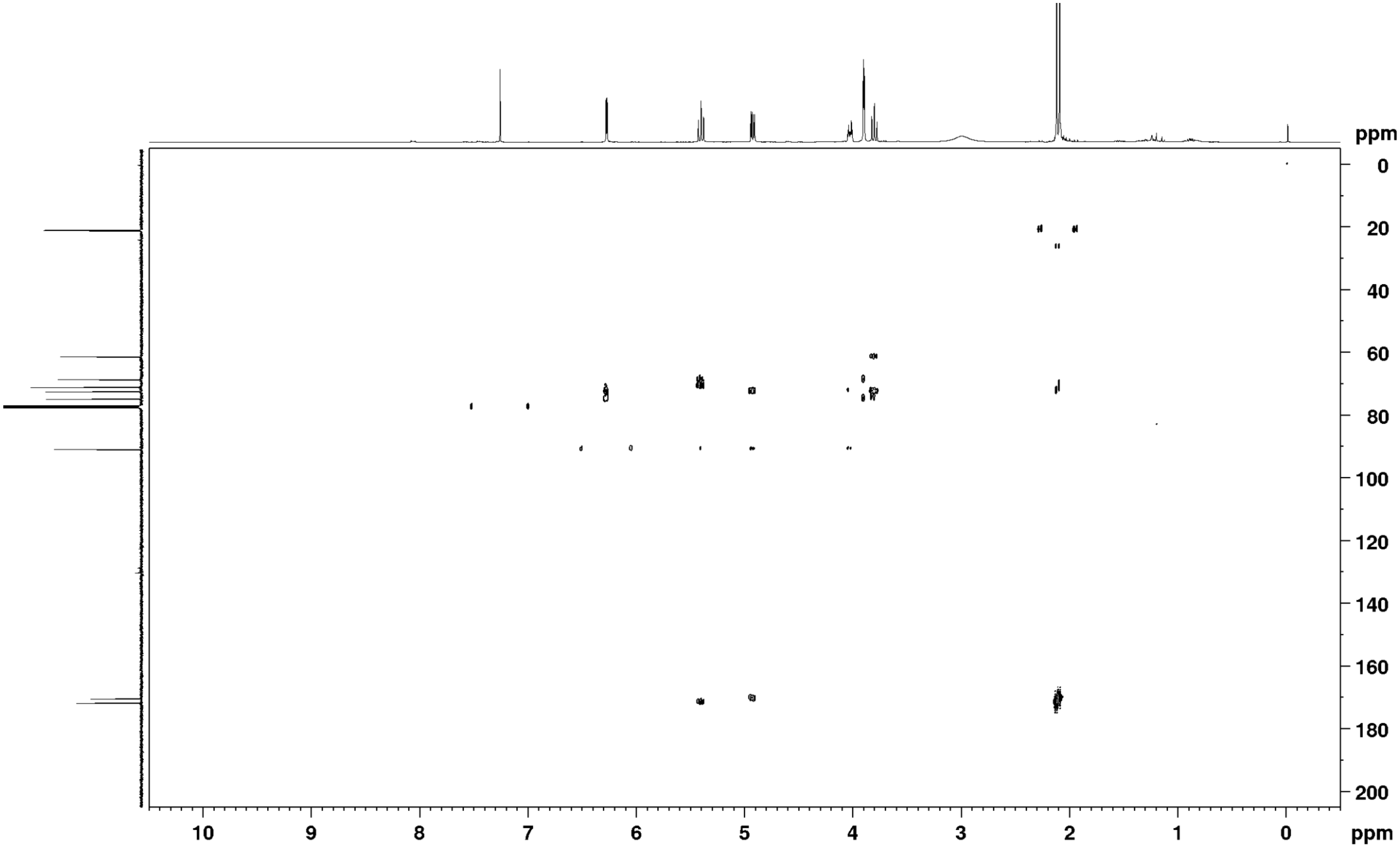

2,3-di-*O*-acetyl-α-D-glucopyranosyl chloride (8) $^{13}C\{^1H\}$ NMR (101 MHz) in $CDCl_3$

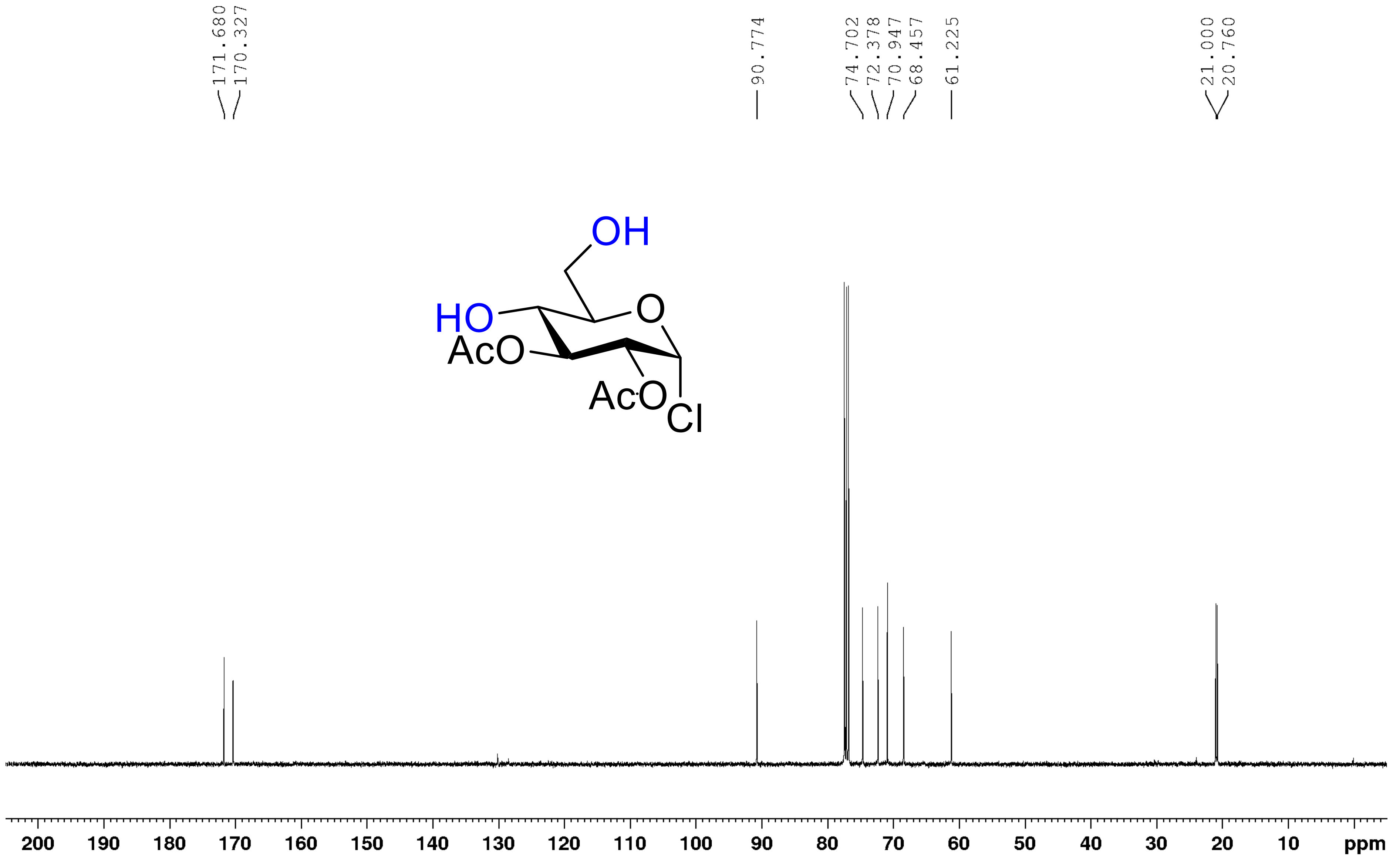

2,3,4,6-tetra-*O*-acetyl-α-D-galactopyranosyl chloride (**9**) ^{1}H NMR (400 MHz) in $CDCl_3$

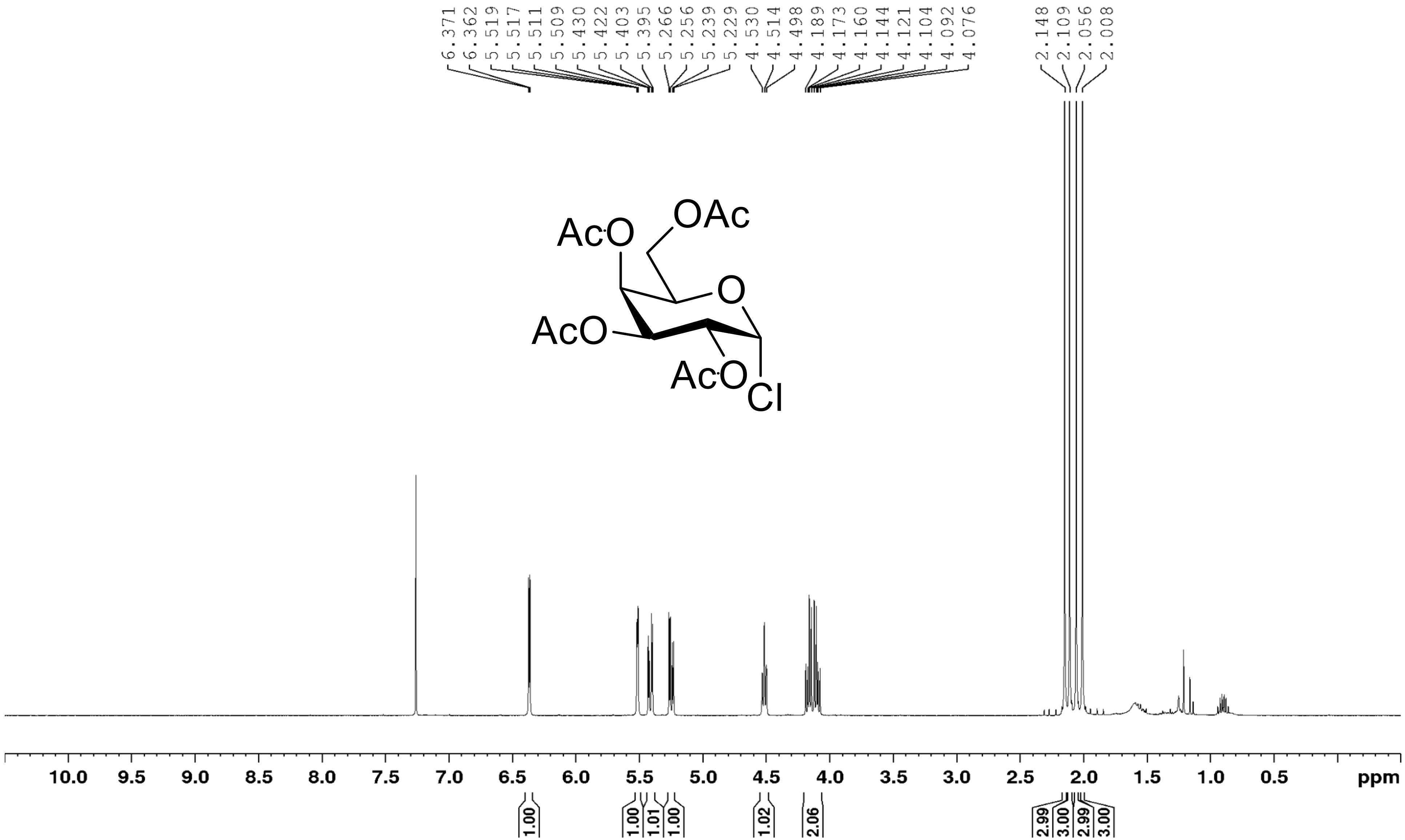

Mixture of 2,3,4-tri-*O*-acetyl-α-D-galactopyranosyl chloride (**10**) and **2,3,6-tri-*O*-acetyl-α-D-galactopyranosyl chloride** (**11**) ~1:1, **10** shown ^{1}H NMR (400 MHz) in $CDCl_3$

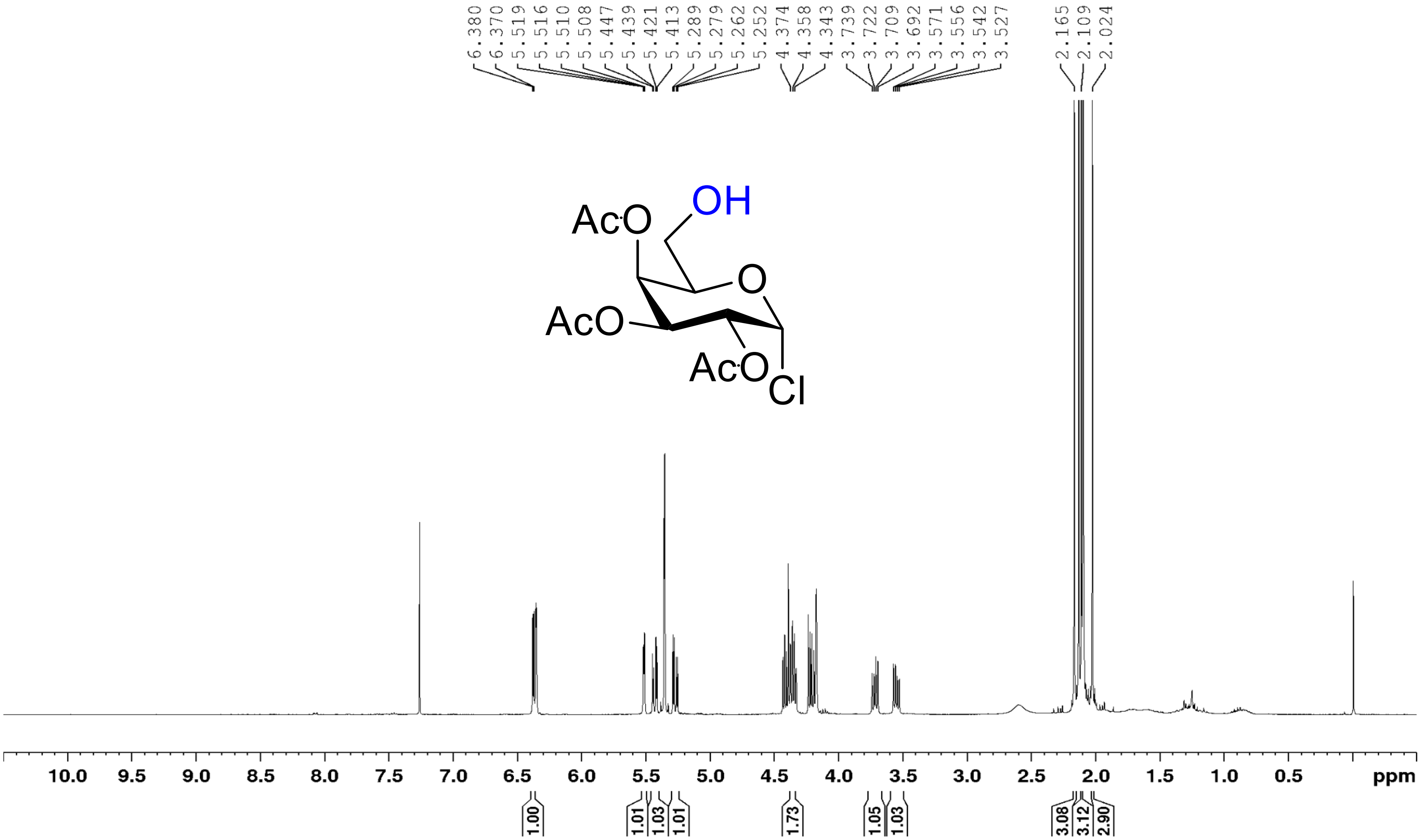

Mixture of 2,3,4-tri-*O*-acetyl-α-D-galactopyranosyl chloride (**10**) and **2,3,6-tri-*O*-acetyl-α-D-galactopyranosyl chloride** (**11**) ~1:1, **10** shown ^{1}H-^{1}H COSY NMR (400 MHz) in $CDCl_3$

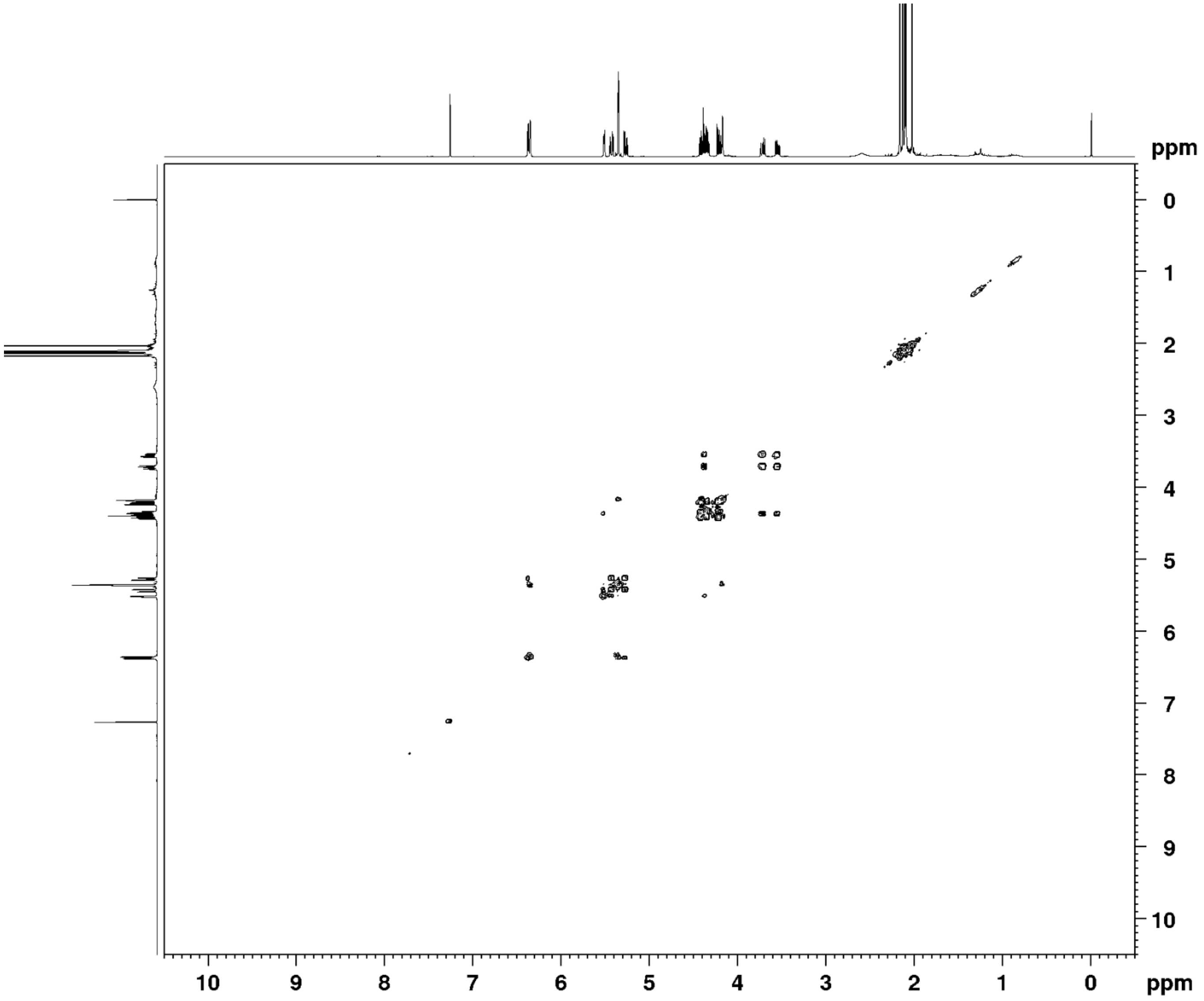

Mixture of 2,3,4-tri-*O*-acetyl-α-D-galactopyranosyl chloride (**10**) and **2,3,6-tri-*O*-acetyl-α-D-galactopyranosyl chloride** (**11**) ~1:9, **11** shown ^{1}H NMR (400 MHz) in $CDCl_3$

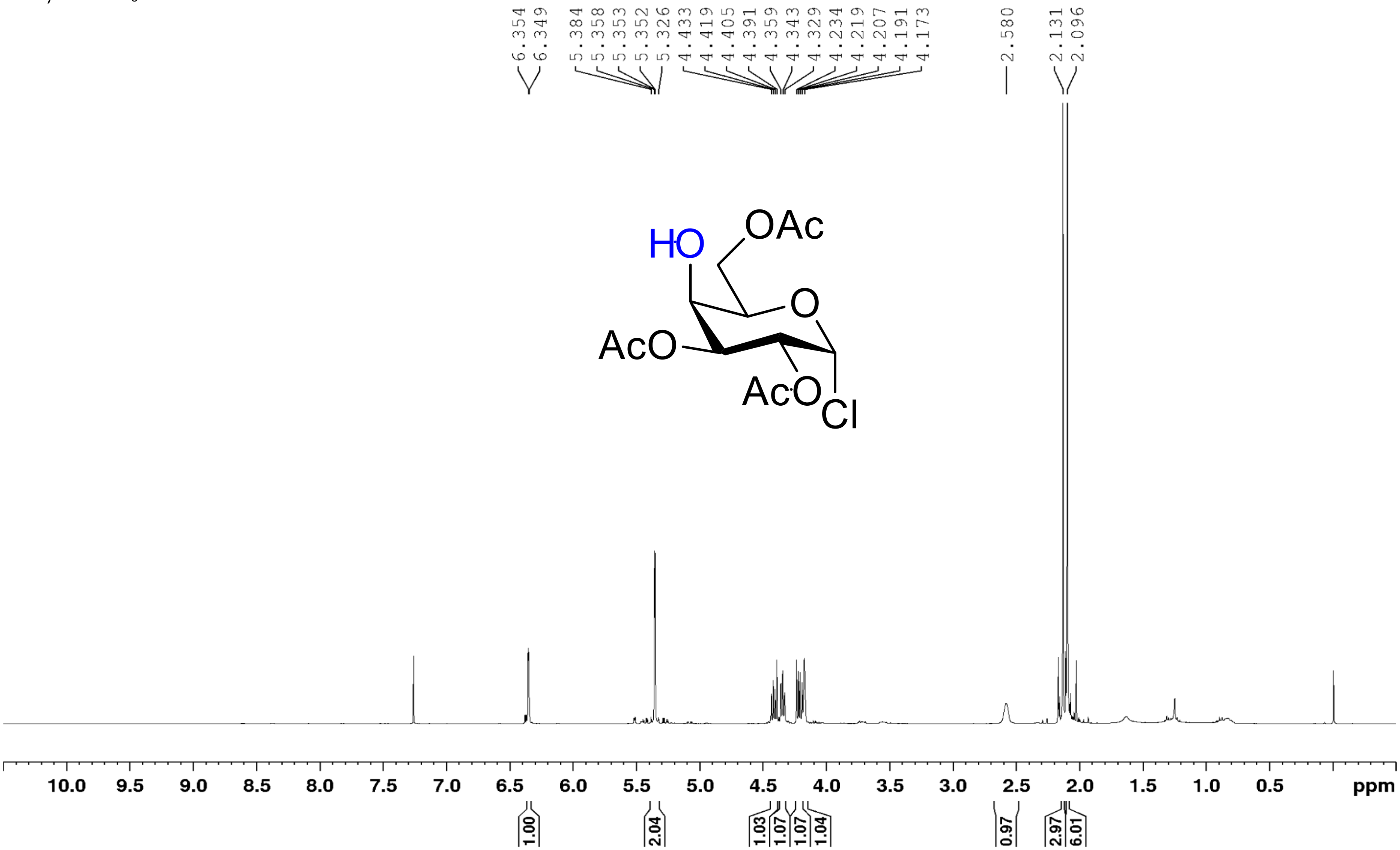

Mixture of 2,3,4-tri-*O*-acetyl-α-D-galactopyranosyl chloride (**10**) and **2,3,6-tri-*O*-acetyl-α-D-galactopyranosyl chloride** (**11**) ~1:9, **11** shown ^1H-^1H COSY NMR (400 MHz) in $CDCl_3$

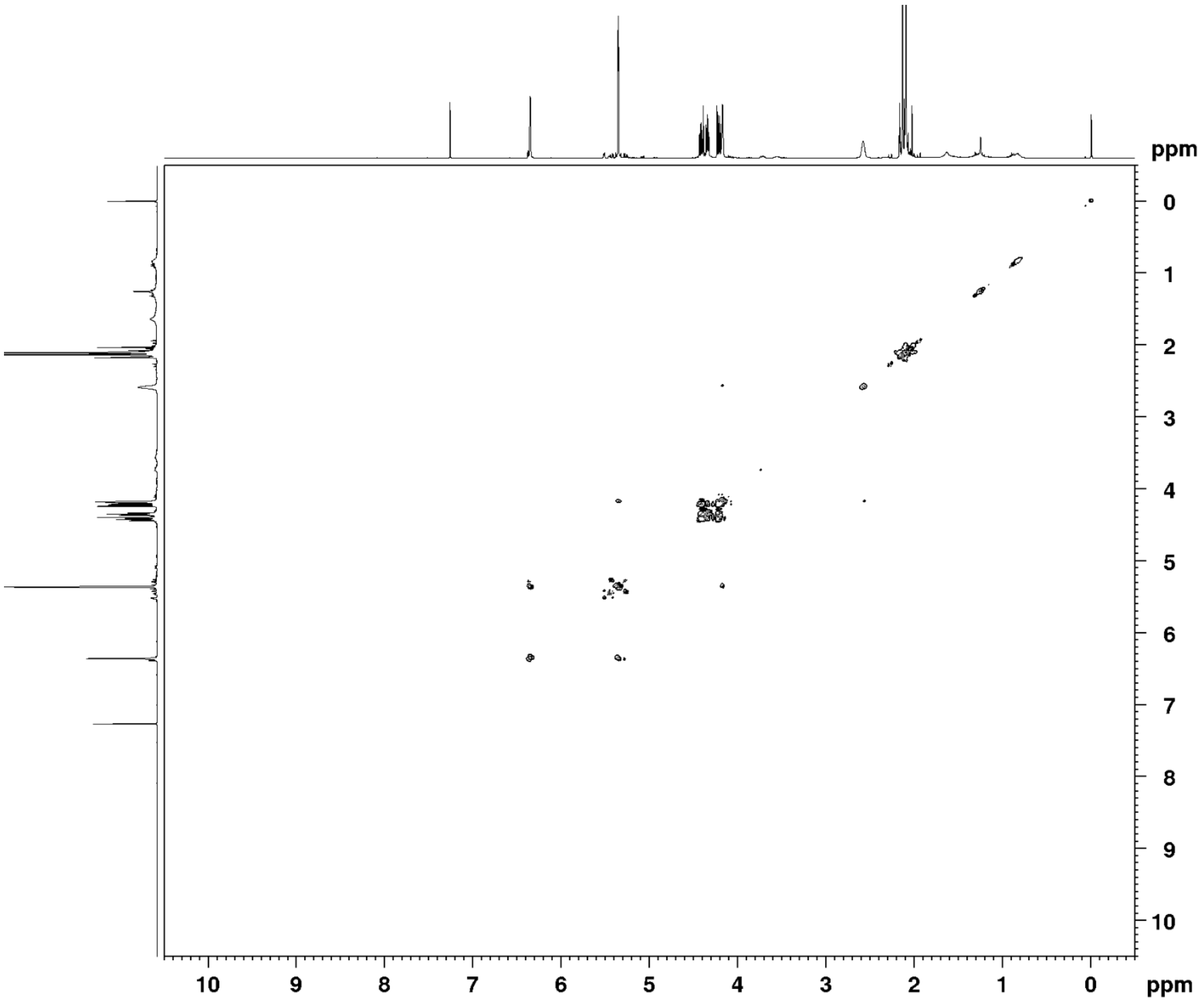

Mixture of 2,3,4-tri-*O*-acetyl-α-D-galactopyranosyl chloride (**10**) and **2,3,6-tri-*O*-acetyl-α-D-galactopyranosyl chloride** (**11**) ~1:9, **11** shown ^{1}H-$^{13}C\{^{1}H\}$ HSQC NMR (400 & 101 MHz) in $CDCl_3$

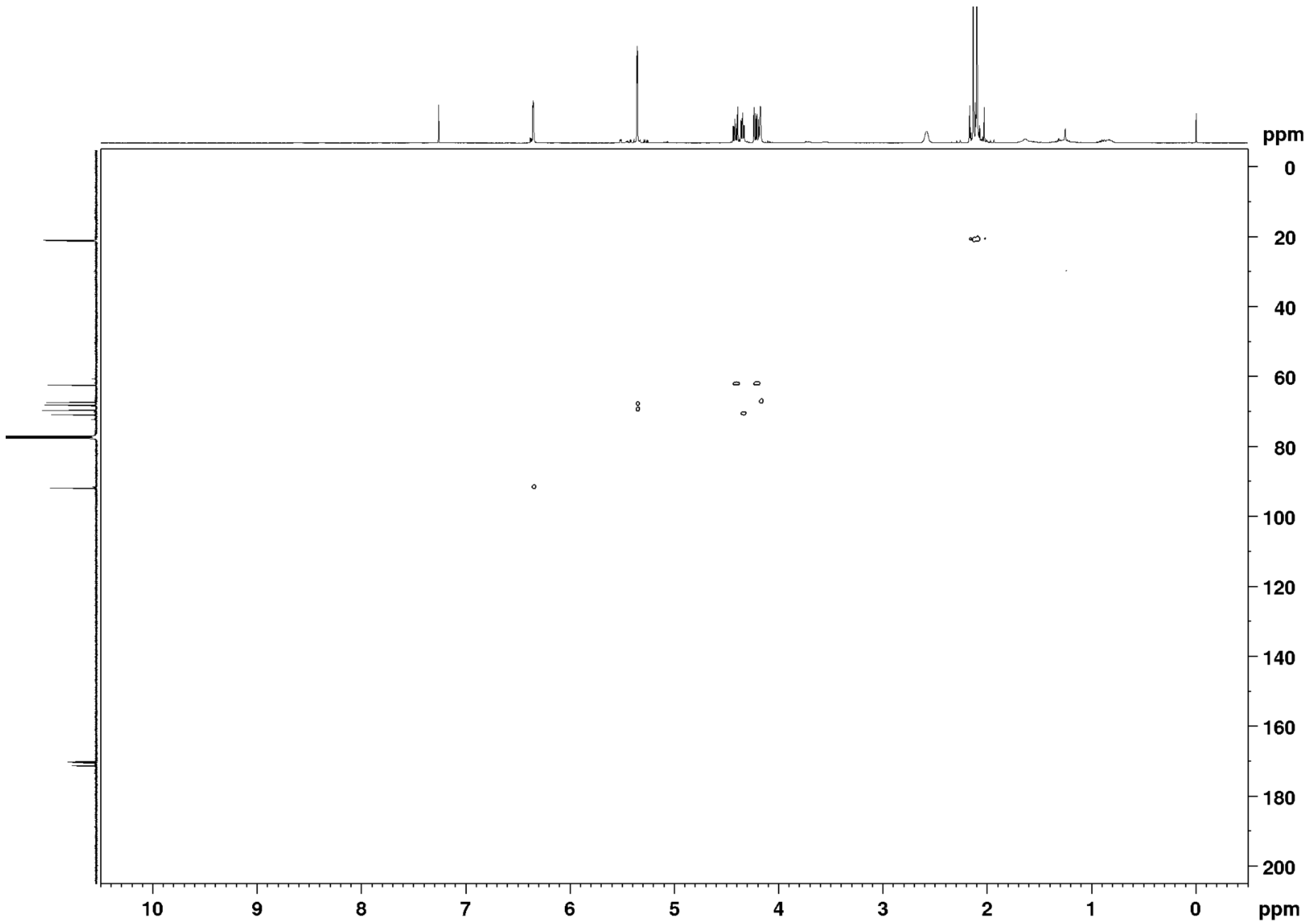

Mixture of 2,3,4-tri-*O*-acetyl-α-D-galactopyranosyl chloride (**10**) and **2,3,6-tri-*O*-acetyl-α-D-galactopyranosyl chloride** (**11**) ~1:9, **11** shown ^{1}H-$^{13}C\{^{1}H\}$ HMBC NMR (400 & 101 MHz) in $CDCl_3$

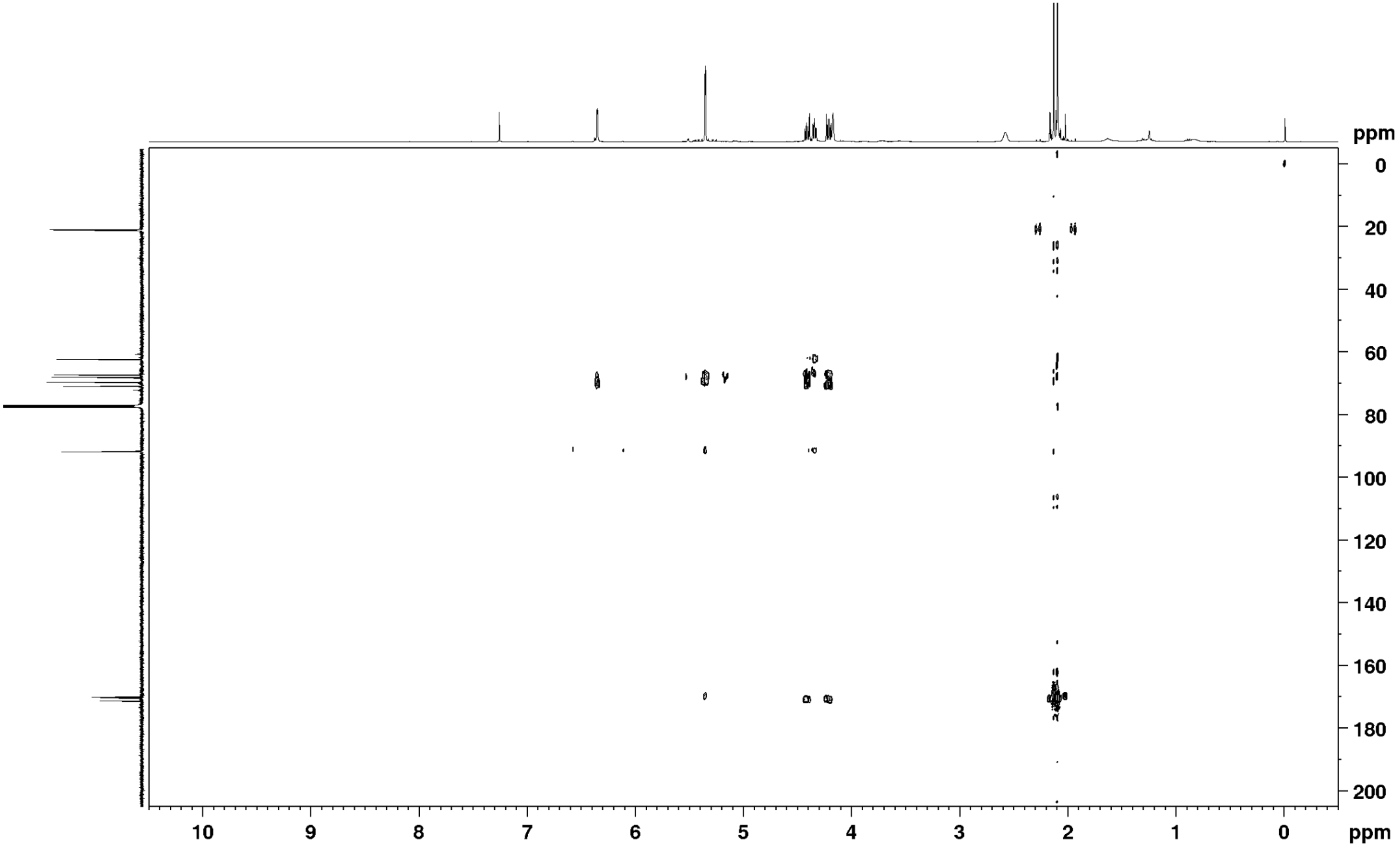

Mixture of 2,3,4-tri-*O*-acetyl-α-D-galactopyranosyl chloride (**10**) and **2,3,6-tri-*O*-acetyl-α-D-galactopyranosyl chloride** (**11**) ~1:9, **11** shown $^{13}C\{^{1}H\}$ NMR (101 MHz) in $CDCl_3$

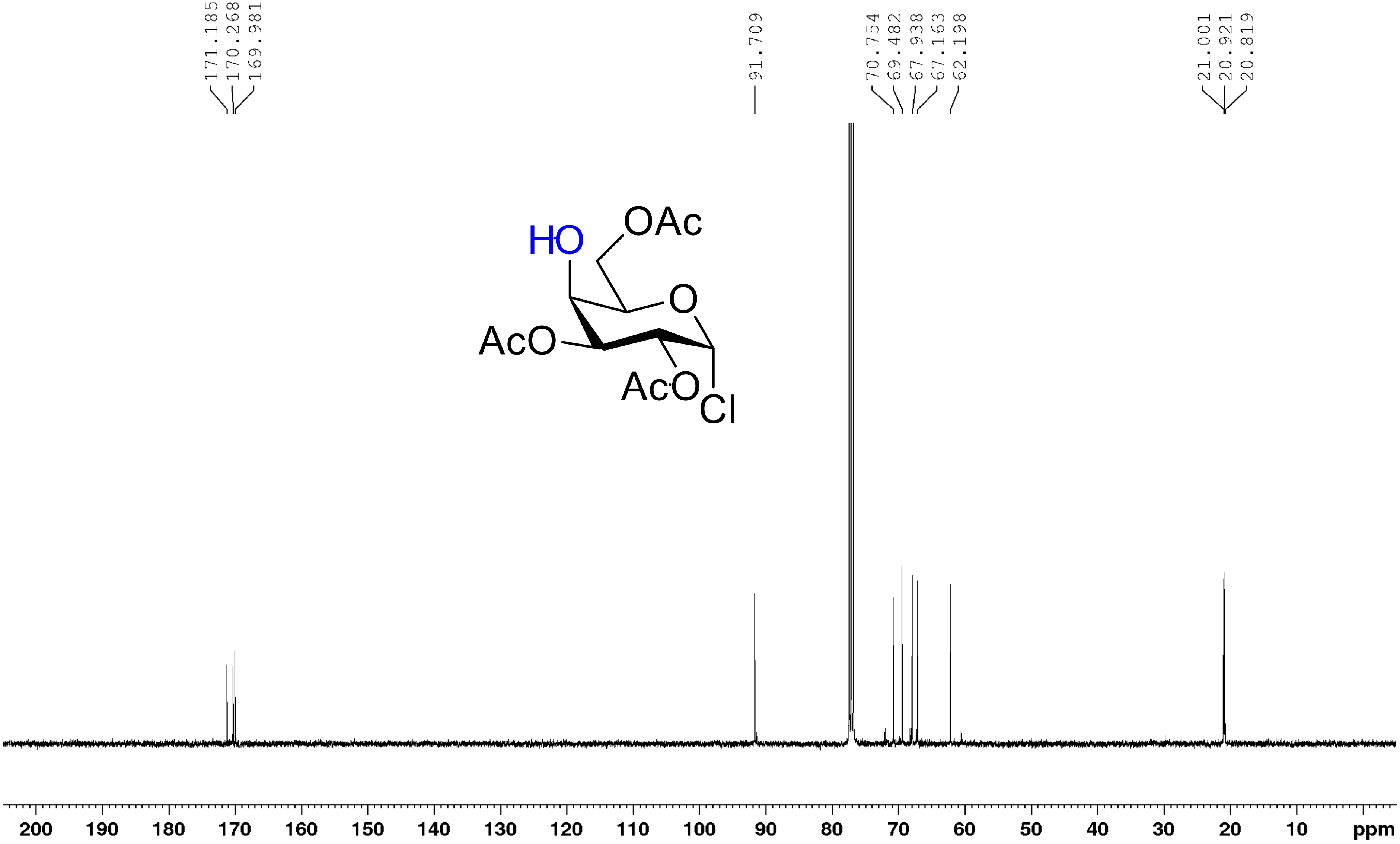

4-chlorophenyl 2,3,4,6-tetra-*O*-acetyl-1-thio-β-D-galactopyranoside (**12**) ^{1}H NMR (400 MHz) in $CDCl_3$

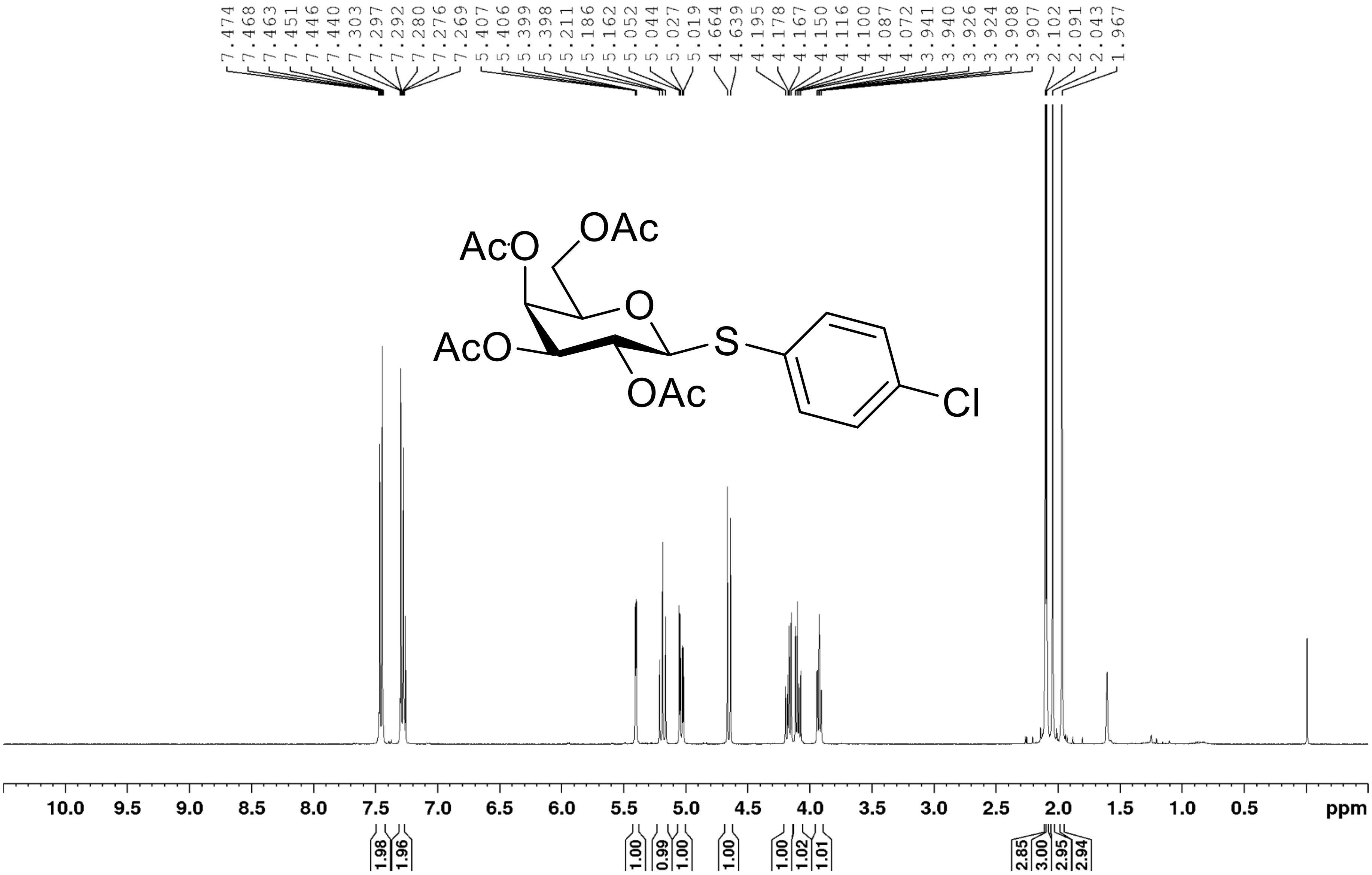

4-chlorophenyl 2,3,4-tri-*O*-acetyl-1-thio-β-D-galactopyranoside (**13**) ^{1}H NMR (400 MHz) in $CDCl_3$

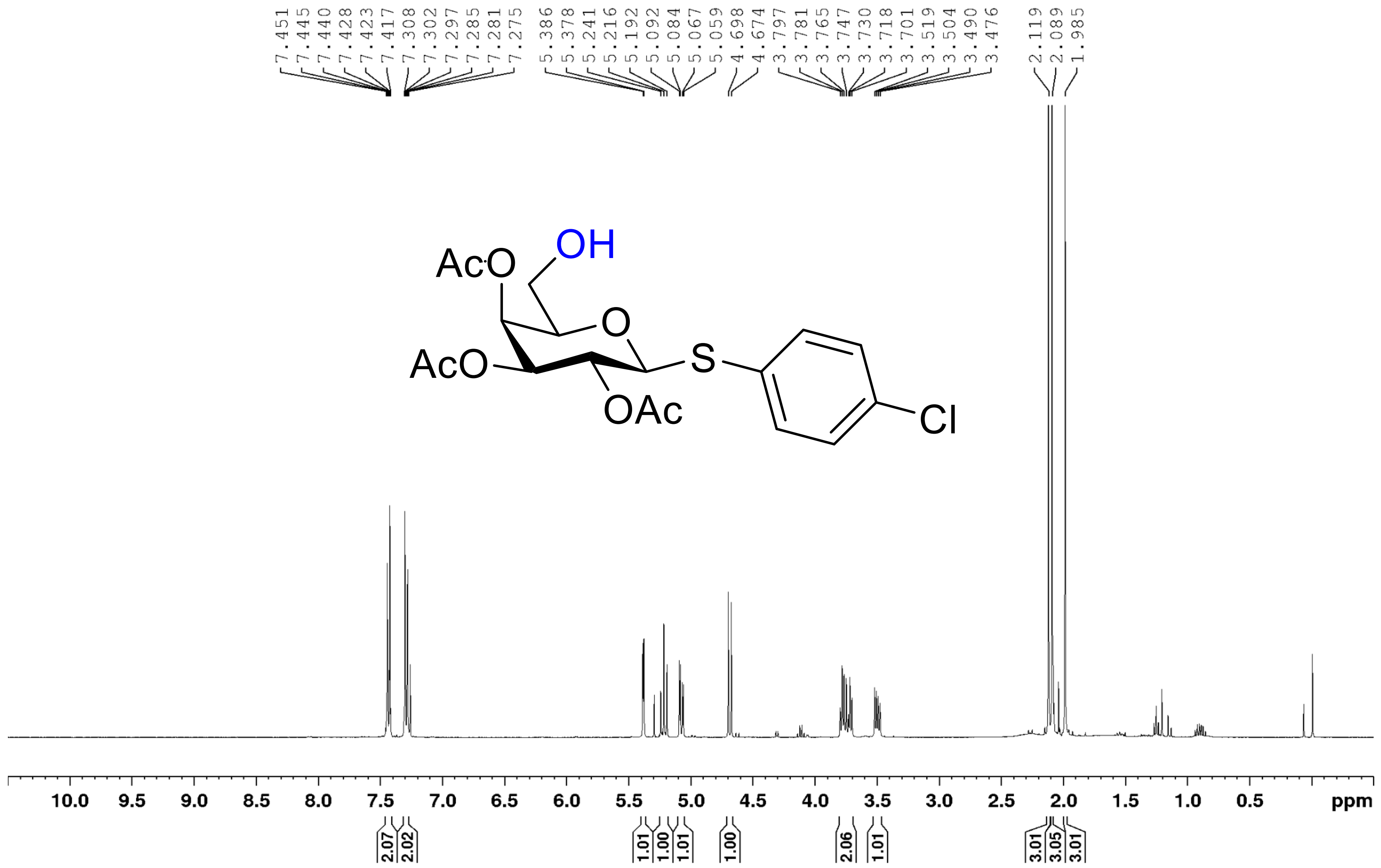

4-chlorophenyl 2,3,4-tri-*O*-acetyl-1-thio-β-D-galactopyranoside (**13**) ^{1}H-^{1}H COSY NMR (400 MHz) in $CDCl_3$

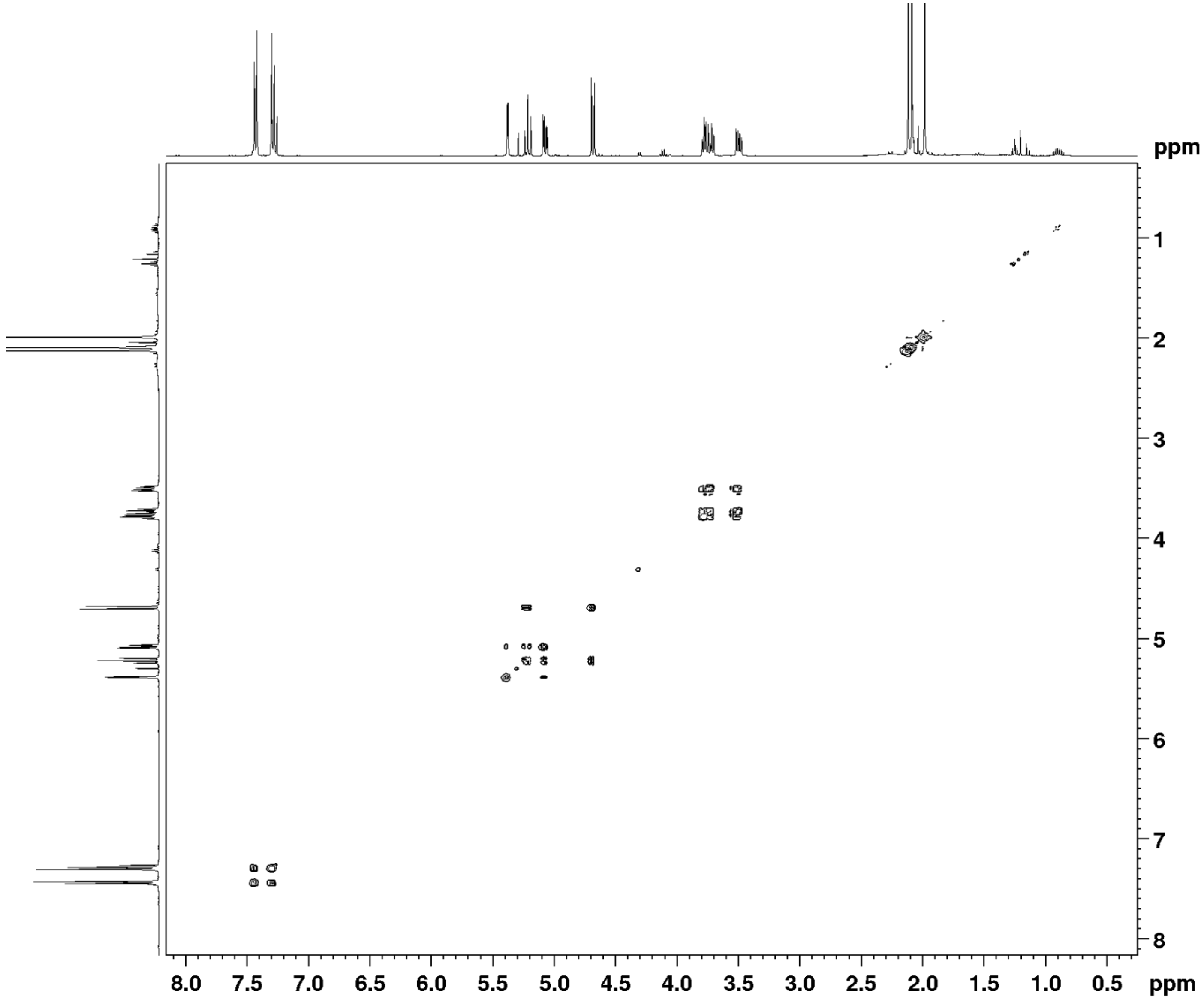

4-chlorophenyl 2,3,4-tri-*O*-acetyl-1-thio-β-D-galactopyranoside (**13**) ^{1}H-$^{13}C\{^{1}H\}$ HSQC NMR (400 & 101 MHz) in $CDCl_3$

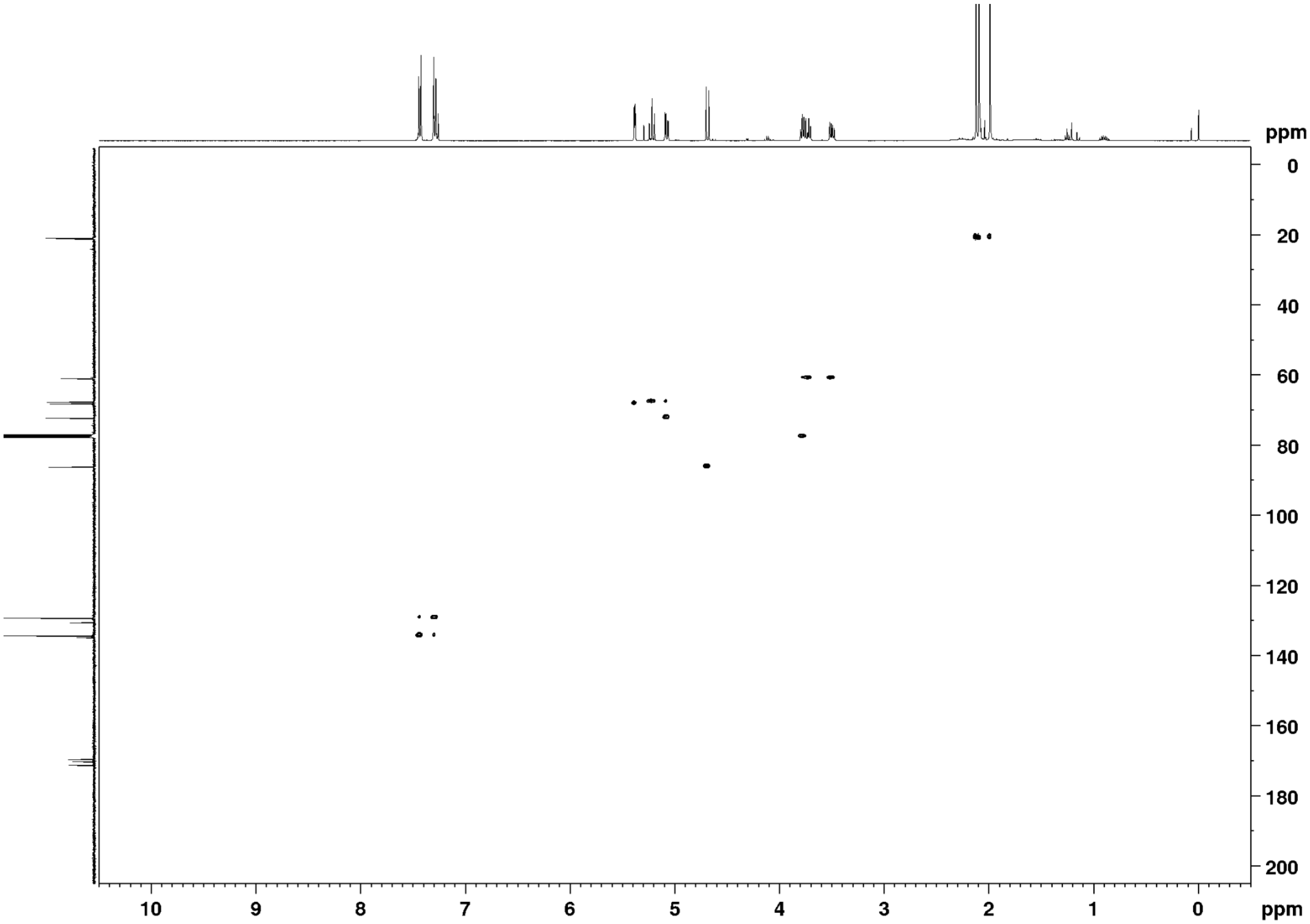

4-chlorophenyl 2,3,4-tri-*O*-acetyl-1-thio-β-D-galactopyranoside (**13**) ^{1}H-$^{13}C\{^{1}H\}$ HMBC NMR (400 & 101 MHz) in $CDCl_3$

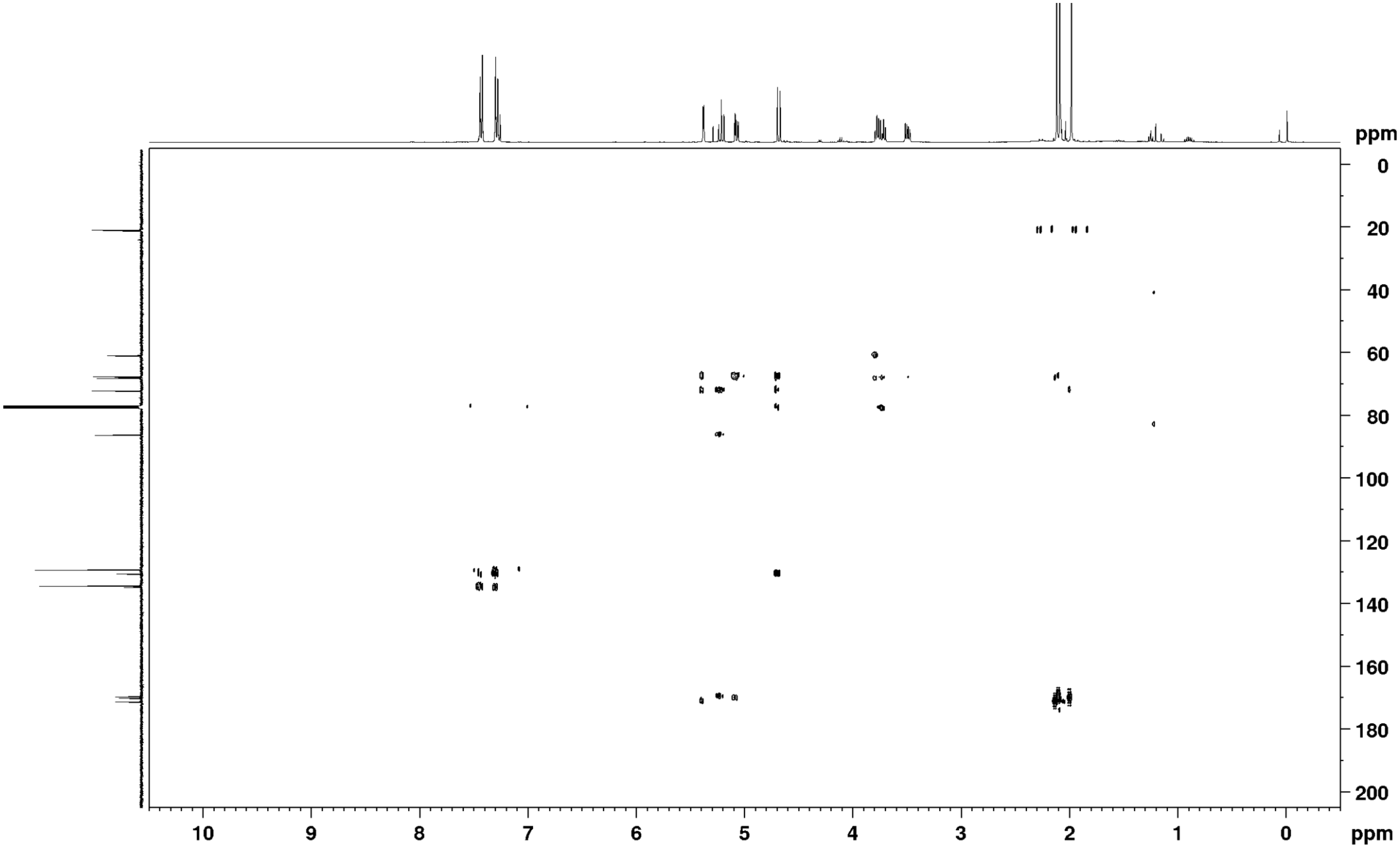

4-chlorophenyl 2,3,4-tri-*O*-acetyl-1-thio-β-D-galactopyranoside (13) $^{13}C\{^{1}H\}$ NMR (101 MHz) in $CDCl_3$

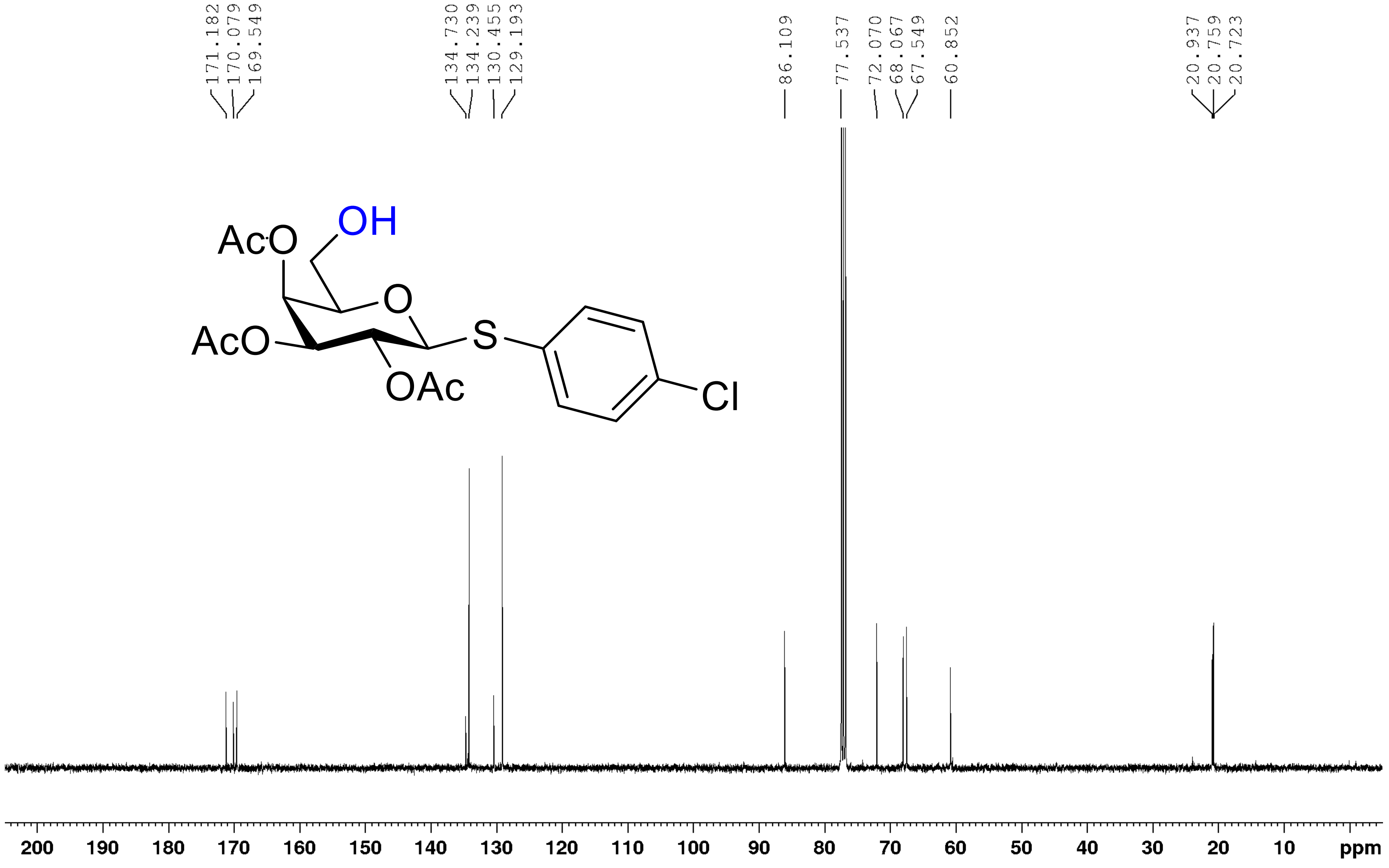

2,6-dimethylphenyl 2,3,4,6-tetra-*O*-acetyl-1-thio-D-galactopyranoside (**14**) α:β 75:25 ^{1}H NMR (400 MHz) in $CDCl_3$

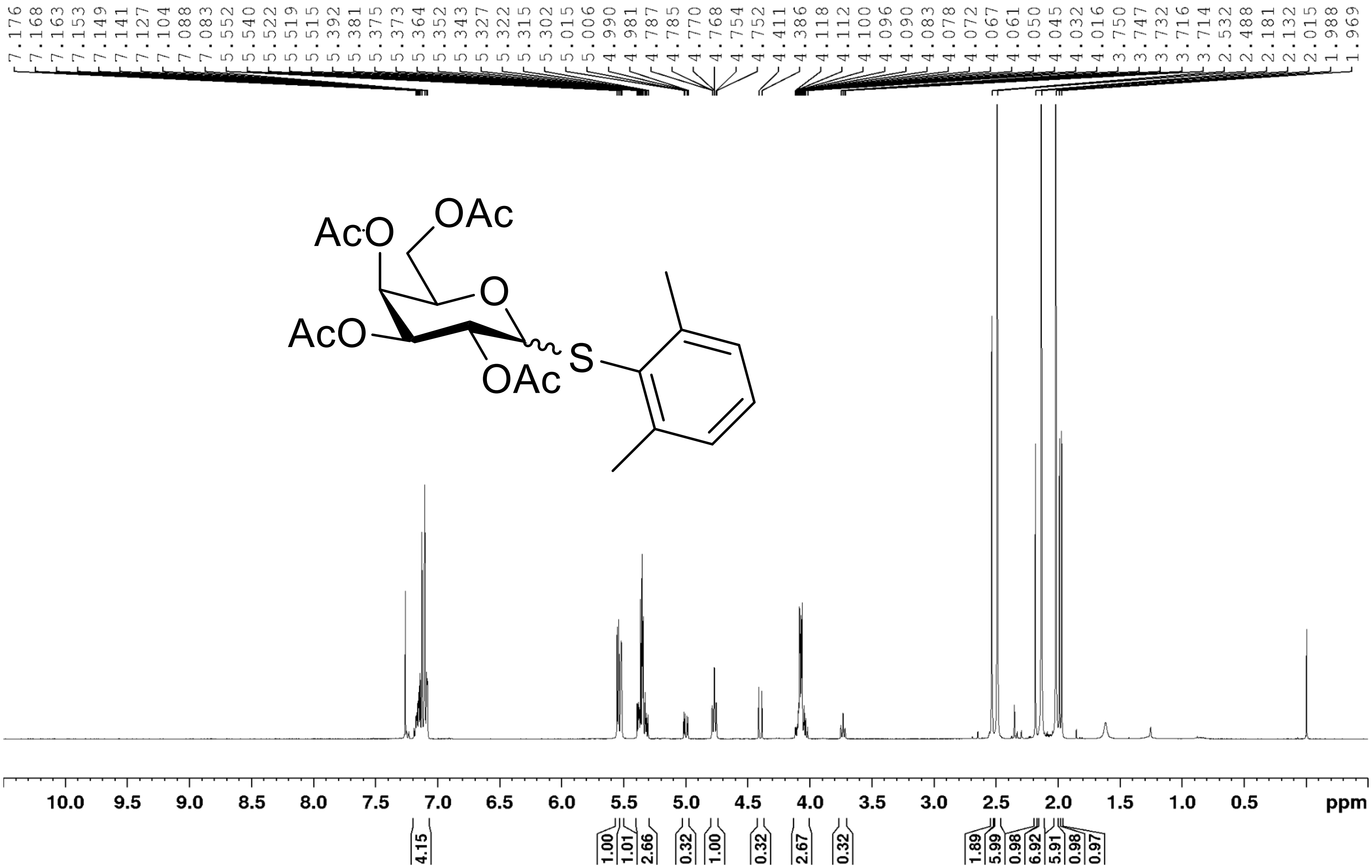

2,6-dimethylphenyl 2,3,4-tri-*O*-acetyl-1-thio-D-galactopyranoside (15) α:β 78:22 ^{1}H NMR (400 MHz) in $CDCl_3$

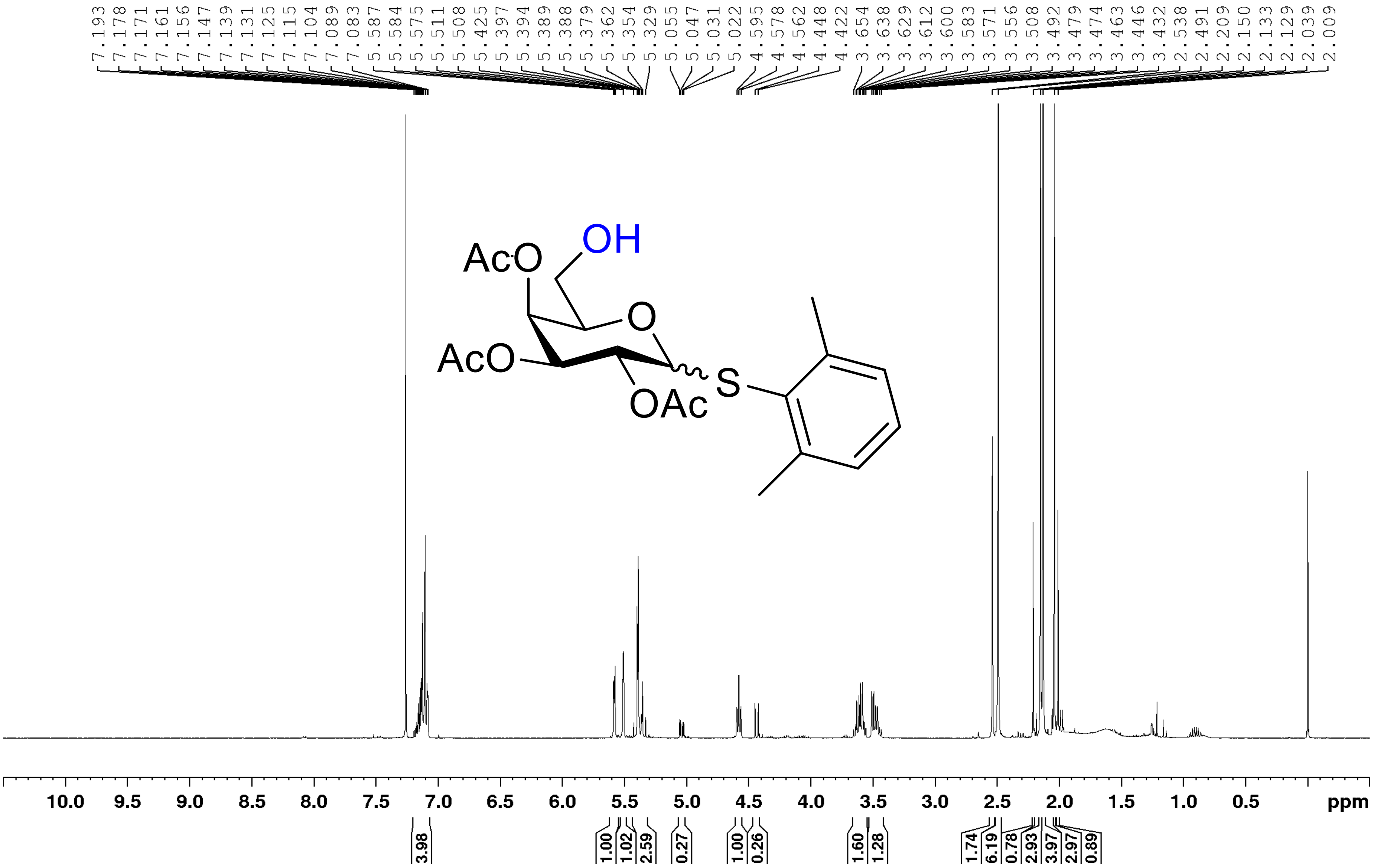

2,6-dimethylphenyl 2,3,4-tri-*O*-acetyl-1-thio-D-galactopyranoside (**15**) α:β 78:22 ^{1}H-^{1}H COSY NMR (400 MHz) in $CDCl_3$

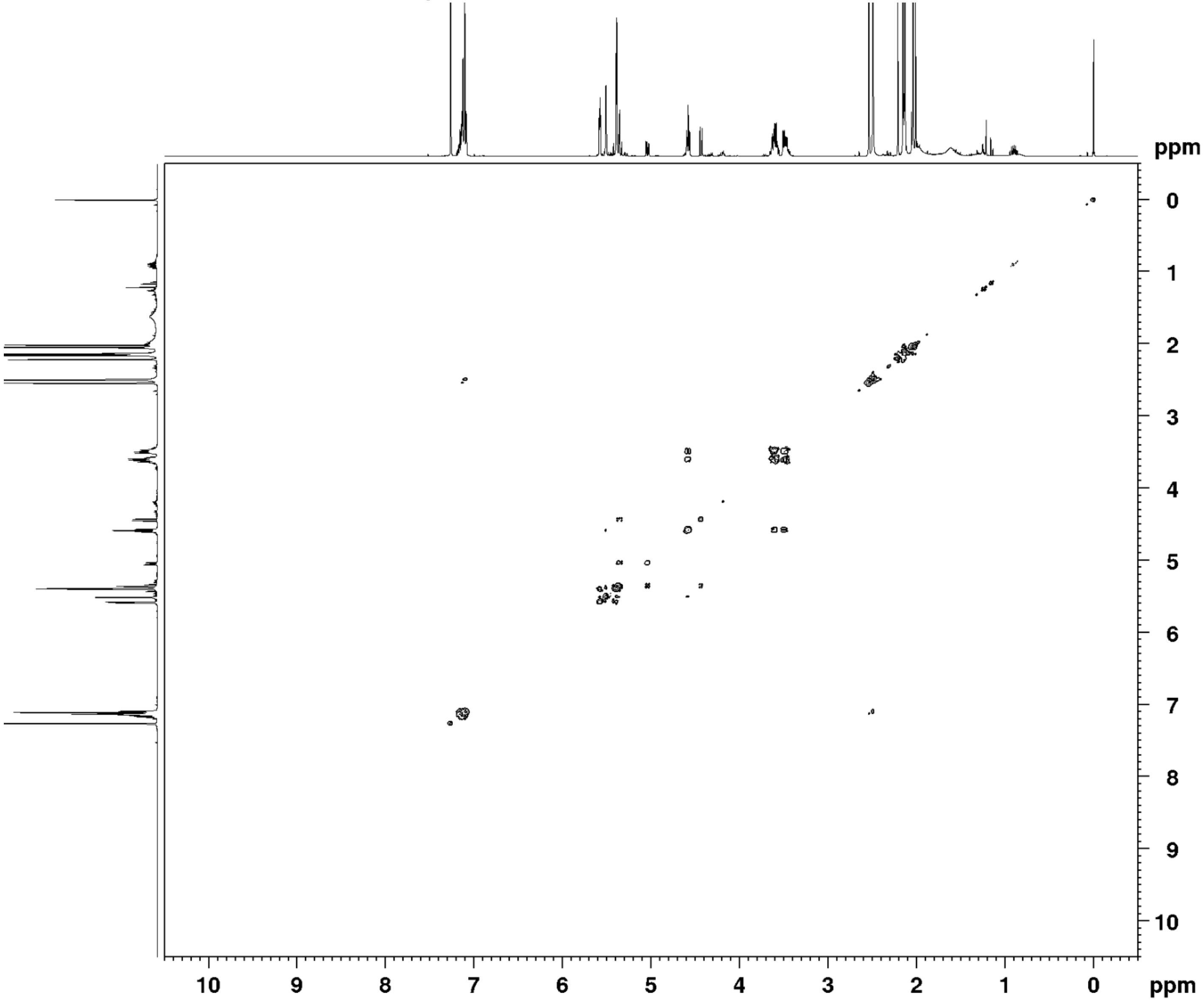

2,6-dimethylphenyl 2,3,4-tri-*O*-acetyl-1-thio-D-galactopyranoside (**15**) α:β 78:22 ^{1}H-$^{13}C\{^{1}H\}$ HSQC NMR (400 & 101 MHz) in $CDCl_3$

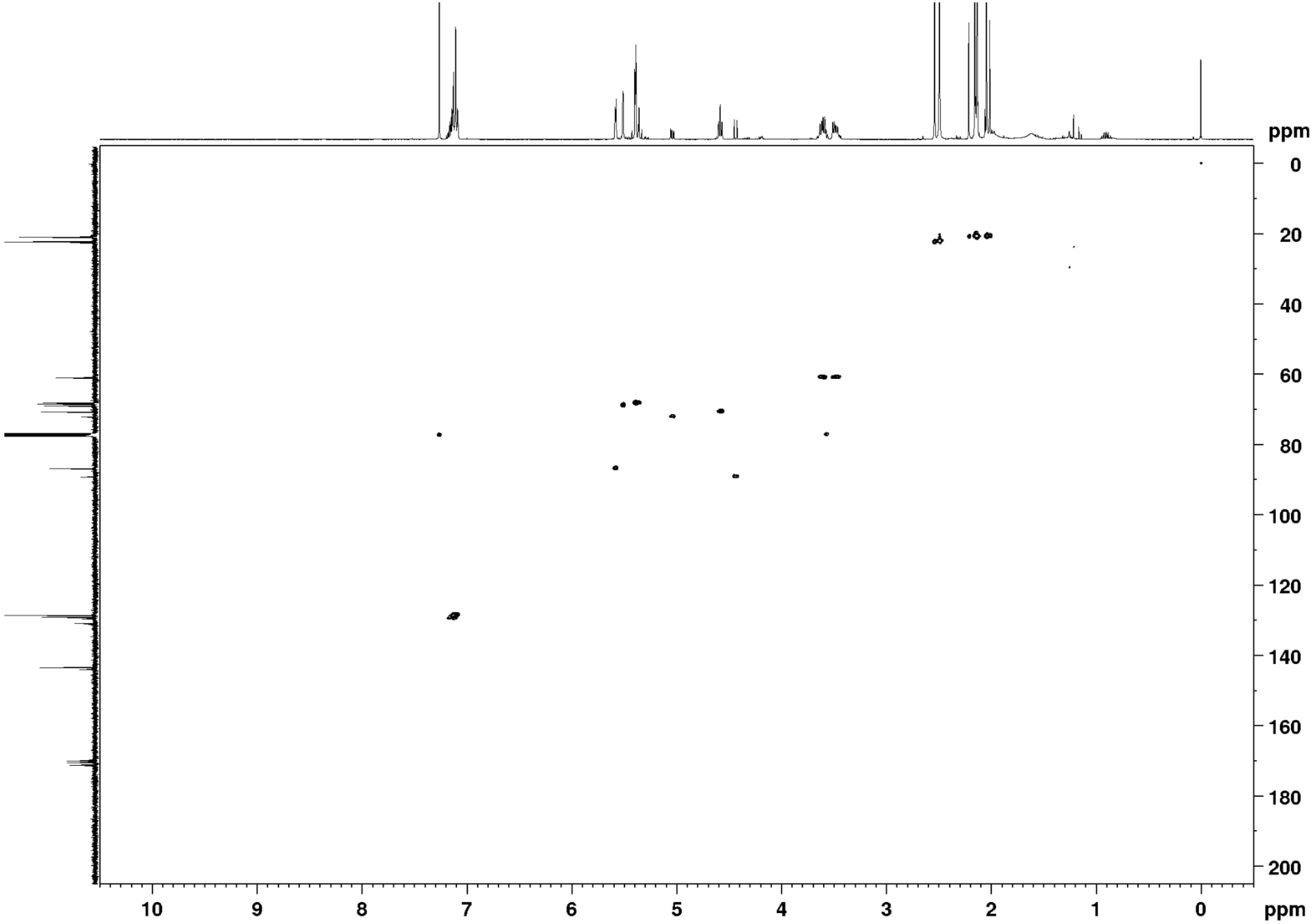

2,6-dimethylphenyl 2,3,4-tri-*O*-acetyl-1-thio-D-galactopyranoside (**15**) α:β 78:22 ^{1}H-$^{13}C\{^{1}H\}$ HMBC NMR (400 & 101 MHz) in $CDCl_3$

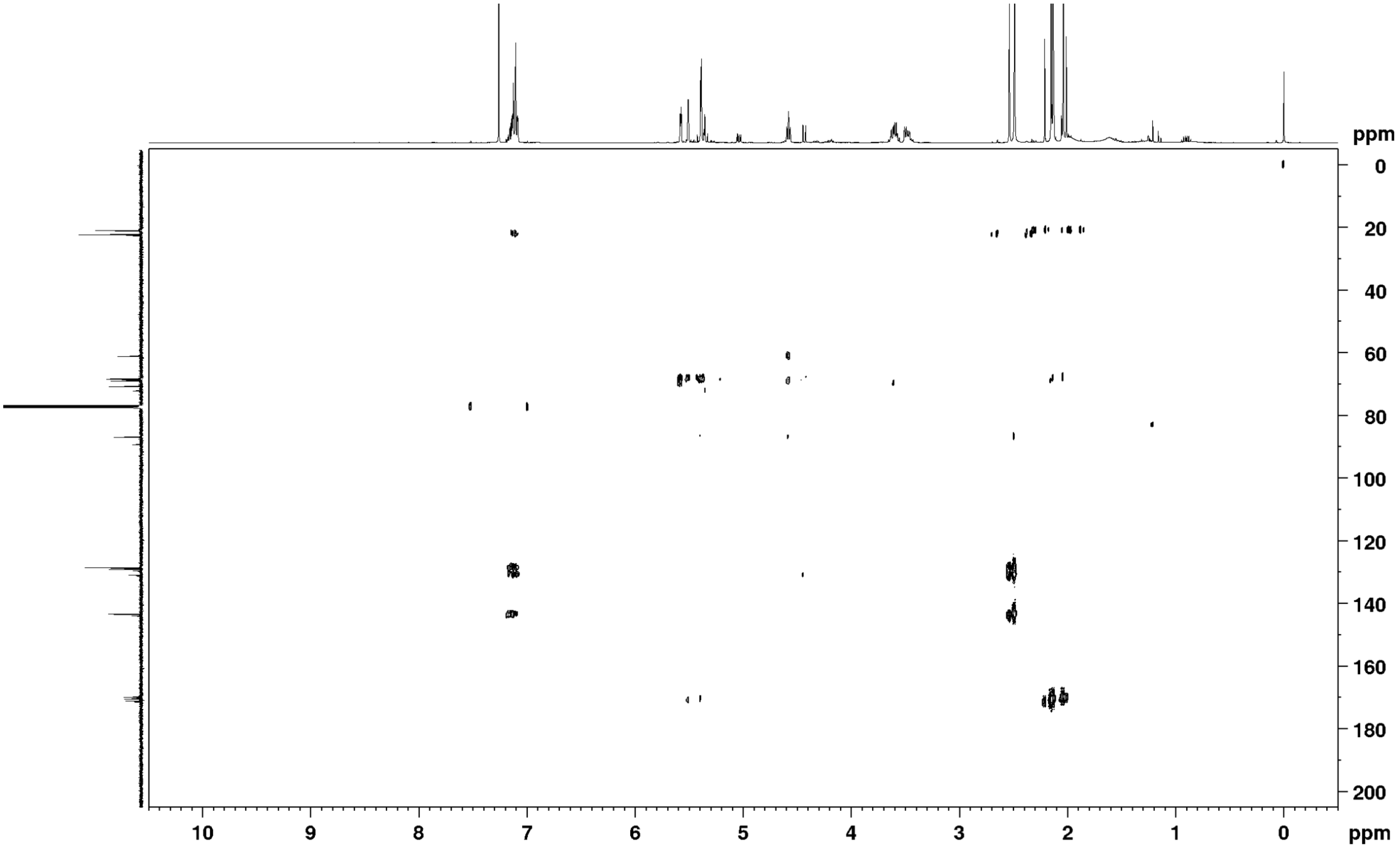

2,6-dimethylphenyl 2,3,4-tri-*O*-acetyl-1-thio-D-galactopyranoside (**15**) α:β 78:22 $^{13}C\{^{1}H\}$ NMR (101 MHz) in $CDCl_3$

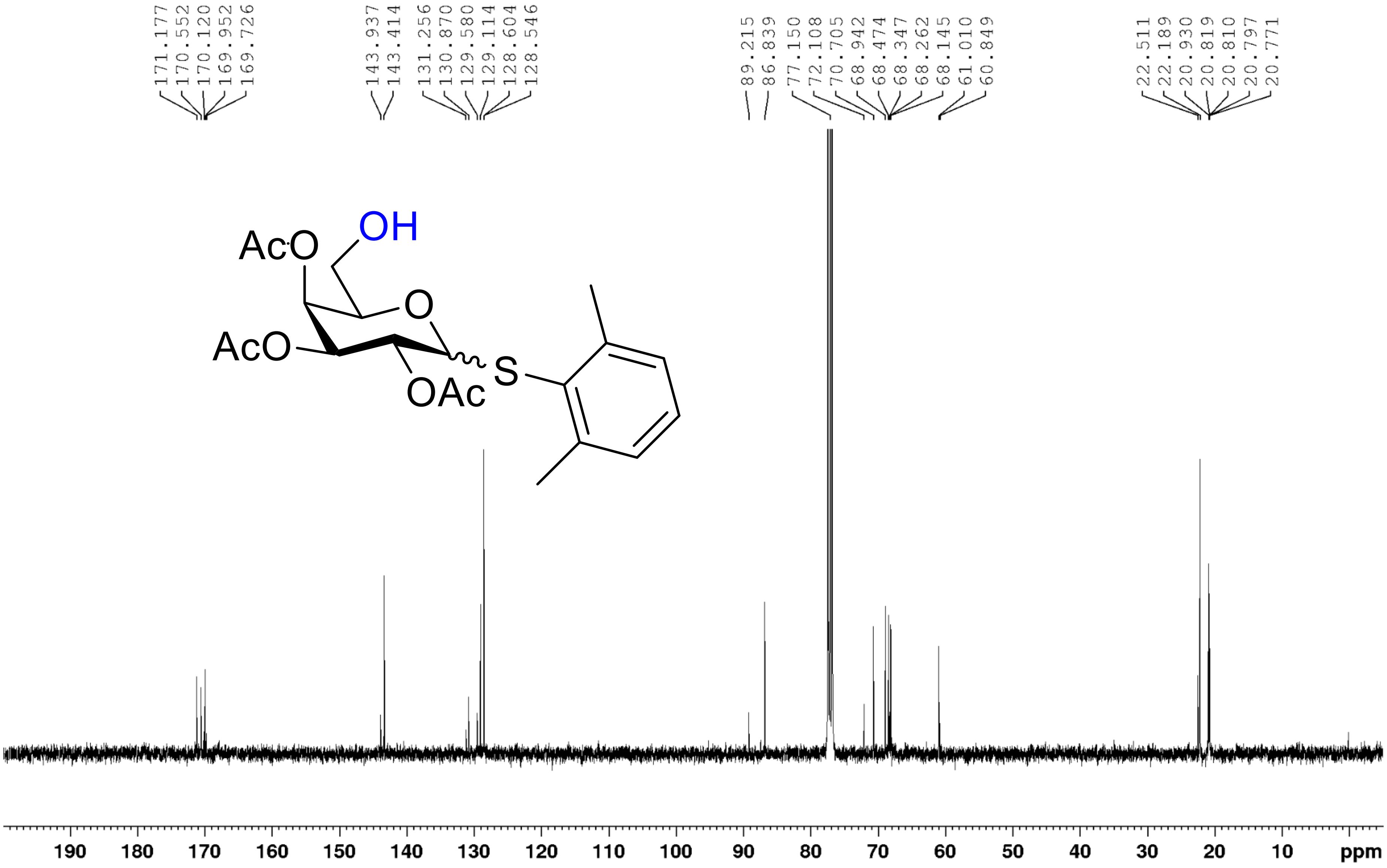

4-chlorophenyl (2,3,4,6-tetra-*O*-acetyl-β-D-galactopyranosyl)-(1→4)-2,3,6-tri-*O*-acetyl-1-thio-D-glucopyranoside (**16**) α:β 21:79 ^{1}H NMR (400 MHz) in $CDCl_3$

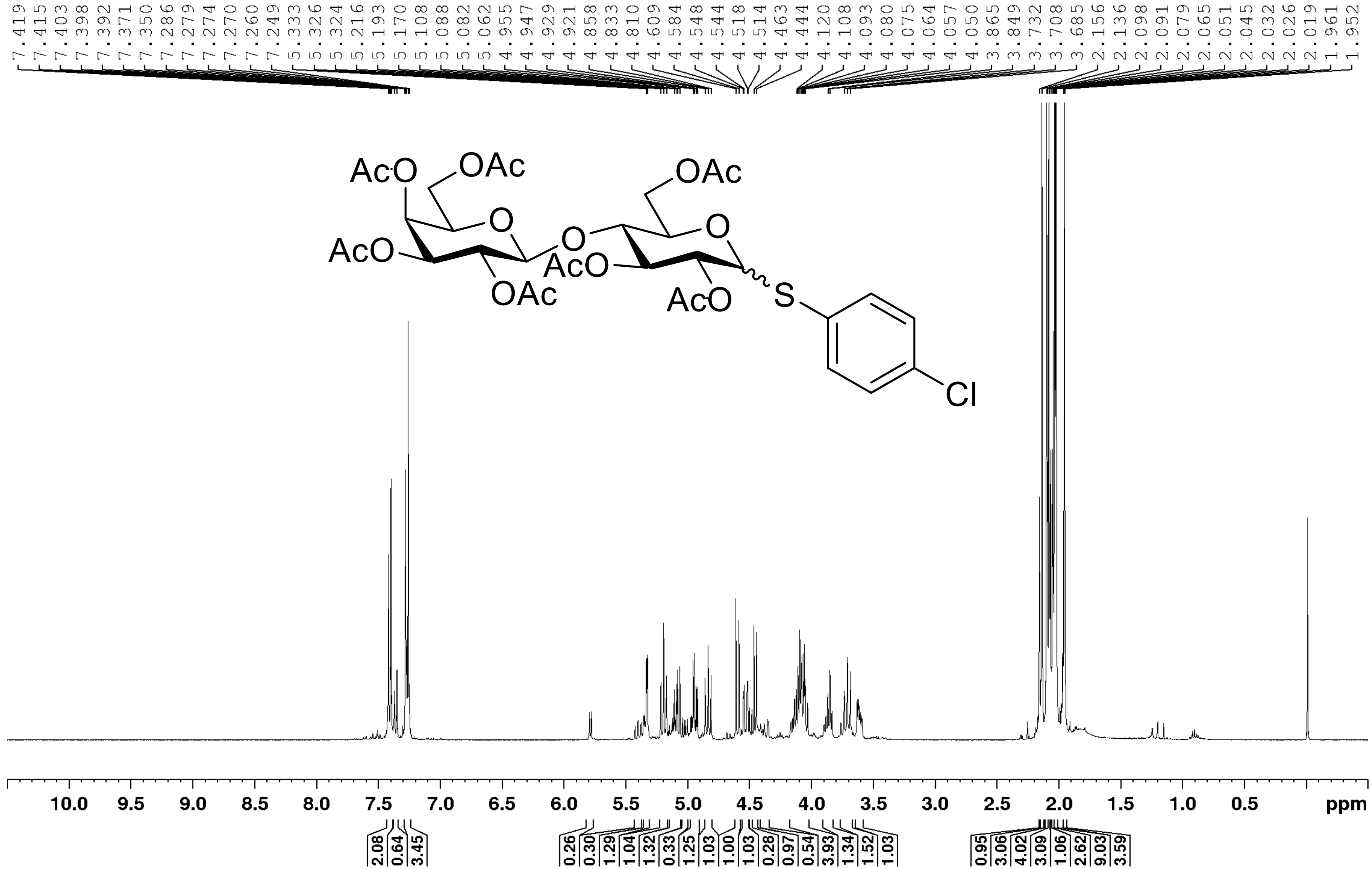

4-chlorophenyl (2,3,4,6-tetra-*O*-acetyl-β-D-galactopyranosyl)-(1→4)-2,3,6-tri-*O*-acetyl-1-thio-D-glucopyranoside (16) α:β 21:79 ^{1}H-^{1}H COSY NMR (400 MHz) in $CDCl_3$

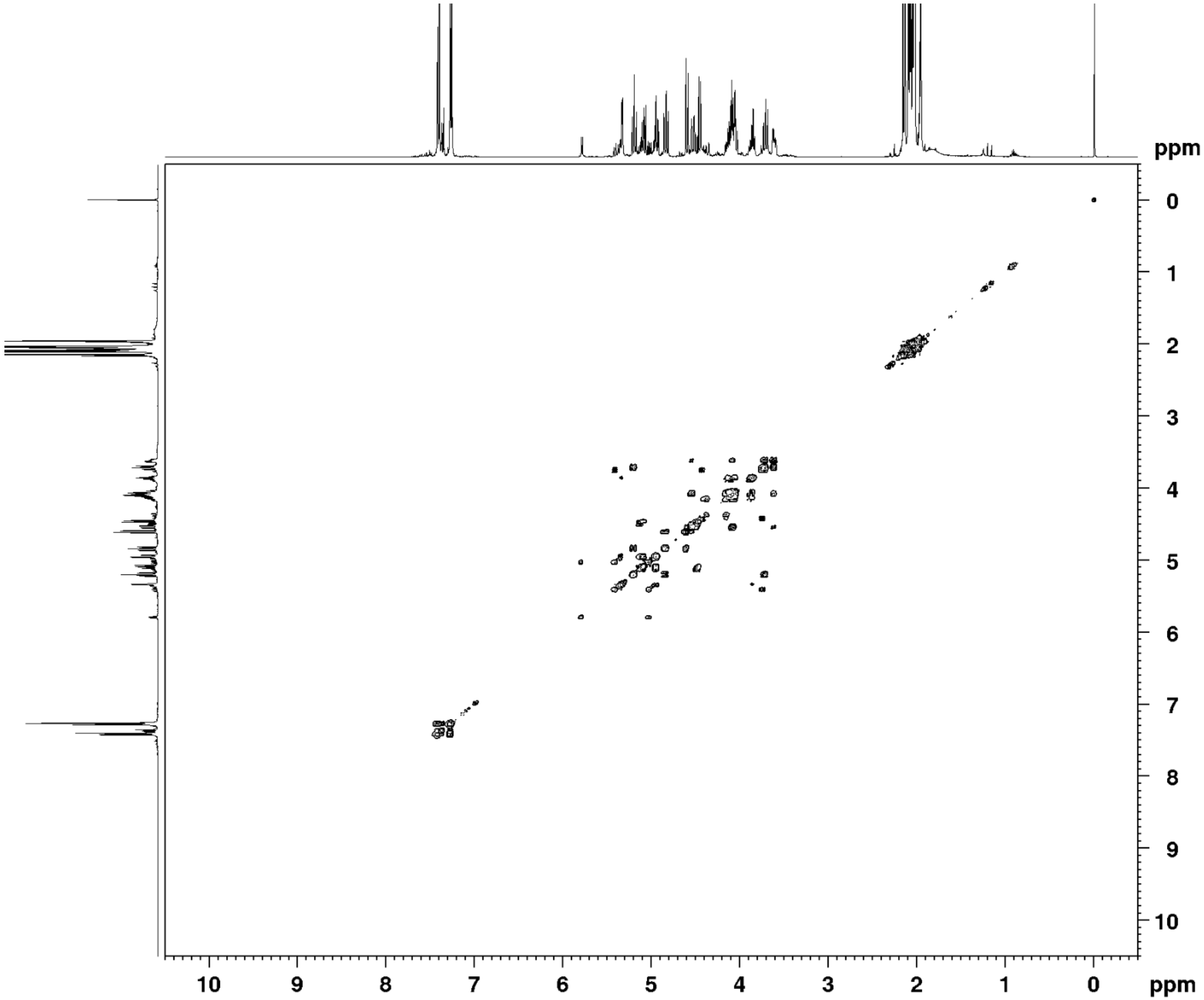

4-chlorophenyl (2,3,4,6-tetra-*O*-acetyl-β-D-galactopyranosyl)-(1→4)-2,3,6-tri-*O*-acetyl-1-thio-D-glucopyranoside (**16**) α:β 21:79 ^{1}H-$^{13}C\{^{1}H\}$ HSQC NMR (400 & 101 MHz) in $CDCl_3$

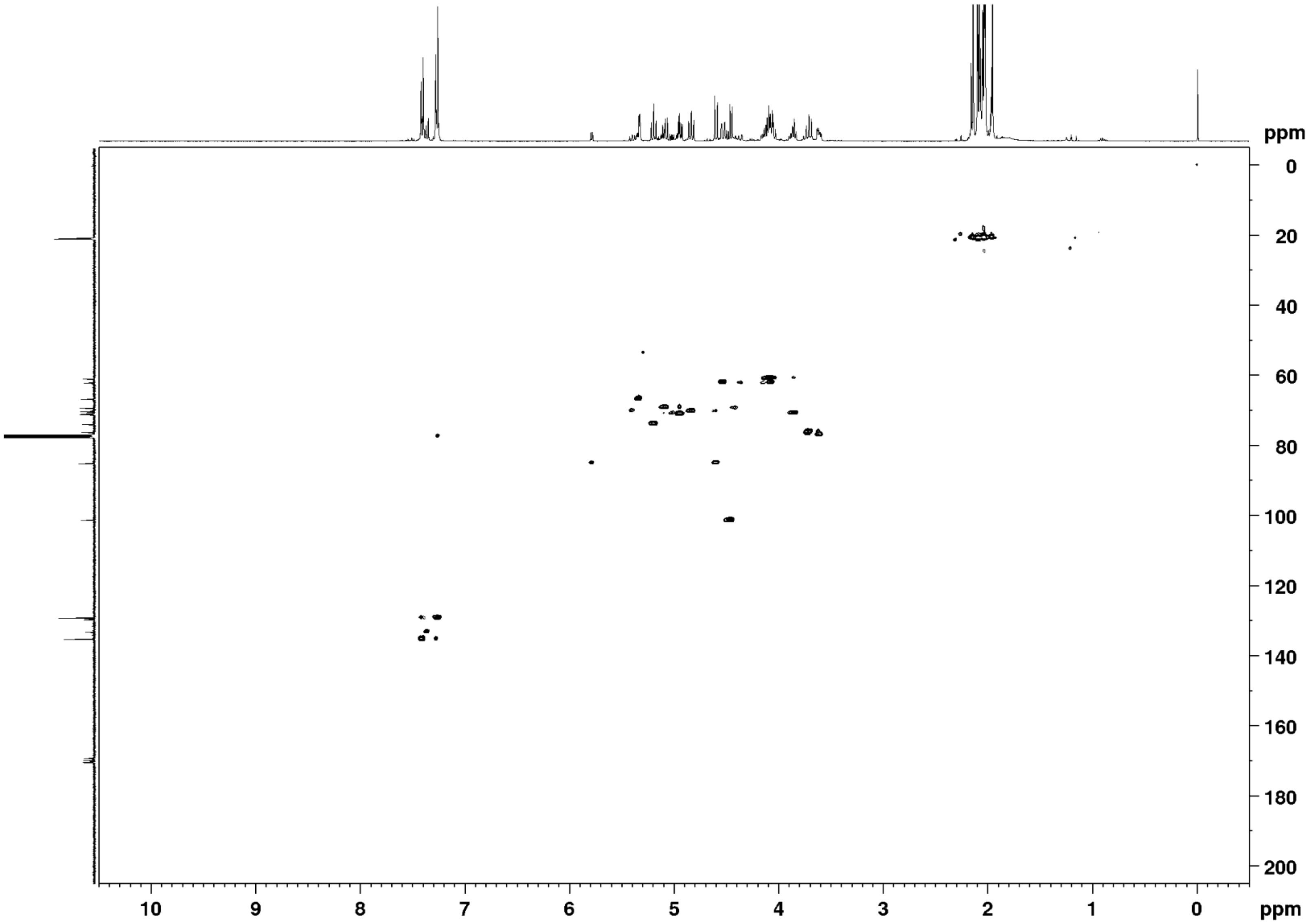

4-chlorophenyl (2,3,4,6-tetra-*O*-acetyl-β-D-galactopyranosyl)-(1→4)-2,3,6-tri-*O*-acetyl-1-thio-D-glucopyranoside (**16**) α:β 21:79 ^{1}H-$^{13}C\{^{1}H\}$ HMBC NMR (400 & 101 MHz) in $CDCl_3$

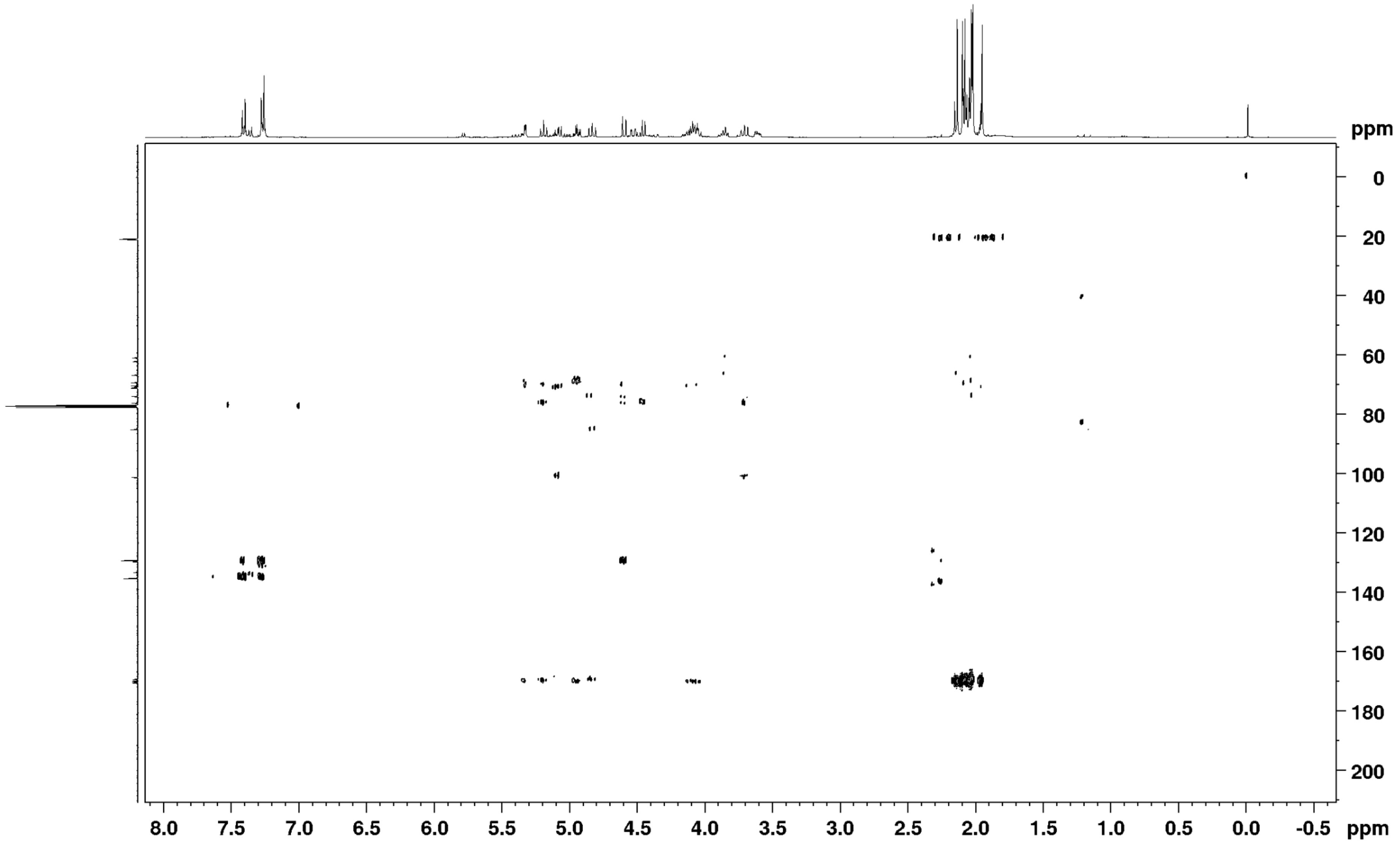

4-chlorophenyl (2,3,4,6-tetra-*O*-acetyl-β-D-galactopyranosyl)-(1→4)-2,3,6-tri-*O*-acetyl-1-thio-D-glucopyranoside (**16**) α:β 21:79 $^{13}C\{^{1}H\}$ NMR (101 MHz) in $CDCl_3$

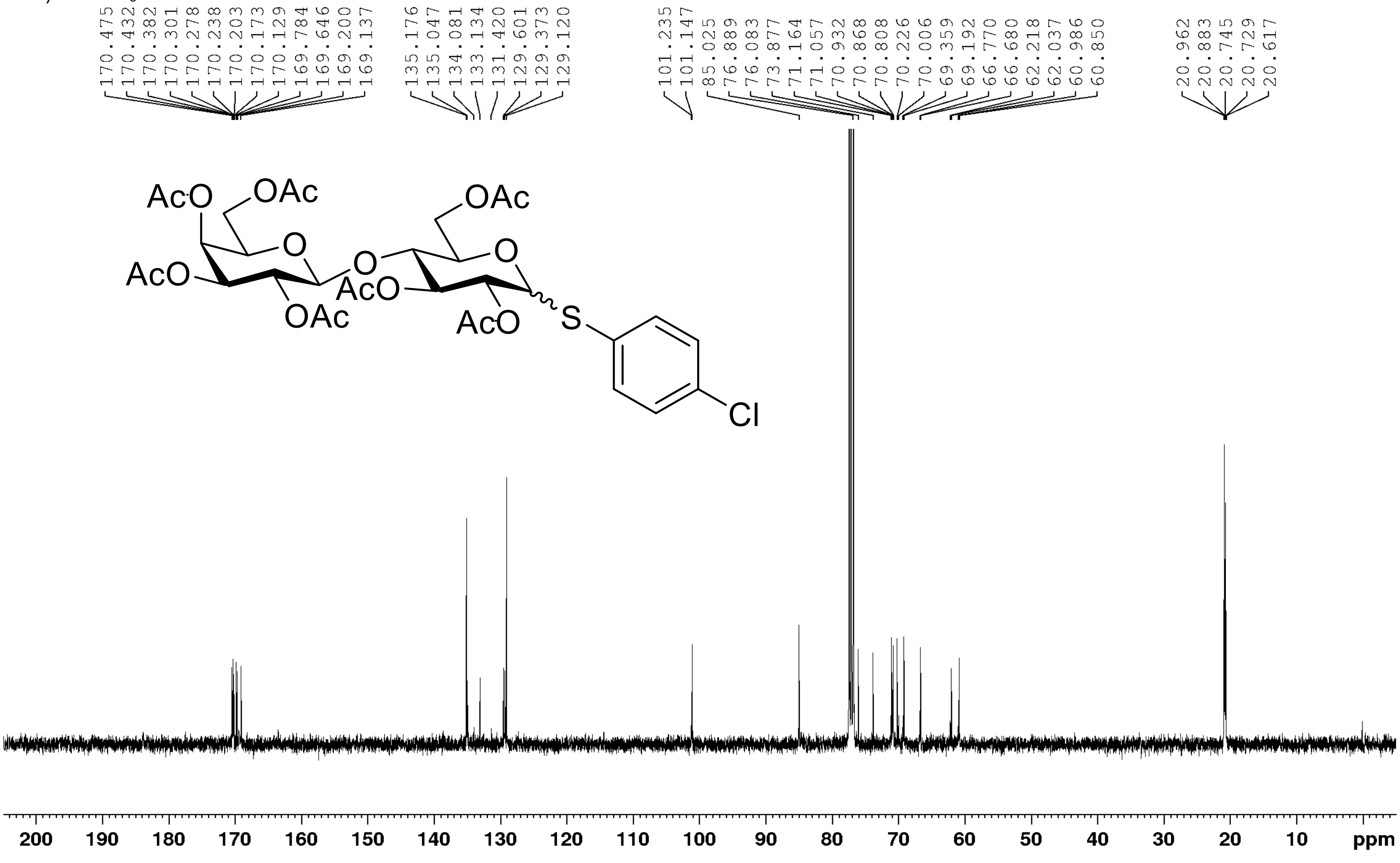

4-chlorophenyl (2,3,4,6-tetra-*O*-acetyl-β-D-galactopyranosyl)-(1→4)-2,3-di-*O*-acetyl-1-thio-β-D-glucopyranoside (17) ^{1}H NMR (400 MHz) in $CDCl_3$

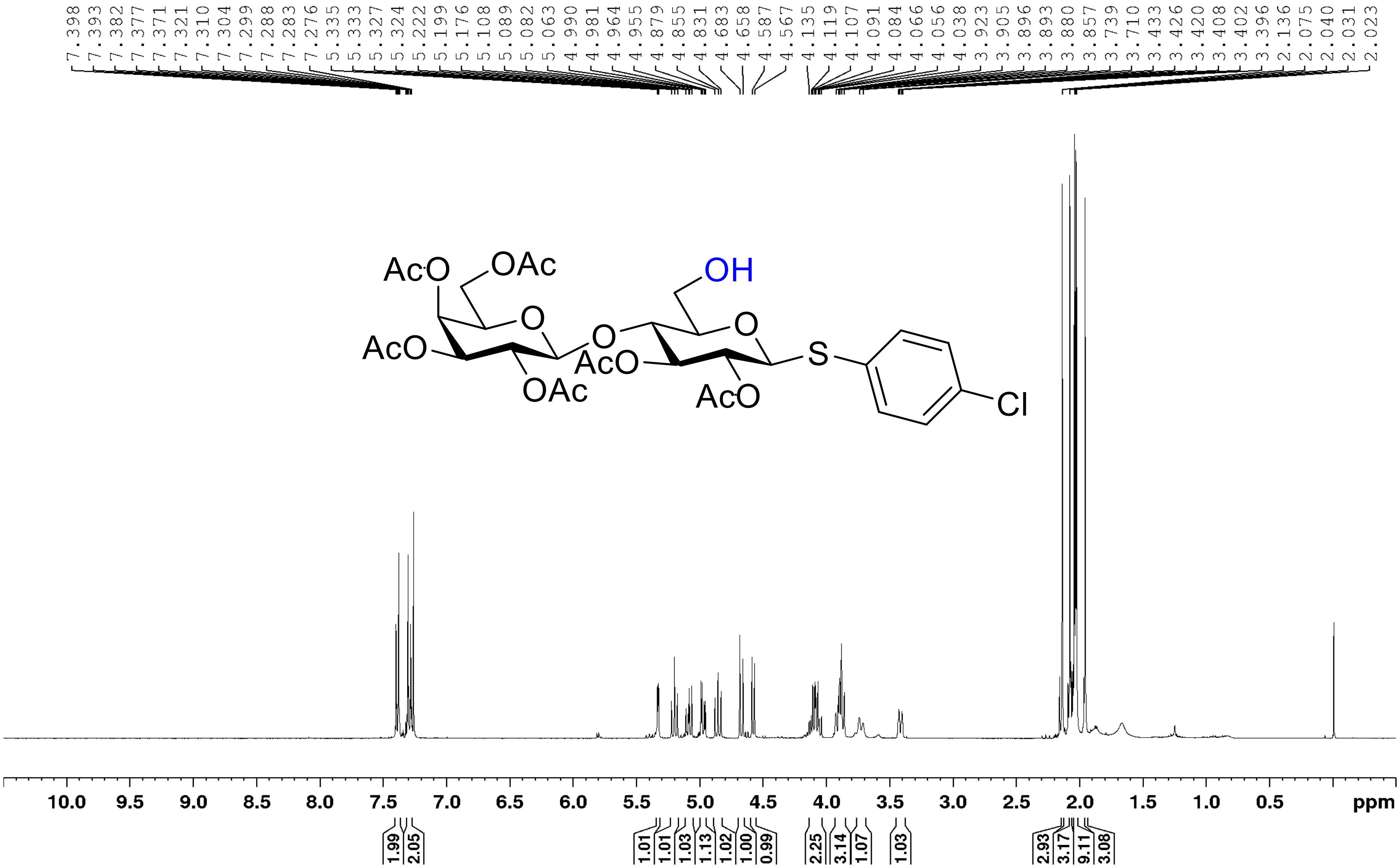

4-chlorophenyl (2,3,4,6-tetra-*O*-acetyl-β-D-galactopyranosyl)-(1→4)-2,3-di-*O*-acetyl-1-thio-β-D-glucopyranoside (**17**) ^{1}H-^{1}H COSY NMR (400 MHz) in $CDCl_3$

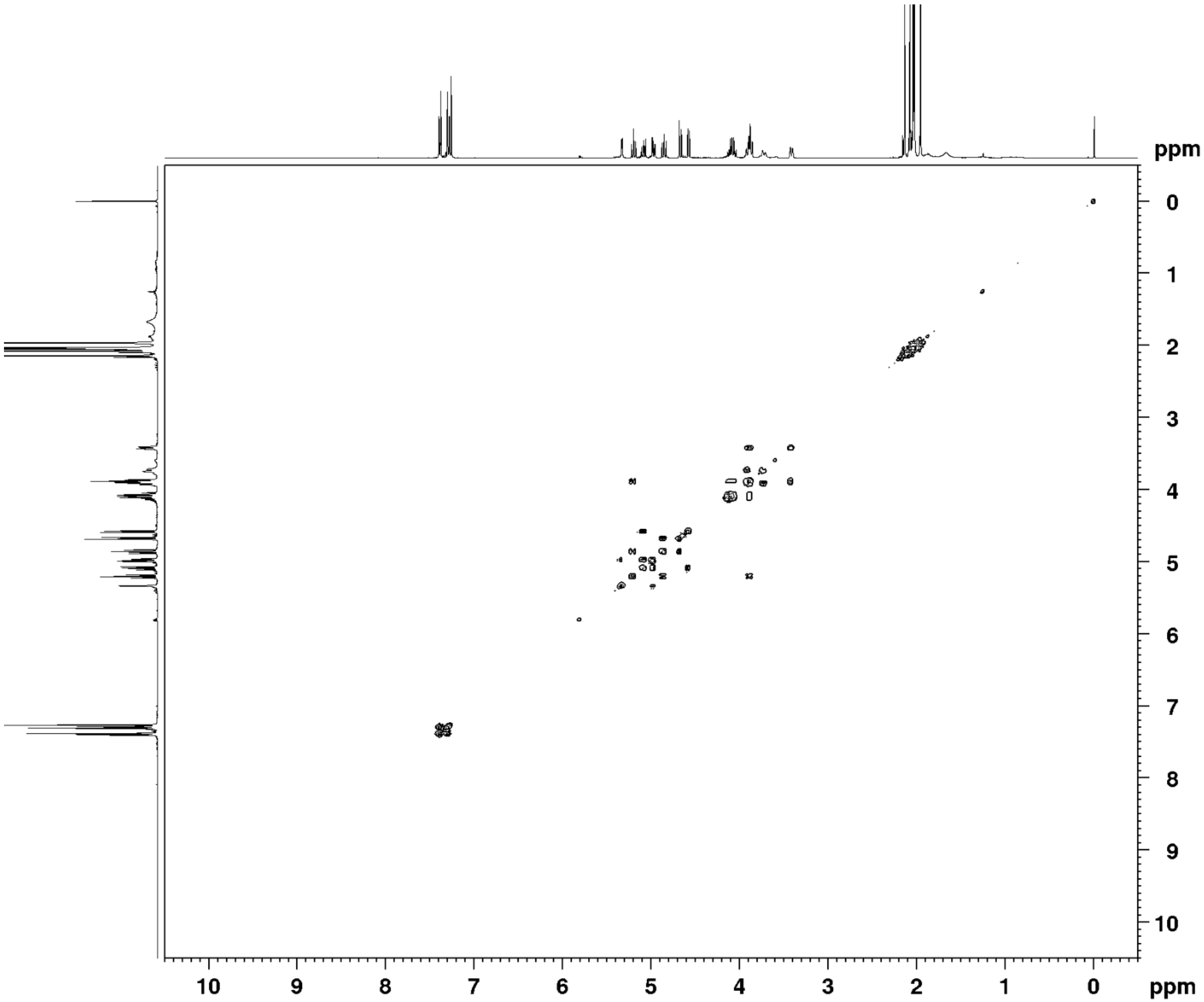

4-chlorophenyl (2,3,4,6-tetra-*O*-acetyl-β-D-galactopyranosyl)-(1→4)-2,3-di-*O*-acetyl-1-thio-β-D-glucopyranoside (17) ^{1}H-$^{13}C\{^{1}H\}$ HSQC NMR (400 & 101 MHz) in $CDCl_3$

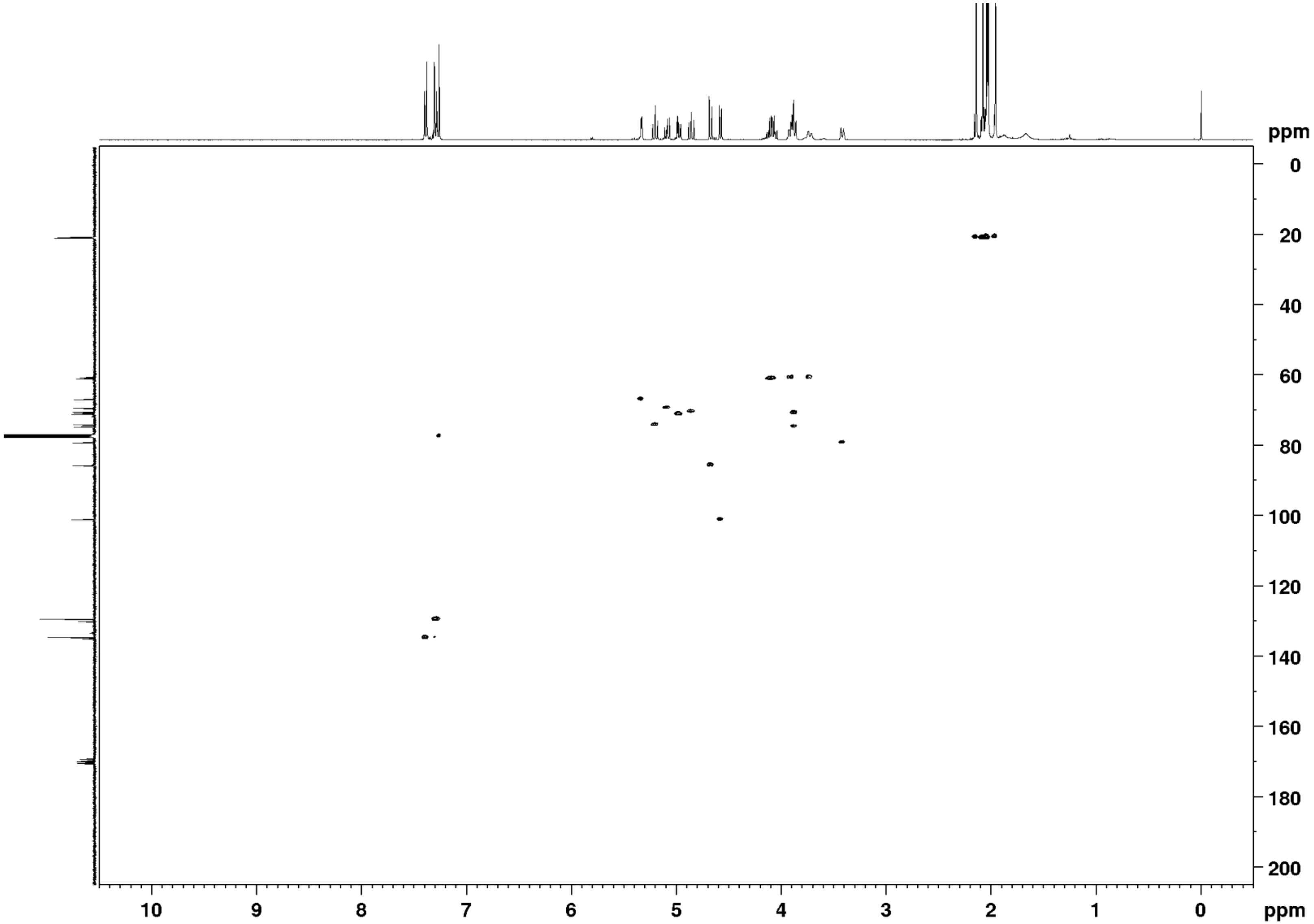

4-chlorophenyl (2,3,4,6-tetra-*O*-acetyl-β-D-galactopyranosyl)-(1→4)-2,3-di-*O*-acetyl-1-thio-β-D-glucopyranoside (17) ^{1}H-$^{13}C\{^{1}H\}$ HMBC NMR (400 & 101 MHz) in $CDCl_3$

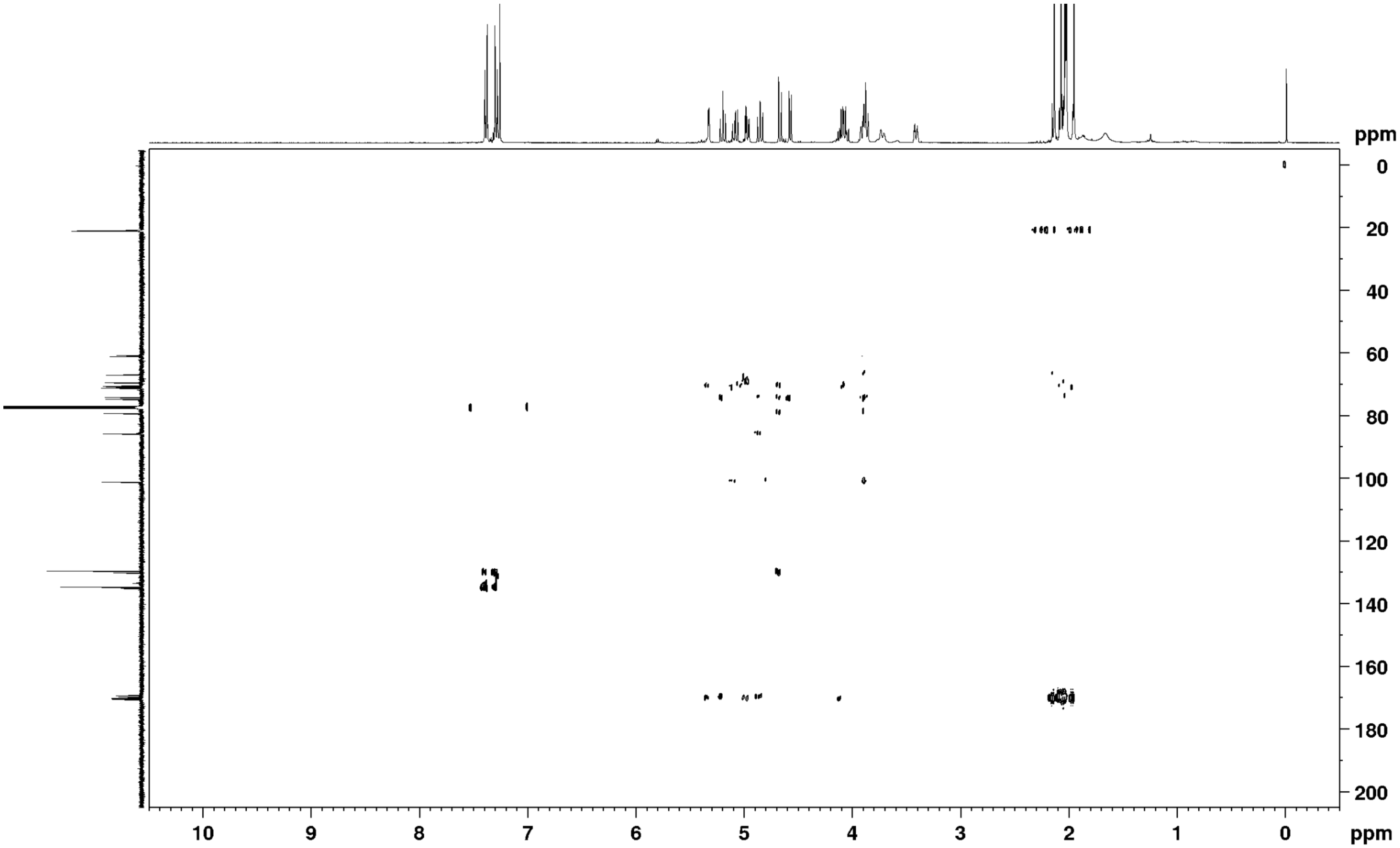

4-chlorophenyl (2,3,4,6-tetra-*O*-acetyl-β-D-galactopyranosyl)-(1→4)-2,3-di-*O*-acetyl-1-thio-β-D-glucopyranoside (**17**) $^{13}C\{^1H\}$ NMR (101 MHz) in $CDCl_3$

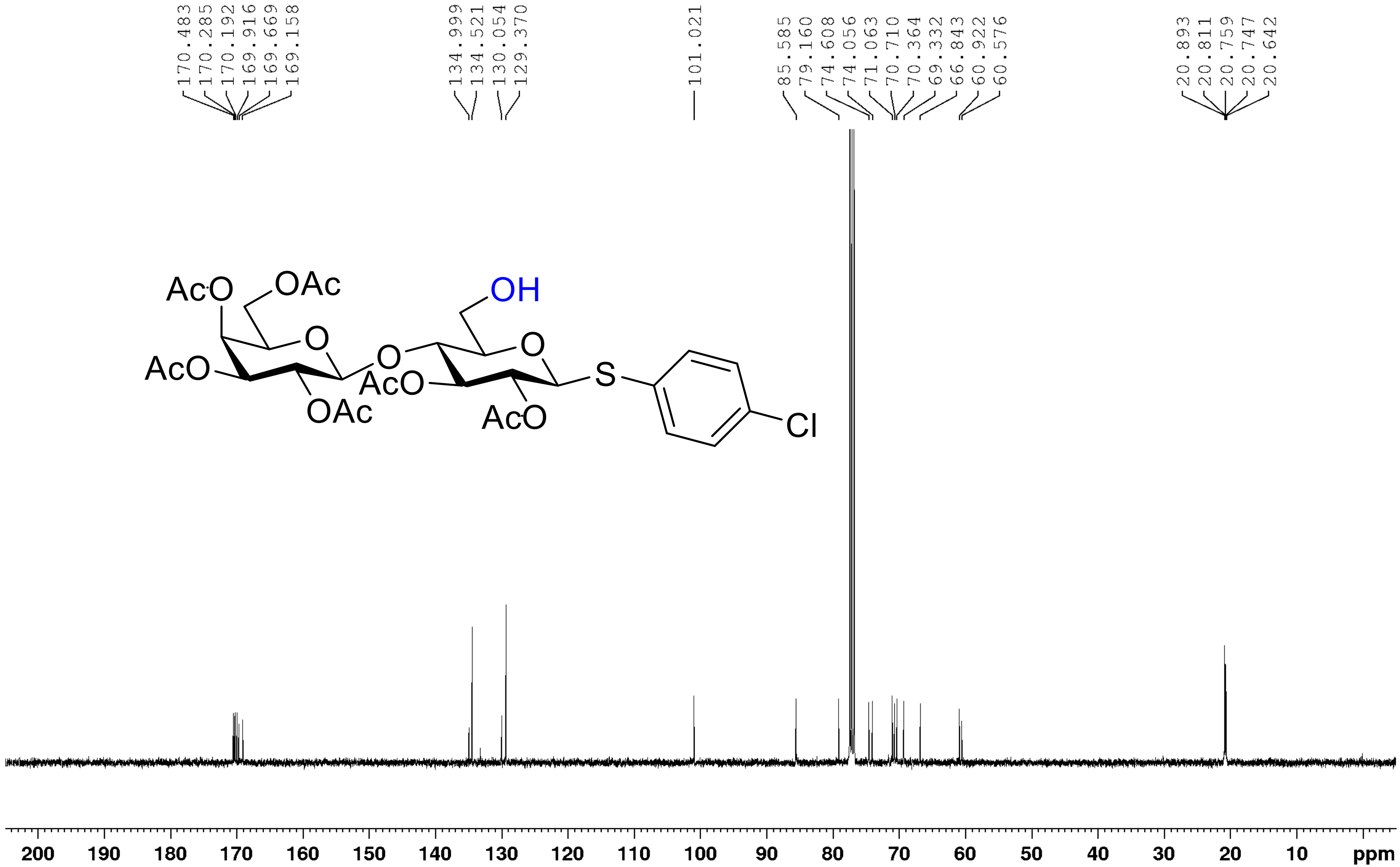

diphenyl 2,3,4,6-tetra-*O*-α-D-galactopyranosyl phosphate (**18**) ^{1}H NMR (400 MHz) in $CDCl_3$

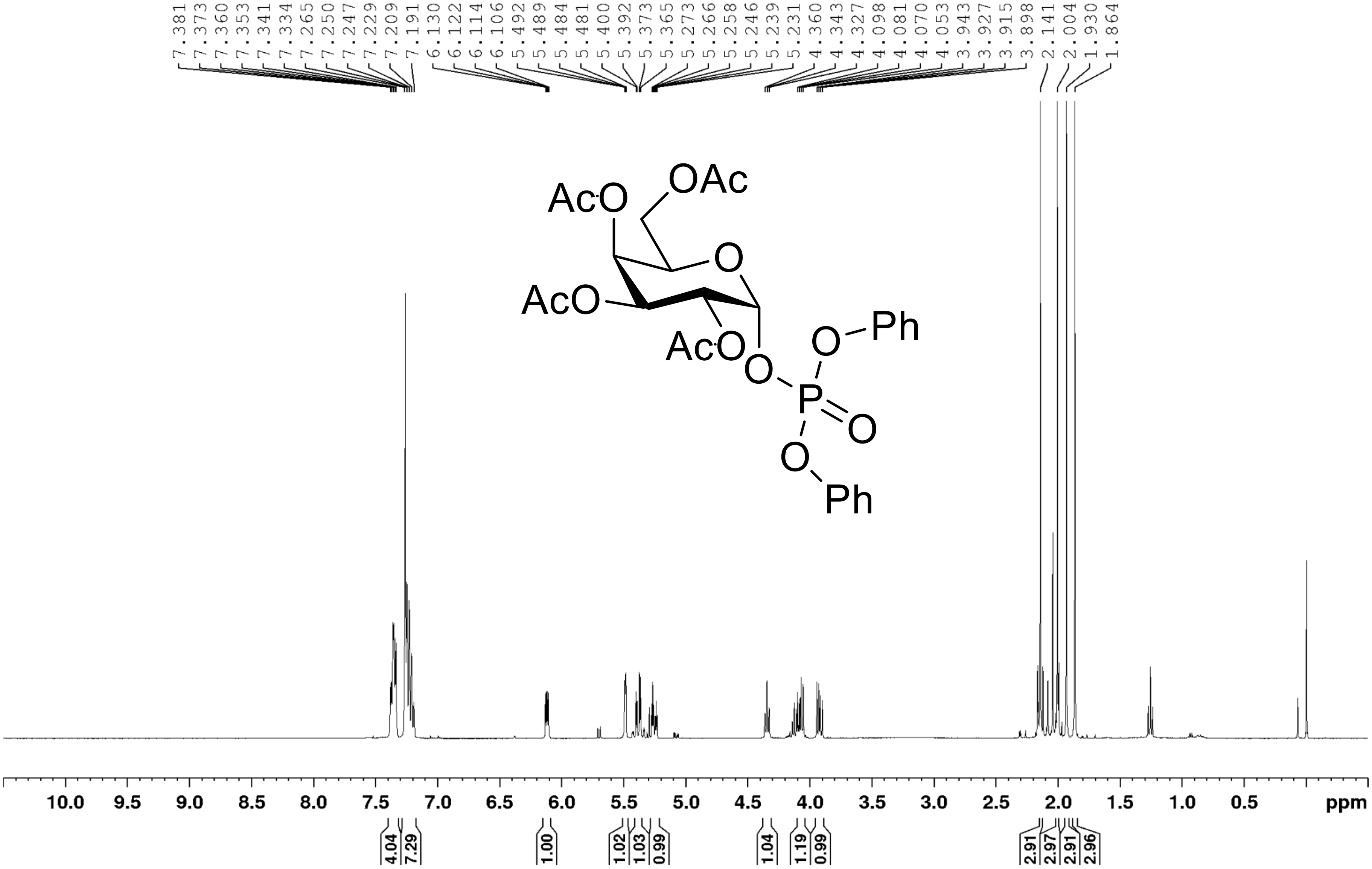

5,5-dimethyl 2,3,4,6-tetra-*O*-D-galactopyranosyl 1,3,2-dioxaphosphinane (19) α:β 1:2 ^{1}H NMR (400 MHz) in $CDCl_3$

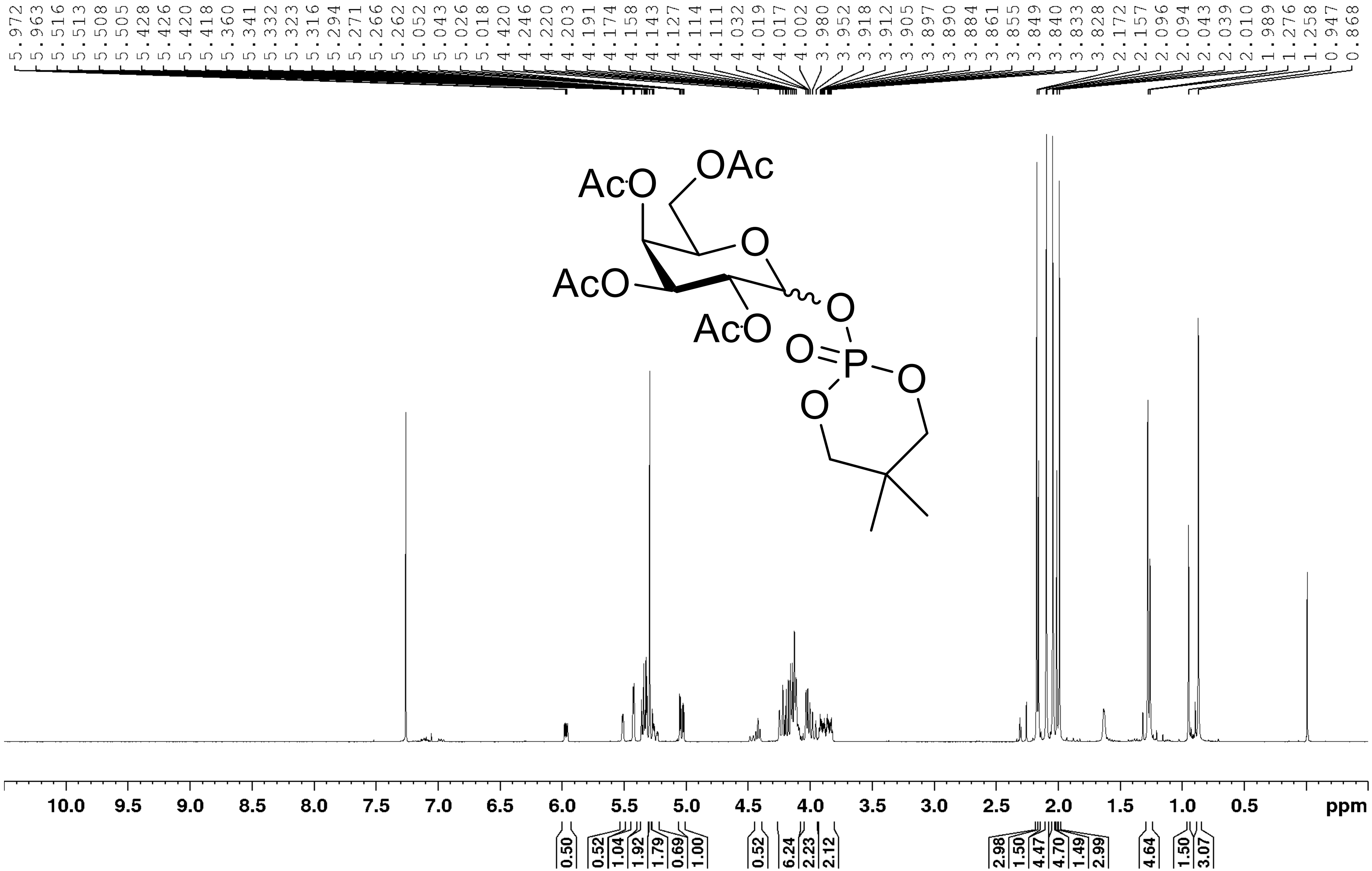

5,5-dimethyl 2,3,4,6-tetra-*O*-D-galactopyranosyl 1,3,2-dioxaphosphinane (**19**) α:β 1:2 ^{1}H-^{1}H COSY NMR (400 MHz) in $CDCl_3$

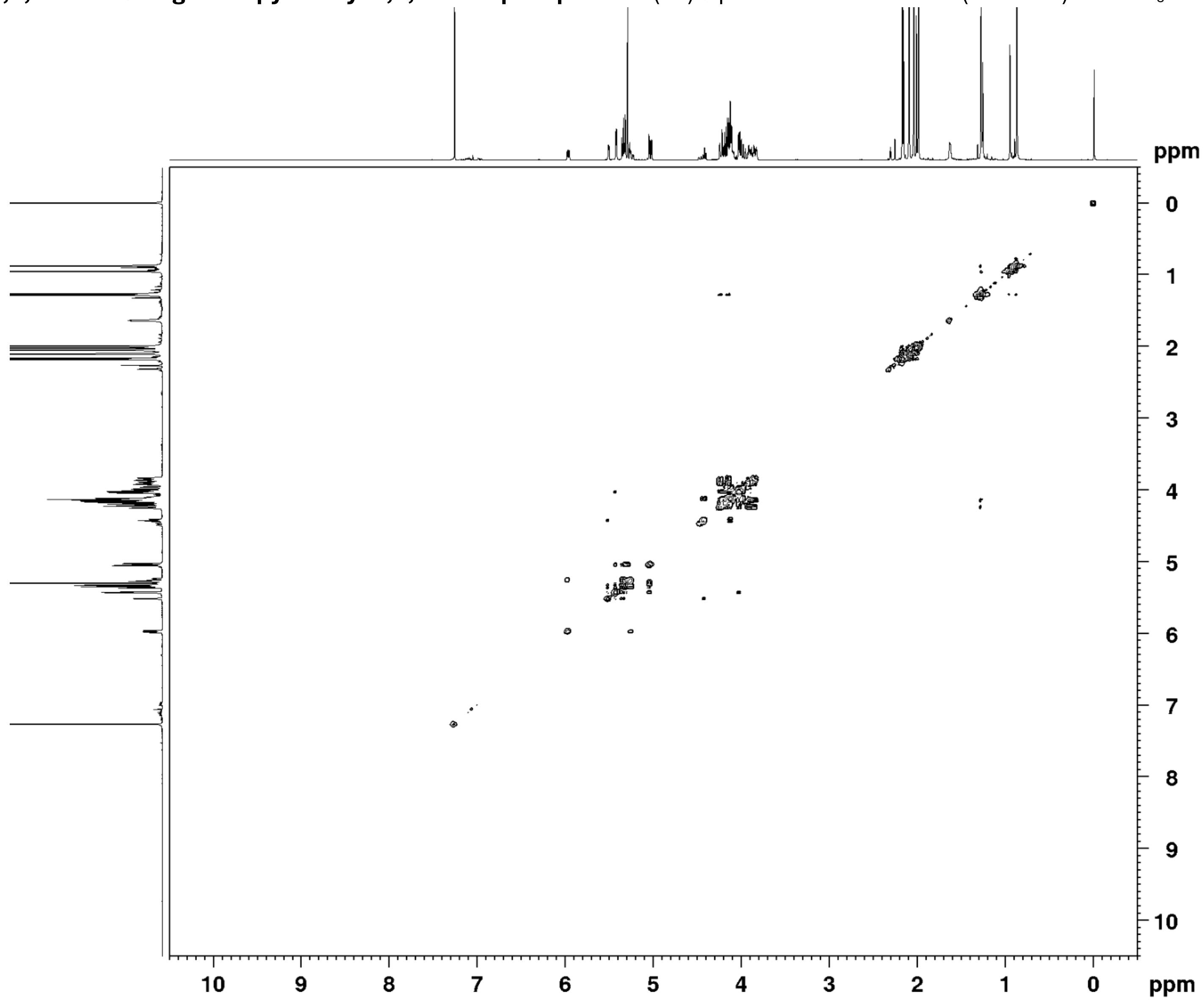

5,5-dimethyl 2,3,4,6-tetra-*O*-D-galactopyranosyl 1,3,2-dioxaphosphinane (**19**) α:β 1:2 ^{1}H-$^{13}C\{^{1}H\}$ HSQC NMR (400 & 101 MHz) in $CDCl_3$

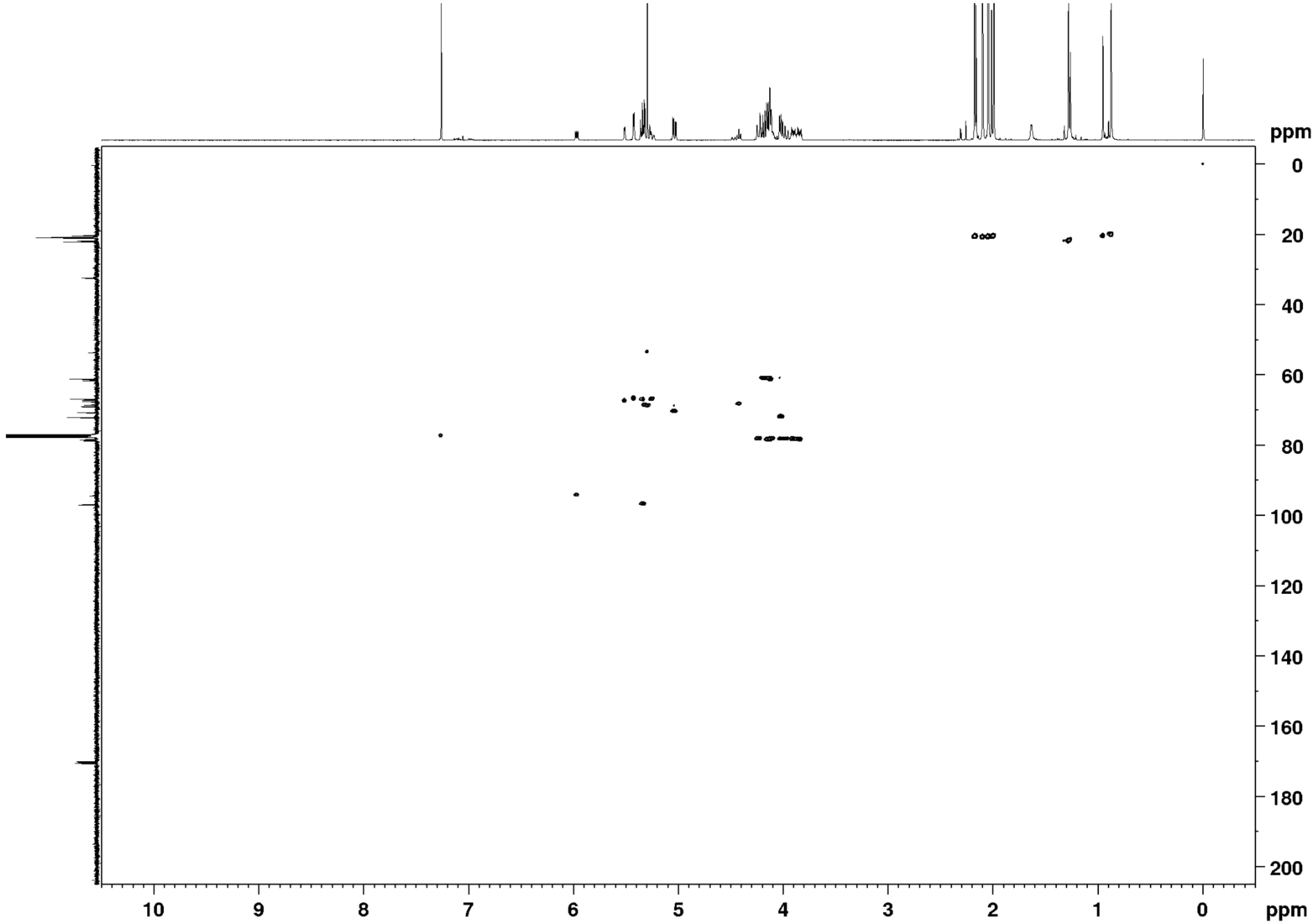

5,5-dimethyl 2,3,4,6-tetra-*O*-D-galactopyranosyl 1,3,2-dioxaphosphinane (**19**) α:β 1:2 ^{1}H-$^{13}C\{^{1}H\}$ HMBC NMR (400 & 101 MHz) in $CDCl_3$

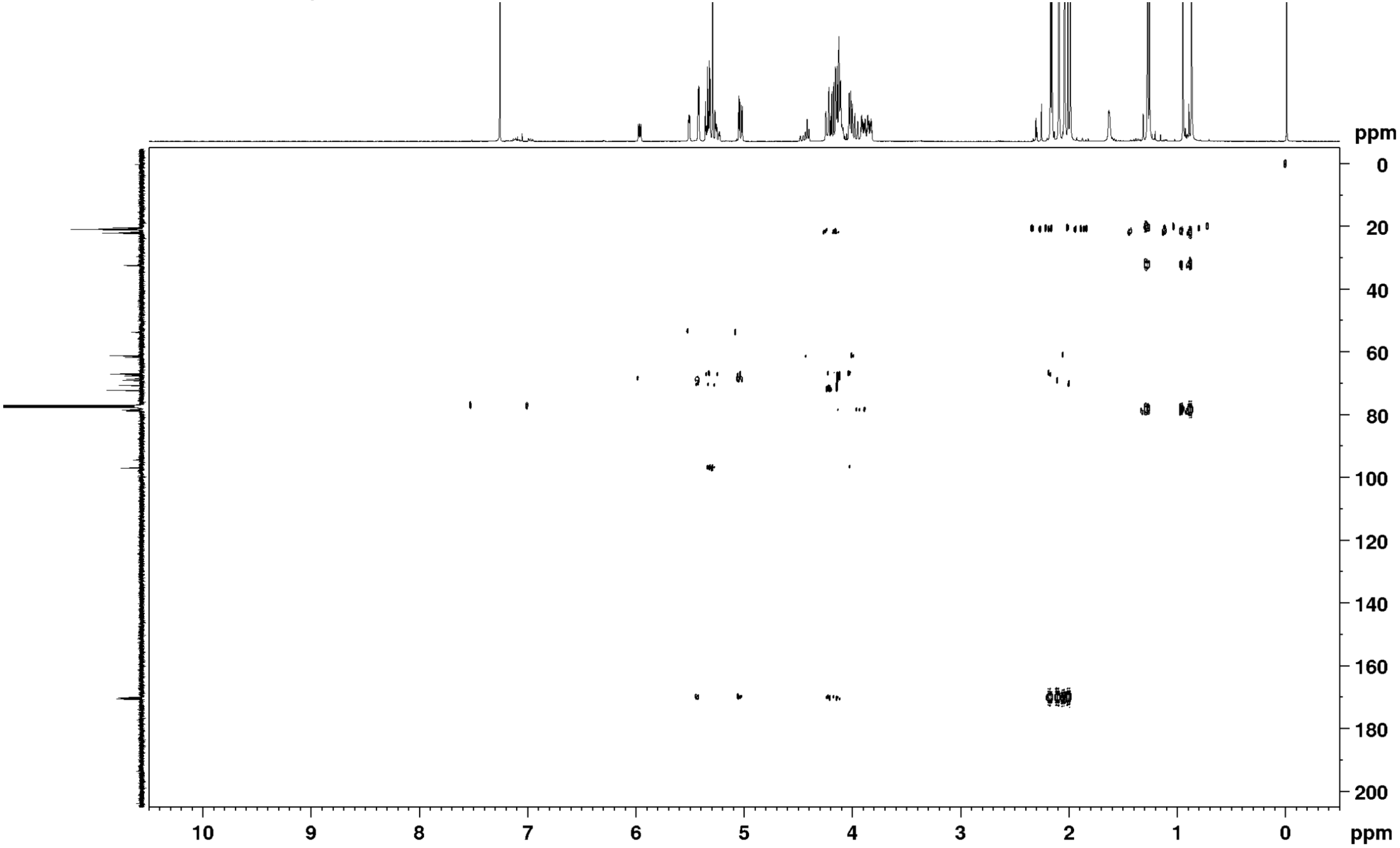

5,5-dimethyl 2,3,4,6-tetra-*O*-D-galactopyranosyl 1,3,2-dioxaphosphinane (19) α:β 1:2 $^{13}C\{^1H\}$ NMR (101 MHz) in $CDCl_3$

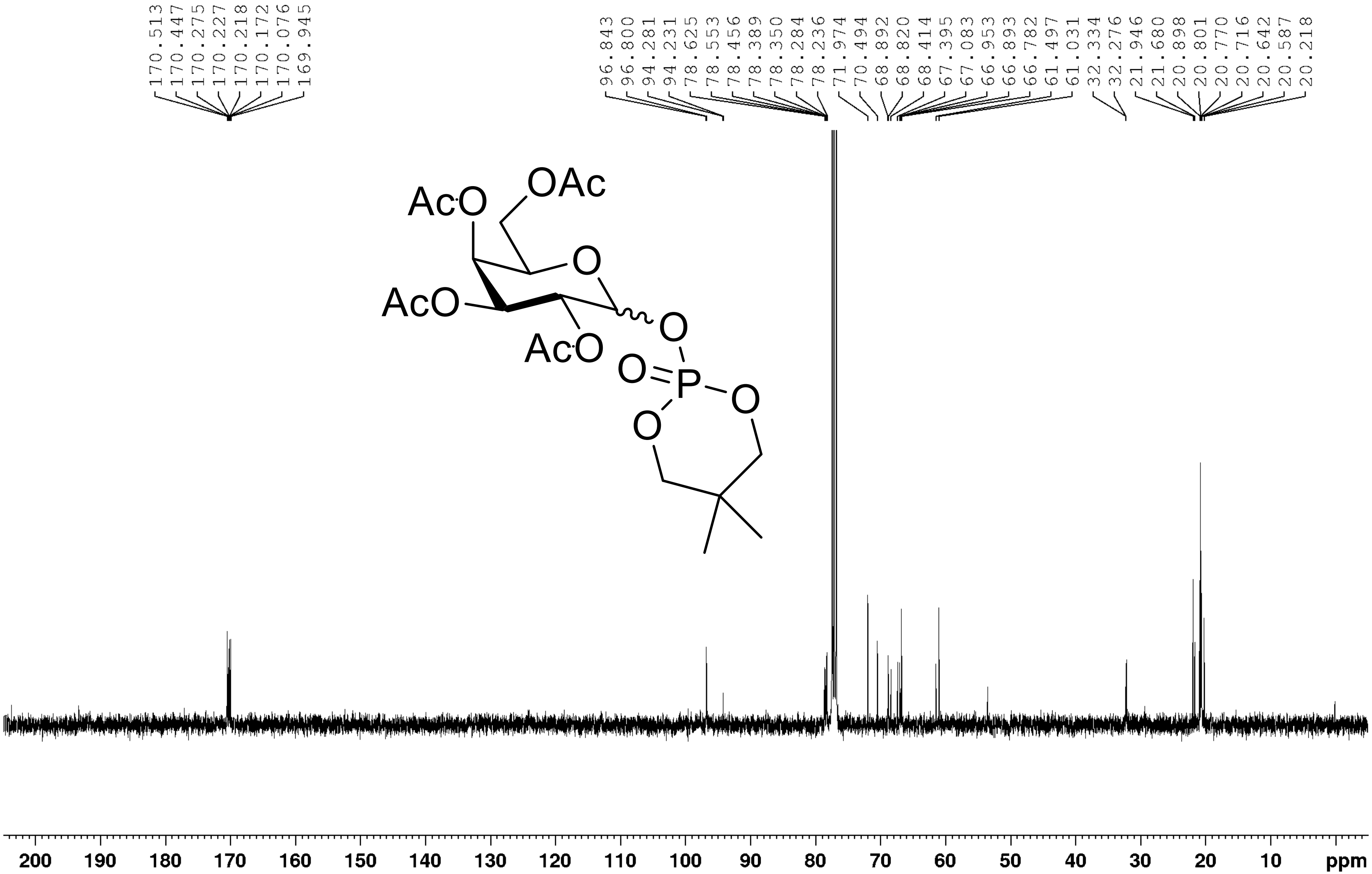

4-chlorophenyl 2,3,4,6-tetra-*O*-acetyl-1-thio-D-mannopyranoside (**21**) α:β 1:9 ^{1}H NMR (400 MHz) in $CDCl_3$

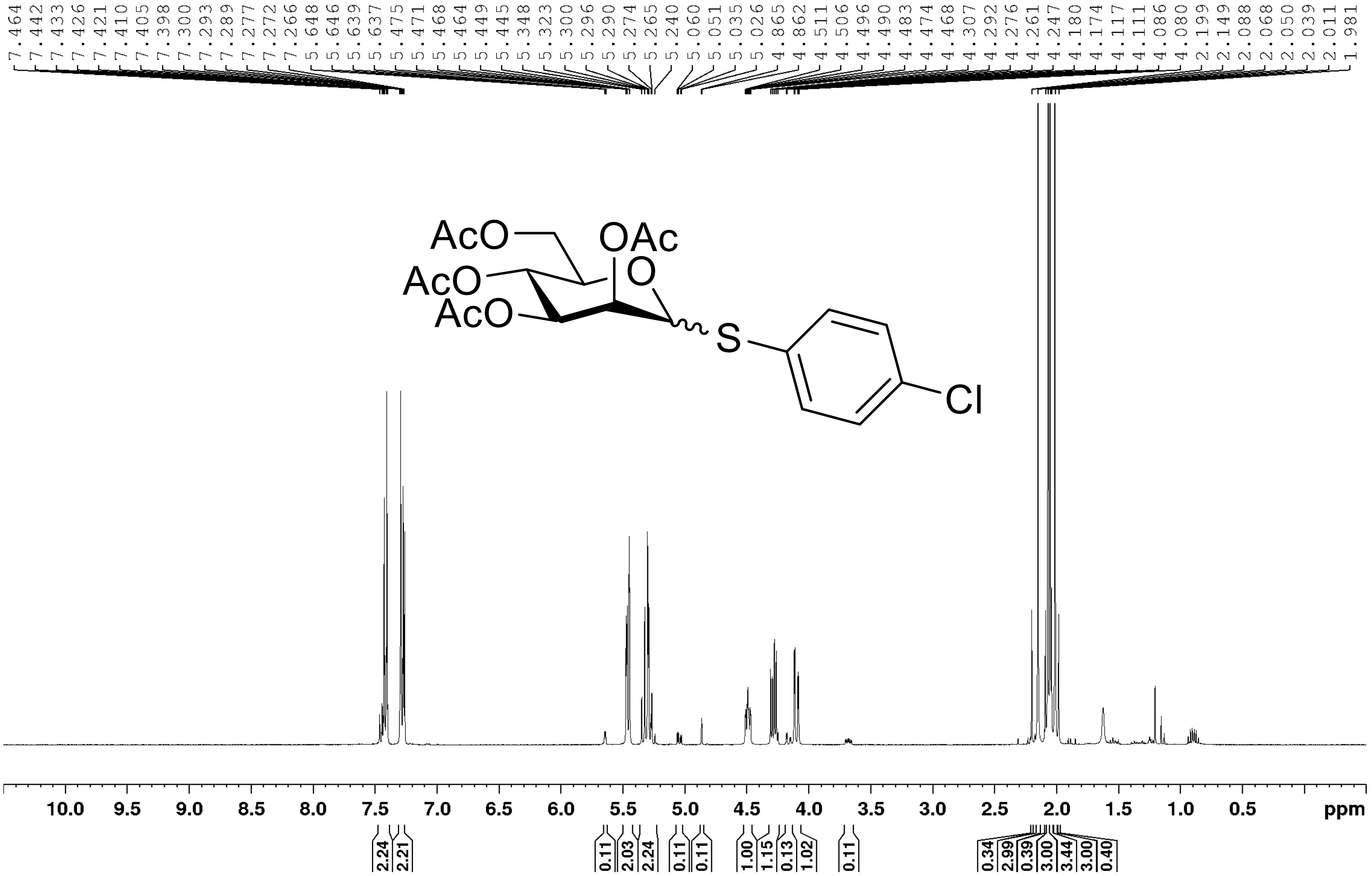

4-chlorophenyl 2,3-di-*O*-acetyl-1-thio-β-D-mannopyranoside (22) ^{1}H NMR (400 MHz) in $CDCl_3$

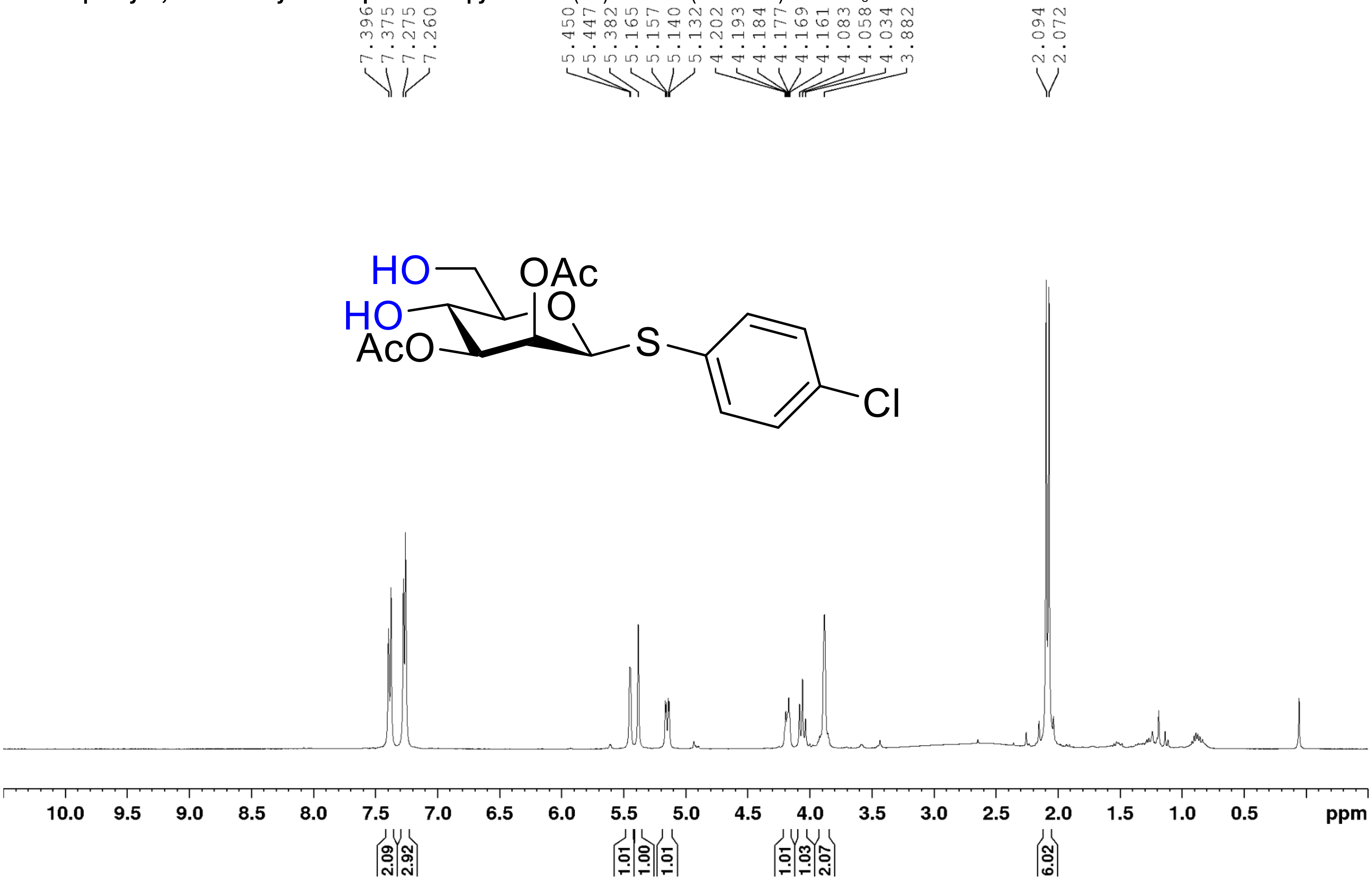

4-chlorophenyl 2,3-di-*O*-acetyl-1-thio-β-D-mannopyranoside (**22**) ^{1}H-^{1}H COSY NMR (400 MHz) in $CDCl_3$

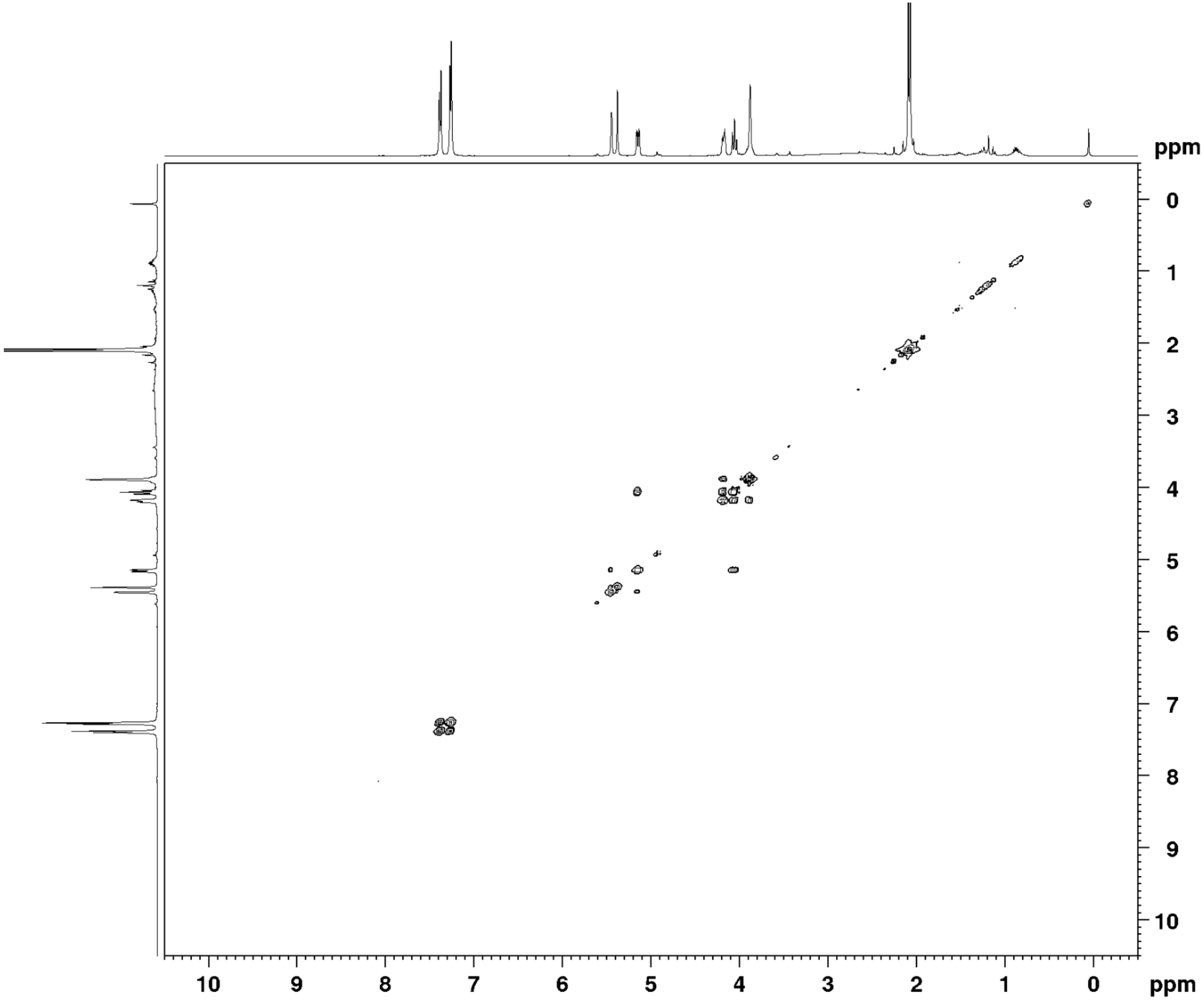

4-chlorophenyl 2,3-di-*O*-acetyl-1-thio-β-D-mannopyranoside (**22**) ^{1}H-$^{13}C\{^{1}H\}$ HSQC NMR (400 & 101 MHz) in $CDCl_3$

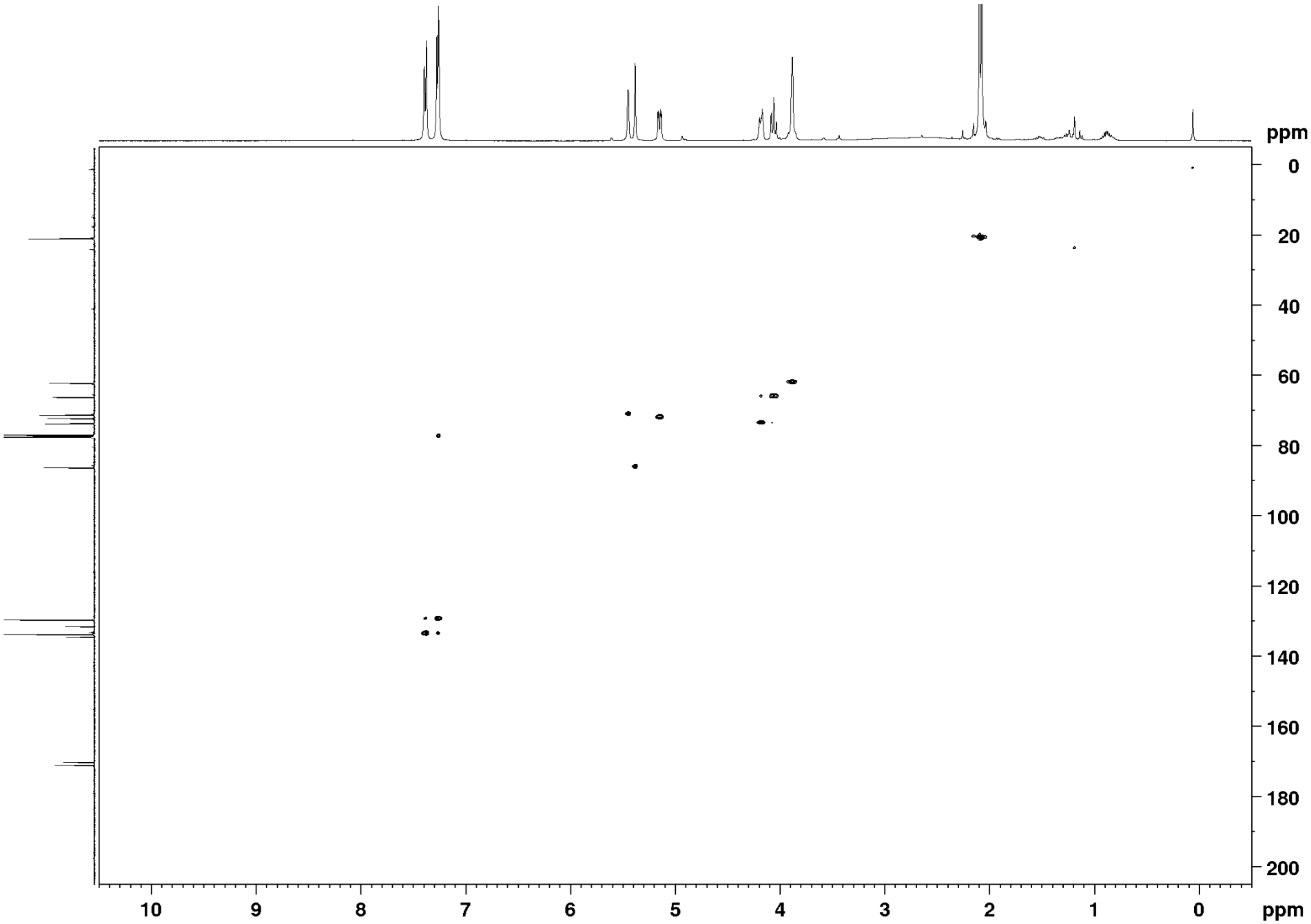

4-chlorophenyl 2,3-di-*O*-acetyl-1-thio-β-D-mannopyranoside (**22**) ^{1}H-$^{13}C\{^{1}H\}$ HMBC NMR (400 & 101 MHz) in $CDCl_3$

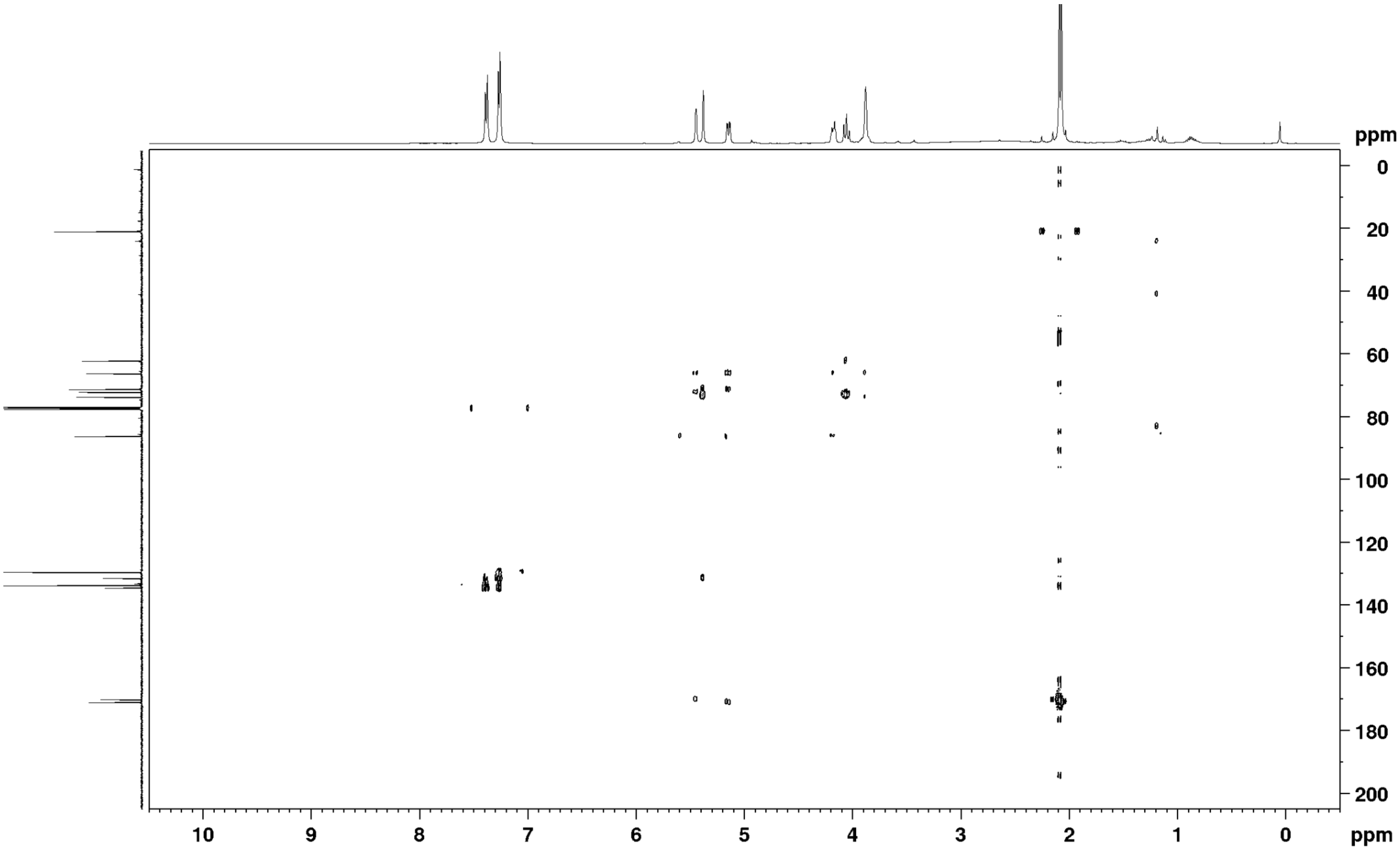

4-chlorophenyl 2,3-di-*O*-acetyl-1-thio-β-D-mannopyranoside (**22**) $^{13}C\{^1H\}$ NMR (101 MHz) in $CDCl_3$

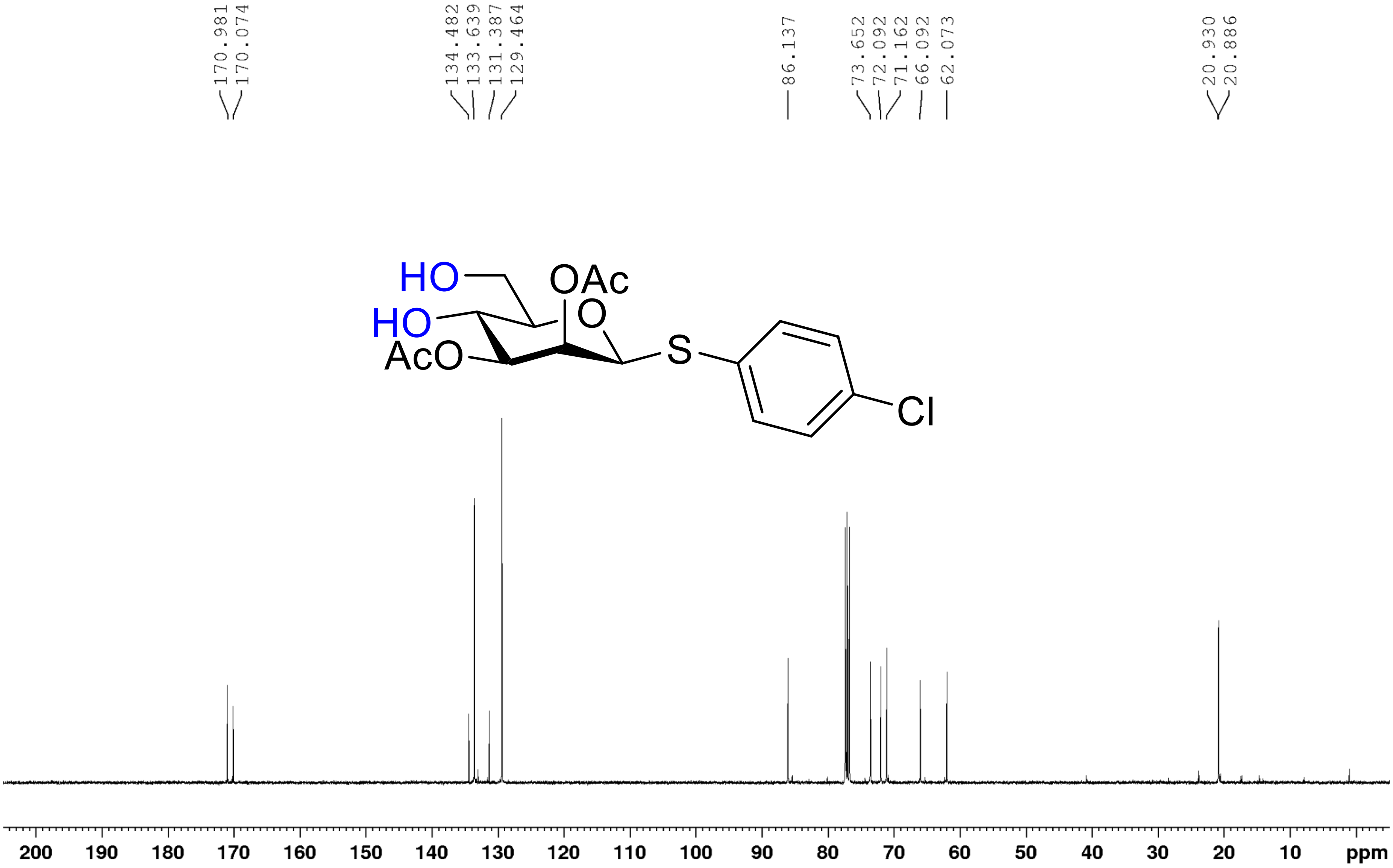

1,2,3,4,6-penta-*O*-acetyl-L-glucopyranoside (**23**) α:β 3:2 ^{1}H NMR (400 MHz) in $CDCl_3$

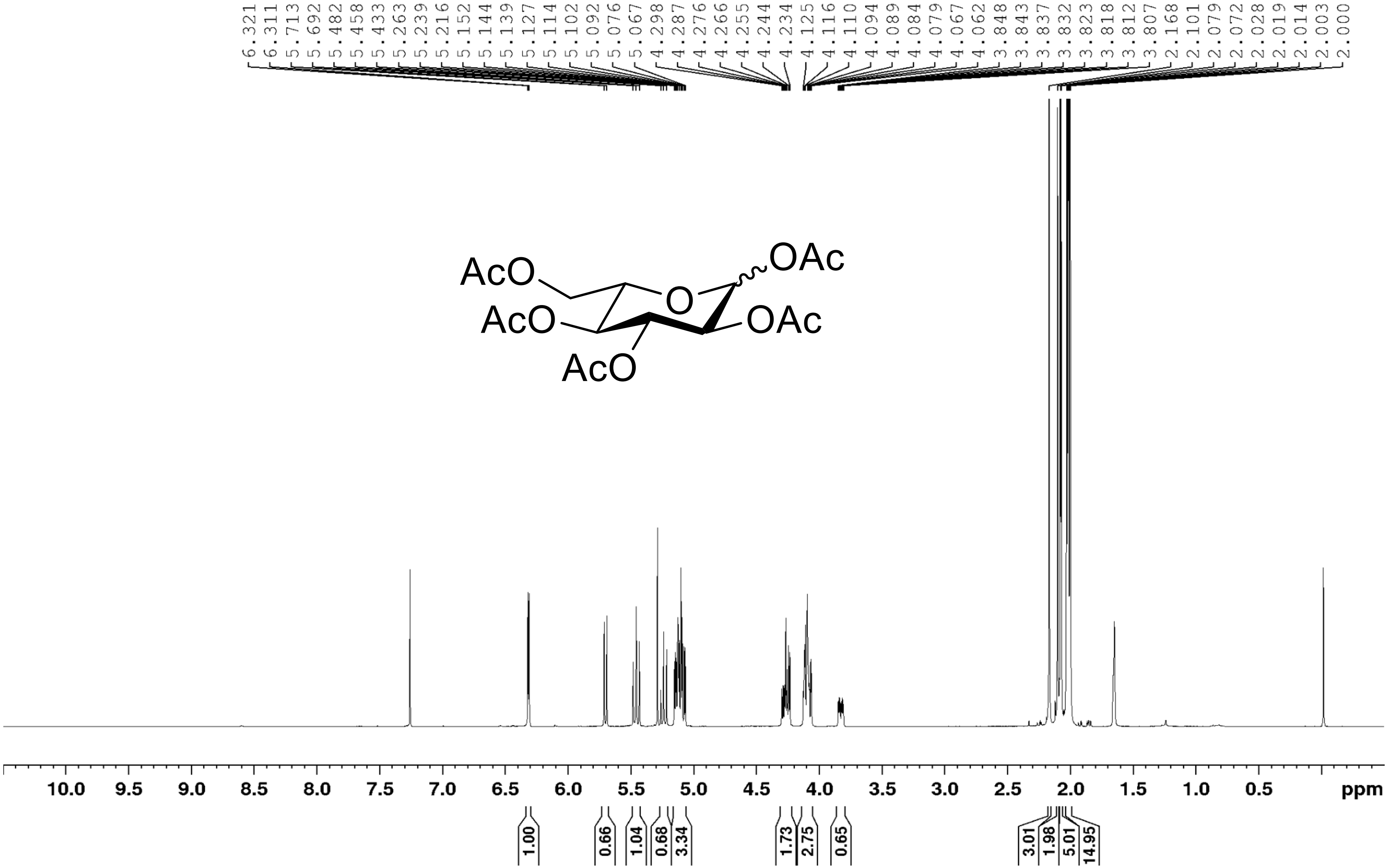

1,2,3,4,6-penta-*O*-acetyl-L-glucopyranoside (**23**) α:β 3:2 ^{1}H-^{1}H COSY NMR (400 MHz) in $CDCl_3$

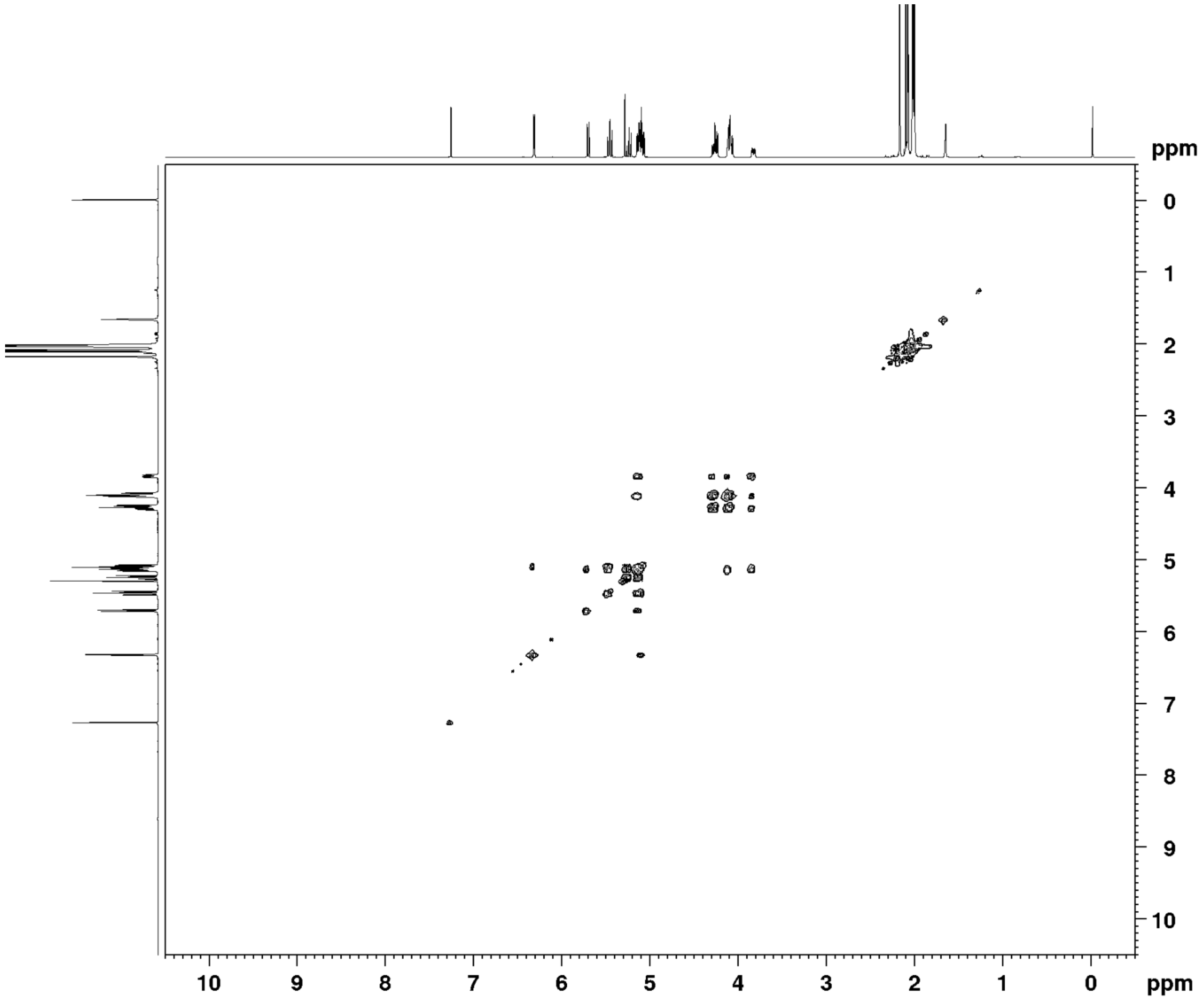

1,2,3,4,6-penta-*O*-acetyl-L-glucopyranoside (**23**) α:β 3:2 ^{1}H-^{13}C{^{1}H} HSQC NMR (400 & 101 MHz) in $CDCl_3$

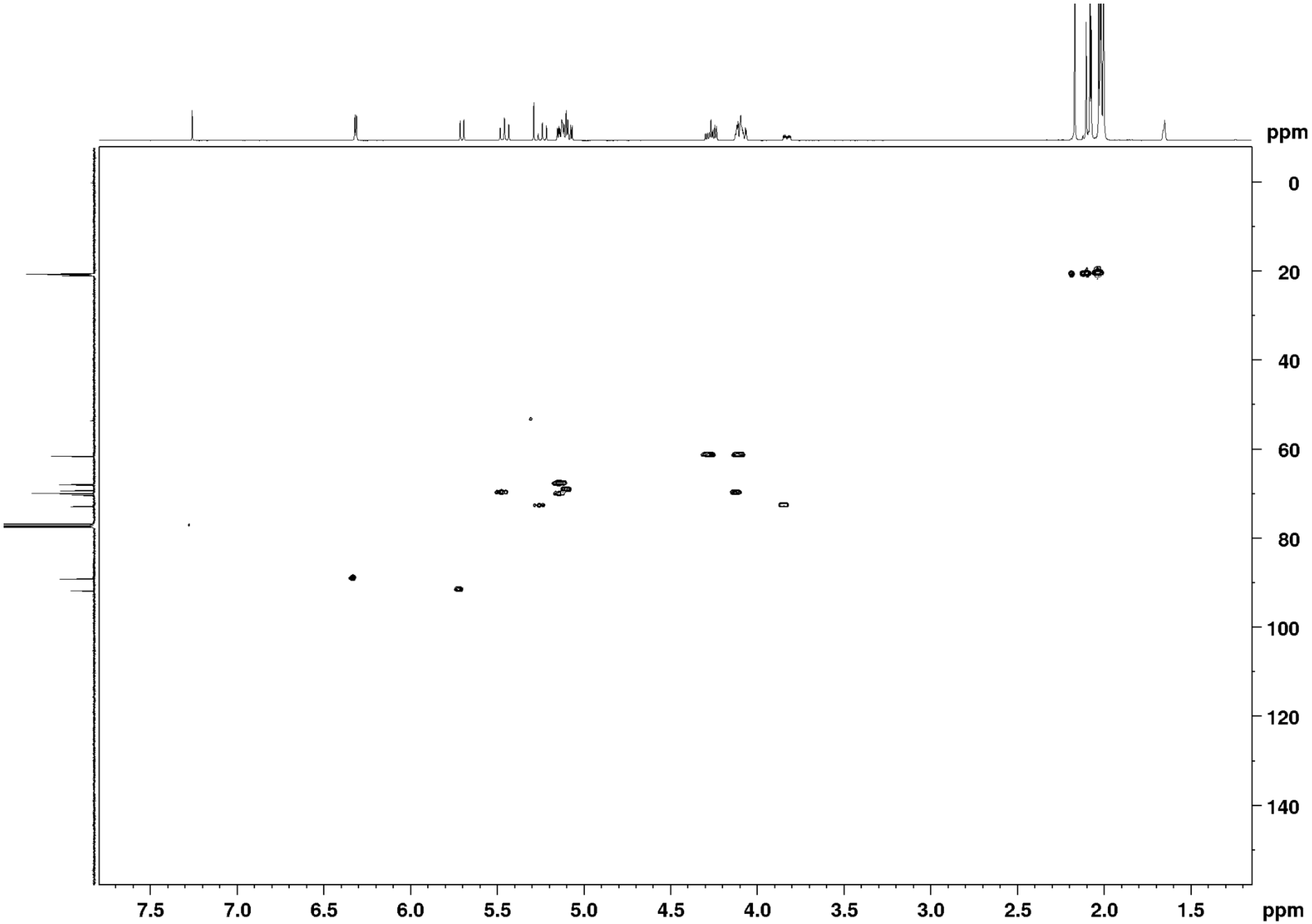

1,2,3,4,6-penta-*O*-acetyl-L-glucopyranoside (**23**) α:β 3:2 ^{1}H-$^{13}C\{^{1}H\}$ HMBC NMR (400 & 101 MHz) in $CDCl_3$

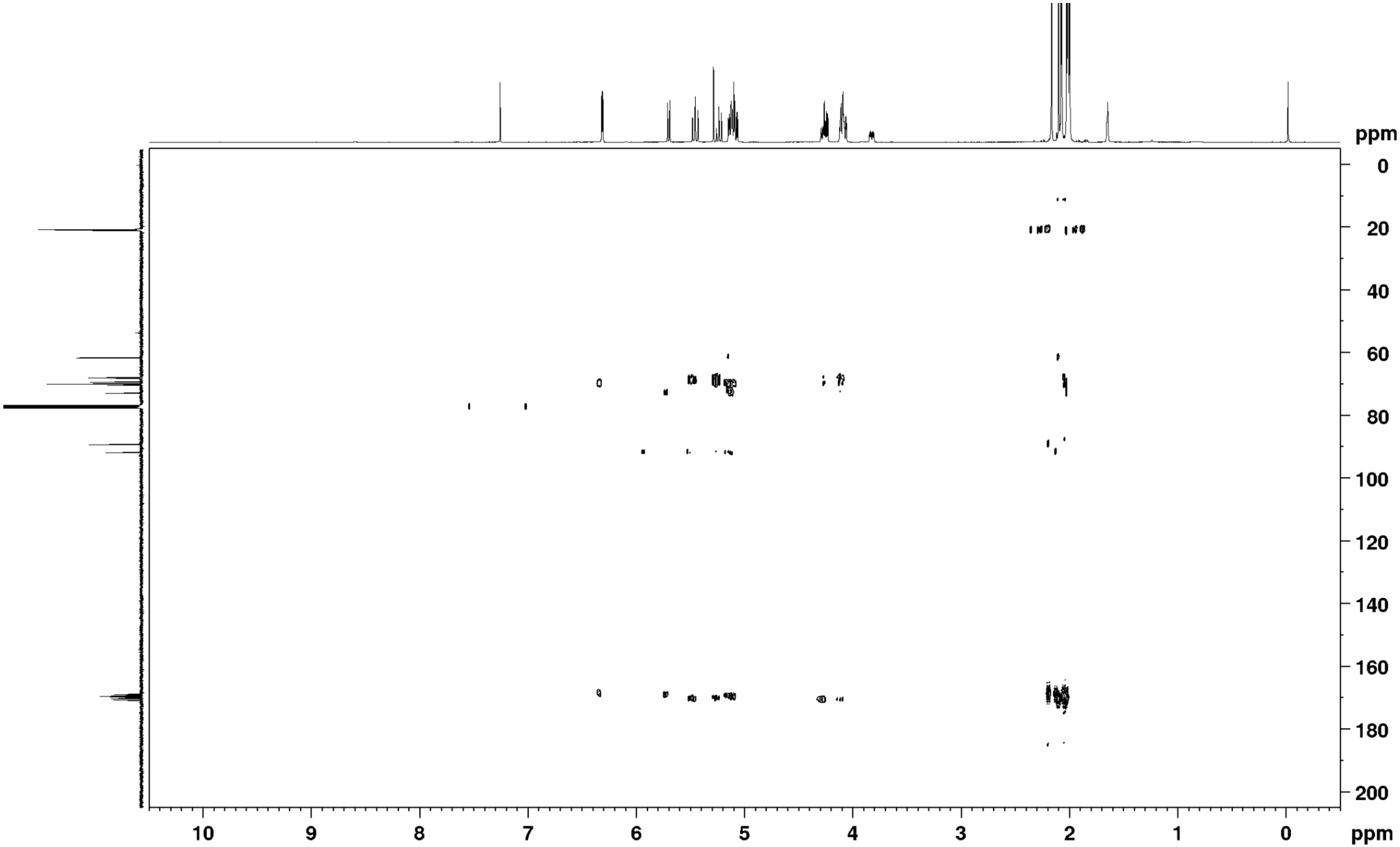

1,2,3,4,6-penta-*O*-acetyl-L-glucopyranoside (**23**) α:β 9:1 ^{1}H NMR (400 MHz) in $CDCl_3$

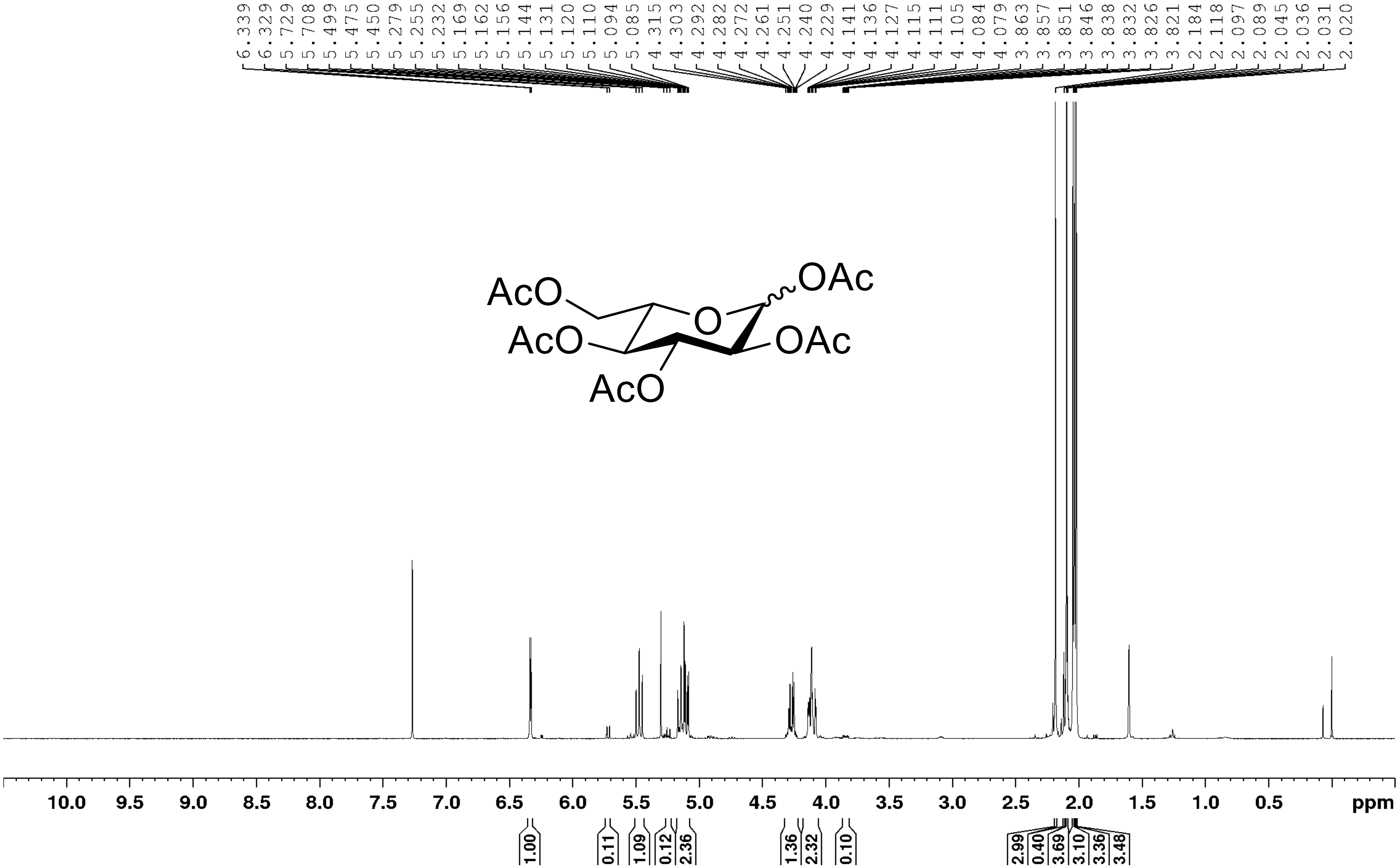

1,2,3,4-tetra-*O*-acetyl-L-glucopyranoside (**24**) α:β 2:1 ^{1}H NMR (400 MHz) in $CDCl_3$

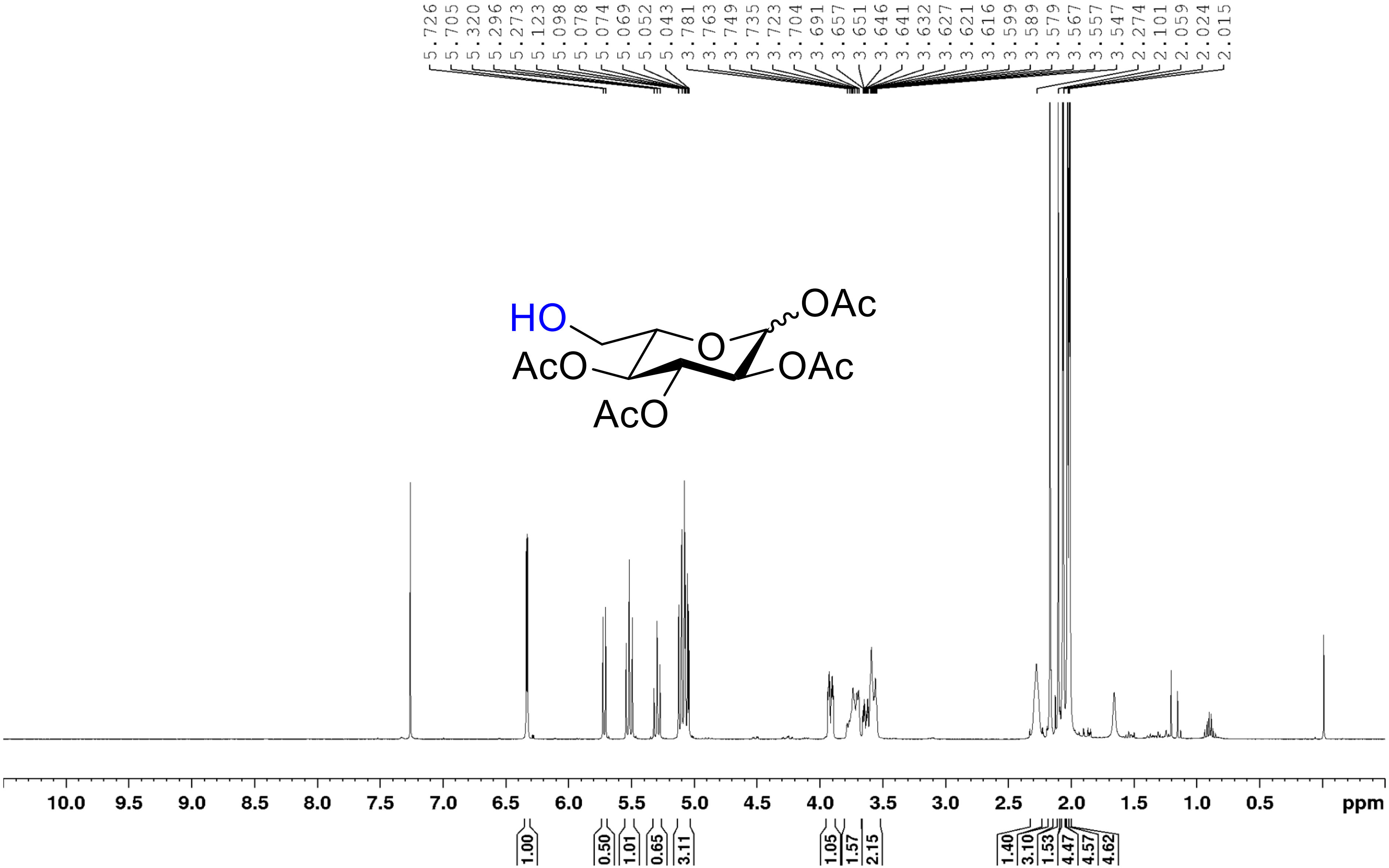

1,2,3,4-tetra-*O*-acetyl-L-glucopyranoside (**24**) α:β 3:2 ^{1}H-^{1}H COSY NMR (400 MHz) in $CDCl_3$

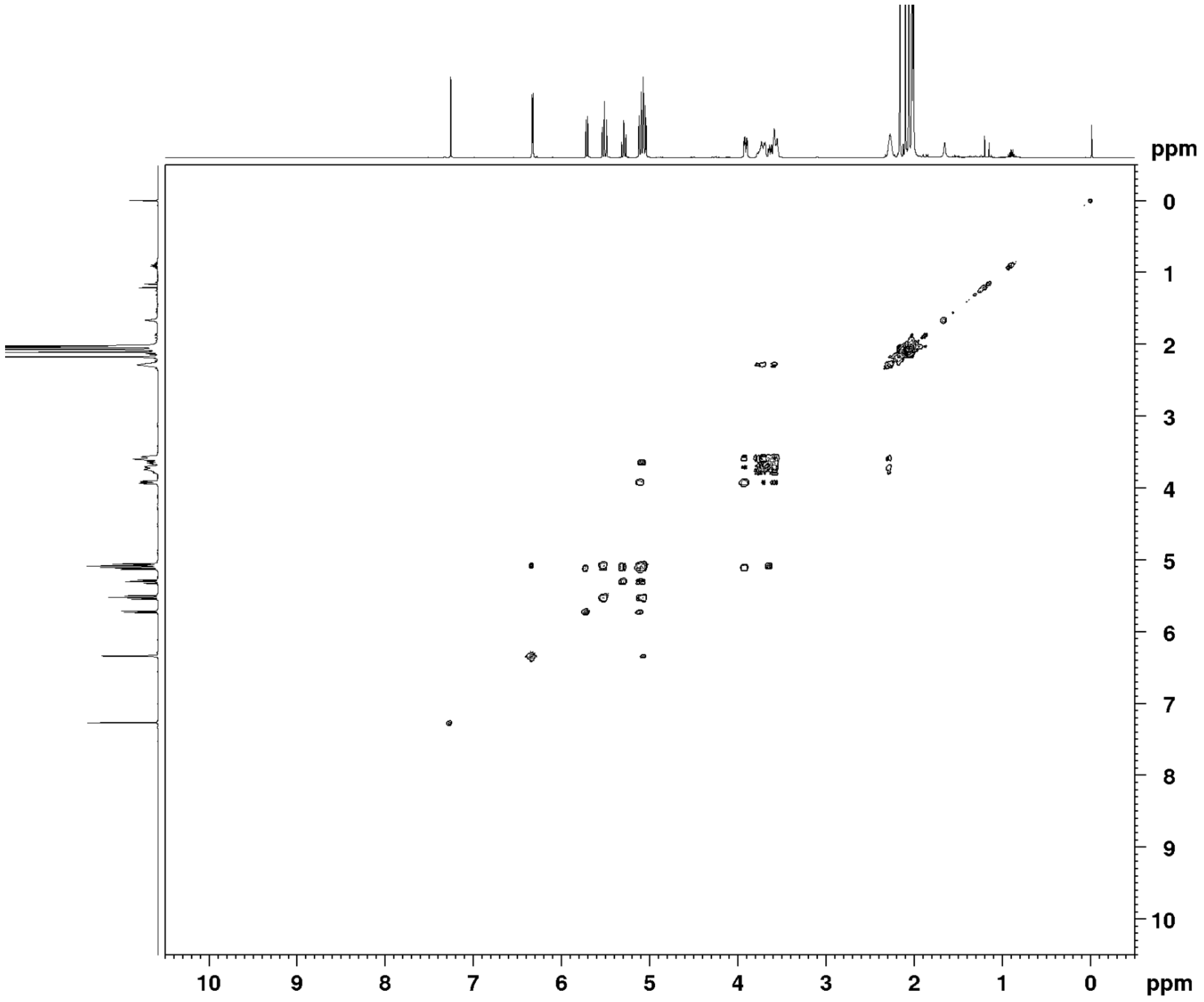

1,2,3,4-tetra-*O*-acetyl-L-glucopyranoside (**24**) α:β 3:2 ^{1}H-$^{13}C\{^{1}H\}$ HSQC NMR (400 & 101 MHz) in $CDCl_3$

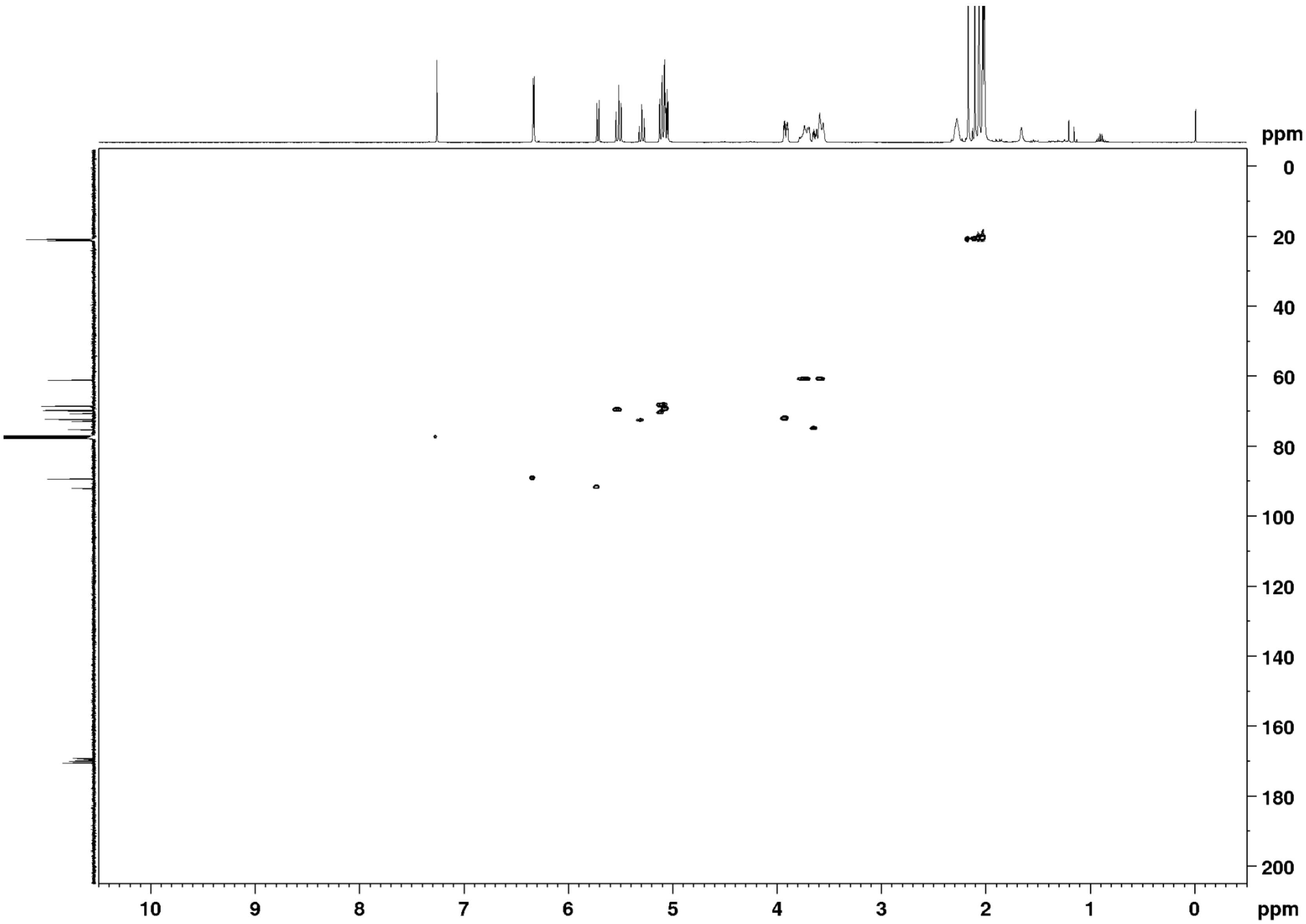

1,2,3,4-tetra-*O*-acetyl-L-glucopyranoside (**24**) α:β 3:2 ^{1}H-$^{13}C\{^{1}H\}$ HMBC NMR (400 & 101 MHz) in $CDCl_3$

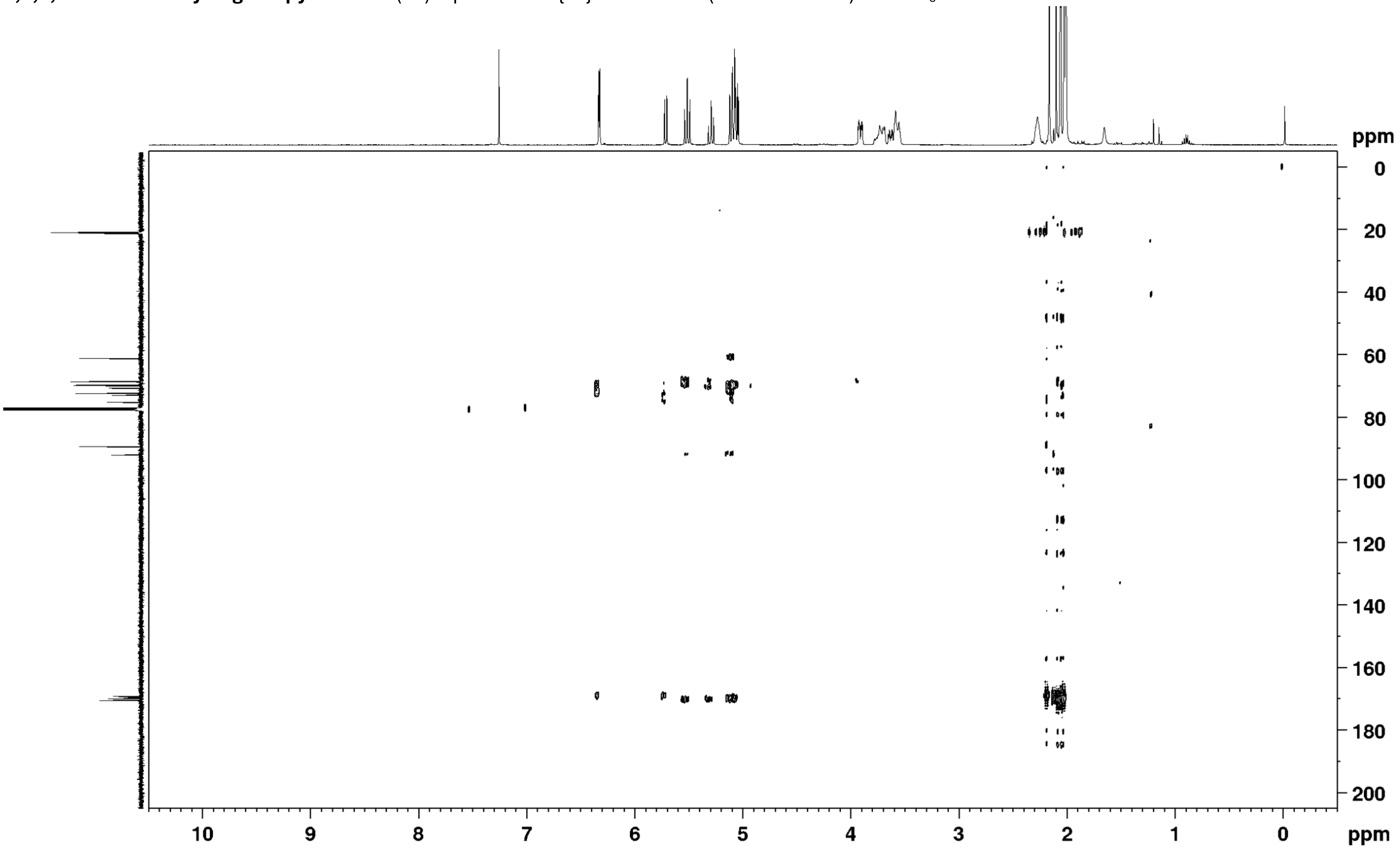

1,2,3,4-tetra-*O*-acetyl-L-glucopyranoside (**24**) α:β 2:1 $^{13}C\{^{1}H\}$ NMR (101 MHz) in $CDCl_3$

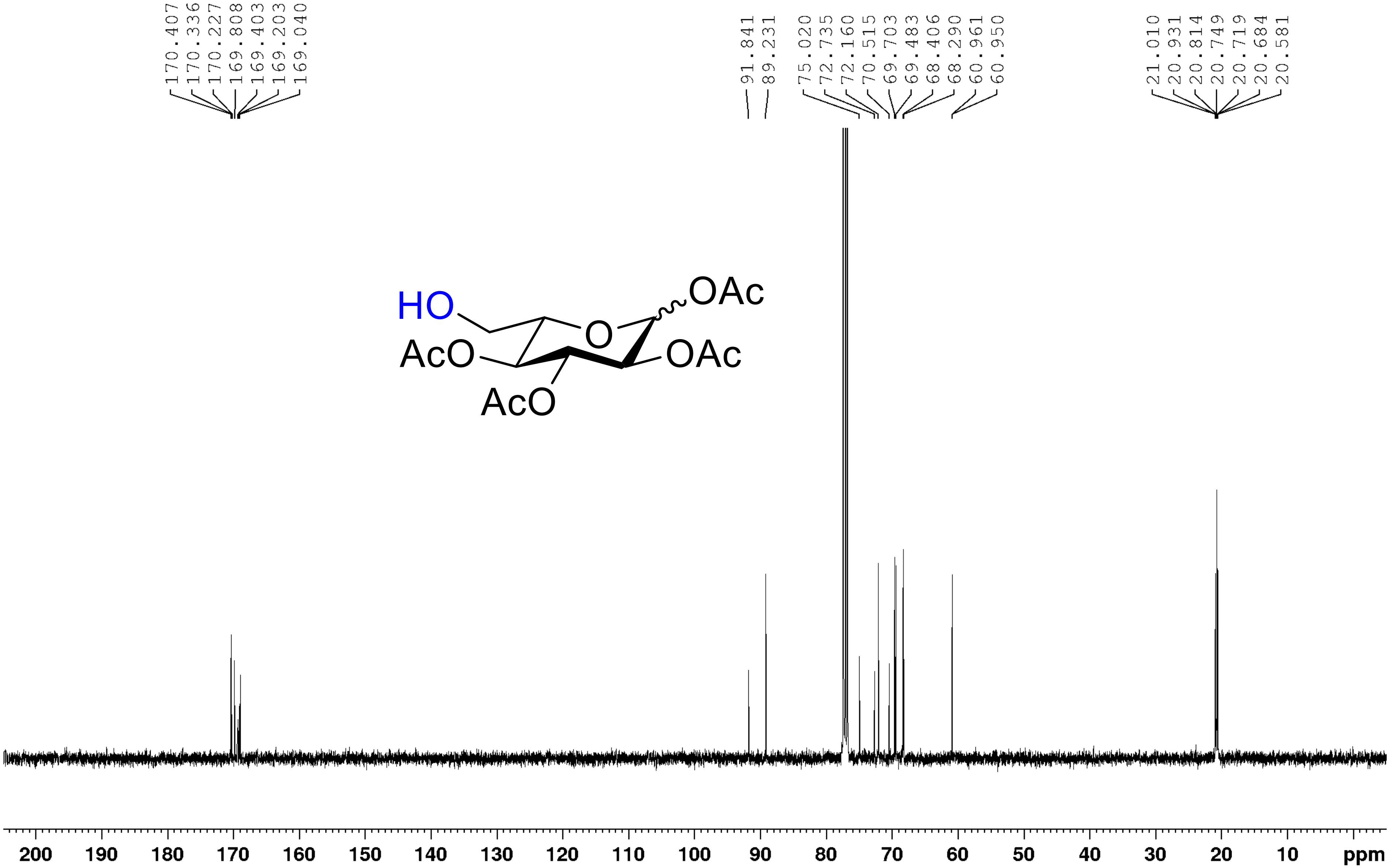

4-chlorophenyl 2,3,4,6-tetra-*O*-acetyl-1-thio-L-glucopyranoside (25) α:β 7:93 ^{1}H NMR (400 MHz) in $CDCl_3$

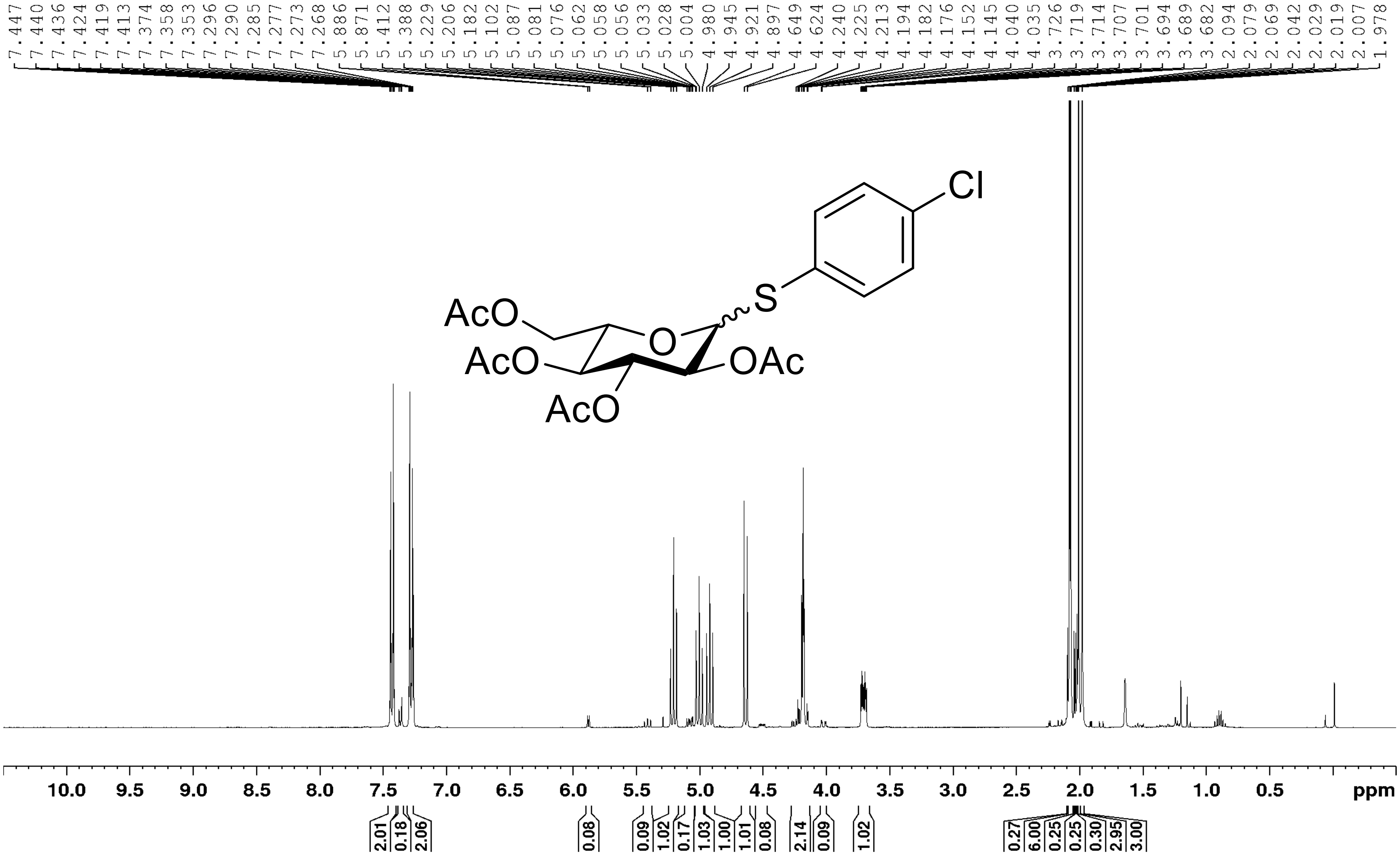

4-chlorophenyl 2,3,4,6-tetra-*O*-acetyl-1-thio-L-glucopyranoside (**25**) α:β 7:93 ^{1}H-^{1}H COSY NMR (400 MHz) in $CDCl_3$

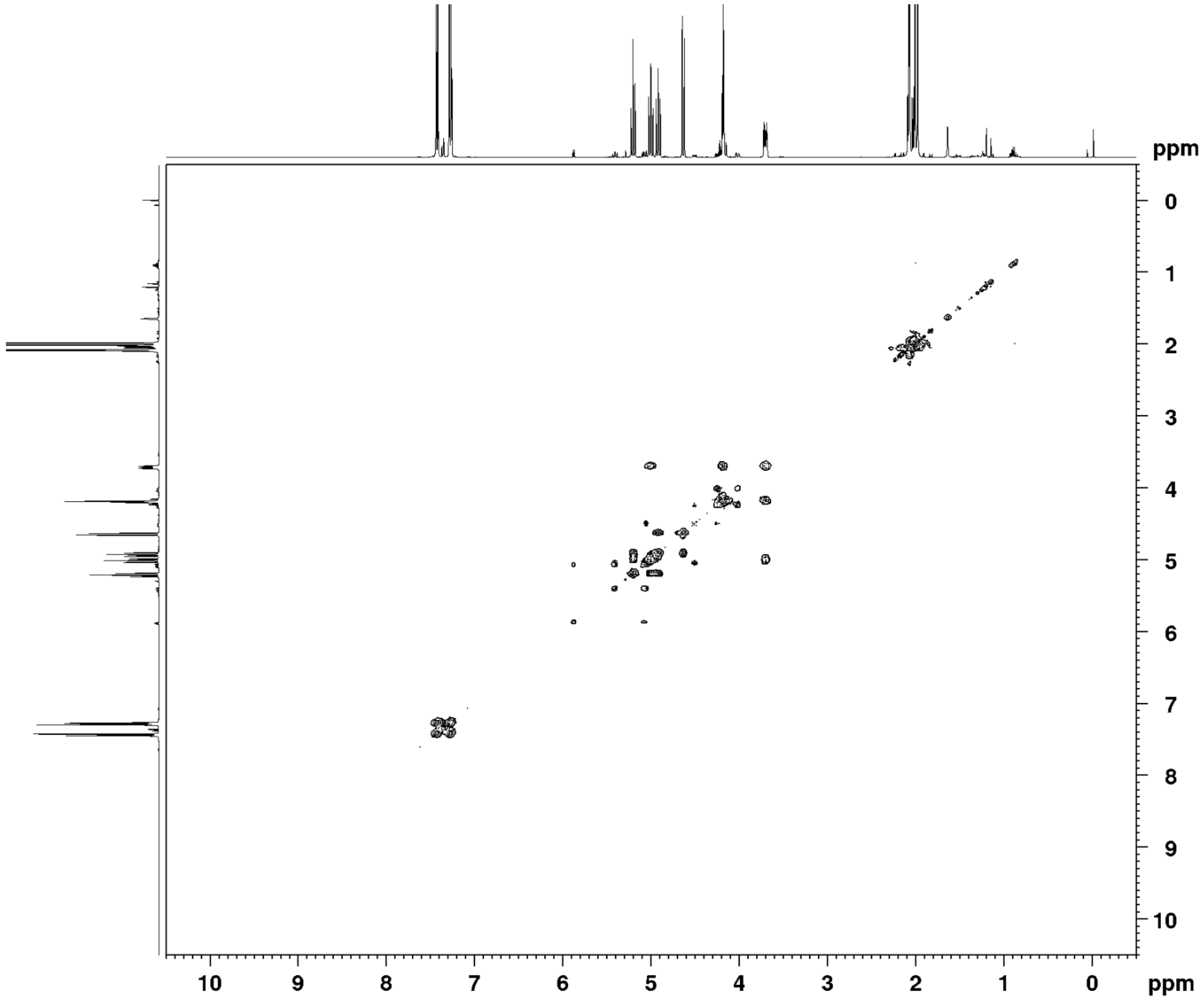

4-chlorophenyl 2,3,4,6-tetra-*O*-acetyl-1-thio-L-glucopyranoside (**25**) α:β 7:93 ^{1}H-$^{13}C\{^{1}H\}$ HSQC NMR (400 & 101 MHz) in $CDCl_3$

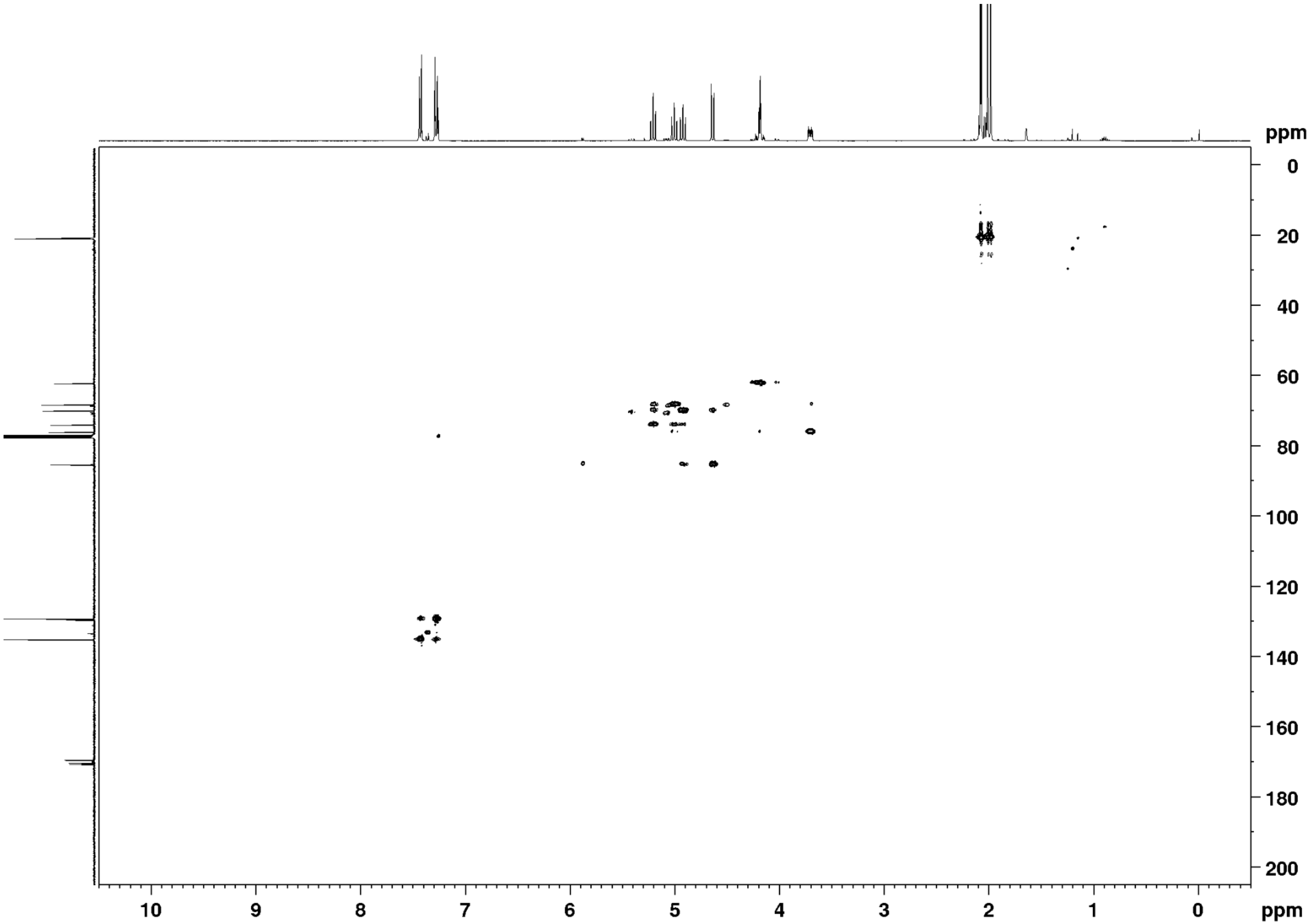

4-chlorophenyl 2,3,4,6-tetra-*O*-acetyl-1-thio-L-glucopyranoside (**25**) α:β 7:93 ^{1}H-$^{13}C\{^{1}H\}$ HMBC NMR (400 & 101 MHz) in $CDCl_3$

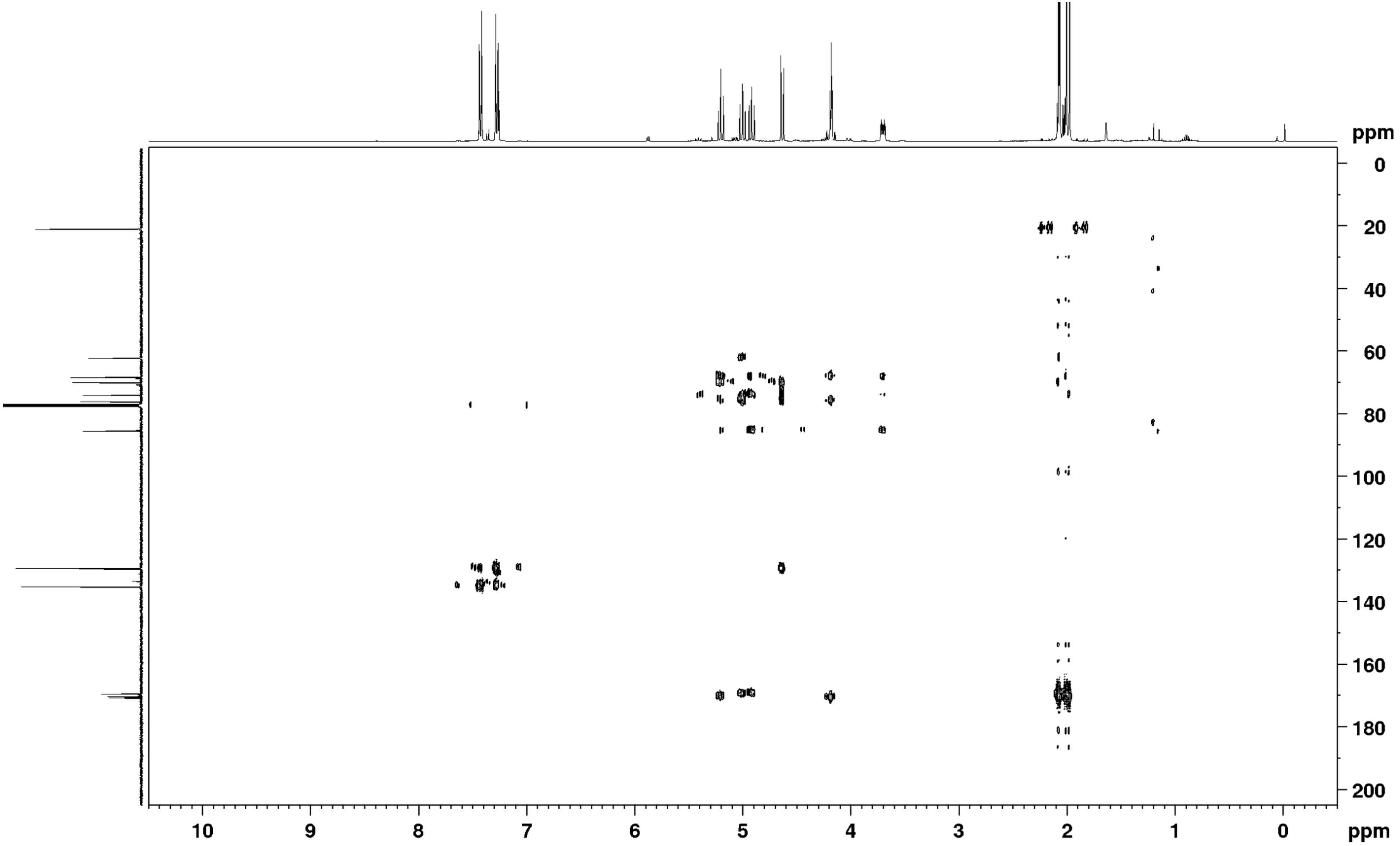

4-chlorophenyl 2,3,4,6-tetra-*O*-acetyl-1-thio-L-glucopyranoside (**25**) α:β 7:93 $^{13}C\{^1H\}$ NMR (101 MHz) in $CDCl_3$

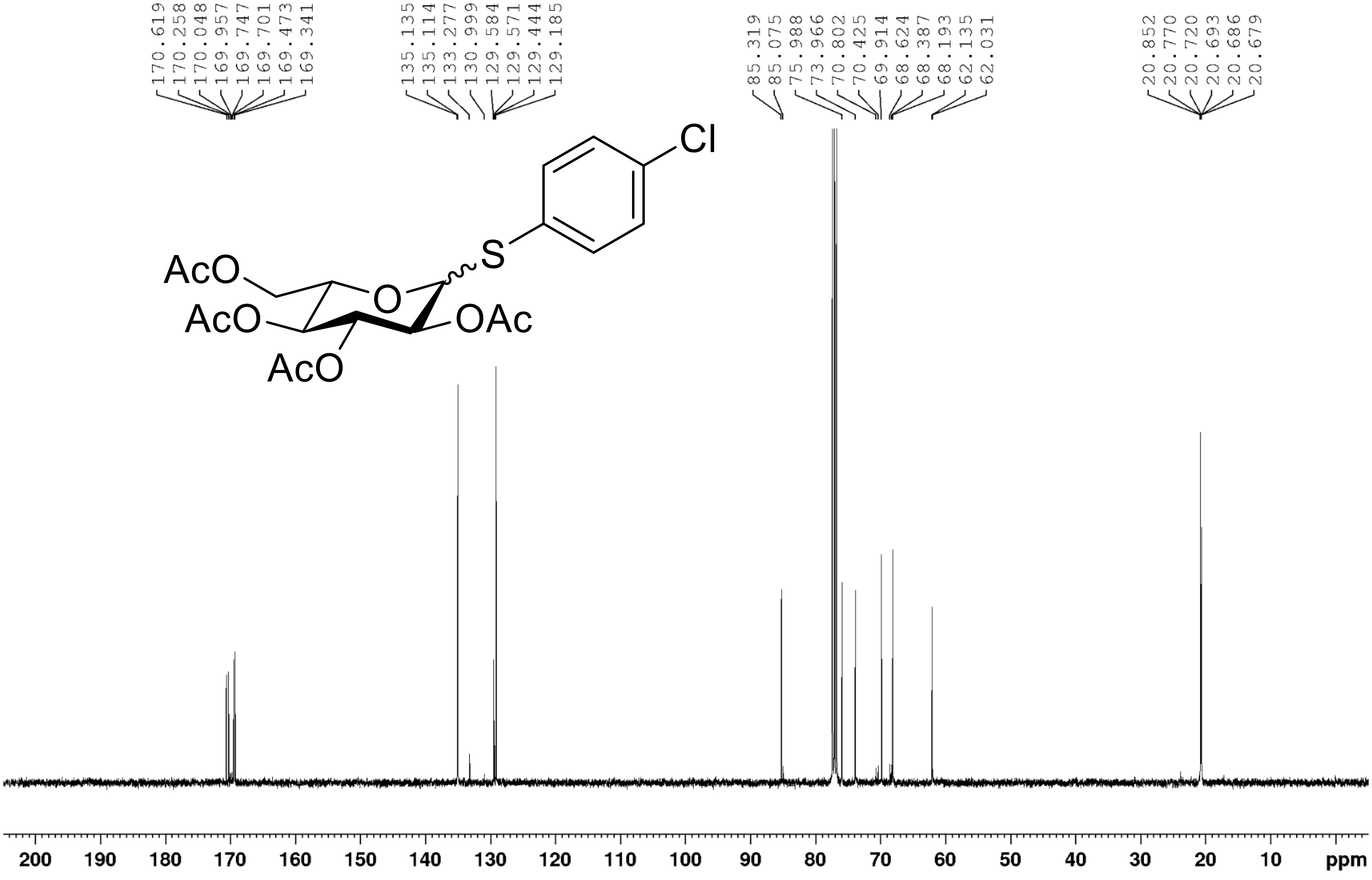

4-chlorophenyl 2,3,4-tri-*O*-acetyl-1-thio-L-glucopyranoside (**26**) α:β 7:93 ^{1}H NMR (400 MHz) in $CDCl_3$

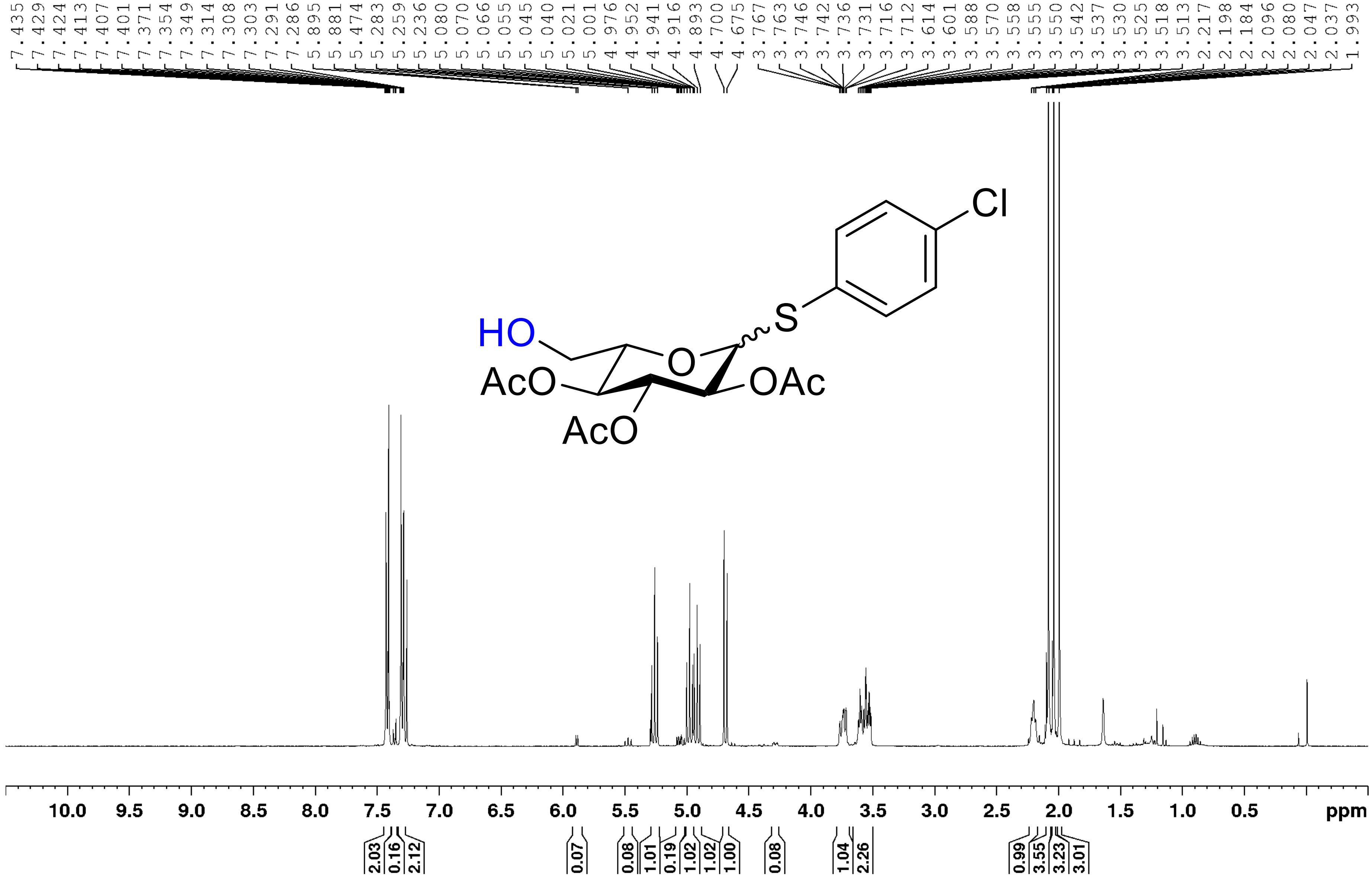

4-chlorophenyl 2,3,4-tri-*O*-acetyl-1-thio-L-glucopyranoside (**26**) α:β 7:93 ^1H-^1H COSY NMR (400 MHz) in $CDCl_3$

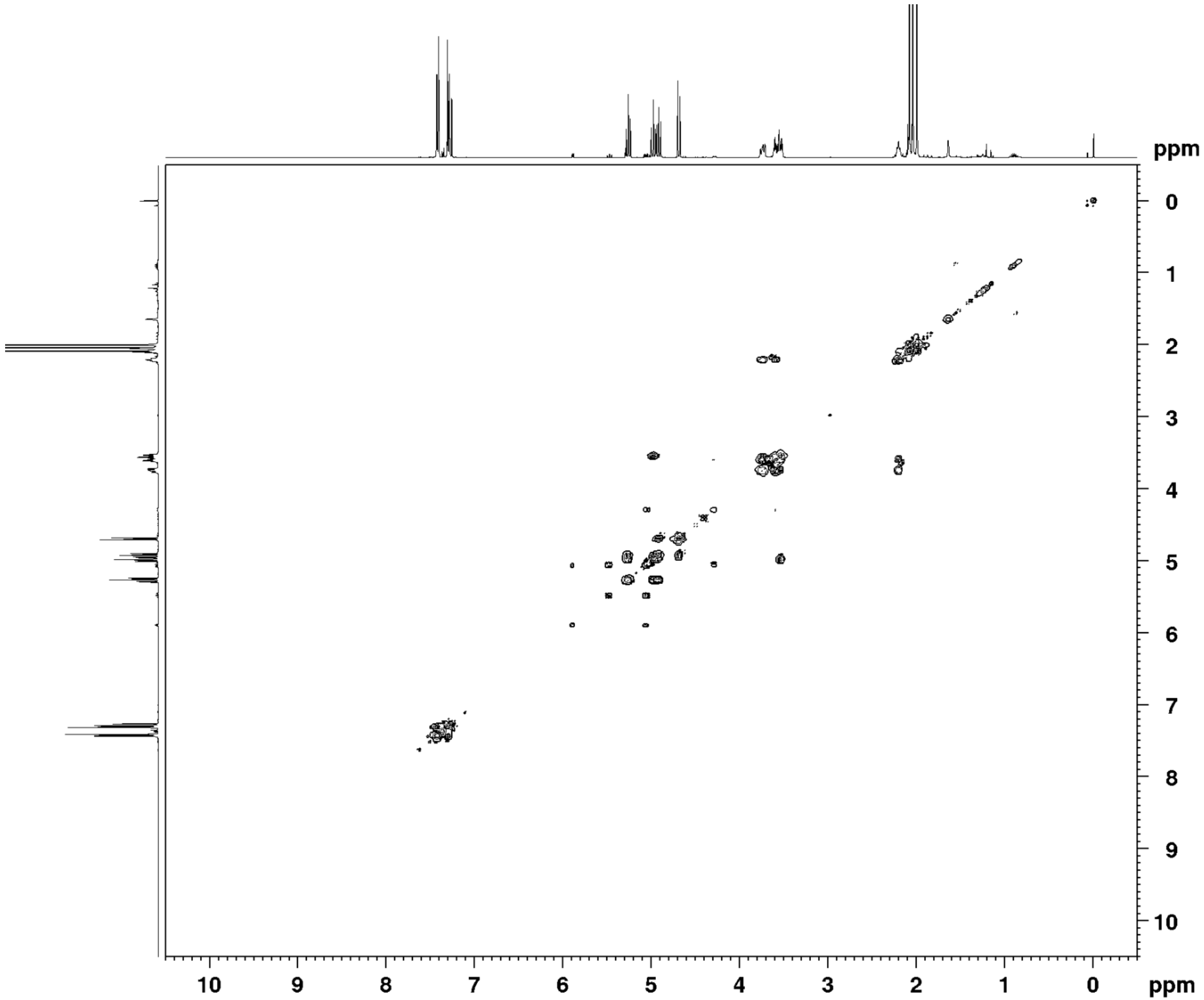

4-chlorophenyl 2,3,4-tri-*O*-acetyl-1-thio-L-glucopyranoside (**26**) α:β 7:93 ^{1}H-$^{13}C\{^{1}H\}$ HSQC NMR (400 & 101 MHz) in $CDCl_3$

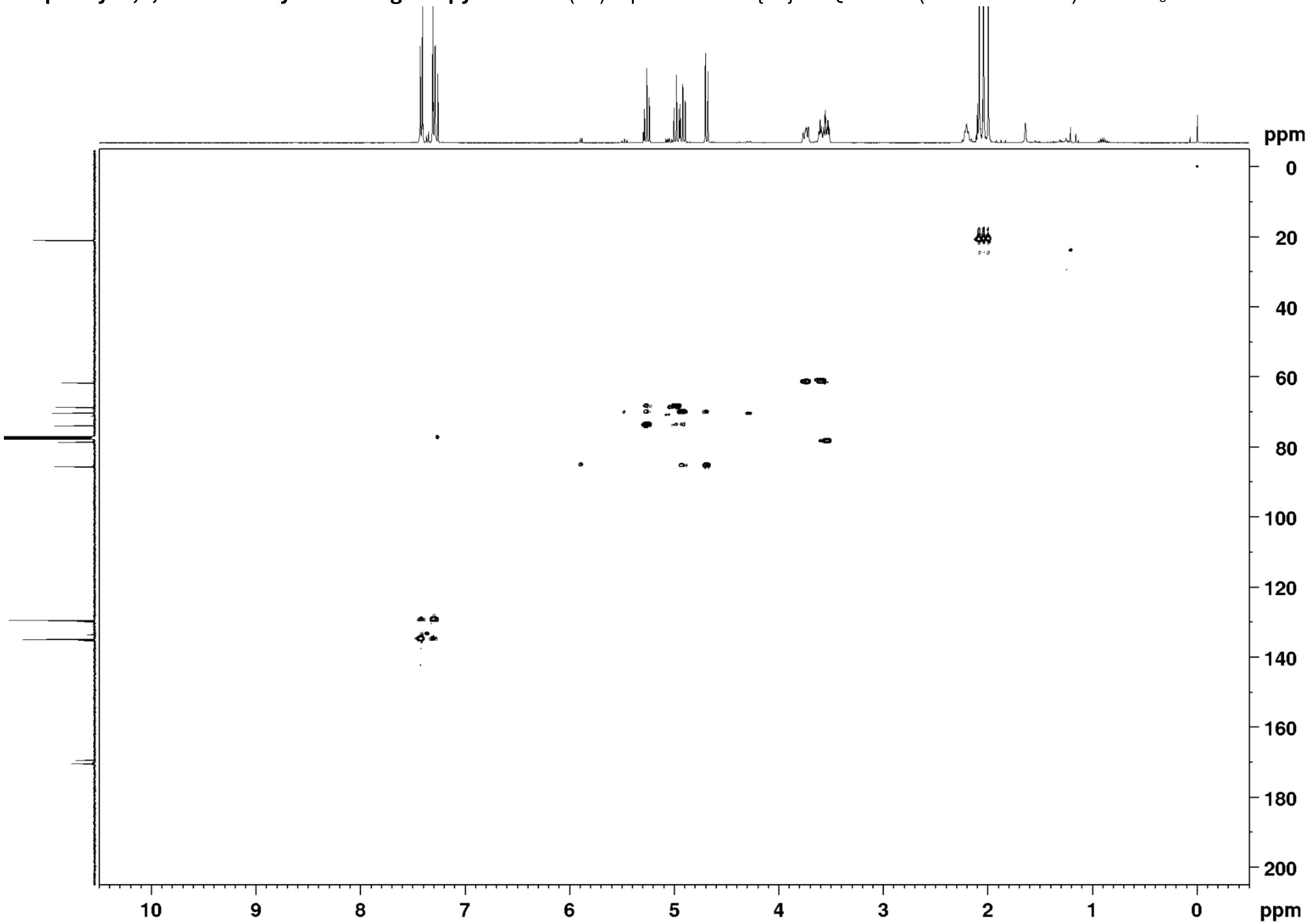

4-chlorophenyl 2,3,4-tri-*O*-acetyl-1-thio-L-glucopyranoside (**26**) α:β 7:93 ^{1}H-$^{13}C\{^{1}H\}$ HMBC NMR (400 & 101 MHz) in $CDCl_3$

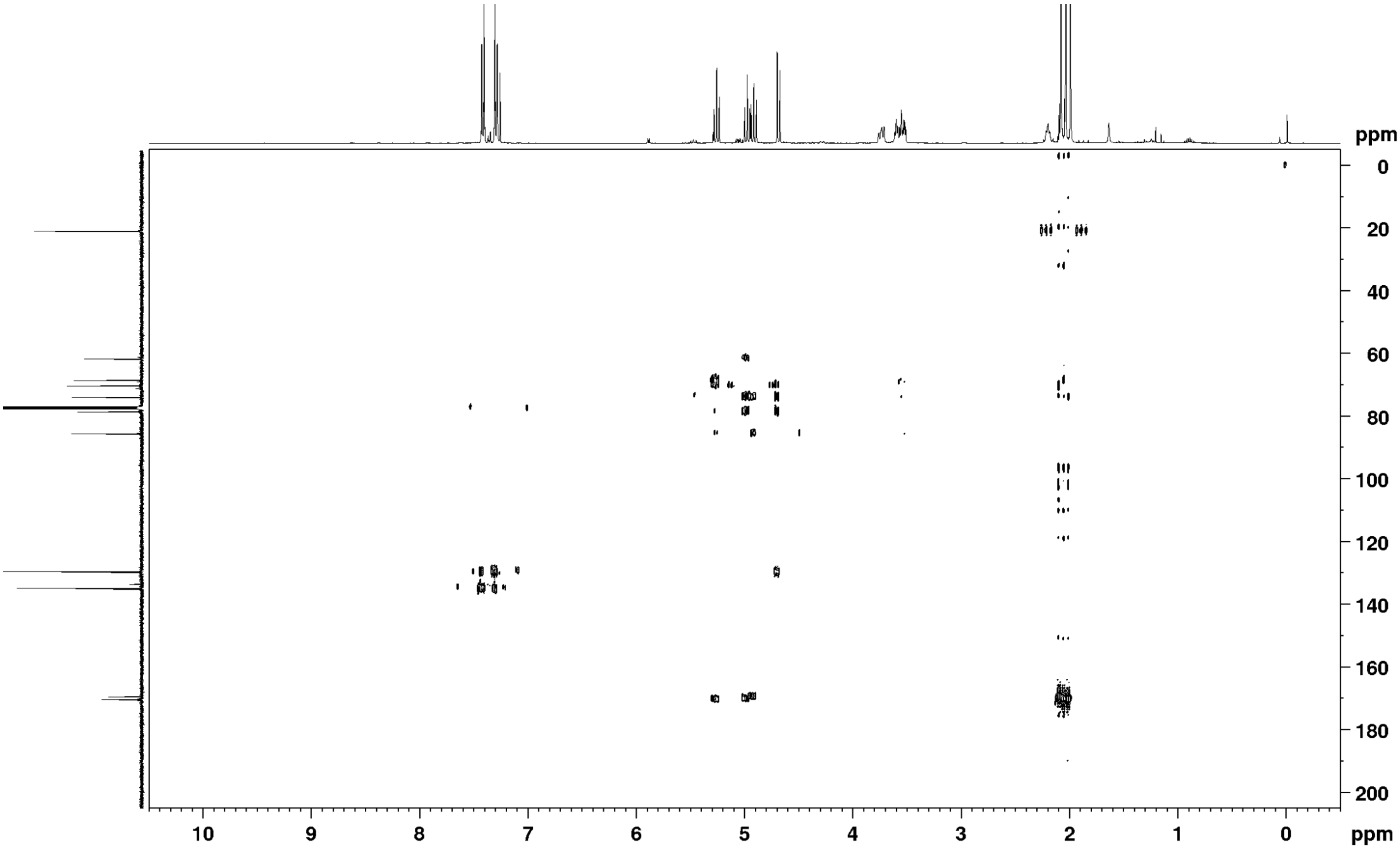

4-chlorophenyl 2,3,4-tri-*O*-acetyl-1-thio-L-glucopyranoside (**26**) α:β 7:93 $^{13}C\{^{1}H\}$ NMR (101 MHz) in $CDCl_3$

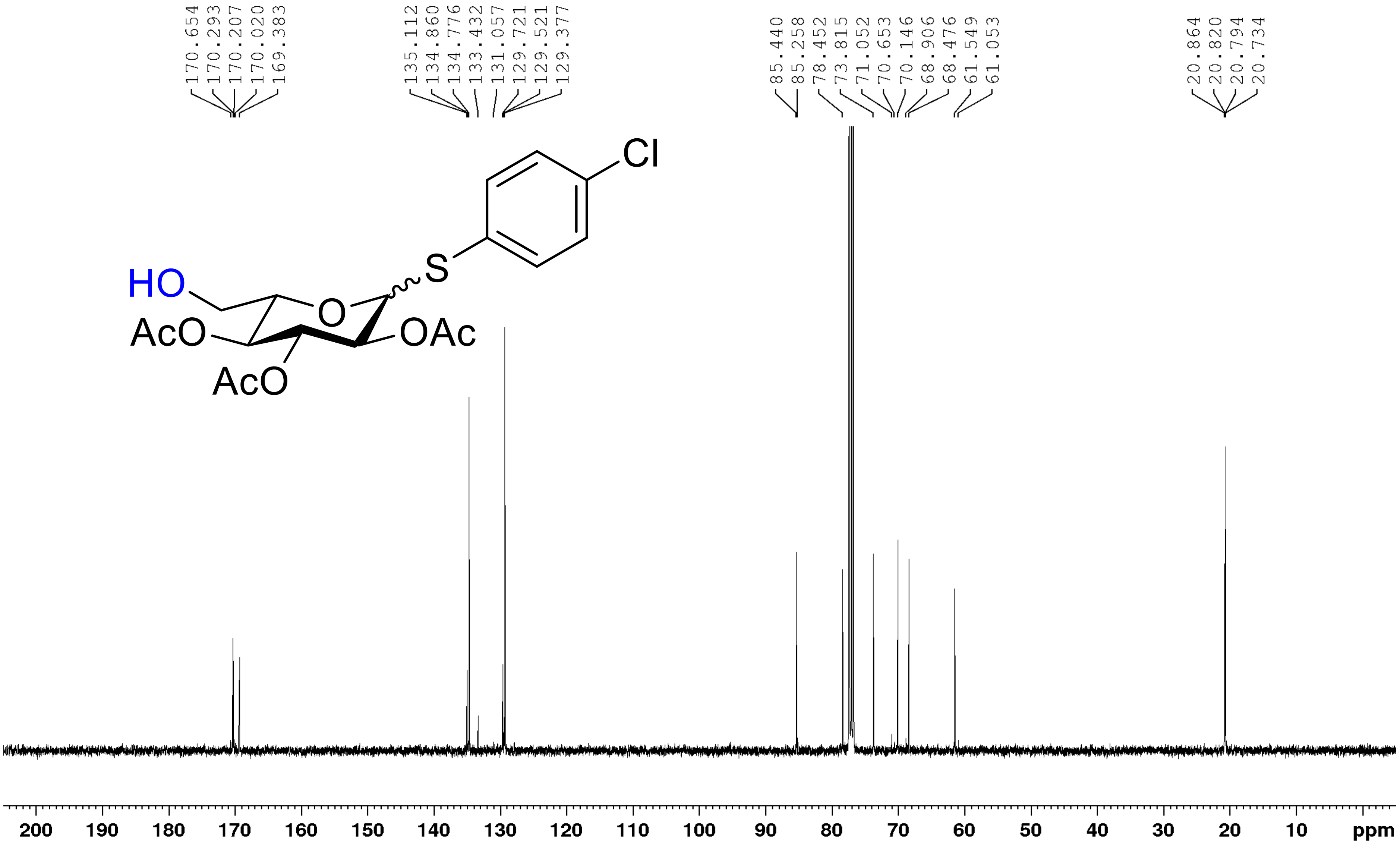

1,2,3,4-tetra-*O*-acetyl-D-arabinopyranoside (**27**) α:(β+furanose) 94:6 ^{1}H NMR (400 MHz) in $CDCl_3$

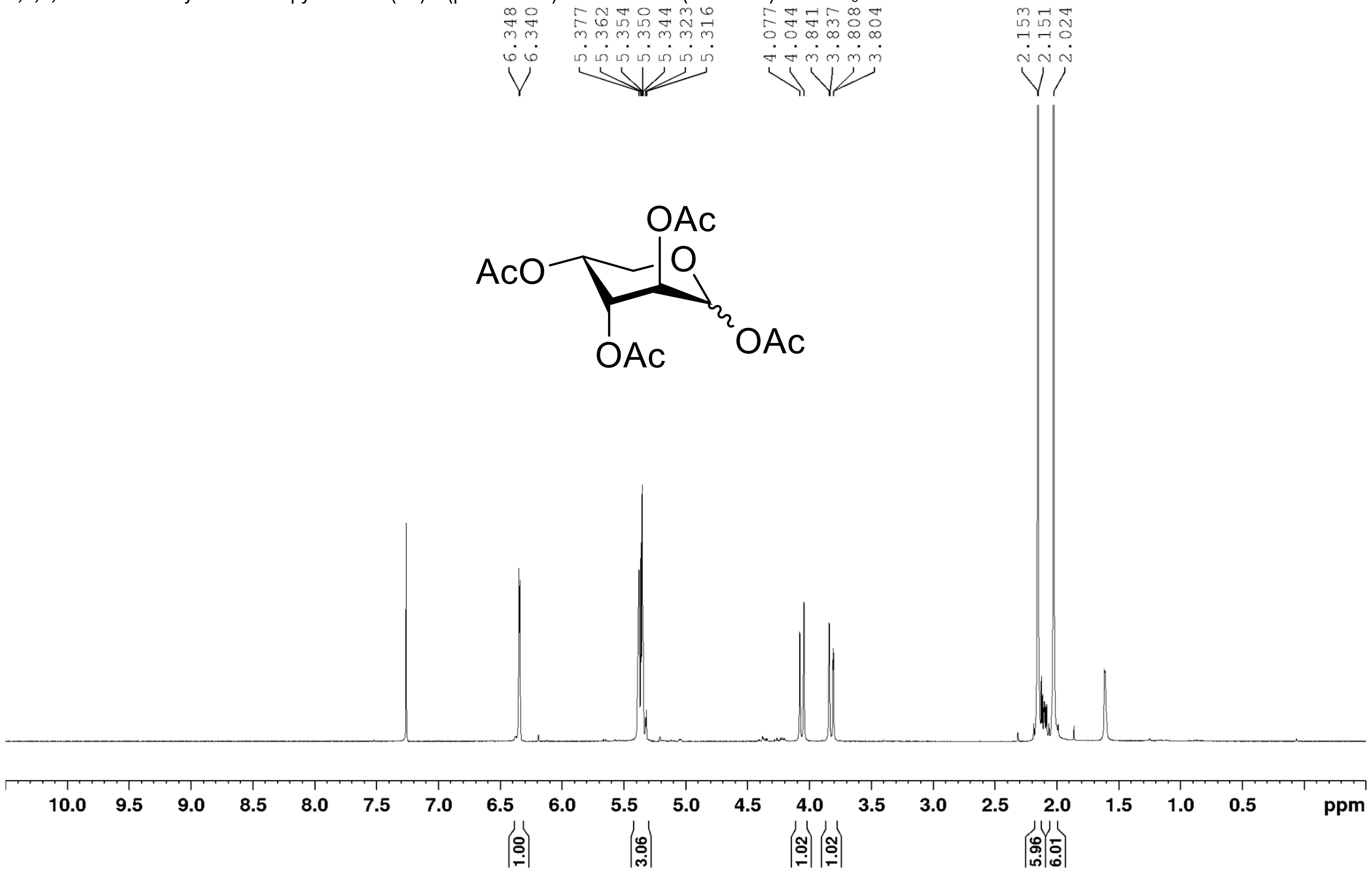

1,2,3,4-tetra-*O*-acetyl-L-arabinopyranoside (**28**) α:(β+furanose) 91:9 ^{1}H NMR (400 MHz) in $CDCl_3$

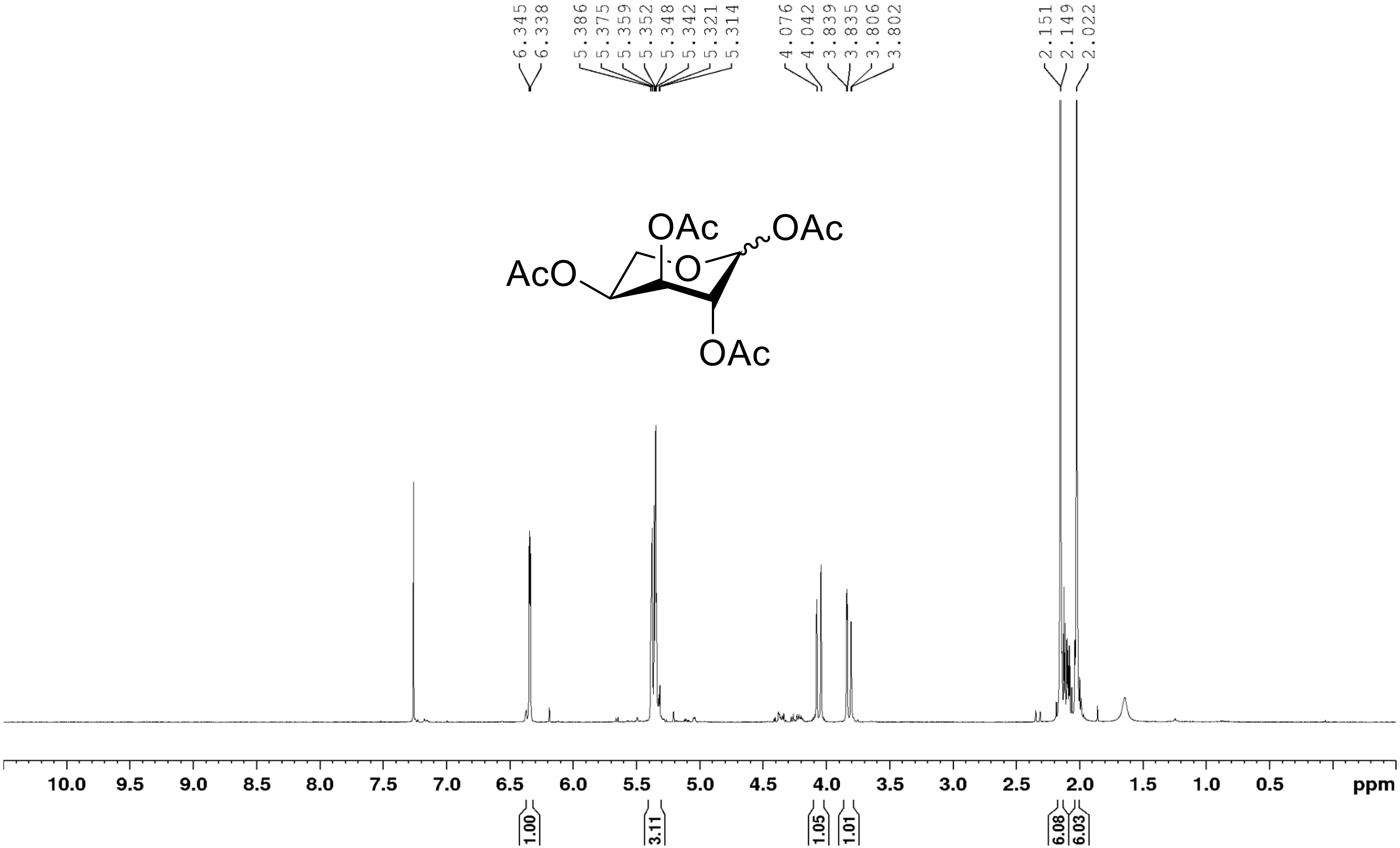

4-chlorophenyl 2,3,4-tri-*O*-acetyl-1-thio-D-arabinopyranoside (**29**) α:β 17:83 ^{1}H NMR (400 MHz) in $CDCl_3$

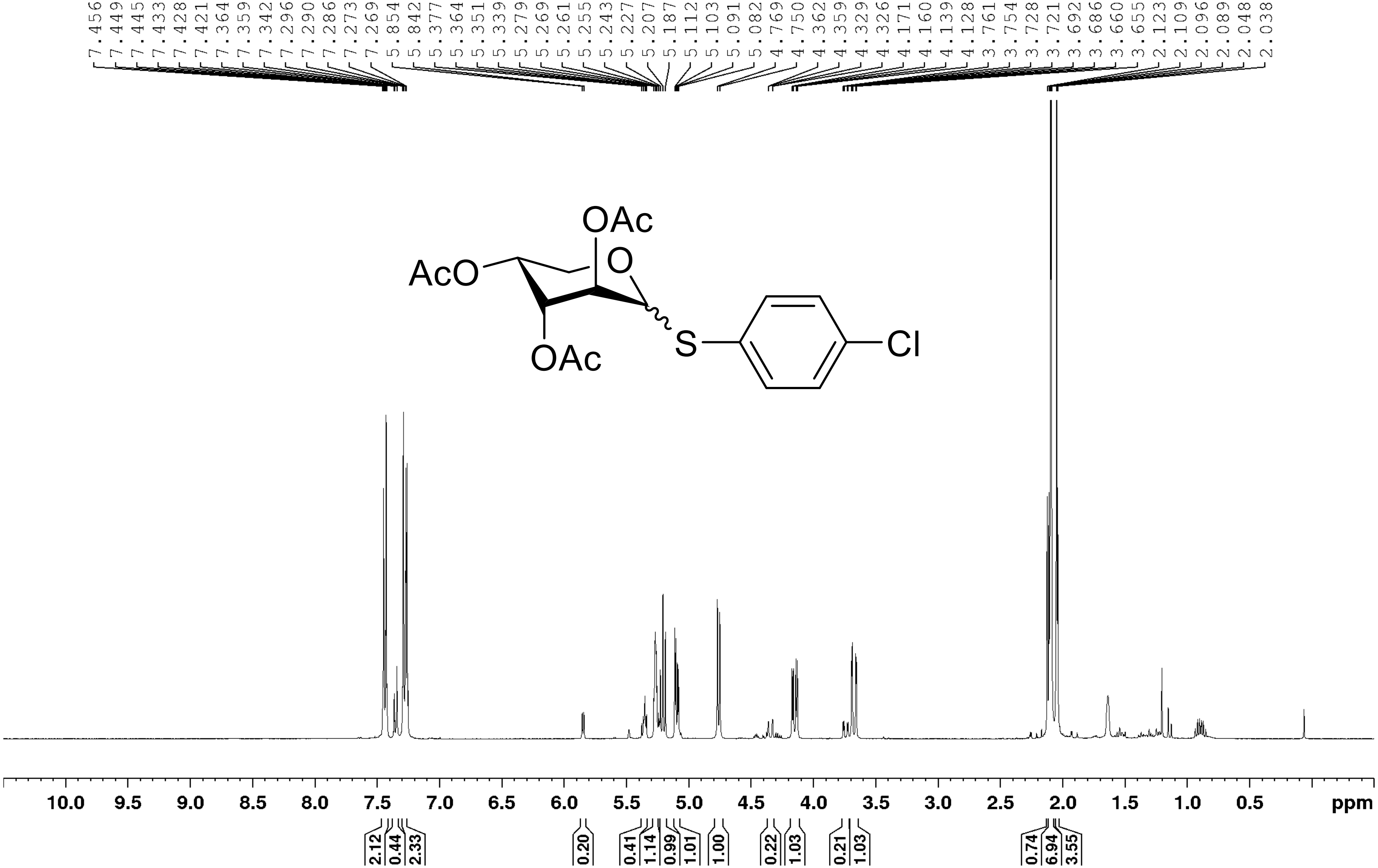

4-chlorophenyl 2,3,4-tri-*O*-acetyl-1-thio-D-arabinopyranoside (**29**) α:β 17:83 ^{1}H-^{1}H COSY NMR (400 MHz) in $CDCl_3$

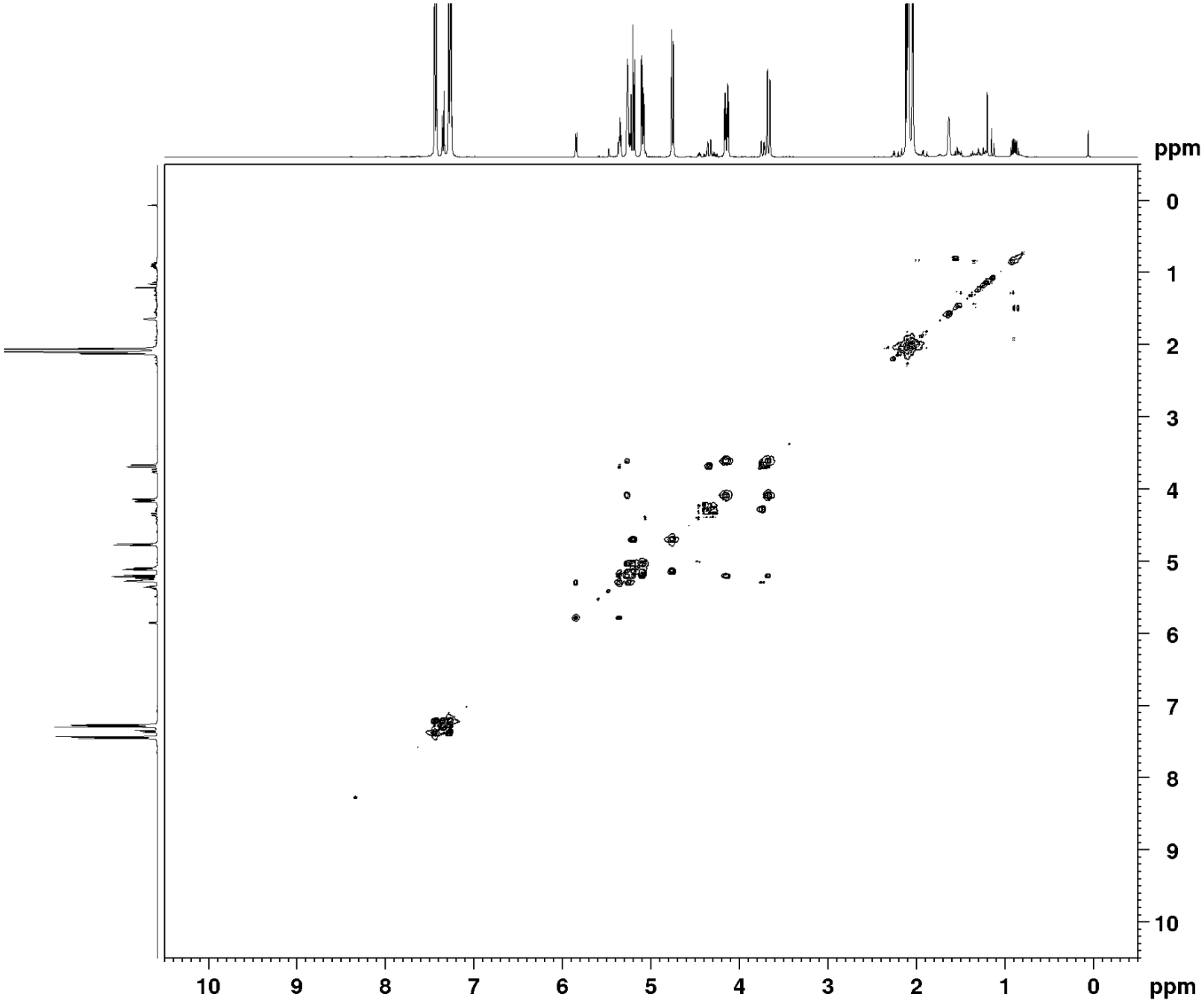

4-chlorophenyl 2,3,4-tri-*O*-acetyl-1-thio-D-arabinopyranoside (**29**) α:β 17:83 ^1H-$^{13}C\{^1H\}$ HSQC NMR (400 & 101 MHz) in $CDCl_3$

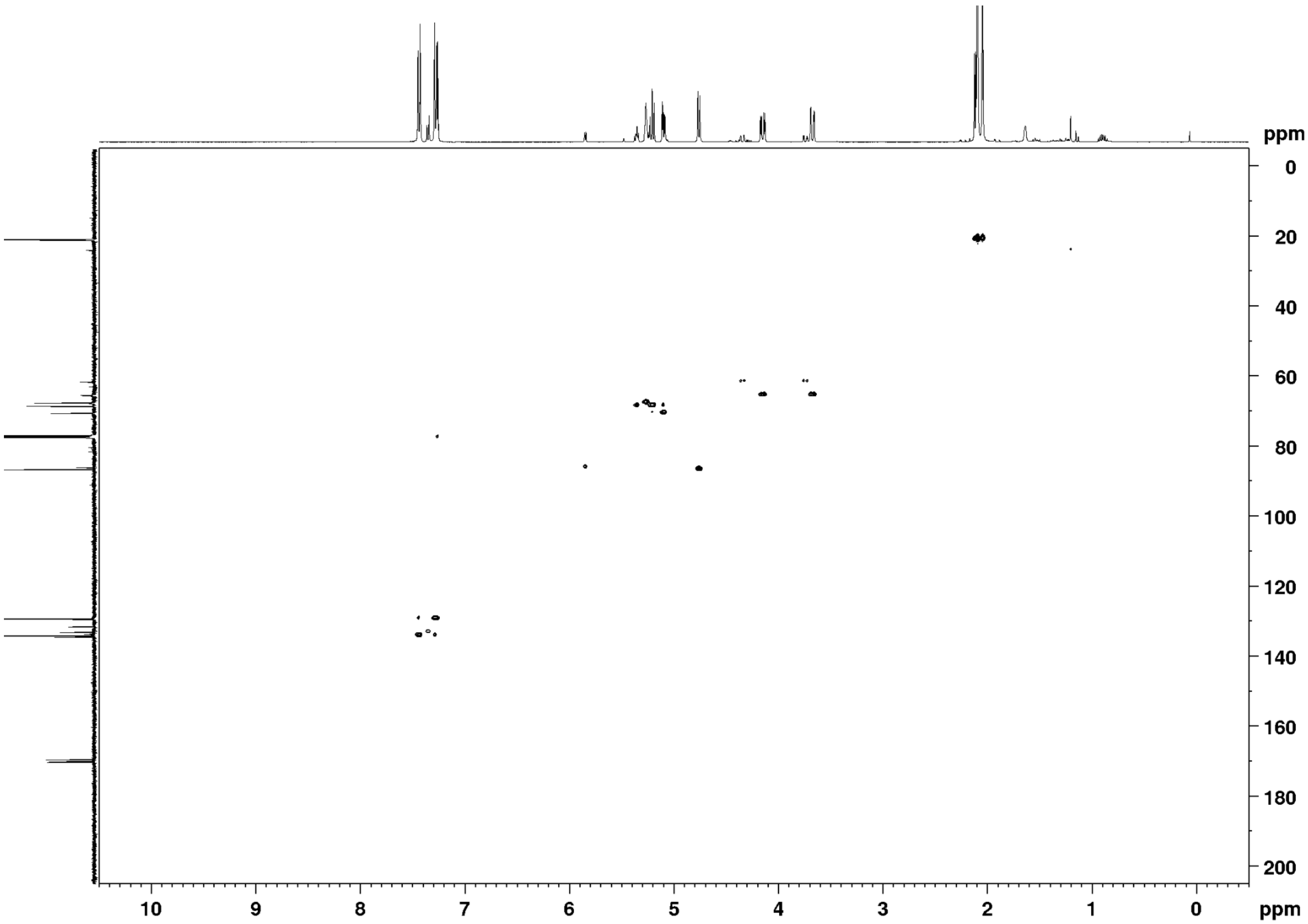

4-chlorophenyl 2,3,4-tri-*O*-acetyl-1-thio-D-arabinopyranoside (**29**) α:β 17:83 ^{1}H-$^{13}C\{^{1}H\}$ HMBC NMR (400 & 101 MHz) in $CDCl_3$

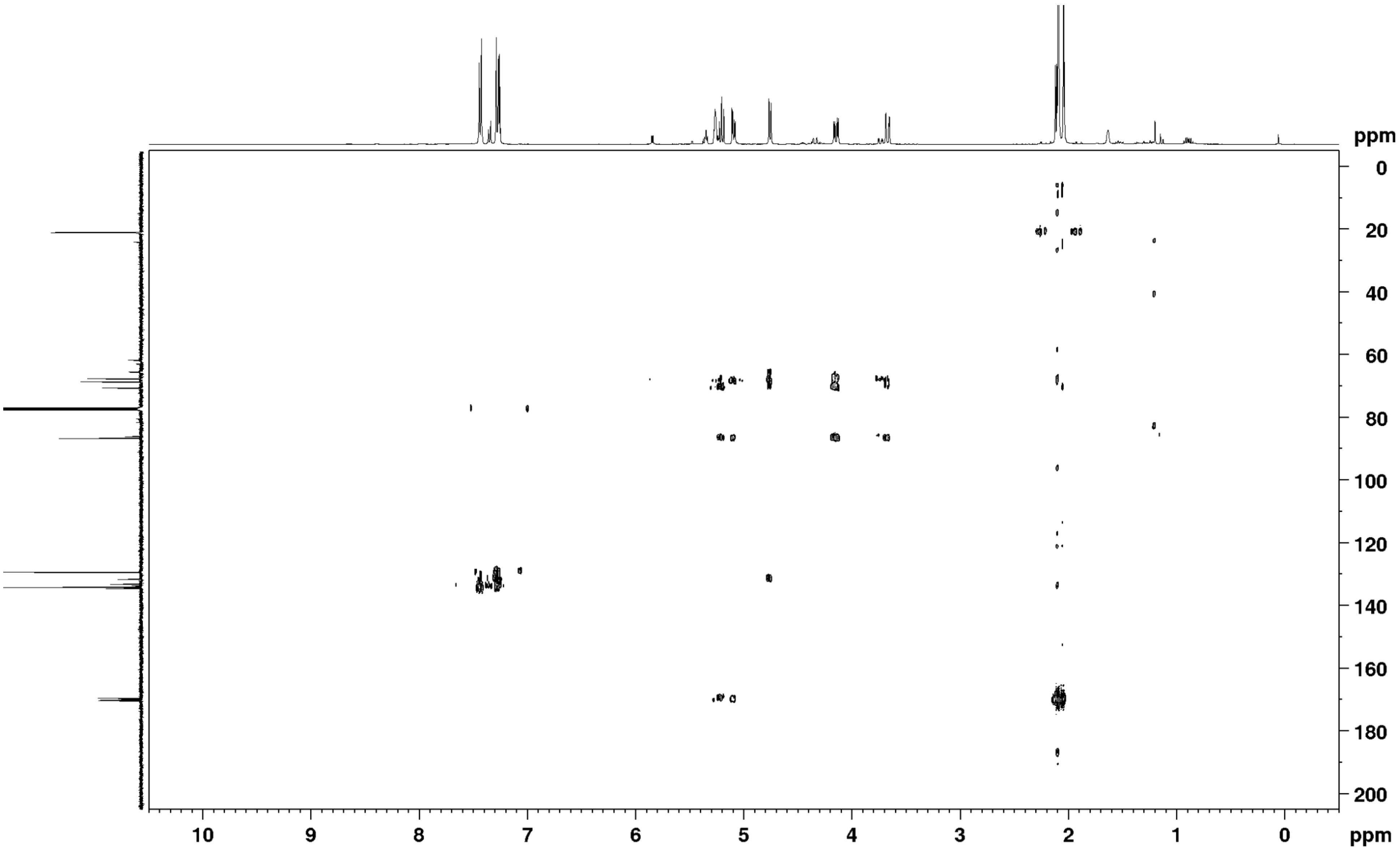

4-chlorophenyl 2,3,4-tri-*O*-acetyl-1-thio-D-arabinopyranoside (**29**) α:β 17:83 $^{13}C\{^1H\}$ NMR (101 MHz) in $CDCl_3$

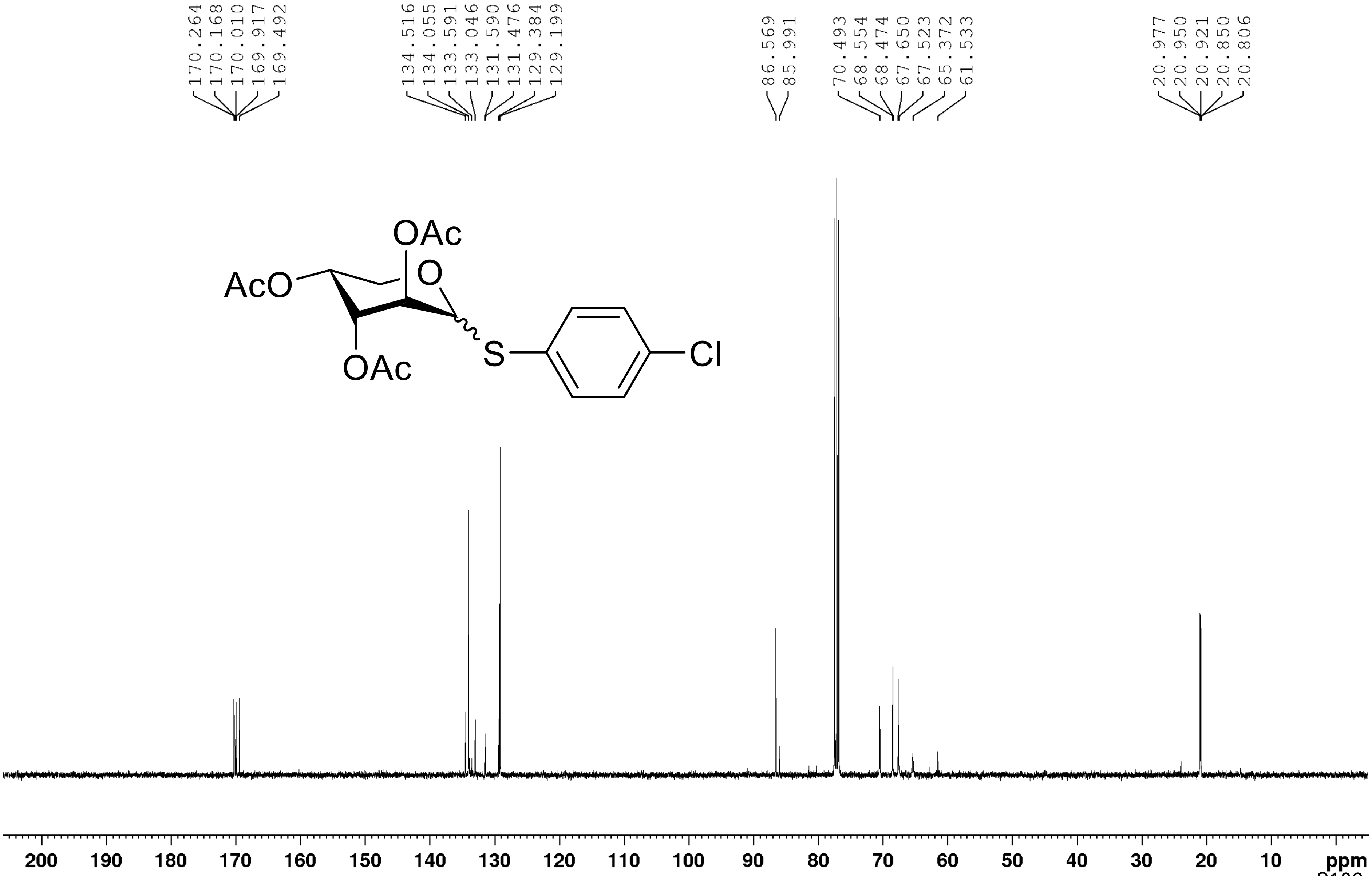

4-chlorophenyl 2-*O*-acetyl-1-thio-β-D-arabinopyranoside (30) ^{1}H NMR (400 MHz) in $CDCl_3$

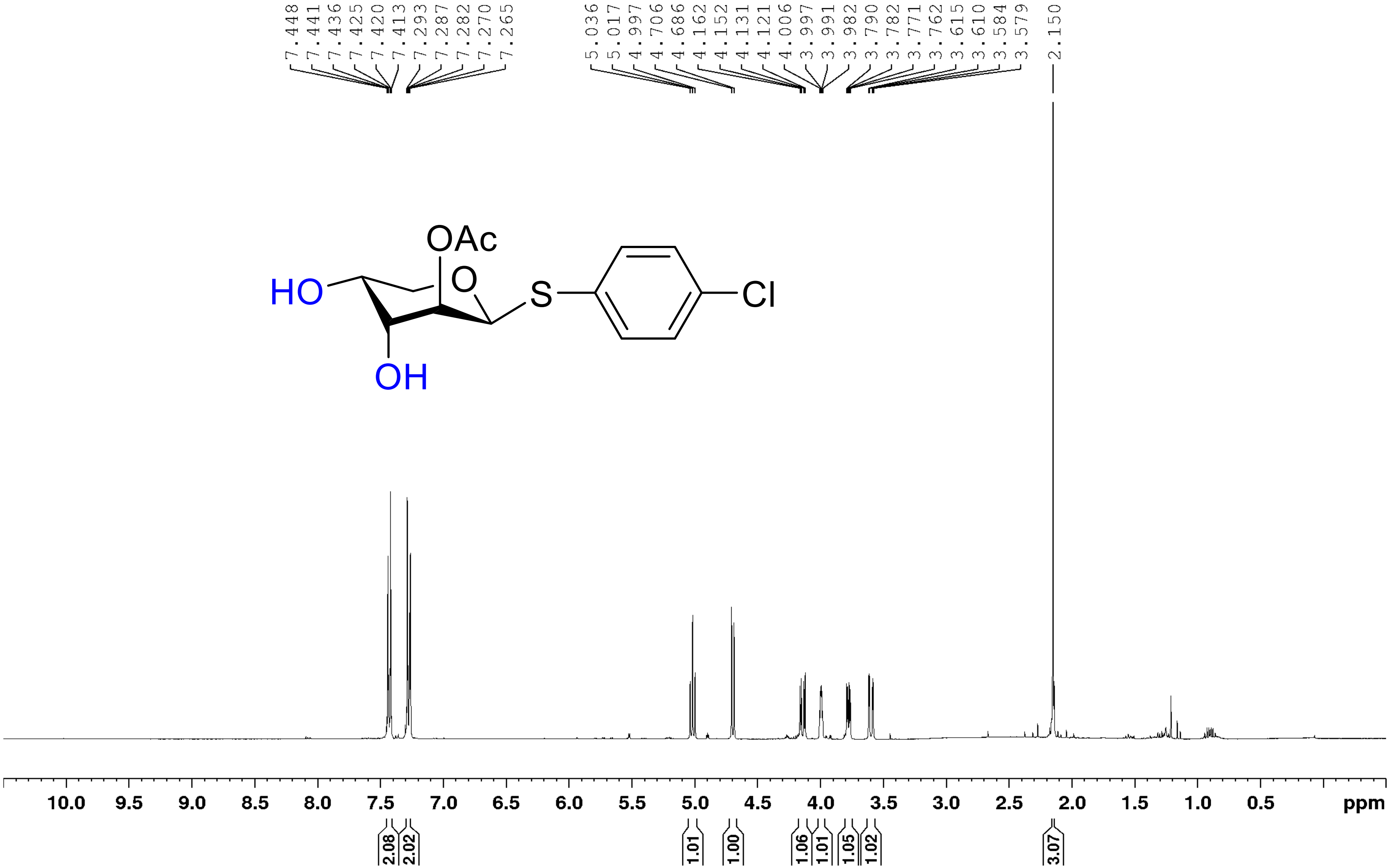

4-chlorophenyl 2-*O*-acetyl-1-thio-β-D-arabinopyranoside (**30**) ^{1}H-^{1}H COSY NMR (400 MHz) in $CDCl_3$

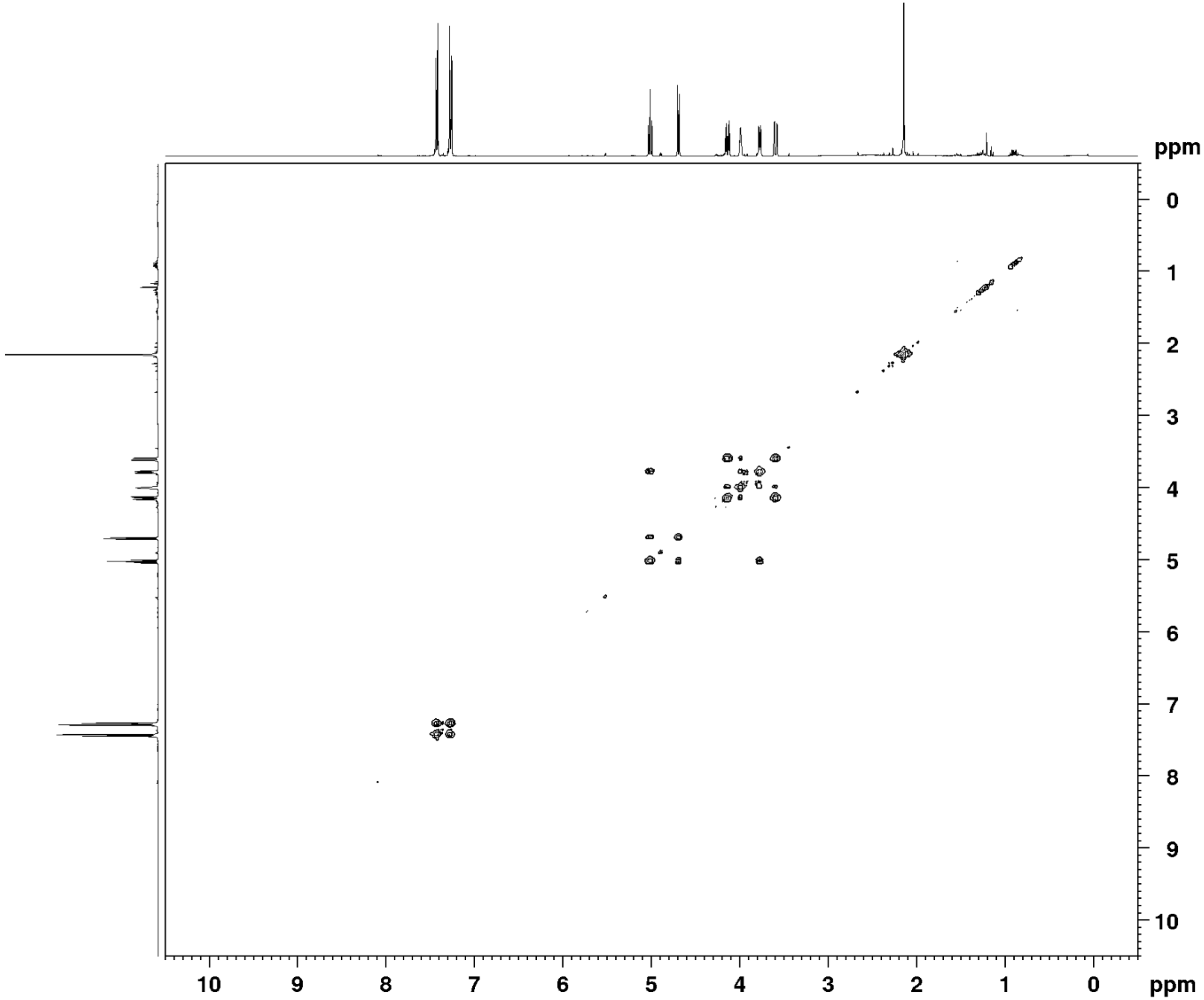

4-chlorophenyl 2-*O*-acetyl-1-thio-β-D-arabinopyranoside (**30**) ^{1}H-$^{13}C\{^{1}H\}$ HSQC NMR (400 & 101 MHz) in $CDCl_3$

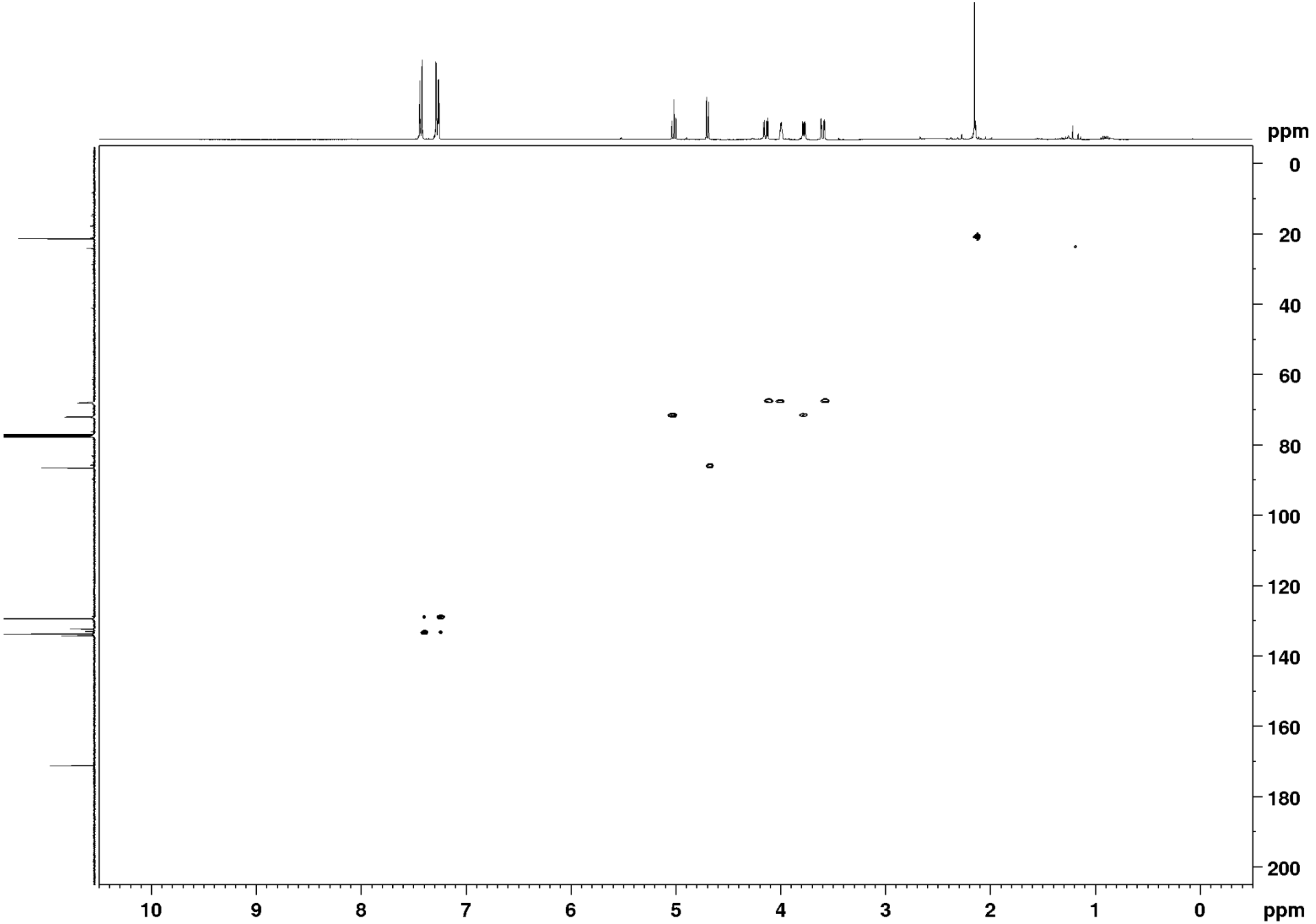

4-chlorophenyl 2-*O*-acetyl-1-thio-β-D-arabinopyranoside (**30**) ^{1}H-^{13}C{^{1}H} HMBC NMR (400 & 101 MHz) in $CDCl_3$

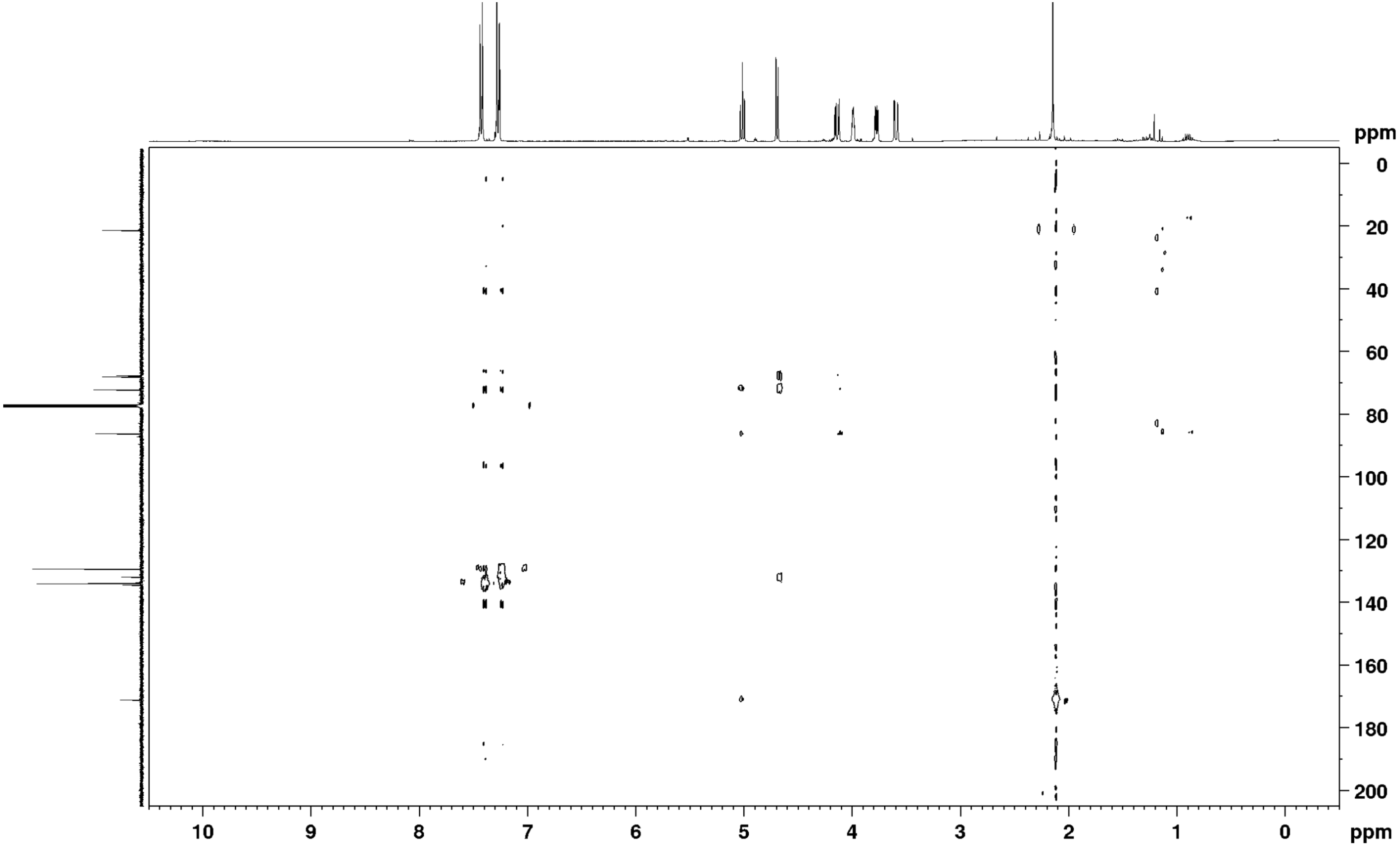

4-chlorophenyl 2-*O*-acetyl-1-thio-β-D-arabinopyranoside (30) $^{13}C\{^{1}H\}$ NMR (101 MHz) in $CDCl_3$

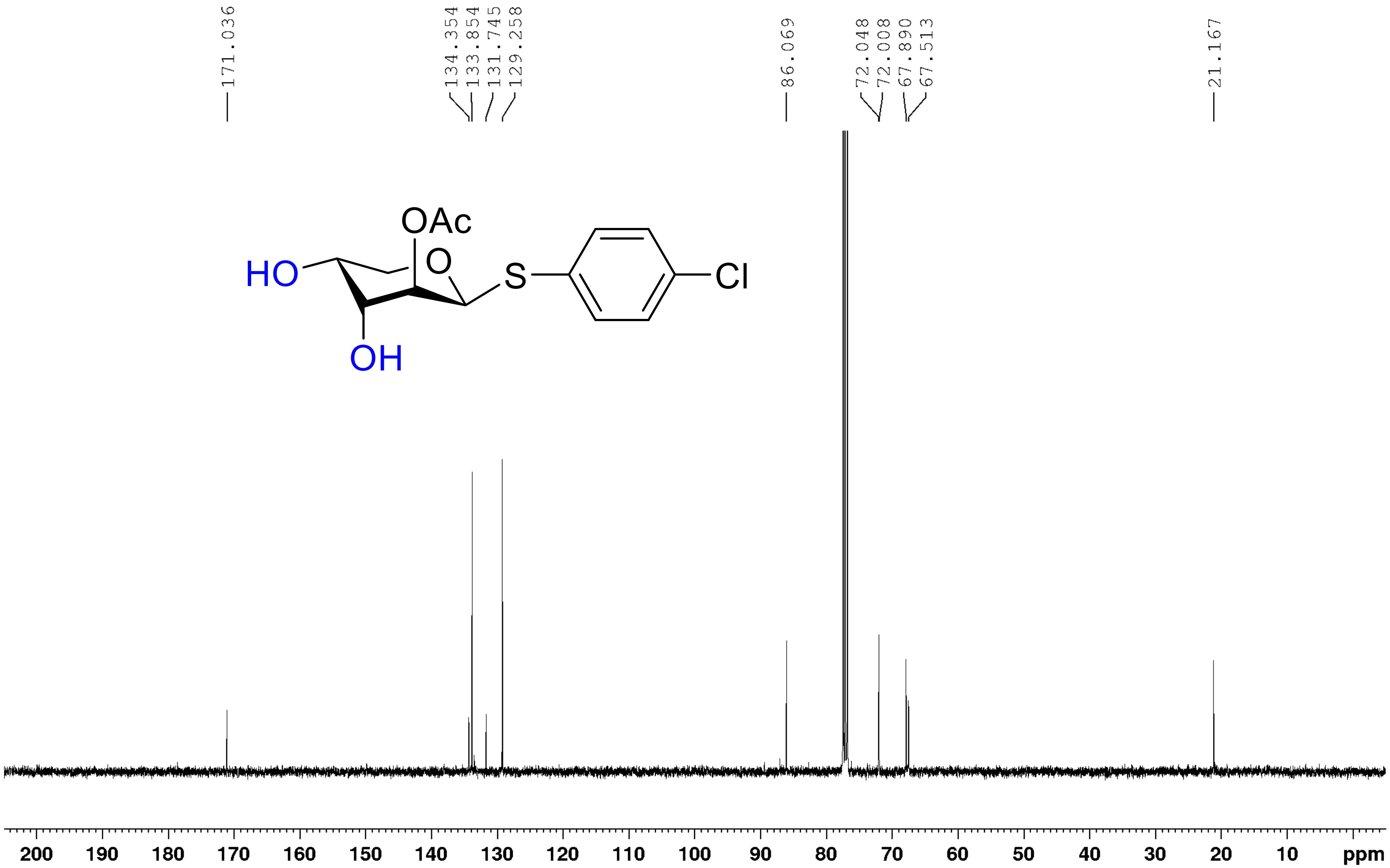

4-chlorophenyl 2,3,4-tri-*O*-acetyl-1-thio-L-arabinopyranoside (31) α:β 17:83 ^{1}H NMR (400 MHz) in $CDCl_3$

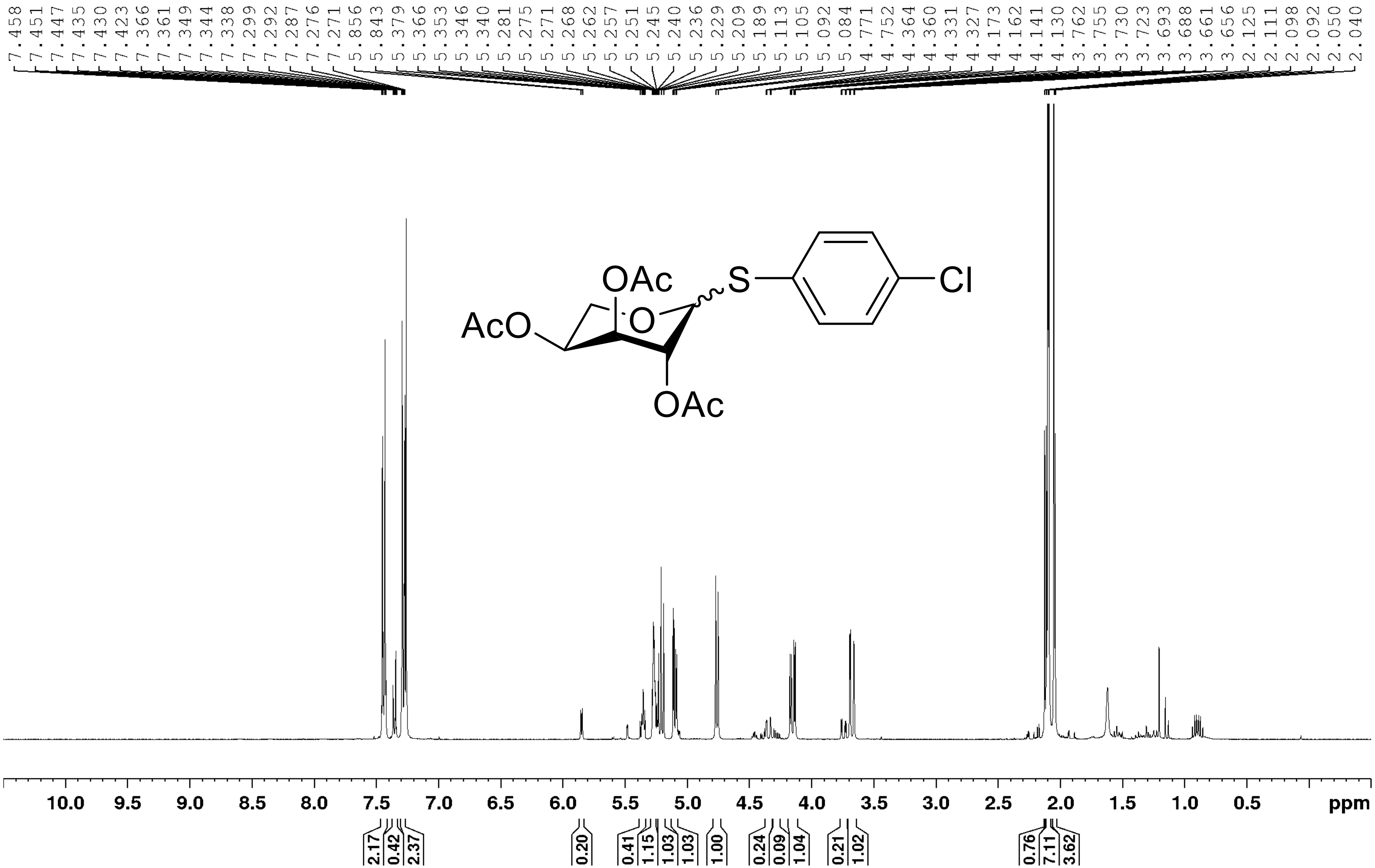

4-chlorophenyl 2,3,4-tri-*O*-acetyl-1-thio-L-arabinopyranoside (31) α:β 17:83 ^{1}H-^{1}H COSY NMR (400 MHz) in $CDCl_3$

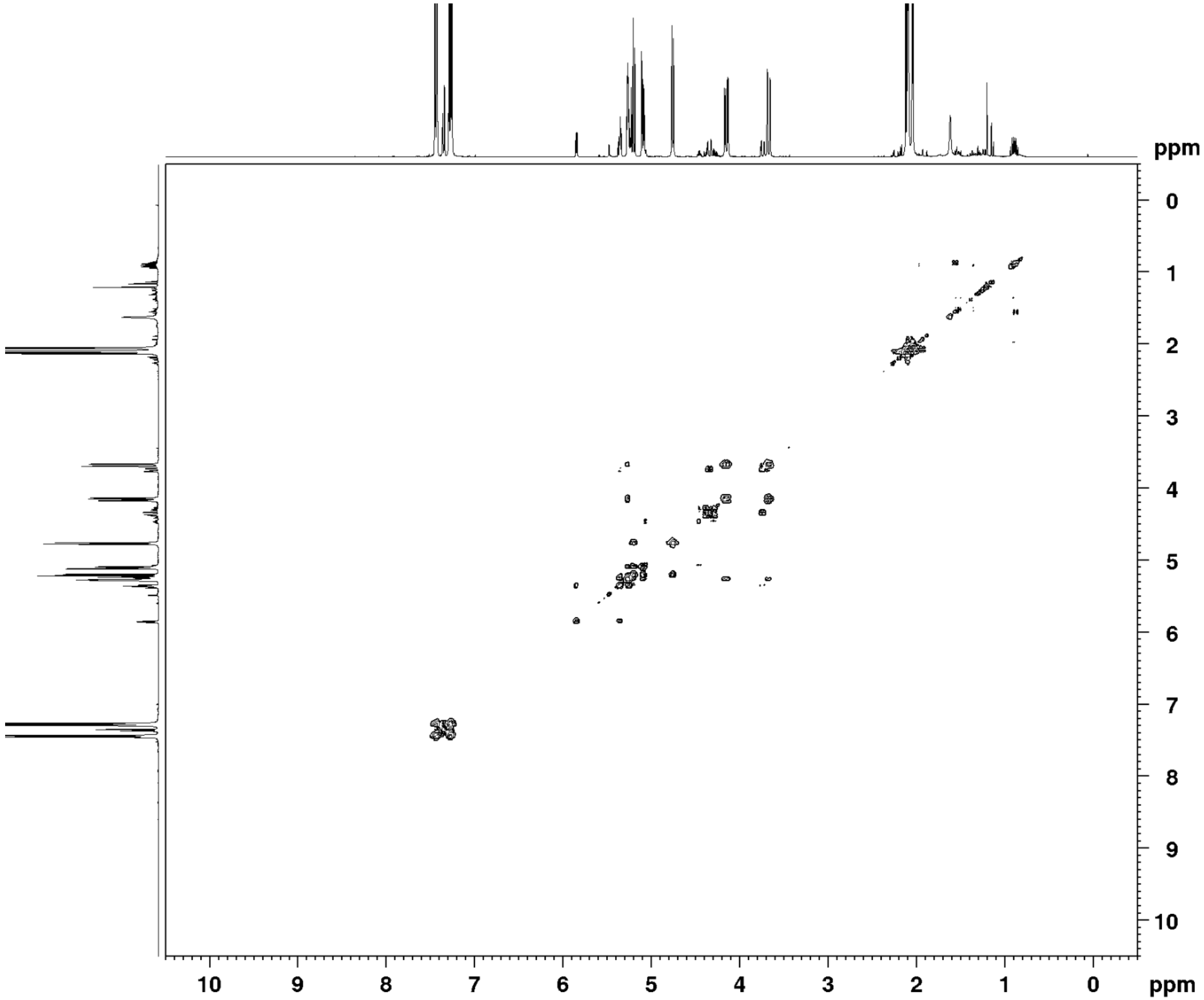

4-chlorophenyl 2,3,4-tri-*O*-acetyl-1-thio-L-arabinopyranoside (**31**) α:β 17:83 ^{1}H-$^{13}C\{^{1}H\}$ HSQC NMR (400 & 101 MHz) in $CDCl_3$

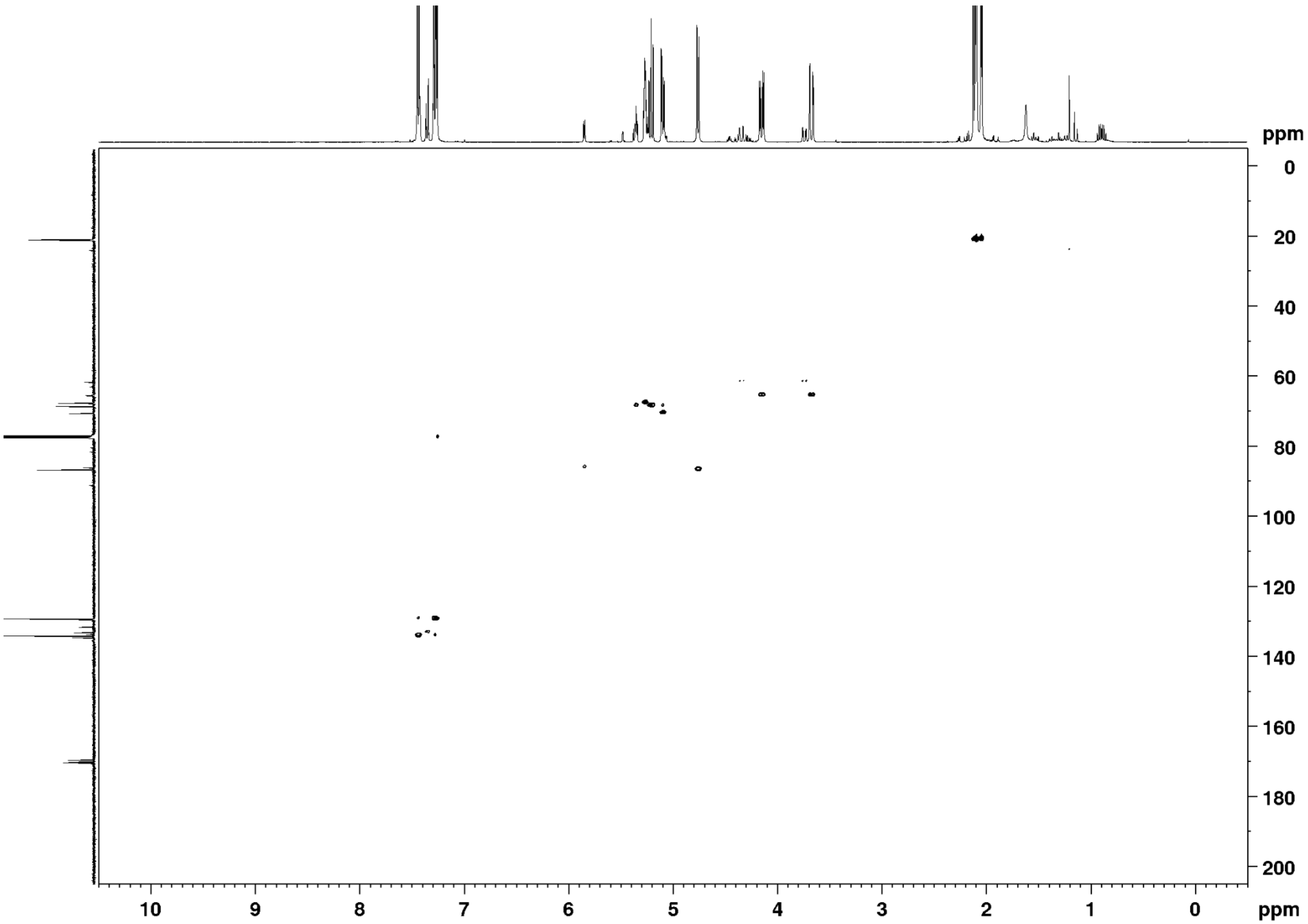

4-chlorophenyl 2,3,4-tri-*O*-acetyl-1-thio-L-arabinopyranoside (31) α:β 17:83 ^{1}H-$^{13}C\{^{1}H\}$ HMBC NMR (400 & 101 MHz) in $CDCl_3$

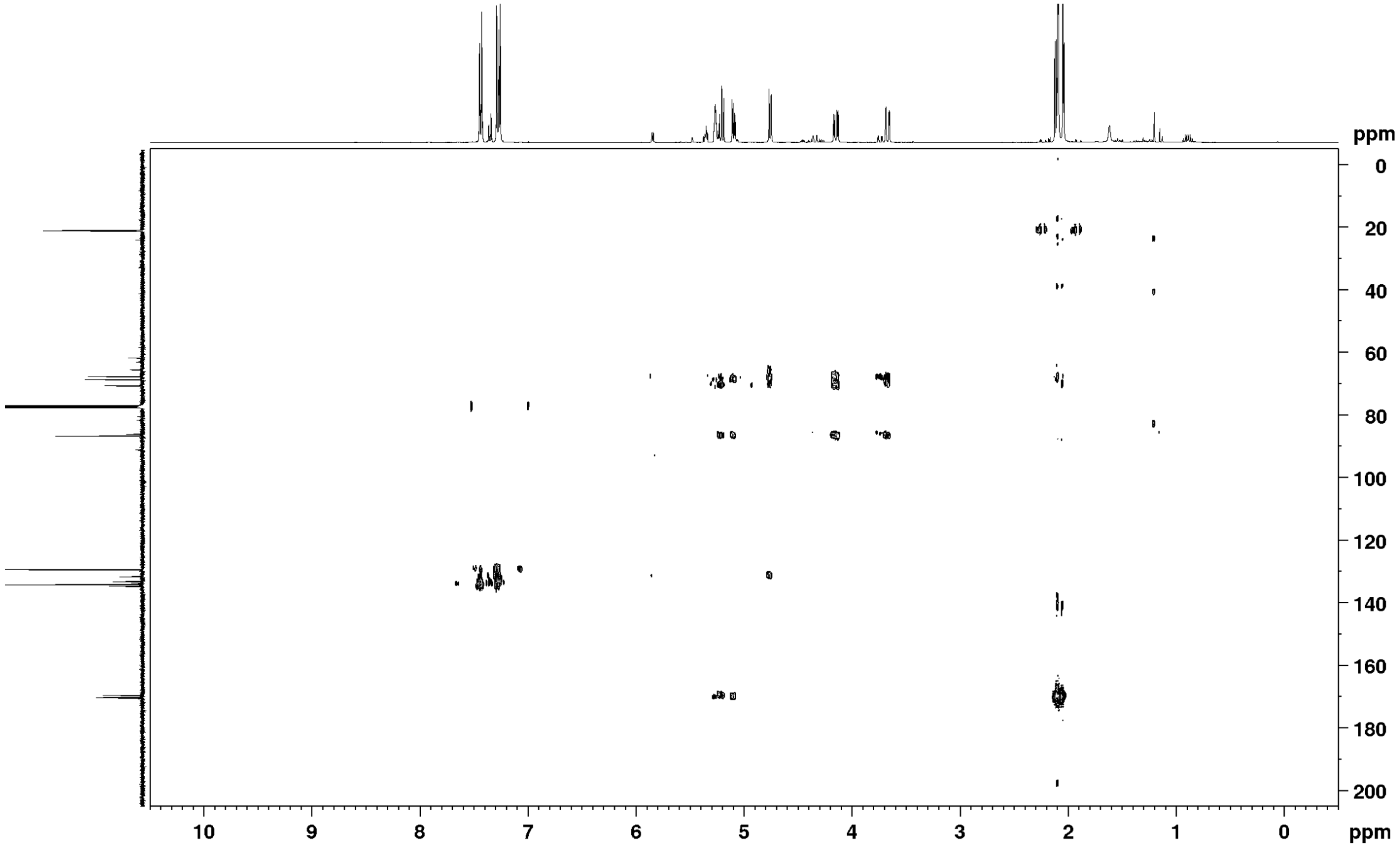

4-chlorophenyl 2,3,4-tri-*O*-acetyl-1-thio-L-arabinopyranoside (31) α:β 17:83 $^{13}C\{^{1}H\}$ NMR (101 MHz) in $CDCl_3$

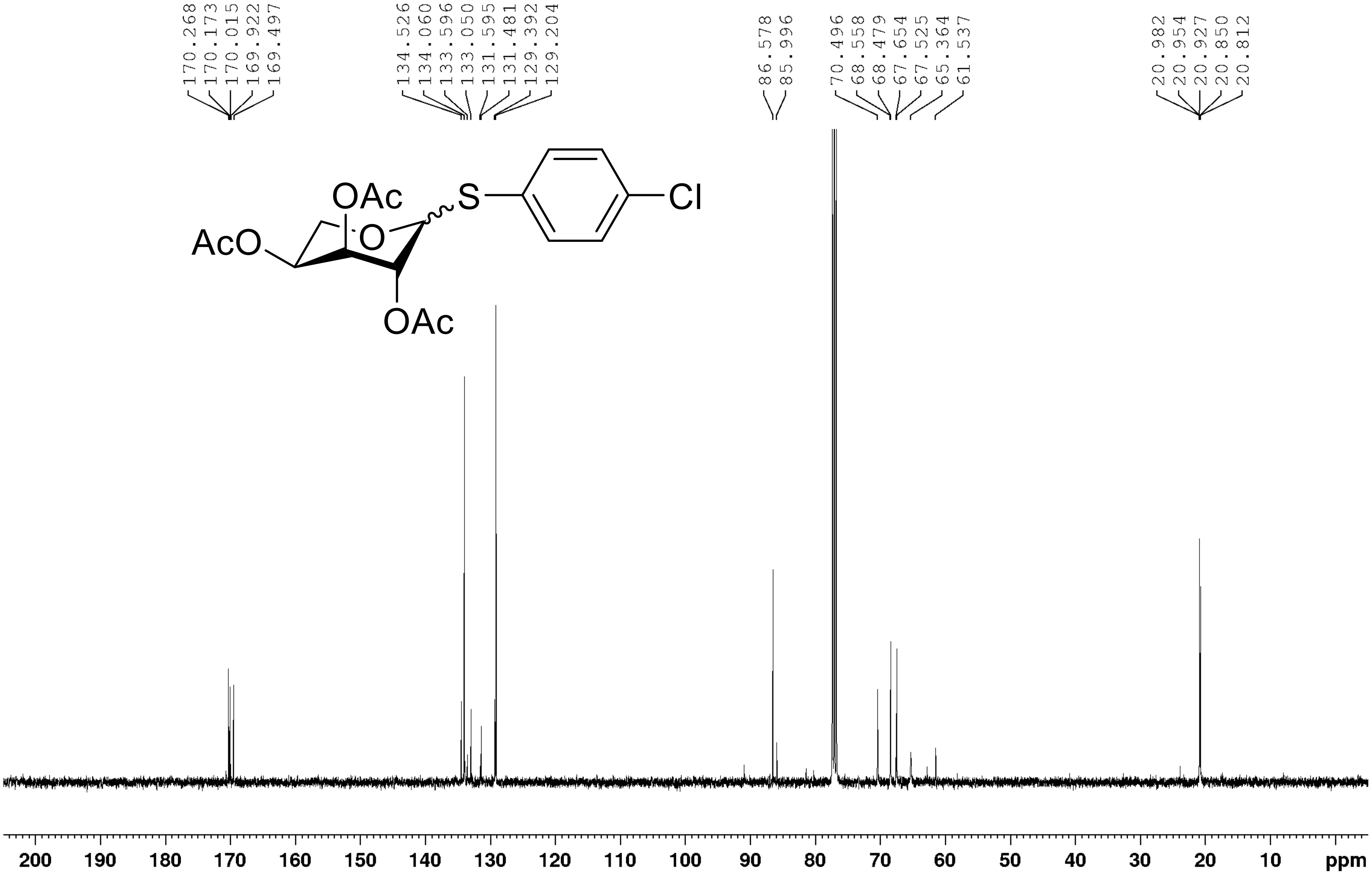

1,2,3,4-tetra-*O*-acetyl-6-deoxy-L-mannopyranoside (**32**) α:β 83:17 ^{1}H NMR (400 MHz) in $CDCl_3$

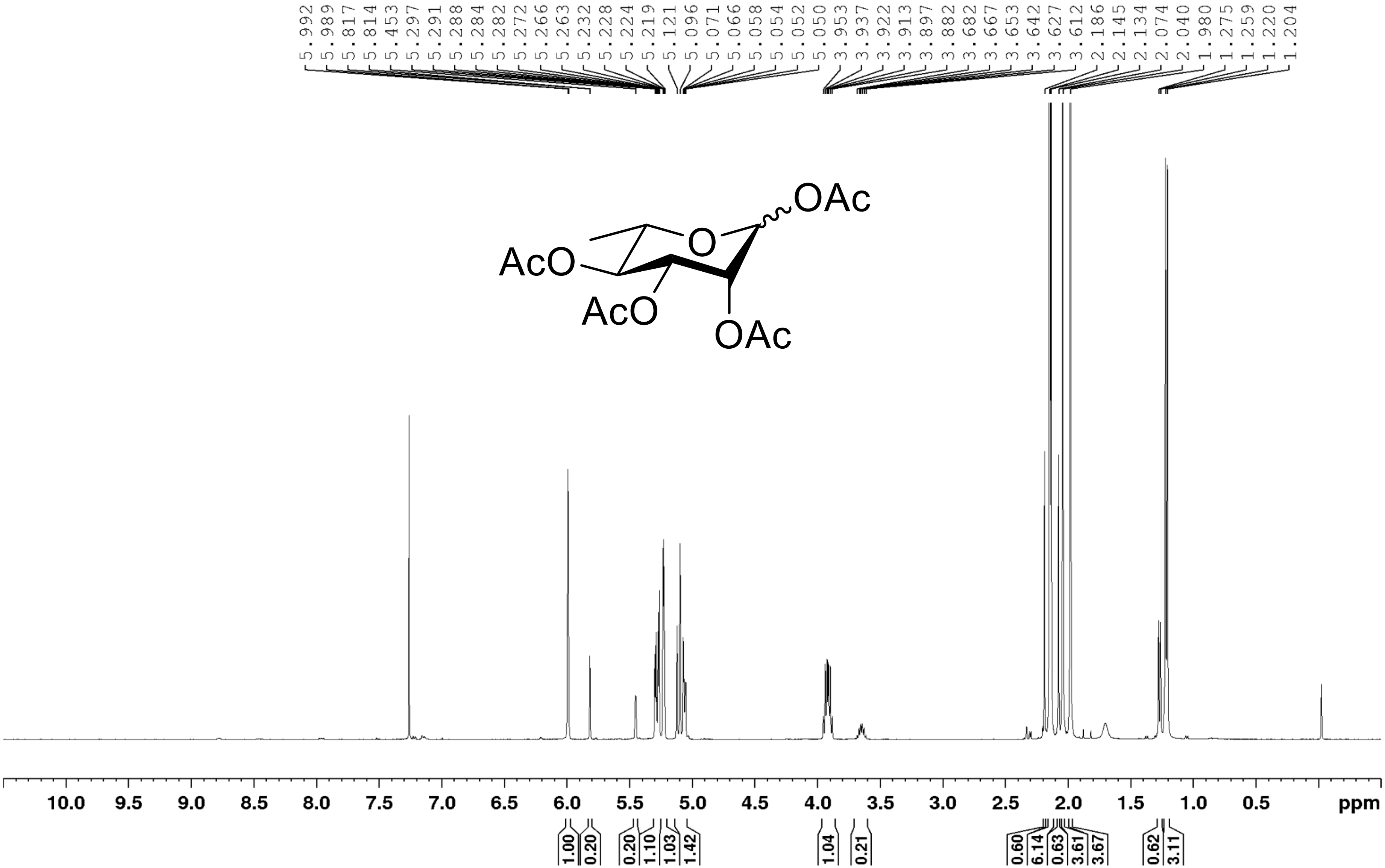

2,3,4-tri-*O*-acetyl-6-deoxy-L-mannopyranose (**33**) α:β 91:9 ^{1}H NMR (400 MHz) in $CDCl_3$

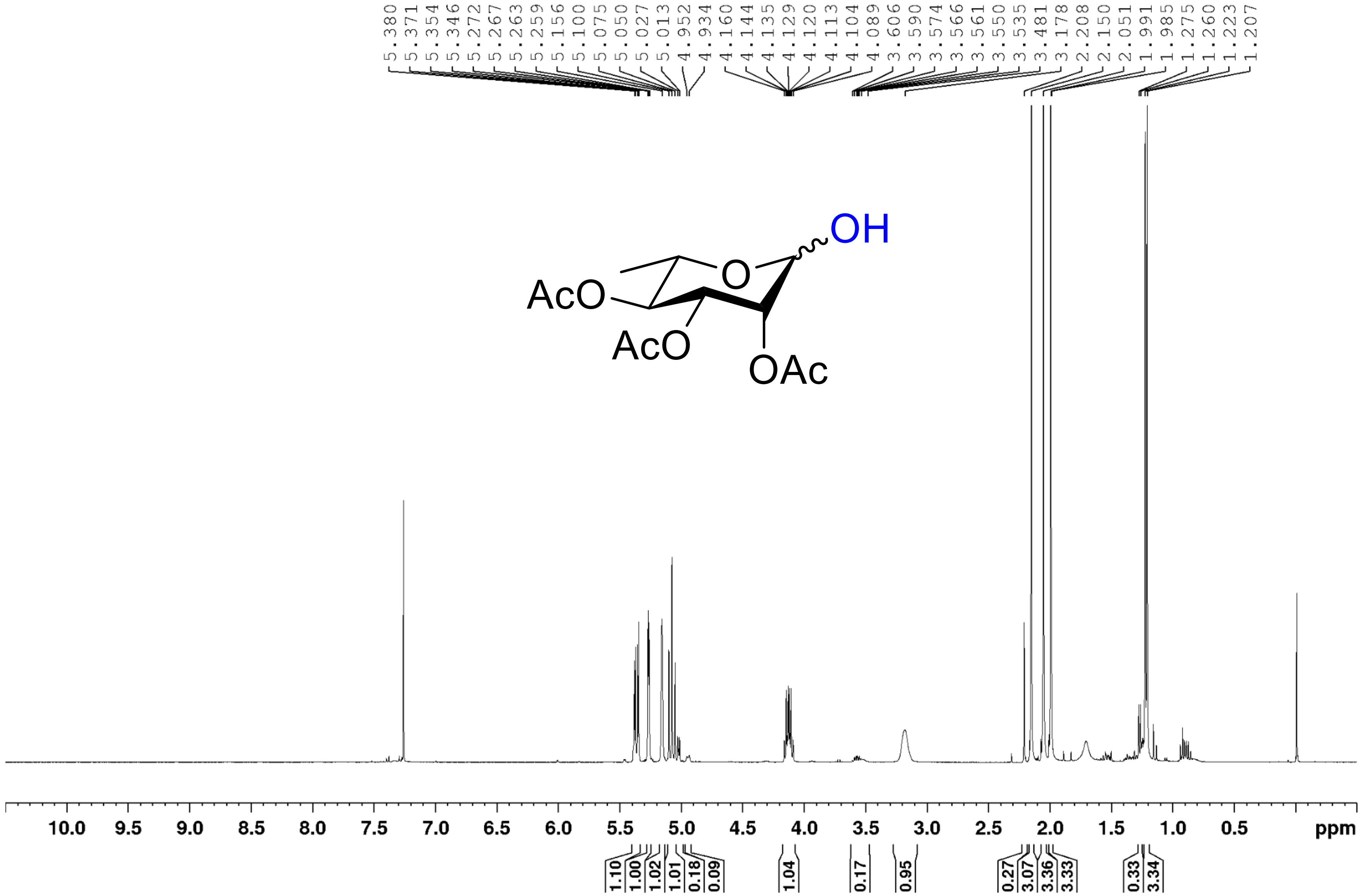

2,3,4-tri-*O*-acetyl-6-deoxy-L-mannopyranose (**33**) α:β 91:9 ^{1}H-^{1}H COSY NMR (400 MHz) in $CDCl_3$

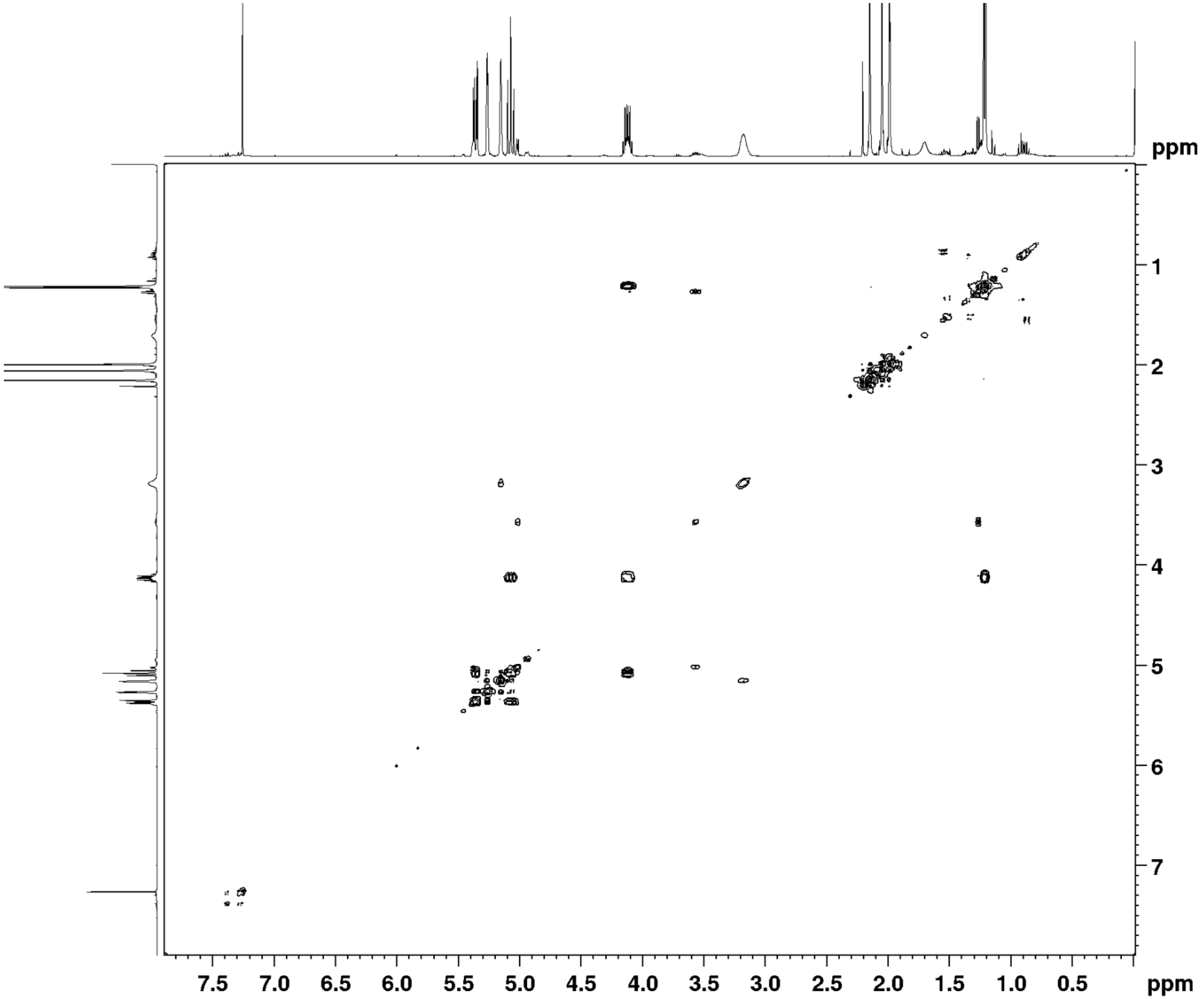

4-chlorophenyl 2,3,4-tri-*O*-acetyl-6-deoxy-1-thio-L-mannopyranoside (**34**) α:β 69:31 ^{1}H NMR (400 MHz) in $CDCl_3$

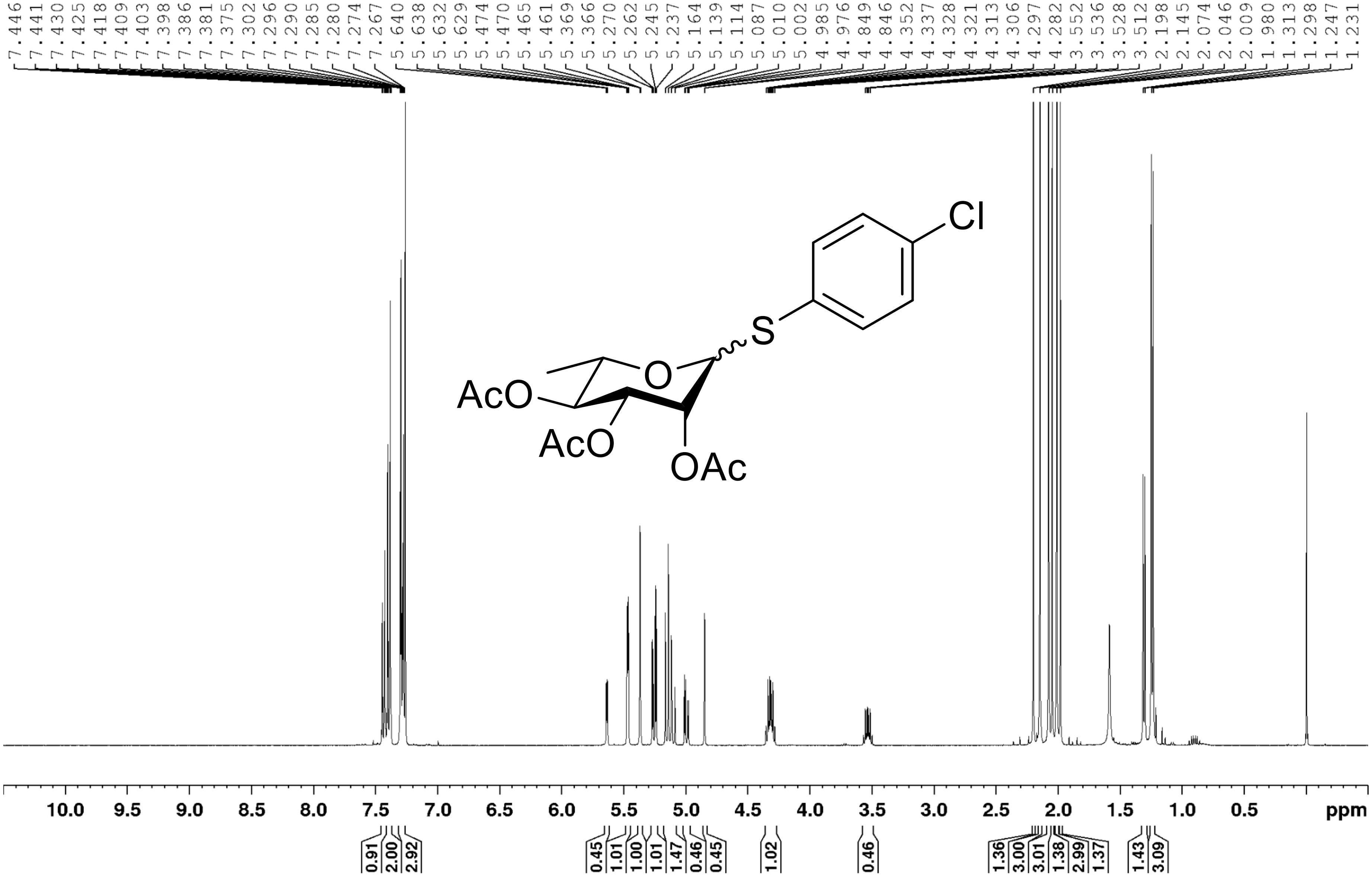

1,2,3,4,6-penta-*O*-benzoyl-D-glucopyranoside (**35**) α:β 73:27 ^{1}H NMR (400 MHz) in $CDCl_3$

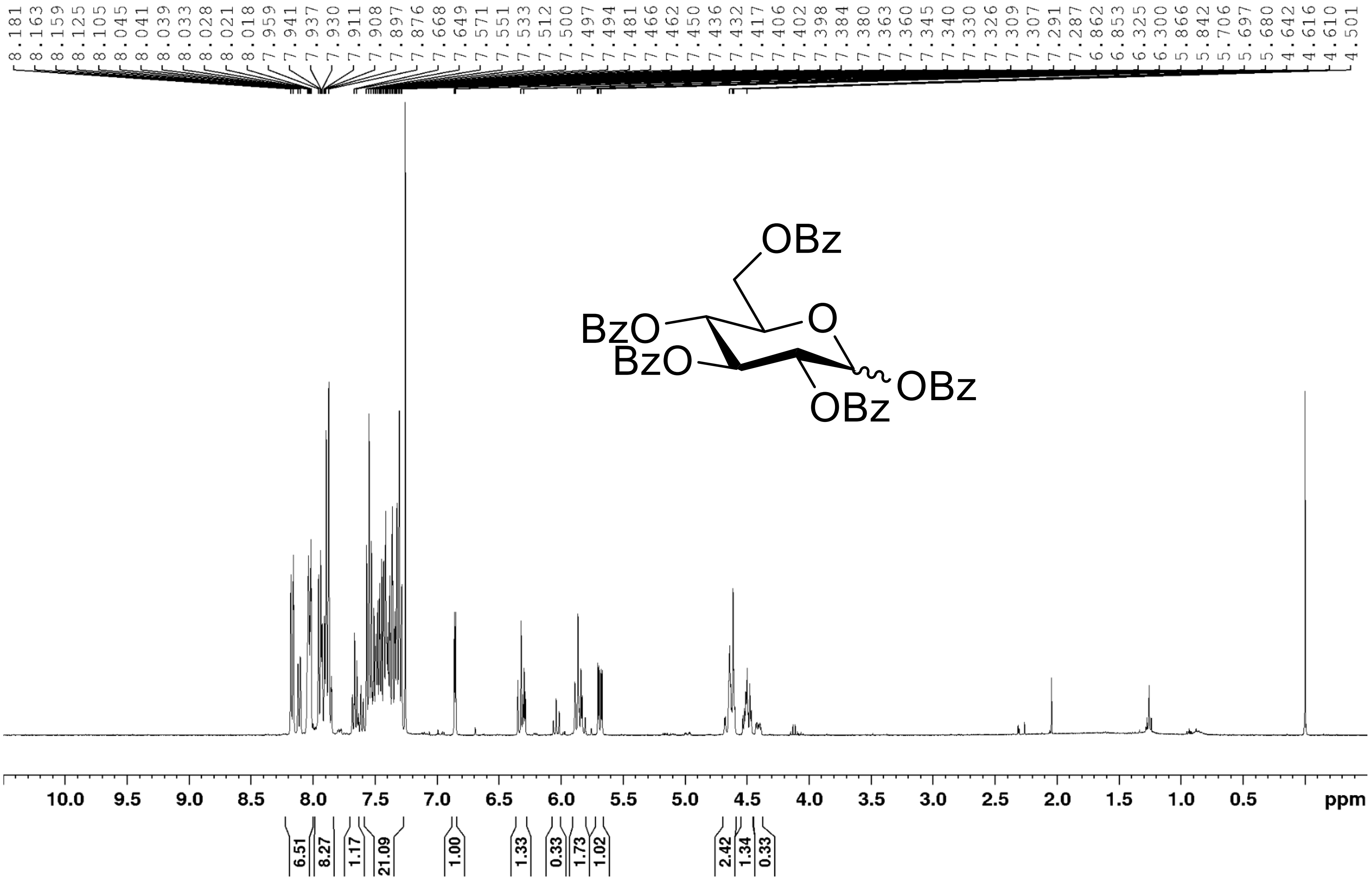

1,2,3,4-tetra-*O*-benzoyl-D-glucopyranoside (**36**) α:β 88:12 ^{1}H NMR (400 MHz) in $CDCl_3$

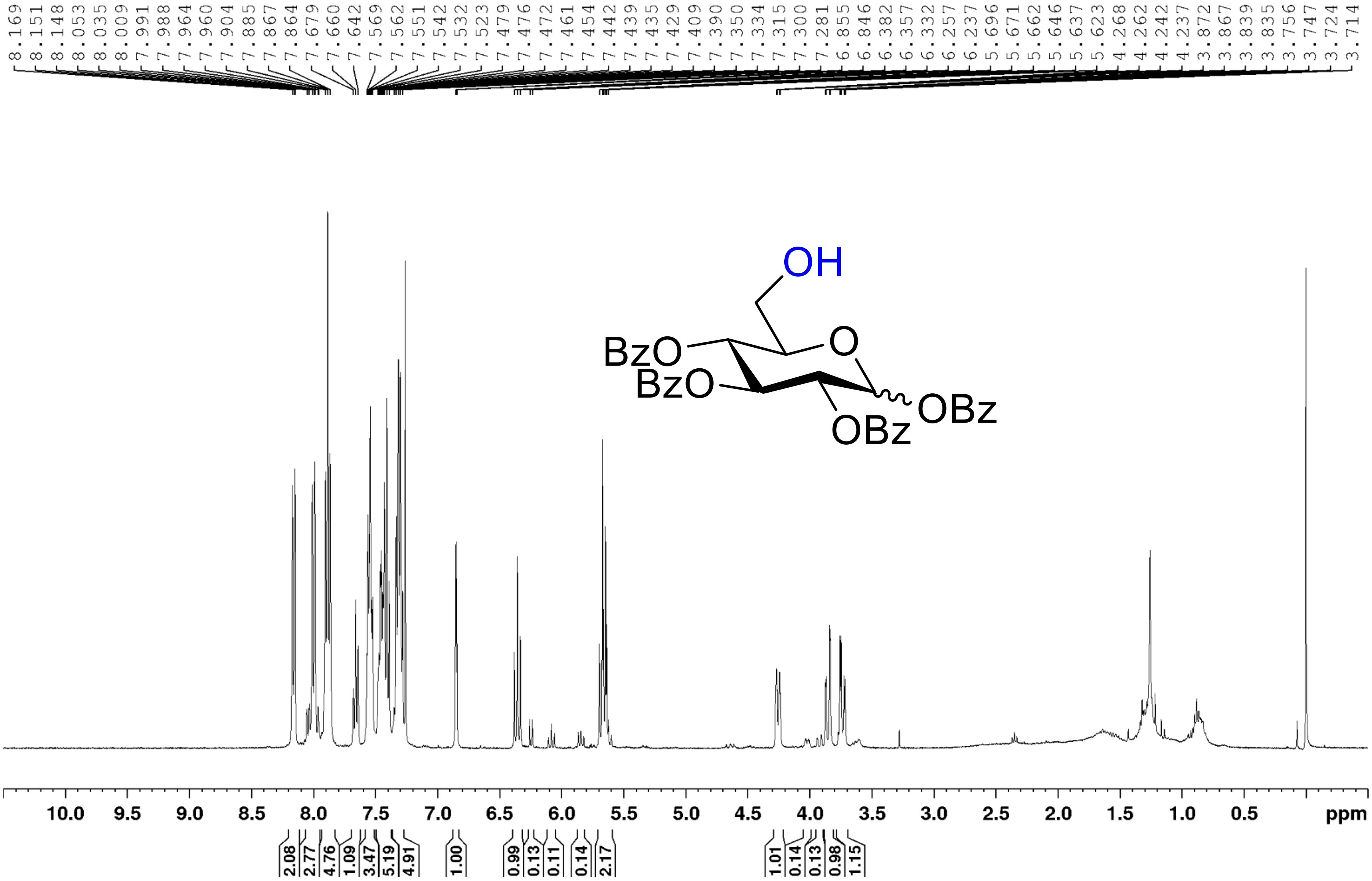

1,2,3,4-tetra-*O*-benzoyl-D-glucopyranoside (**36**) α:β 88:12 ^{1}H-^{1}H COSY NMR (400 MHz) in $CDCl_3$

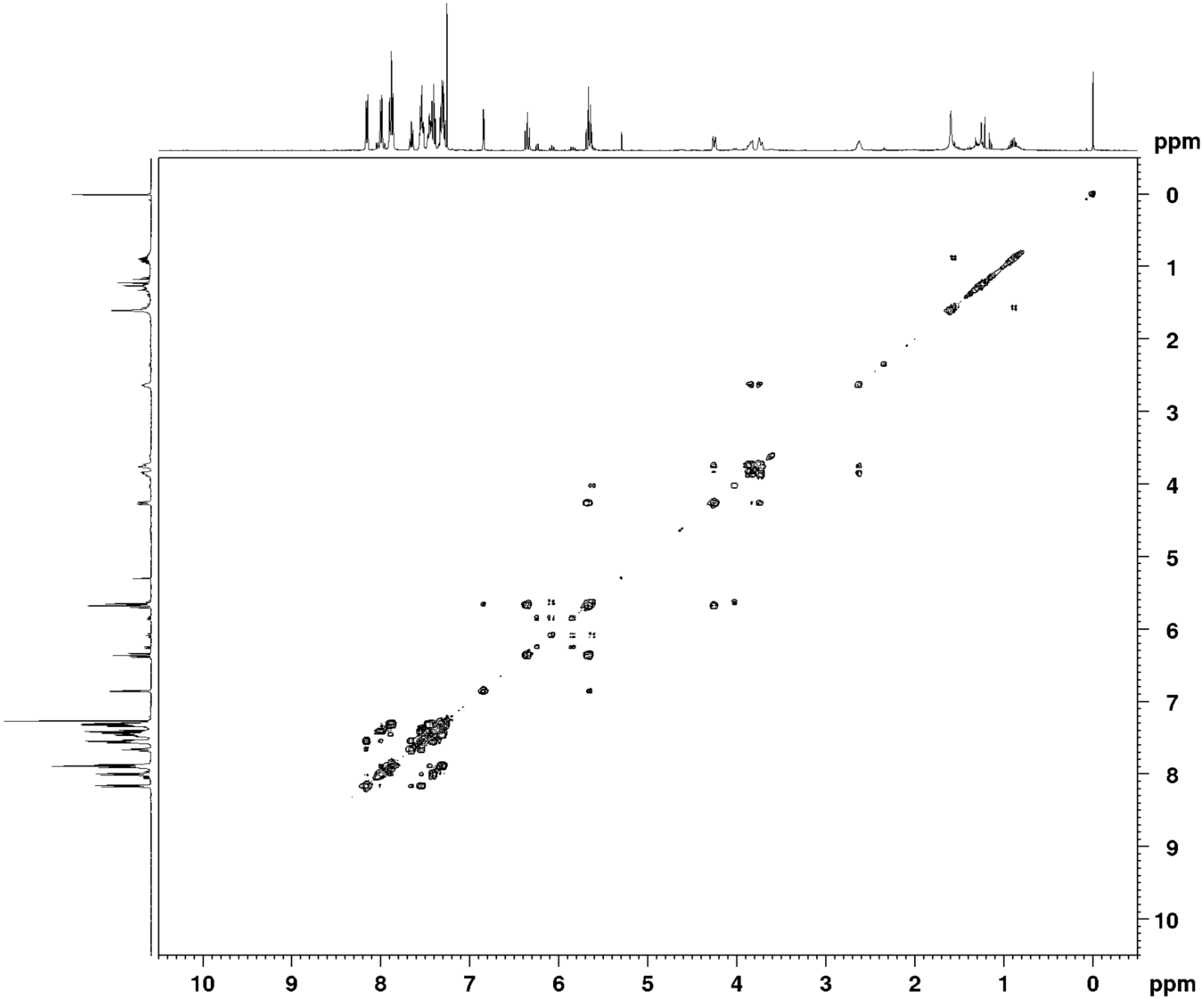

1,2,3,4-tetra-*O*-benzoyl-D-glucopyranoside (**36**) α:β 88:12 ^{1}H-$^{13}C\{^{1}H\}$ HSQC NMR (400 & 101 MHz) in $CDCl_3$

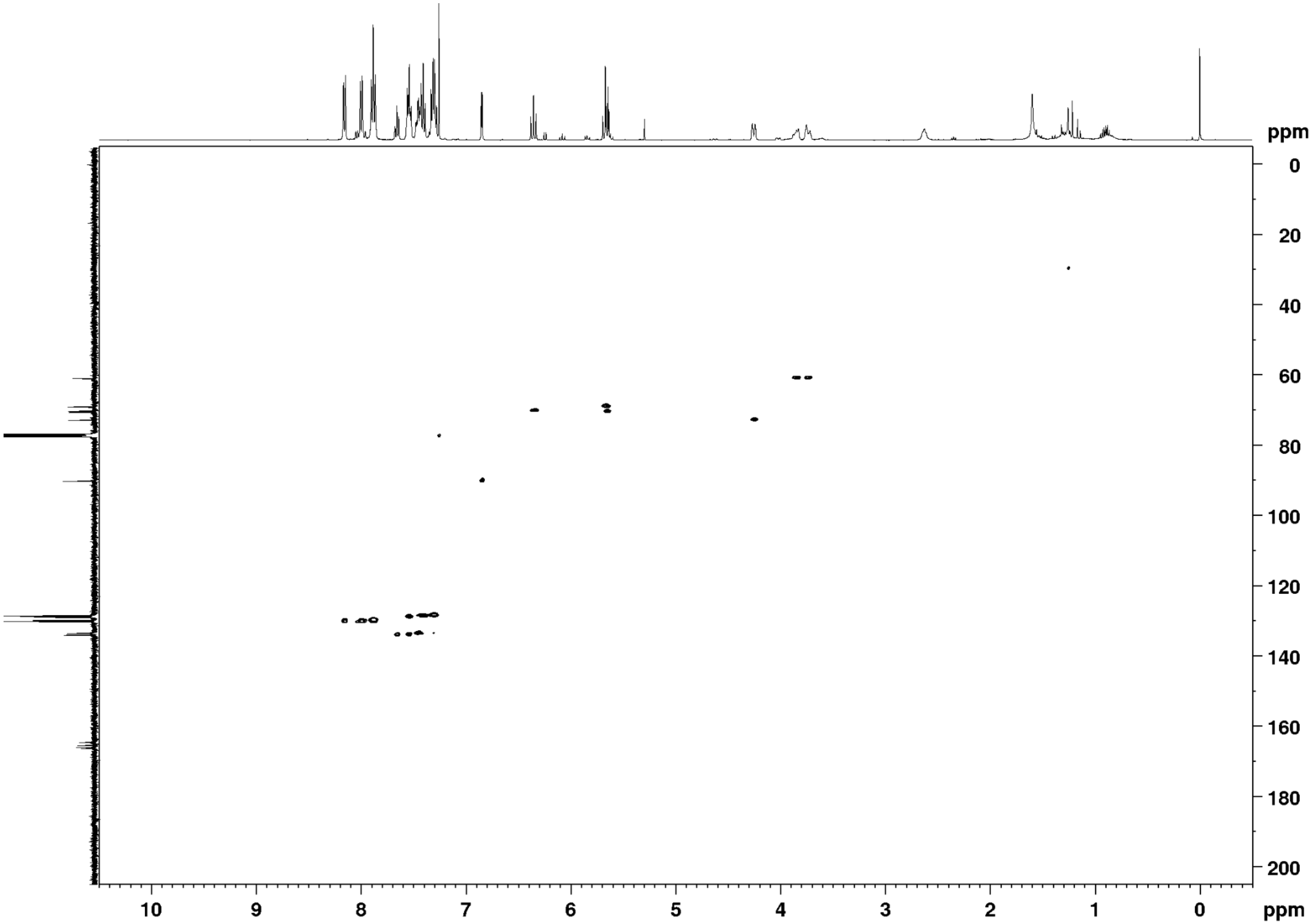

1,2,3,4-tetra-*O*-benzoyl-D-glucopyranoside (**36**) α:β 88:12 ^{1}H-$^{13}C\{^{1}H\}$ HMBC NMR (400 & 101 MHz) in $CDCl_3$

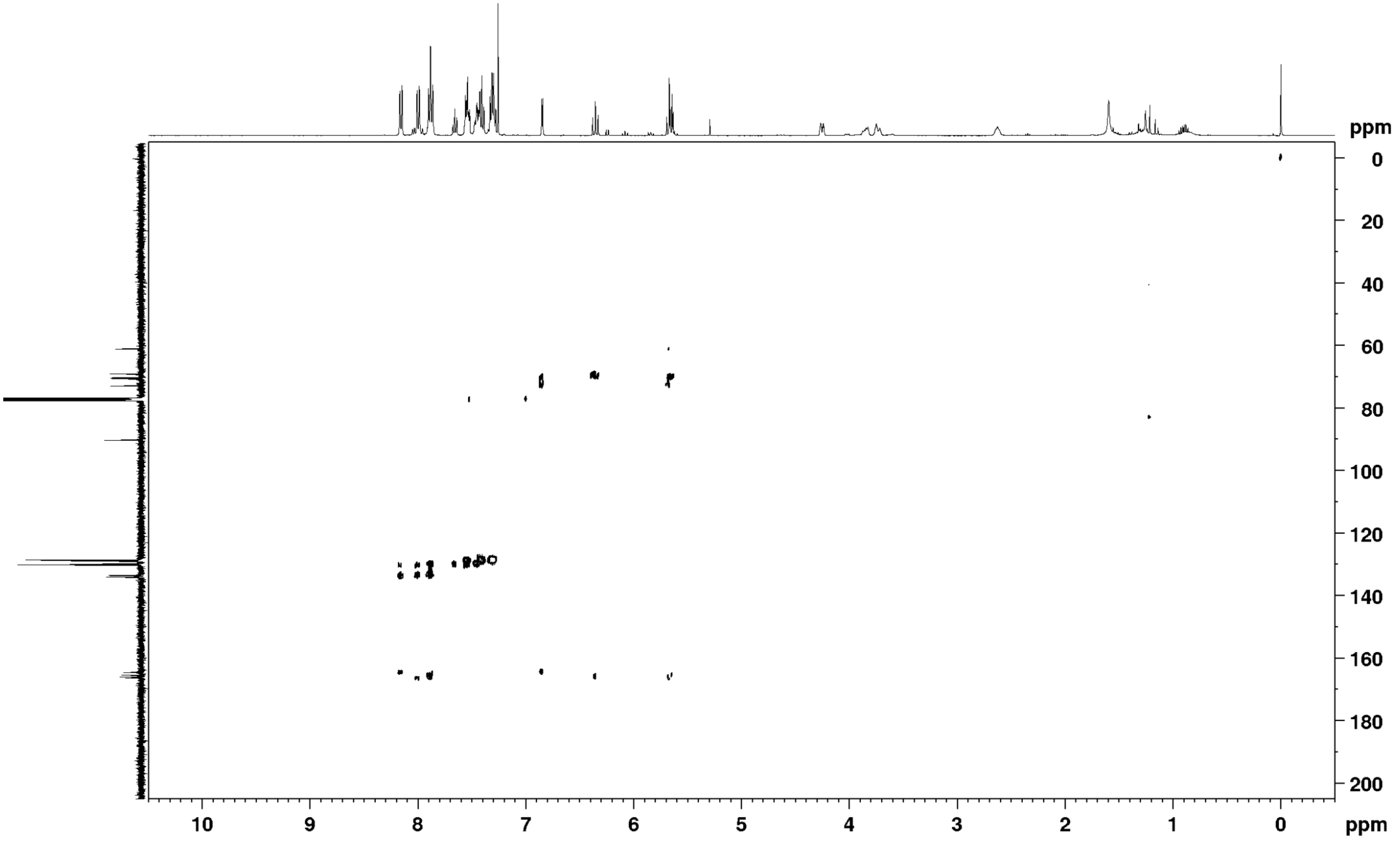

1,2,3,4,6-penta-*O*-benzoyl-D-galactopyranoside (**37**) α:β 73:27 ^{1}H NMR (400 MHz) in $CDCl_3$

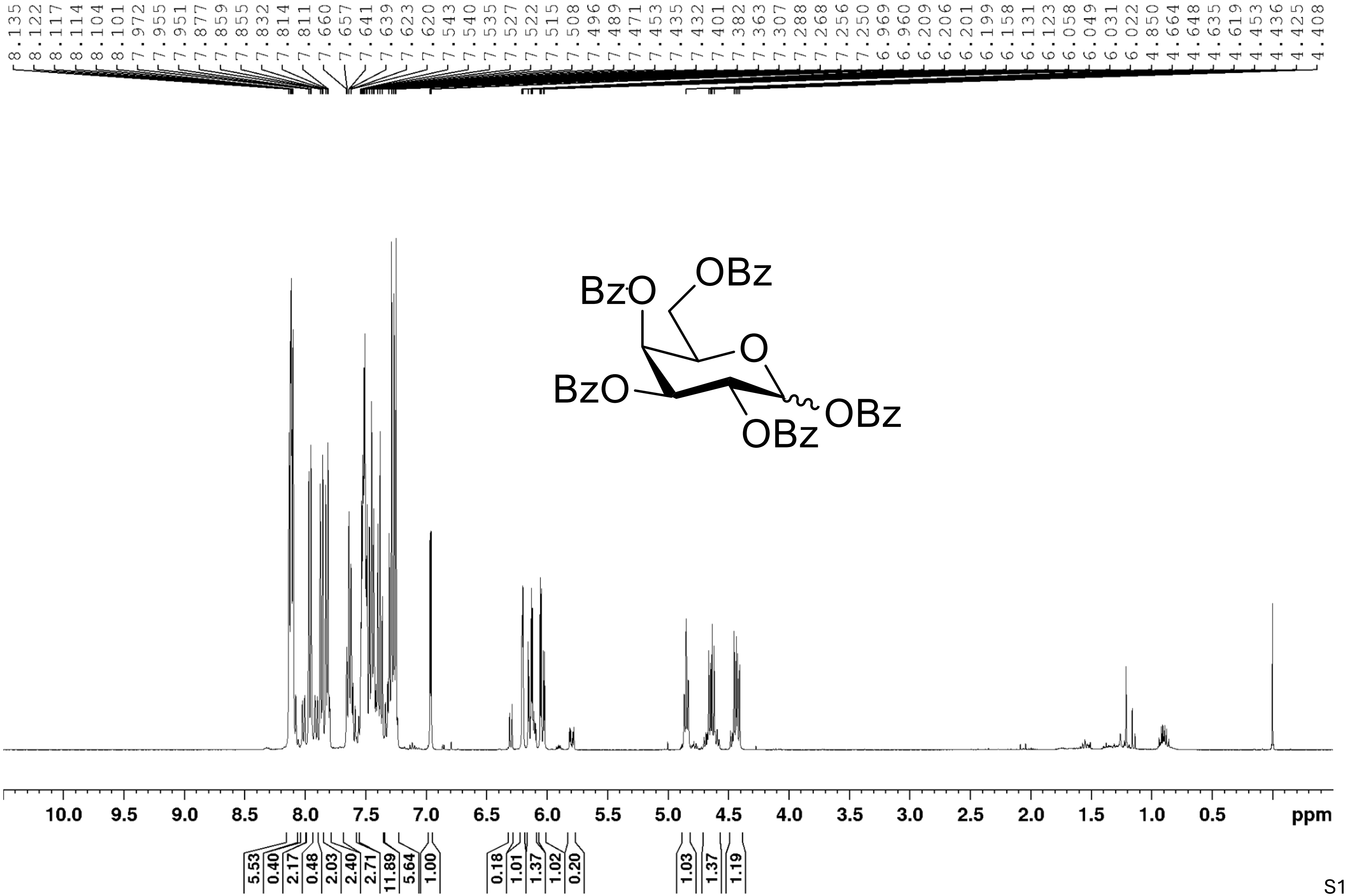

1,2,3,4,6-penta-*O*-pivaloyl-D-glucopyranoside (**38**) α:β 4:96 ^{1}H NMR (400 MHz) in $CDCl_3$

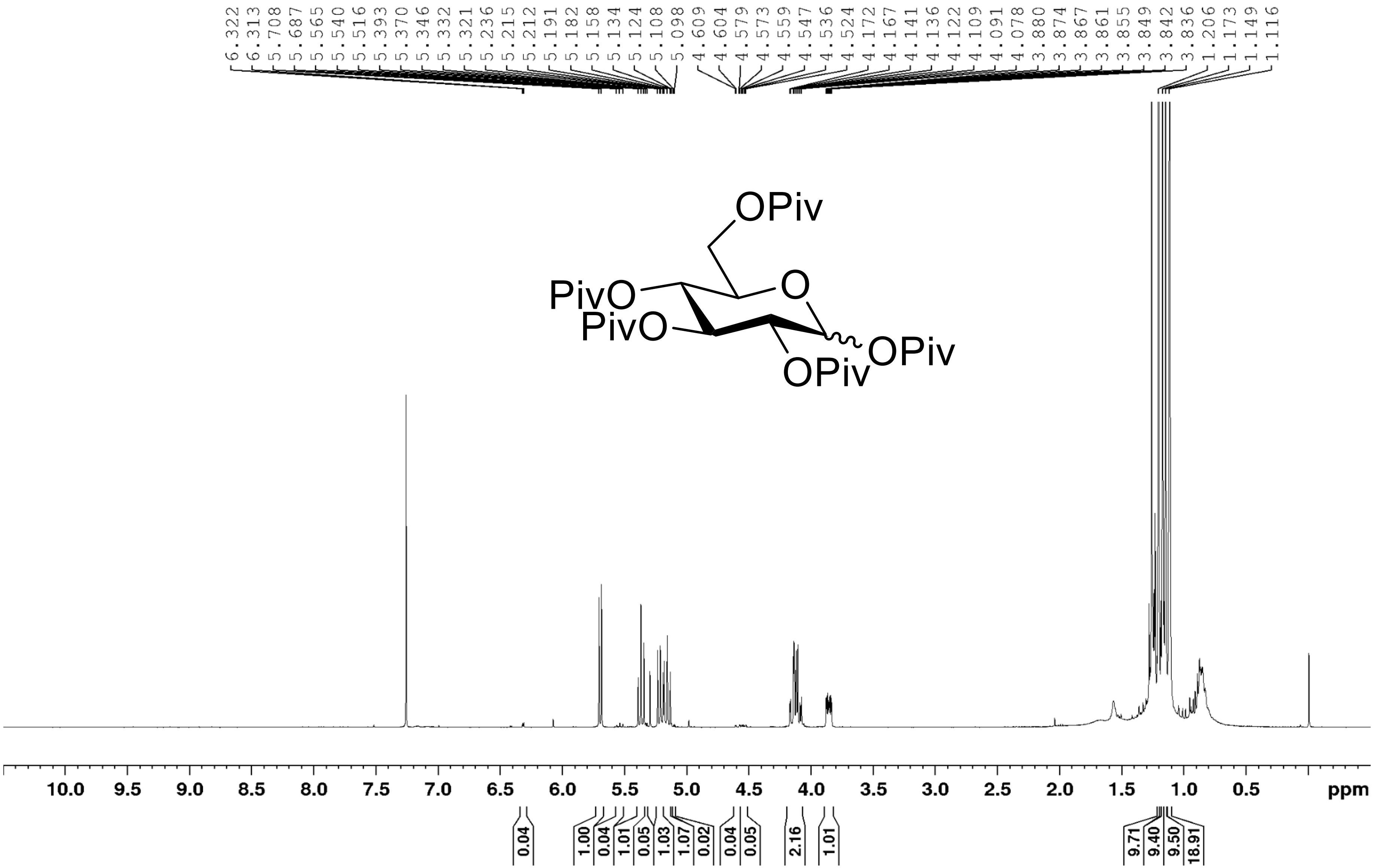

1,2,3,4,6-penta-*O*-butyryl-D-glucopyranoside (**39**) α:β 2:1 ^{1}H NMR (400 MHz) in $CDCl_3$

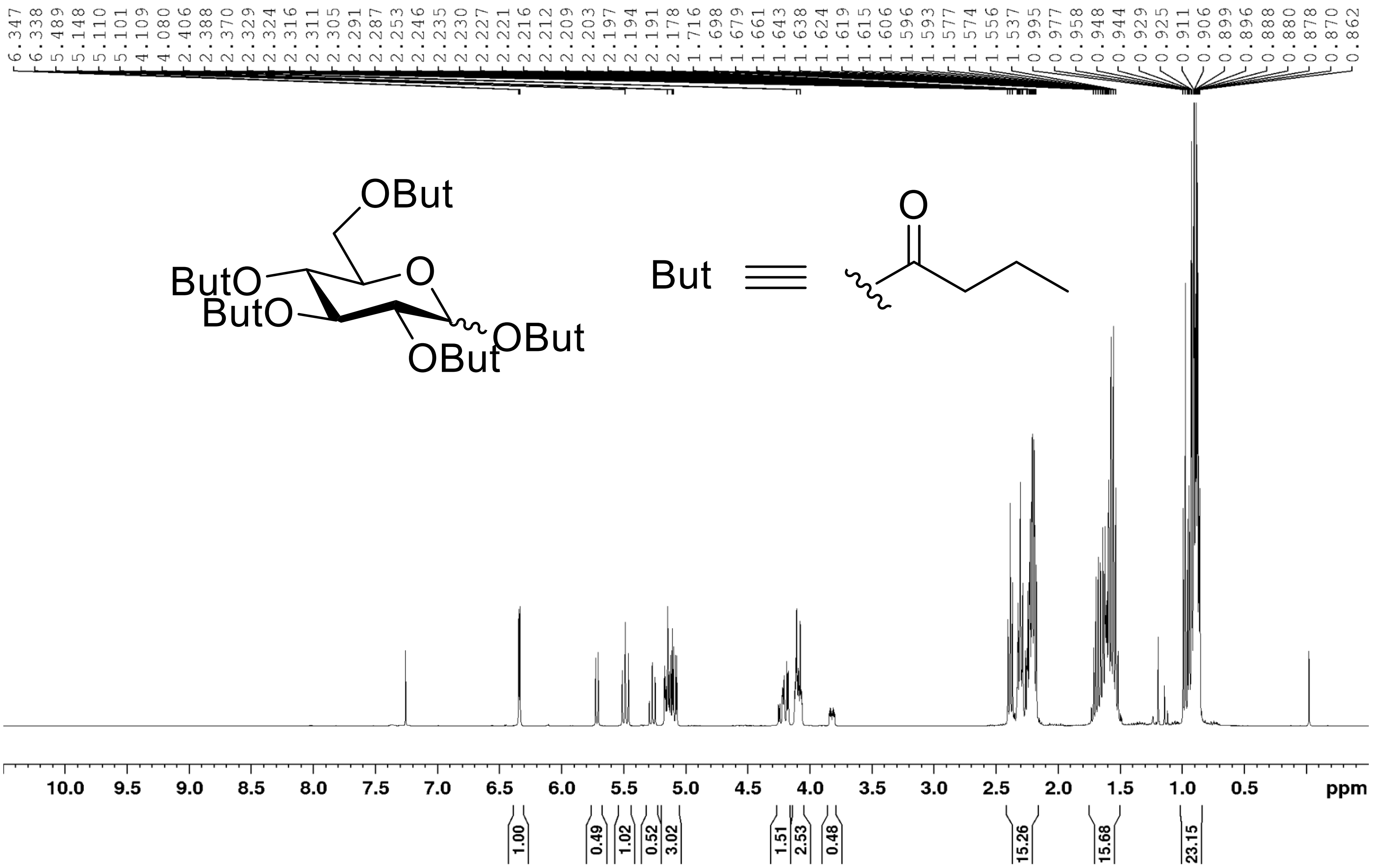

1,2,3,4-tetra-*O*-butyryl-α-D-glucopyranoside (**40**) and 2,3,4,6-tetra-*O*-butyryl-D-glucopyranoside (**41**) ^{1}H NMR (400 MHz) in $CDCl_3$

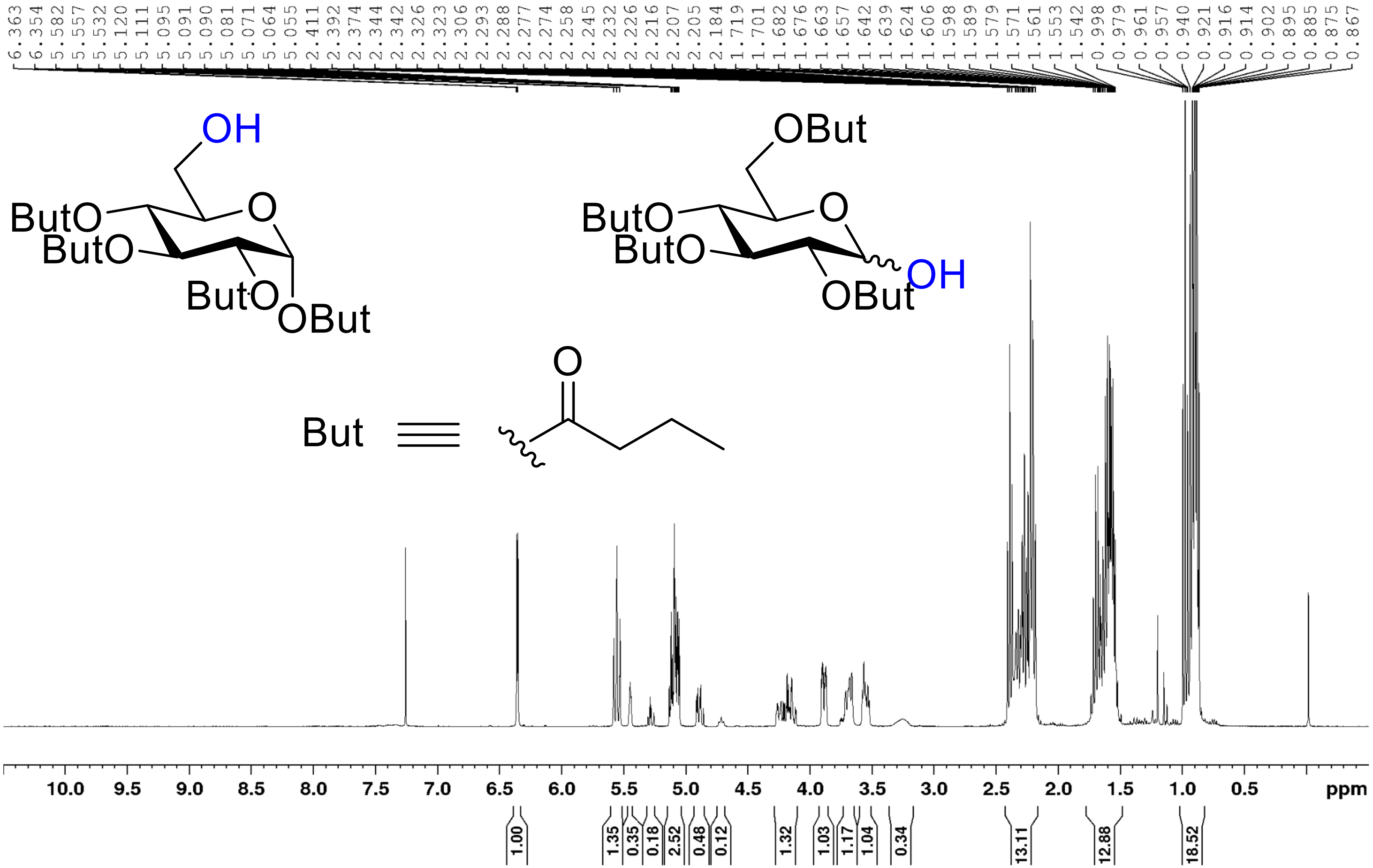

1,2,3,4-tetra-*O*-butyryl-α-D-glucopyranoside (**40**) and 2,3,4,6-tetra-*O*-butyryl-D-glucopyranoside (**41**) ^{1}H-^{1}H COSY NMR (400 MHz) in $CDCl_3$

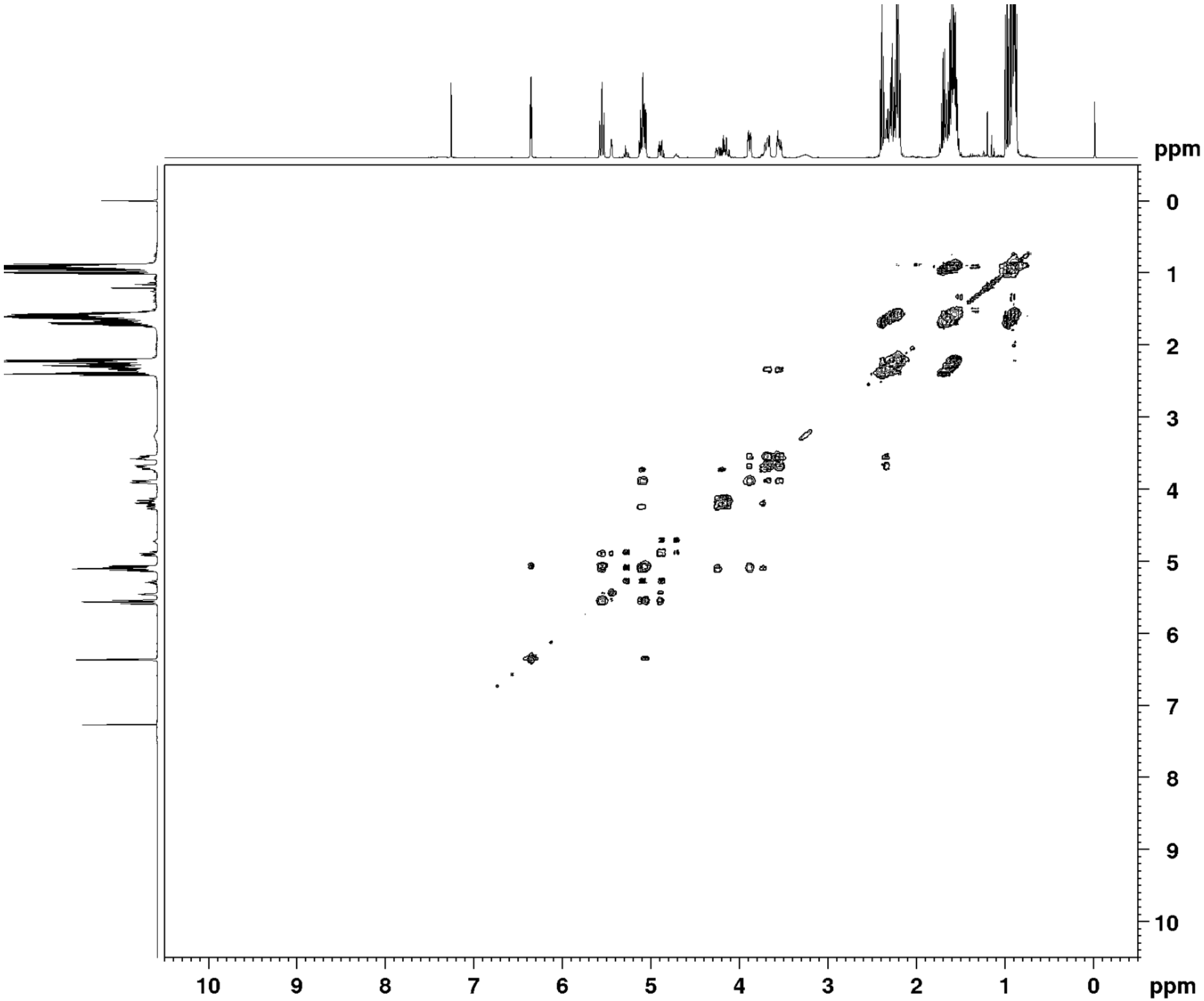

1,2,3,4-tetra-*O*-butyryl-α-D-glucopyranoside (**40**) and 2,3,4,6-tetra-*O*-butyryl-D-glucopyranoside (**41**) ^{1}H-^{1}H NOESY NMR (400 MHz) in $CDCl_3$

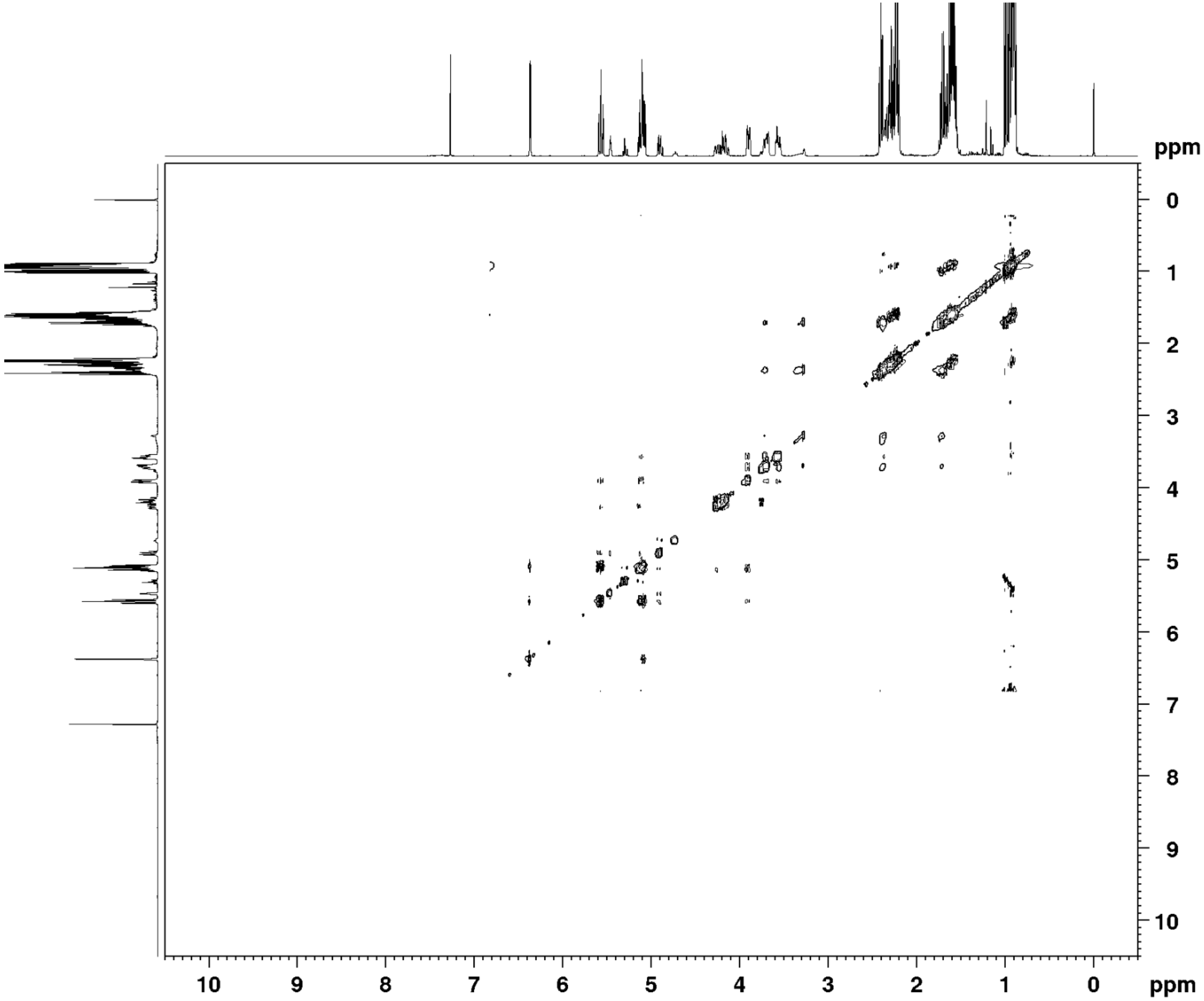

1,2,3,4-tetra-*O*-butyryl-α-D-glucopyranoside (**40**) and 2,3,4,6-tetra-*O*-butyryl-D-glucopyranoside (**41**) ^{1}H-^{13}C{^{1}H} HSQC NMR (400 & 101 MHz) in $CDCl_3$

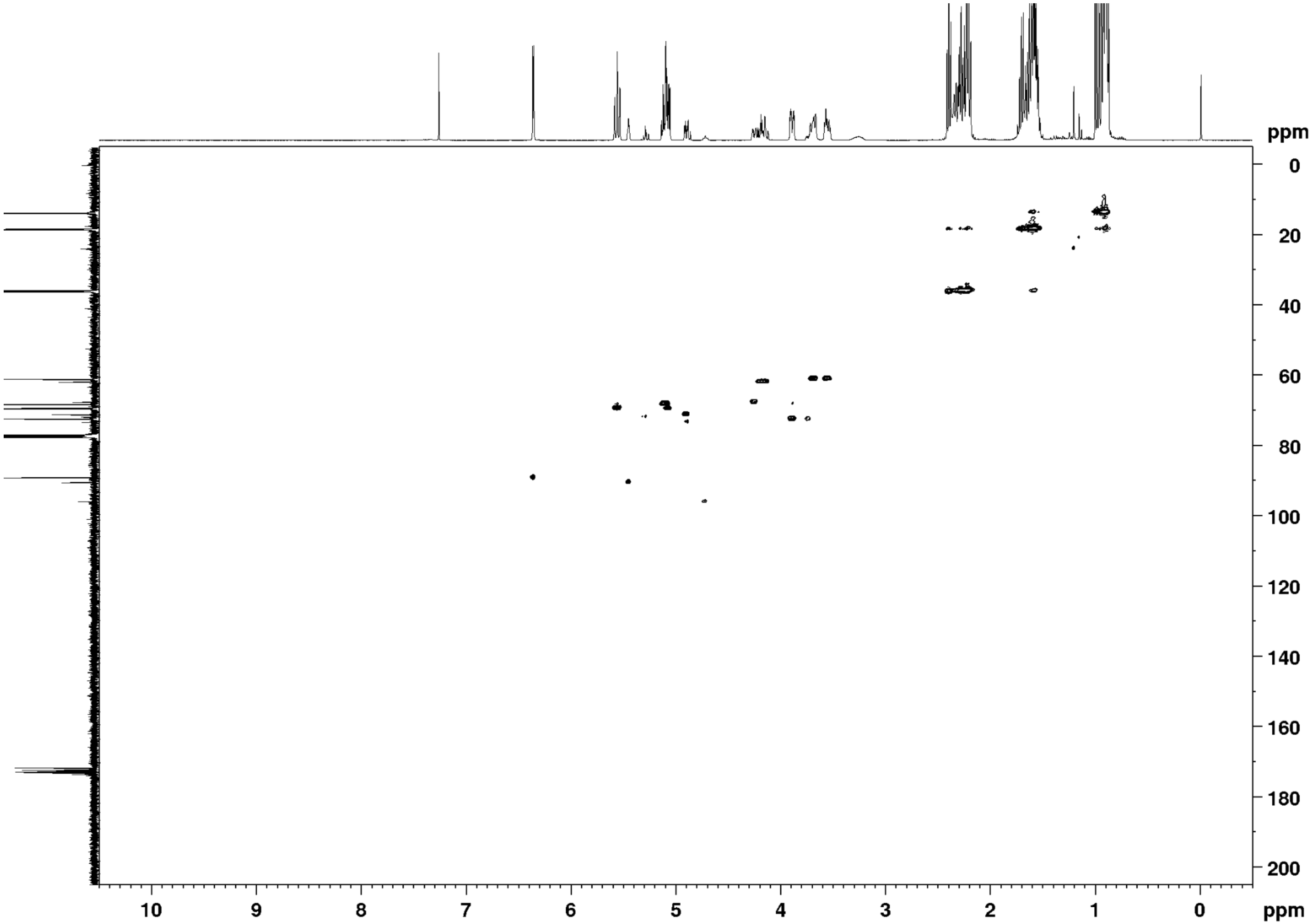

1,2,3,4-tetra-*O*-butyryl-α-D-glucopyranoside (**40**) and 2,3,4,6-tetra-*O*-butyryl-D-glucopyranoside (**41**) ^{1}H-^{13}C{^{1}H} HMBC NMR (400 & 101 MHz) in $CDCl_3$

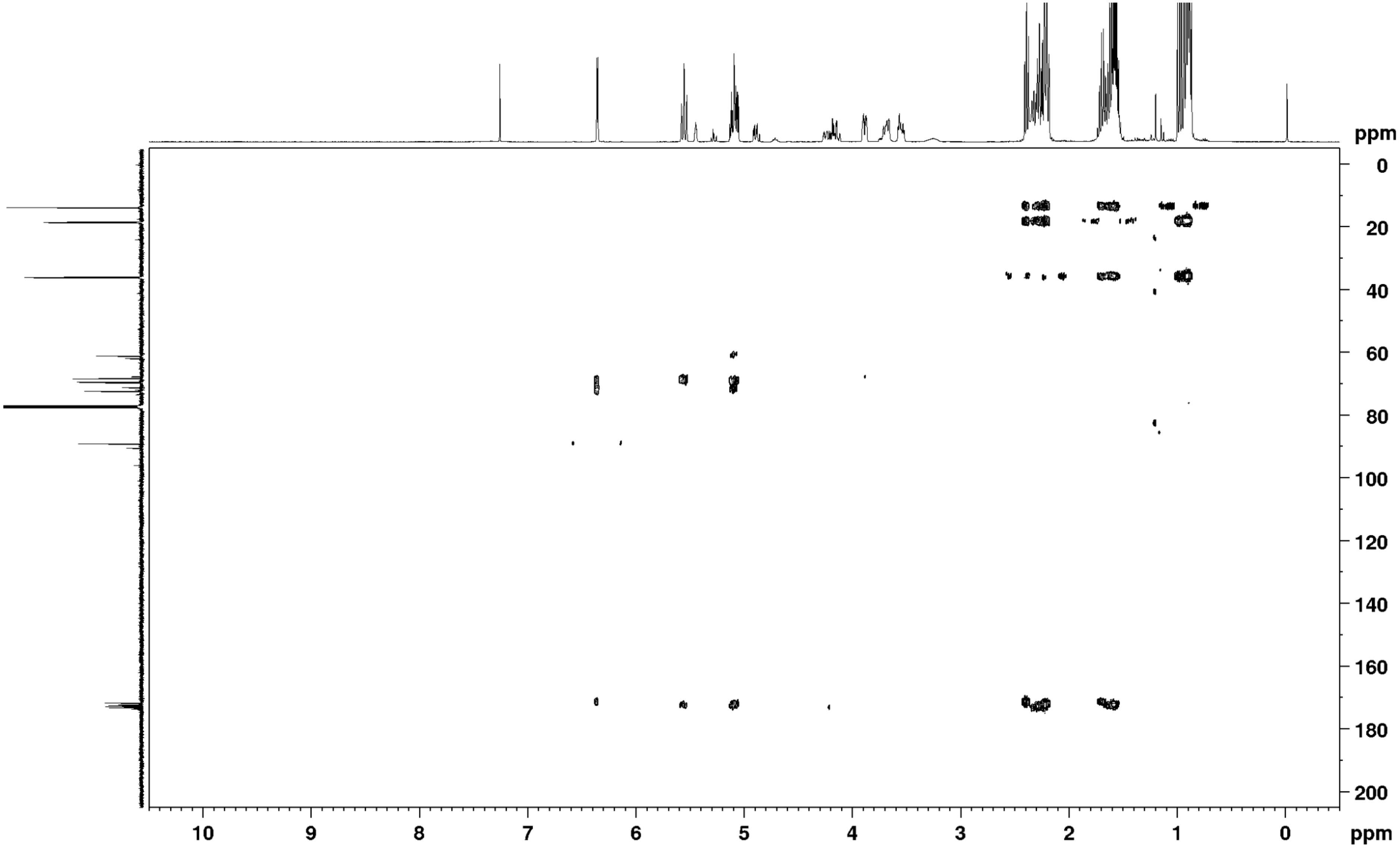

1,2,3,4-tetra-*O*-butyryl-α-D-glucopyranoside (**40**) and 2,3,4,6-tetra-*O*-butyryl-D-glucopyranoside (**41**) $^{13}C\{^{1}H\}$ NMR (101 MHz) in $CDCl_3$

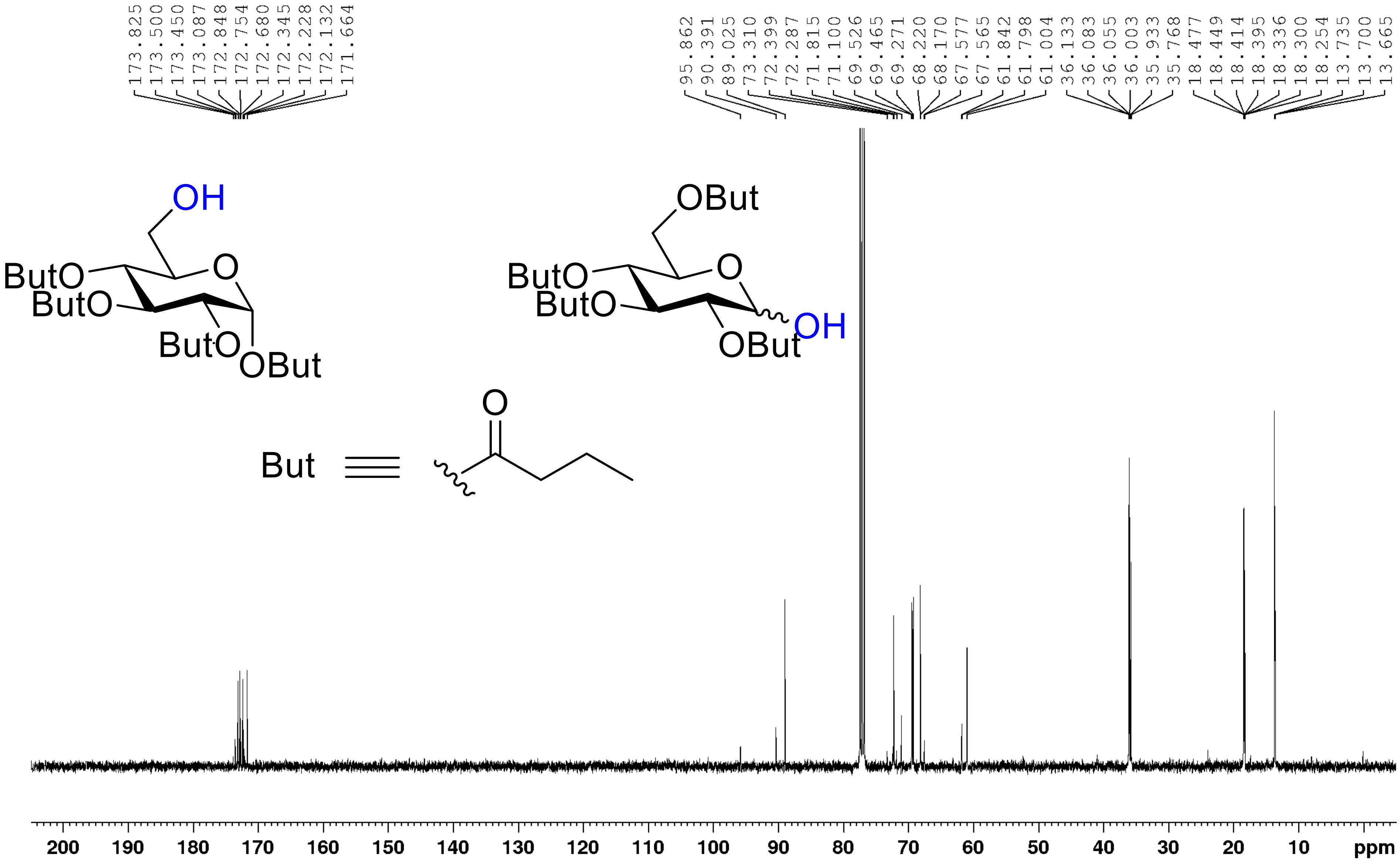

1,2,3-tri-*O*-acetyl-α-D-glucopyranoside (**43**) ^{1}H NMR (400 MHz) in $CDCl_3$

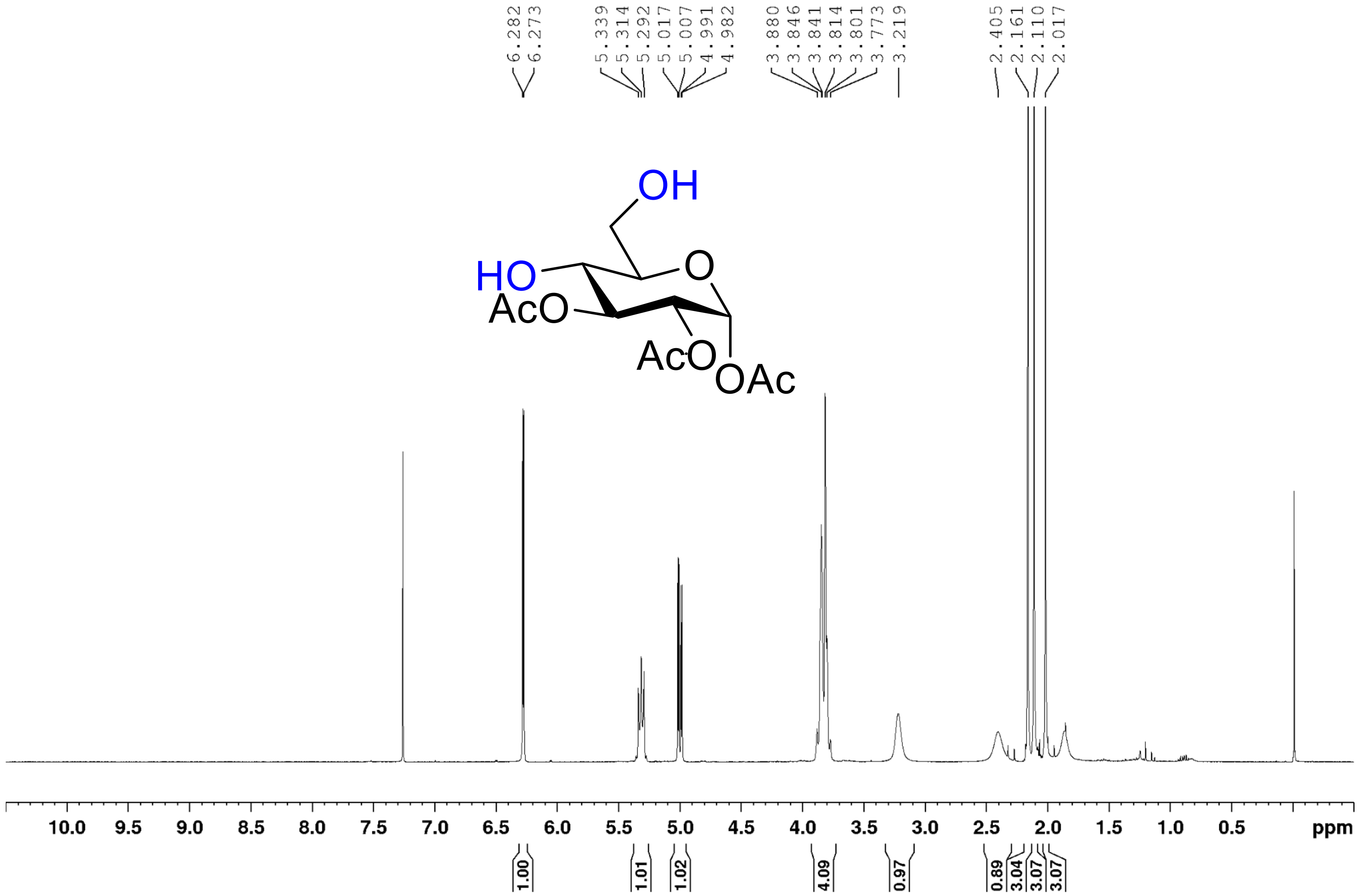

1,2,3-tri-*O*-acetyl-α-d-glucopyranoside (**43**) ^{1}H-^{1}H COSY NMR (400 MHz) in $CDCl_3$

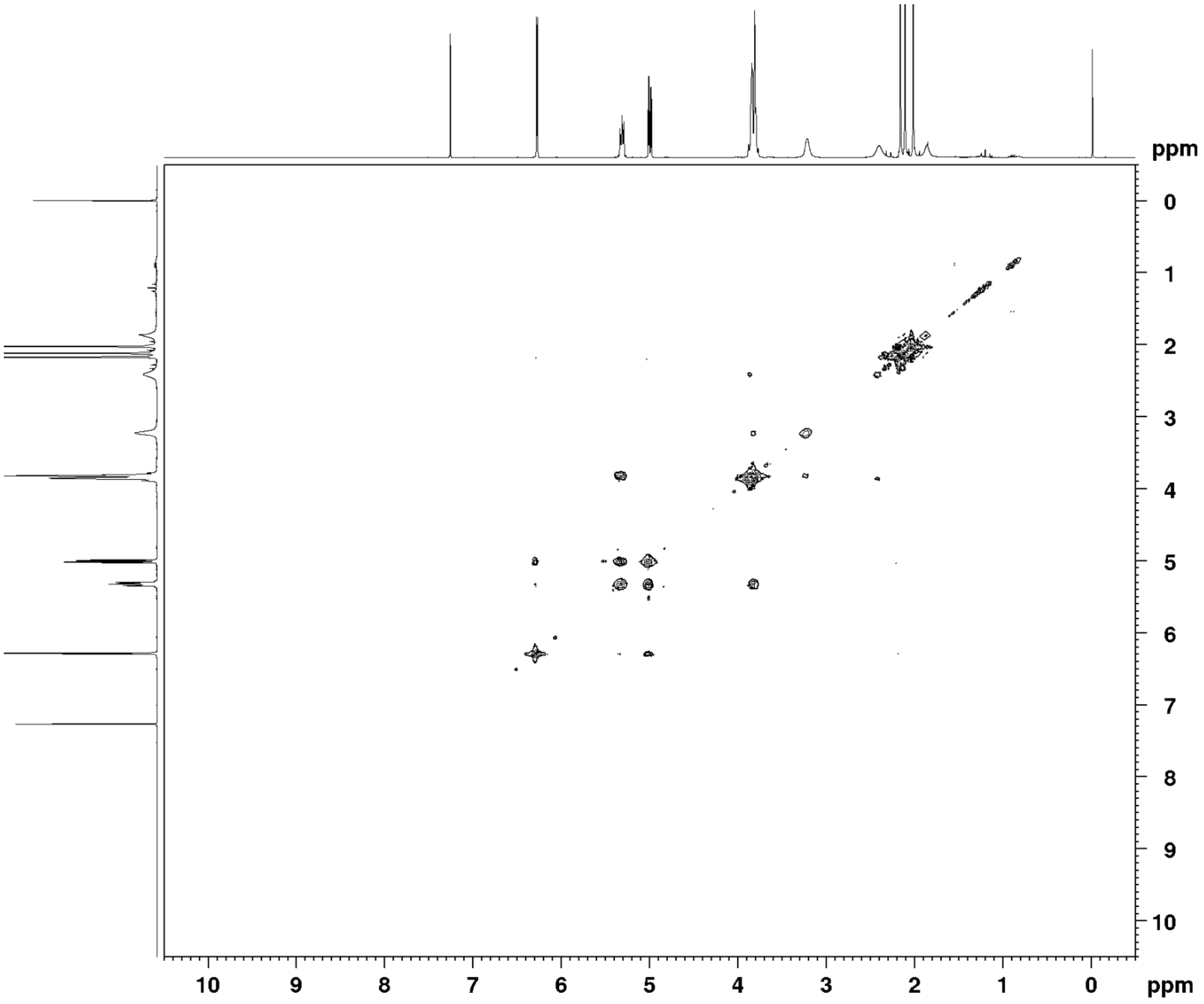

Diphenyl 2,4,6-tri-*O*-α-D-galactopyranosyl phosphate (S1), diphenyl 2,3,6-tri-*O*-α-D-galactopyranosyl phosphate (S2) and **diphenyl 2,3,4-tri-*O*-α-D-galactopyranosyl phosphate** (**S3**); **S1**:**S2**:**S3** 10:10:1 ^{1}H NMR (400 MHz) in $CDCl_3$

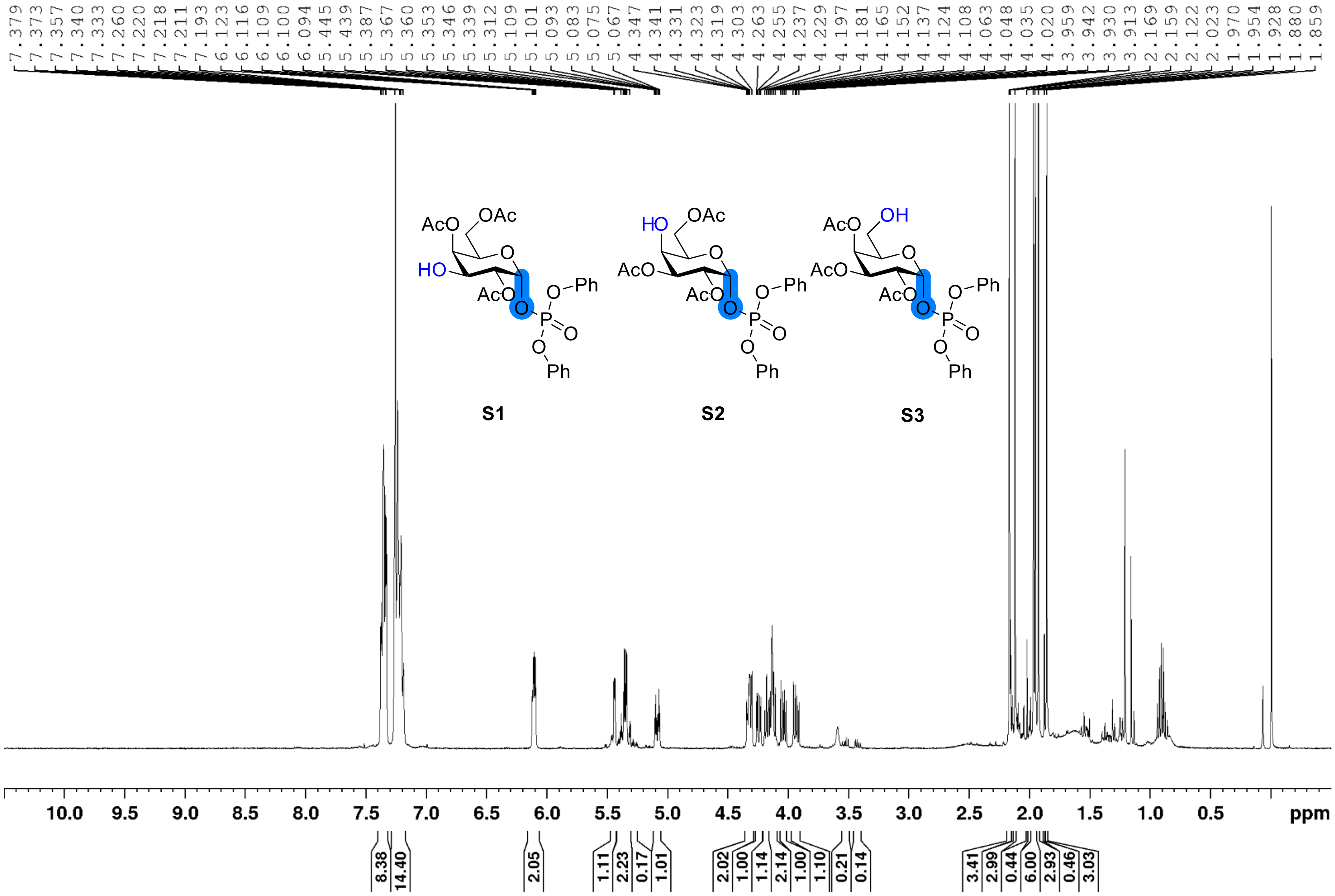

Diphenyl 2,4,6-tri-*O*-α-D-galactopyranosyl phosphate (S1), diphenyl 2,3,6-tri-*O*-α-D-galactopyranosyl phosphate (S2) and **diphenyl 2,3,4-tri-*O*-α-D-galactopyranosyl phosphate** (**S3**); **S1**:**S2**:**S3** 10:10:1 ^{1}H-^{1}H COSY NMR (400 MHz) in $CDCl_3$

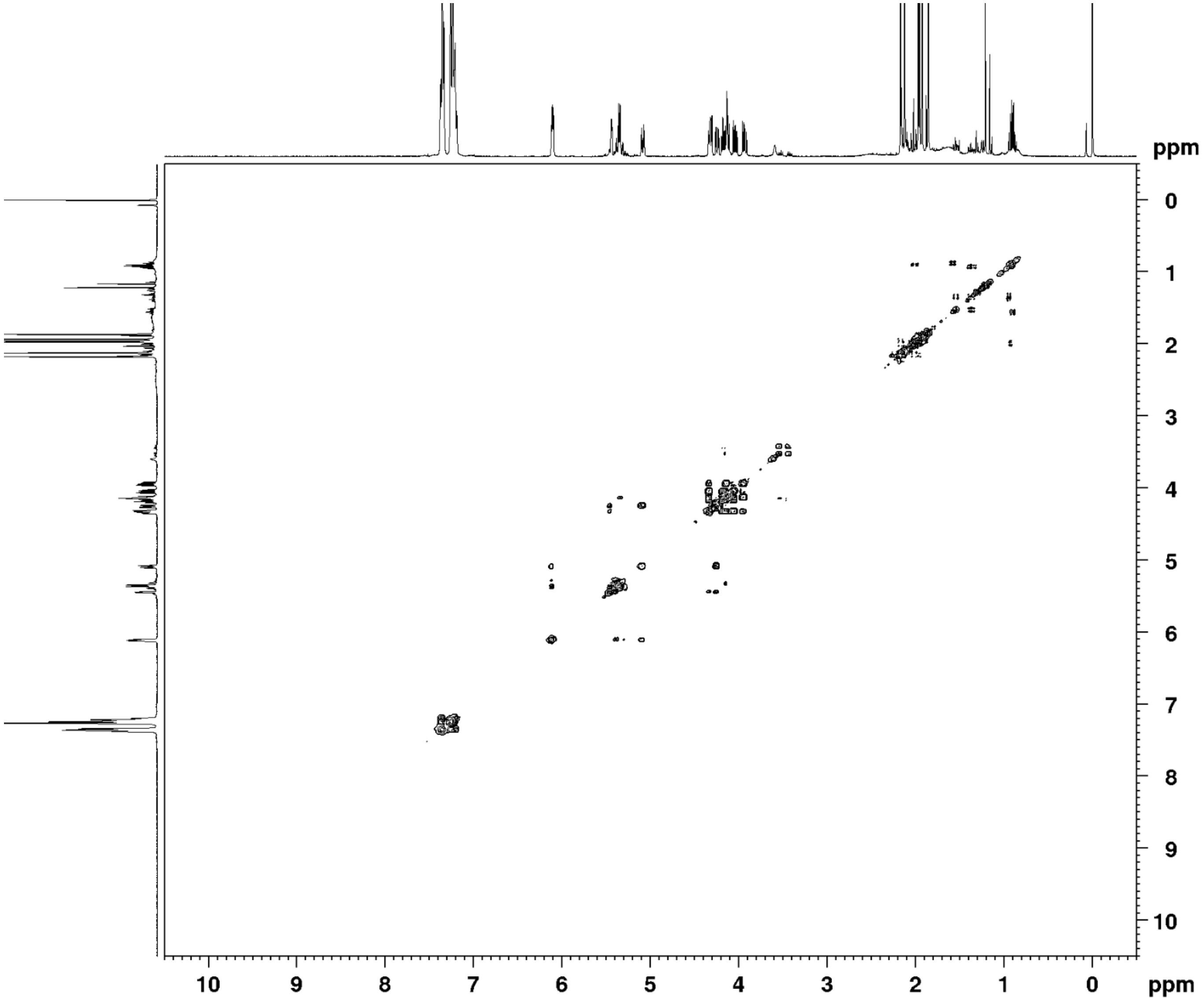

Diphenyl 2,4,6-tri-*O*-α-D-galactopyranosyl phosphate (S1), diphenyl 2,3,6-tri-*O*-α-D-galactopyranosyl phosphate (S2) and **diphenyl 2,3,4-tri-*O*-α-D-galactopyranosyl phosphate** (**S3**); **S1**:**S2**:**S3** 10:10:1 ^{1}H-^{13}C{^{1}H} HSQC NMR (400 & 101 MHz) in $CDCl_3$

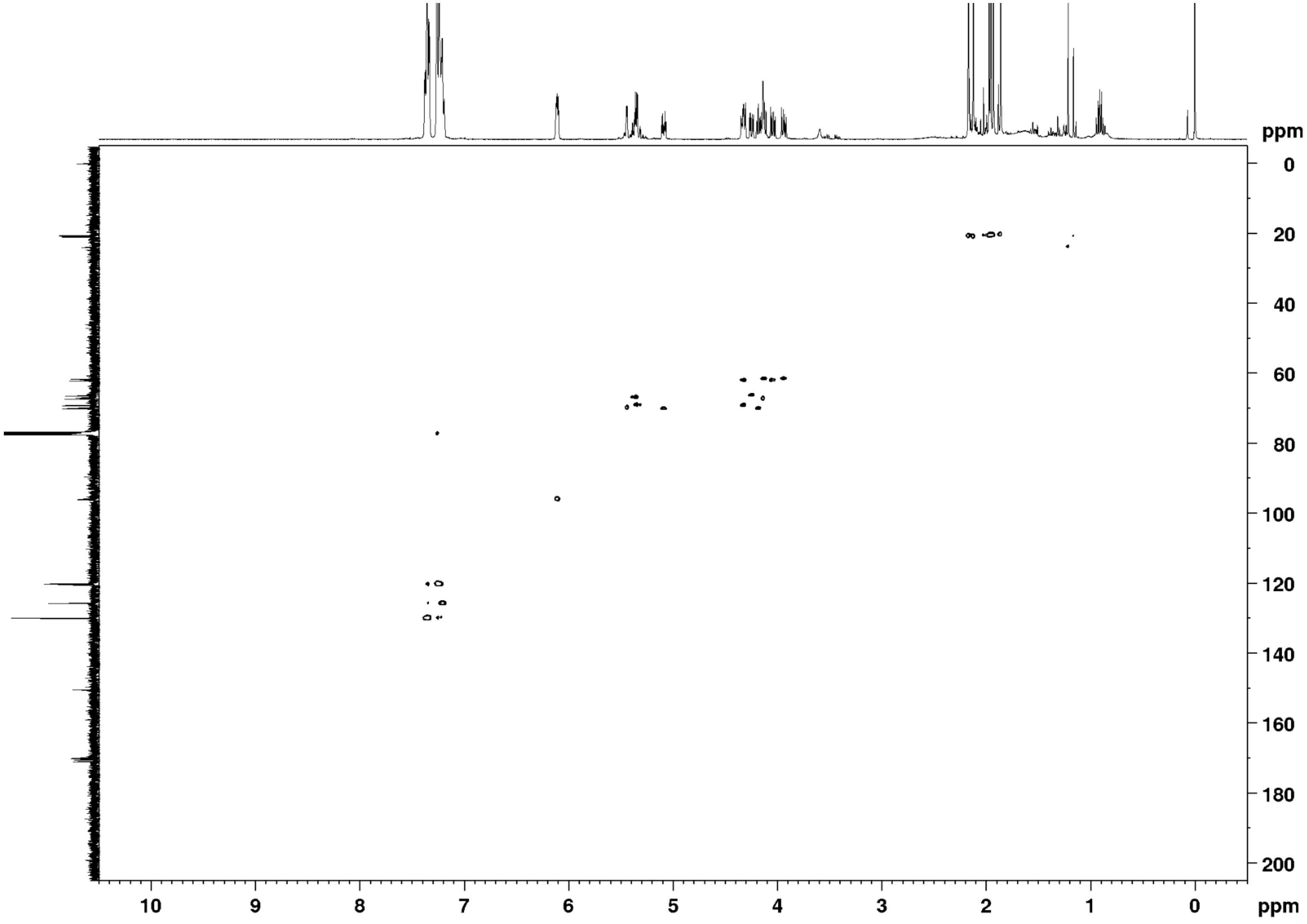

Diphenyl 2,4,6-tri-*O*-α-D-galactopyranosyl phosphate (S1), diphenyl 2,3,6-tri-*O*-α-D-galactopyranosyl phosphate (S2) and **diphenyl 2,3,4-tri-*O*-α-D-galactopyranosyl phosphate** (**S3**); **S1**:**S2**:**S3** 10:10:1 ^{1}H-$^{13}C\{^{1}H\}$ HMBC NMR (400 & 101 MHz) in $CDCl_3$

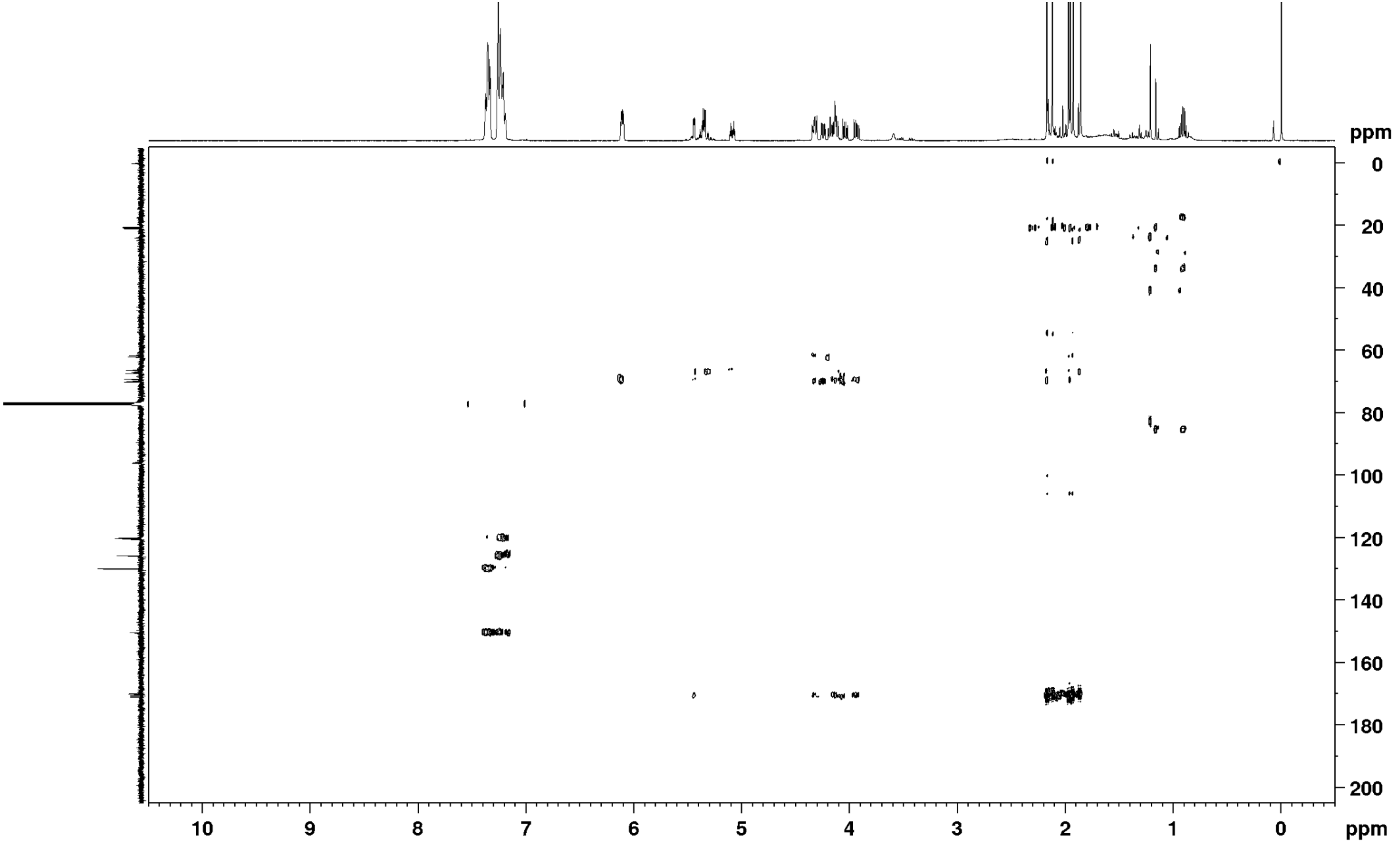

Diphenyl 2,4,6-tri-*O*-α-D-galactopyranosyl phosphate (S1), diphenyl 2,3,6-tri-*O*-α-D-galactopyranosyl phosphate (S2) and **diphenyl 2,3,4-tri-*O*-α-D-galactopyranosyl phosphate** (**S3**); **S1**:**S2**:**S3** 10:10:1 $^{13}C\{^1H\}$ NMR (101 MHz) in $CDCl_3$

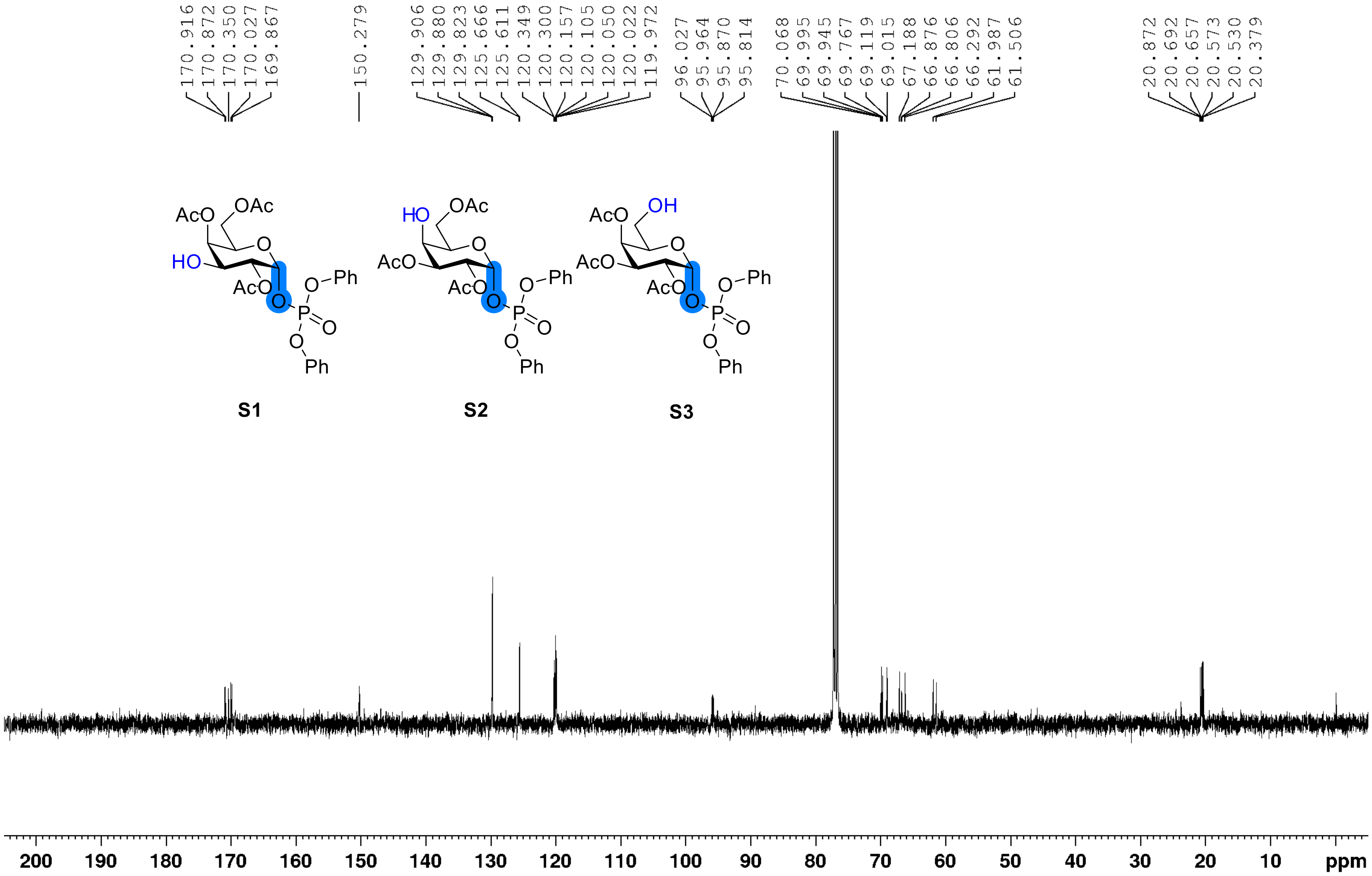

References

(1) Painter, E. P. The Anomerization of Sugar Acetates. Glucose Pentaacetates in Acetic Acid—Acetic Anhydride Mixtures Catalyzed by Sulfuric and Perchloric Acids. *J. Am. Chem. Soc.* **1953**, *75* (5), 1137–1146. https://doi.org/10.1021/ja01101a039.

(2) Bonner, W. A. The Acid-Catalyzed Anomerization of the D-Glucose Pentaacetates. A Kinetic Thermodynamic and Mechanistic Study. *J. Am. Chem. Soc.* **1951**, *73* (6), 2659–2666. https://doi.org/10.1021/ja01150a070.

(3) Chang, C.-W.; Chang, S.-S.; Chao, C.-S.; Mong, K.-K. T. A Mild and General Method for Preparation of α-Glycosyl Chlorides. *Tetrahedron Lett.* **2009**, *50* (31), 4536–4540. https://doi.org/10.1016/j.tetlet.2009.05.077.

(4) Wang, Q.; Fu, J.; Zhang, J. A Facile Preparation of Peracylated α-Aldopyranosyl Chlorides with Thionyl Chloride and Tin Tetrachloride. *Carbohydr. Res.* **2008**, *343* (17), 2989–2991. https://doi.org/10.1016/j.carres.2008.08.037.

(5) Crich, D.; Wu, B. Stereoselective Iterative One-Pot Synthesis of *N* -Glycolylneuraminic Acid-Containing Oligosaccharides. *Org. Lett.* **2008**, *10* (18), 4033–4035. https://doi.org/10.1021/ol801548k.

(6) Tsvetkov, Y. E.; Byramova, N. É.; Backinowsky, L. V. A Simple Preparation of Aromatic 1-Thioglycosides. *Carbohydr. Res.* **1983**, *115*, 254–258. https://doi.org/10.1016/0008-6215(83)88156-7.

(7) Agoston, K.; Hederos, M. J.; Bajza, I.; Dekany, G. Kilogram Scale Chemical Synthesis of 2'-Fucosyllactose. *Carbohydr. Res.* **2019**, *476*, 71–77. https://doi.org/10.1016/j.carres.2019.03.006.

(8) Li, Z.; Gildersleeve, J. C. Mechanistic Studies and Methods To Prevent Aglycon Transfer of Thioglycosides. *J. Am. Chem. Soc.* **2006**, *128* (35), 11612–11619. https://doi.org/10.1021/ja063247q.

(9) Sabesan, S.; Neira, S. Synthesis of Glycosyl Phosphates and Azides. *Carbohydr. Res.* **1992**, *223*, 169–185. https://doi.org/10.1016/0008-6215(92)80015-S.

(10) Ermolenko, L.; Sasaki, N. A. Diastereoselective Synthesis of All Eight L-Hexoses from L-Ascorbic Acid. *J. Org. Chem.* **2006**, *71* (2), 693–703. https://doi.org/10.1021/jo0521192.

(11) Herde, Z. D.; John, P. D.; Alvarez-Fonseca, D.; Satyavolu, J.; Burns, C. T. Stereoselective Acetylation of Hemicellulosic C5-Sugars. *Carbohydr. Res.* **2017**, *443–444*, 1–14. https://doi.org/10.1016/j.carres.2017.03.008.

(12) Angles d'Ortoli, T.; Widmalm, G. Synthesis of the Tetrasaccharide Glycoside Moiety of Solaradixine and Rapid NMR-Based Structure Verification Using the Program CASPER. *Tetrahedron* **2016**, *72* (7), 912–927. https://doi.org/10.1016/j.tet.2015.12.042.

(13) Ma, T.; Li, C.; Liang, H.; Wang, Z.; Yu, L.; Xue, W. InBr3-Catalyzed Synthesis of Aryl 1,2-Trans-Thio(Seleno)Glycosides. *Synlett* **2017**, *28* (17), 2311–2314. https://doi.org/10.1055/s-0036-1588507.

(14) D'Accorso, N. B.; Thiel, I. M. E.; Schüller, M. Proton and C-13 Nuclear Magnetic Resonance Spectra of Some Benzoylated Aldohexoses. *Carbohydr. Res.* **1983**, *124* (2), 177–184. https://doi.org/10.1016/0008-6215(83)88453-5.

(15) Raposo, C. D.; Costa, R.; Petrova, K. T.; Brito, C.; Scotti, M. T.; Cardoso, M. M. Development of Novel Galactosylated PLGA Nanoparticles for Hepatocyte Targeting Using Molecular Modelling. *Polymers (Basel)* **2020**, *12* (1), 94. https://doi.org/10.3390/polym12010094.

(16) Lemaire, S.; Houpis, I. N.; Xiao, T.; Li, J.; Digard, E.; Gozlan, C.; Liu, R.; Gavryushin, A.; Diène, C.; Wang, Y.; Farina, V.; Knochel, P. Stereoselective C-Glycosylation Reactions with Arylzinc Reagents. *Org. Lett.* **2012**, *14* (6), 1480–1483. https://doi.org/10.1021/ol300220p.

(17) Casey, J. P.; Berry, D.; Castro, A.; Taylor, S. J.; Massari, F. E.; Prousfoot, J.; Bogart, E.; Briggs, T. F. Multibiotic Agents and Methods of Using the Same. WO2018226732A1, **2018**. https://patents.google.com/patent/WO2018226732A1/ (accessed 2024-05-30).

(18) Whistler, R. L.; Bemiller, J. N. *Methods in Carbohydrate Chemistry Volume VI General Carbohydrate Methods*; **1972**.

(19) Hunt, K. E.; García-Sosa, A. T.; Shalima, T.; Maran, U.; Vilu, R.; Kanger, T. Synthesis of 6'-Galactosyllactose, a Deviant Human Milk Oligosaccharide, with the Aid of Candida Antarctica Lipase-B. *Org. Biomol. Chem.* **2022**, *20* (23), 4724–4735. https://doi.org/10.1039/d2ob00550f.

(20) Fernandez-Lorente, G.; Palomo, J. M.; Cocca, J.; Mateo, C.; Moro, P.; Terreni, M.; Fernandez-Lafuente, R.; Guisan, J. M. Regio-Selective Deprotection of Peracetylated Sugars via Lipase Hydrolysis. *Tetrahedron* **2003**, *59* (30), 5705–5711. https://doi.org/10.1016/S0040-4020(03)00876-7.

(21) Palomo, J. M.; Filice, M.; Fernandez-Lafuente, R.; Terreni, M.; Guisan, J. M. Regioselective Hydrolysis of Different Peracetylated Β-Monosaccharides by Immobilized Lipases from Different Sources. Key Role of The Immobilization. *Adv. Synth. Catal.* **2007**, *349* (11–12), 1969–1976. https://doi.org/10.1002/adsc.200700055.

(22) Horrobin, T.; Tran, C. H.; Crout, D. Esterase-Catalysed Regioselective 6-Deacylation of Hexopyranose per-Acetates, Acid-Catalysed Rearrangement to the 4-Deprotected Products and Conversions of These into Hexose 4- and 6-Sulfates. *J. Chem. Soc. Perkin. 1* **1998**, No. 6, 1069–1080. https://doi.org/10.1039/a708596f.

(23) Filice, M.; Ubiali, D.; Fernandez-Lafuente, R.; Fernandez-Lorente, G.; Guisan, J. M.; Palomo, J. M.; Terreni, M. A Chemo-Biocatalytic Approach in the Synthesis of β-O-Naphtylmethyl-N-Peracetylated Lactosamine. *J. Mol. Catal. B Enzym.* **2008**, *52–53* (1–4), 106–112. https://doi.org/10.1016/j.molcatb.2007.10.013.

(24) Mayfield, A. B.; Metternich, J. B.; Trotta, A. H.; Jacobsen, E. N. Stereospecific Furanosylations Catalyzed by Bis-Thiourea Hydrogen-Bond Donors. *J. Am. Chem. Soc.* **2020**, *142* (8), 4061–4069. https://doi.org/10.1021/jacs.0c00335.

(25) Hennen, W. J.; Sweers, H. M.; Wang, Y. F.; Wong, C. H. Enzymes in Carbohydrate Synthesis. Lipase-Catalyzed Selective Acylation and Deacylation of Furanose and Pyranose Derivatives. *J. Org. Chem.* **1988**, *53* (21), 4939–4945. https://doi.org/10.1021/jo00256a008.

(26) Kováč, P.; Glaudemans, C. P. J. Stereocontrolled Synthesis of 6'-Deoxy-6'-Fluoro Derivatives of Methyl α-Sophoroside, α-Laminaribioside, α-Kojibioside and α-Nigeroside. *J Carbohydr. Chem.* **1988**, *7* (2), 317–335. https://doi.org/10.1080/07328308808058928.

(27) Zi, C.-T.; Yang, D.; Dong, F.-W.; Li, G.-T.; Li, Y.; Ding, Z.-T.; Zhou, J.; Jiang, Z.-H.; Hu, J.-M. Synthesis and Antitumor Activity of Novel Per-Butyrylated Glycosides of Podophyllotoxin and Its Derivatives. *Bioorg. Med. Chem.* **2015**, *23* (7), 1437–1446. https://doi.org/10.1016/j.bmc.2015.02.021.
